# Transcriptomic Responses of Atlantic Salmon (*Salmo salar*) to Environmental Enrichment during Juvenile Rearing

**DOI:** 10.1371/journal.pone.0118378

**Published:** 2015-03-05

**Authors:** Melissa L. Evans, Tiago S. Hori, Matthew L. Rise, Ian A. Fleming

**Affiliations:** 1 Department of Ocean Sciences, Ocean Sciences Center, Memorial University of Newfoundland, St. John’s, Newfoundland, Canada, A1C 5S7; Ghent University, BELGIUM

## Abstract

Captive rearing programs (hatcheries) are often used in conservation and management efforts for at-risk salmonid fish populations. However, hatcheries typically rear juveniles in environments that contrast starkly with natural conditions, which may lead to phenotypic and/or genetic changes that adversely affect the performance of juveniles upon their release to the wild. Environmental enrichment has been proposed as a mechanism to improve the efficacy of population restoration efforts from captive-rearing programs; in this study, we examine the influence of environmental enrichment during embryo and yolk-sac larval rearing on the transcriptome of Atlantic salmon (*Salmo salar*). Full siblings were reared in either a hatchery environment devoid of structure or an environment enriched with gravel substrate. At the end of endogenous feeding by juveniles, we examined patterns of gene transcript abundance in head tissues using the cGRASP-designed Agilent 4×44K microarray. Significance analysis of microarrays (SAM) indicated that 808 genes were differentially transcribed between the rearing environments and a total of 184 gene ontological (GO) terms were over- or under-represented in this gene list, several associated with mitosis/cell cycle and muscle and heart development. There were also pronounced differences among families in the degree of transcriptional response to rearing environment enrichment, suggesting that gene-by-environment effects, possibly related to parental origin, could influence the efficacy of enrichment interventions.

## Introduction

The environmental conditions experienced during development play a central role in determining phenotype, a phenomenon known as developmental phenotypic plasticity [1,2]. However, individual ability to match phenotype to environment may complicate conservation efforts for species raised in captivity for part or all of the life-cycle [3]. For instance, captive-rearing programs for salmonid fishes typically raise juveniles in egg trays or tanks during early life, in stark contrast to the conditions experienced in natural environments [4]. In the wild, female salmonids typically construct nests in gravel lining the substratum of a river, wherein their embryos develop over the winter. Embryos then hatch into yolk-sac larvae and continue to feed on endogenous resources while in the gravel nest before emerging as exogenously feeding juveniles in the spring [5]. Compared to juveniles reared under natural conditions, salmonid fishes reared in hatcheries may exhibit phenotypes, such as reduced risk sensitivity (anti-predator behavior), more aggressive food-seeking behaviors, and altered coloration, that are maladaptive outside of captivity [6–8]. Indeed, a growing number of studies have shown that captive-reared salmonids experience poor survival and reproductive success in the wild compared to fish reared in their natural environment [9–14].

Captive rearing and breeding program efficacy ultimately depends on their ability to produce individuals that will contribute to wild population productivity [4,15,16]. Alternative rearing methods, including the addition of structural enrichment to hatchery environments, so as to better mimic natural rearing conditions, represent a promising means of achieving this goal. Previous studies have demonstrated that exposure to enriched environment leads to increased shelter seeking behavior, learning ability, and dominance in juvenile salmon when compared to counterparts from unenriched environments [17,18]. Moreover, gross brain size and brain cell proliferation rates are generally lower in juvenile salmon reared in traditional compared to enriched hatchery environments [18–22]. Juveniles from enriched rearing environments are also less stressed, as estimated through basal cortisol levels, than those reared in unenriched environments [23]. Overall, these findings suggest that basic habitat enrichment via the addition of structural components typical of riverine environments leads to the development of behavioral, morphological, or physiological phenotypes that may improve juvenile performance upon their release to the wild.

Our research is aimed at understanding transcriptome-wide variation underlying the differential development of salmon following exposure to traditional and enriched hatchery environments. We were particularly interested in testing for an influence of environmental enrichment during embryo through yolk-sac larval incubation on the gene transcription profiles of neural/sensory systems, given that previous studies have documented effects of enrichment on brain morphology and juvenile behavior [18–20,22,23]. We reared Atlantic salmon (*Salmo salar*) full siblings (sibs) from each of three families in either a barren tank devoid of structure (unenriched environment), or a tank enriched with gravel substrate. At emergence, i.e. at the end of the endogenous feeding stage when the yolk-sac was used up, we characterized the whole-head transcriptome of juveniles reared in each of the environments using the Agilent 4×44K microarray (GEO accession: GSE25938) designed by the Consortium for Genomics Research on All Salmonids Project, cGRASP [24]. Our experimental design enabled the evaluation of gene transcription plasticity in response to differences in hatchery rearing environments and the identification of candidate genes and biological processes affected by habitat enrichment.

## Materials and Methods

### Crosses and experimental design

Three full sib Atlantic salmon family groups, hereafter referred to as family X11, X22, and X35 were generated using wild salmon from the Little River (47° 46' 29'' N, 55° 48' 52'' W) in Newfoundland, Canada, and Saint John River (New Brunswick) strain farmed salmon from marine net pens located on Newfoundland’s south coast, proximate to the Little River (Table 1). Farmed Atlantic salmon were included in this study due to logistical constraints associated with obtaining wild fish. South Newfoundland Atlantic salmon populations have declined in abundance by >40% since the mid-1990s [25]. The families were reared in the Miawpukek First Nation hatchery on the Little River until they reached the eyed stage (February 20, 2012), at which point they were collected and transported to the Ocean Sciences Centre (OSC) at Memorial University of Newfoundland. Each family was then split into two equal groups, with one group subsequently reared in the unenriched environment and the other buried in ~10cm of gravel (i.e. a total of six rearing containers were used, one tank per family per treatment). A total of 150 offspring from the X35 and X22 families and 105 offspring from the X11 family were introduced into each of treatments.

**Table 1:** Atlantic salmon family groups used in rearing experiments.

Family	Cross date	Dam origin	Sire origin
X11	Nov. 2, 2011	Wild, Little River, Newfoundland	Wild, Little River, Newfoundland
X22	Nov. 2, 2011	Wild, Little River, Newfoundland	Farmed, St. John River strain
X35	Nov. 9, 2011	Wild, Little River, Newfoundland	Farmed, St. John River strain

**Fig 1:**
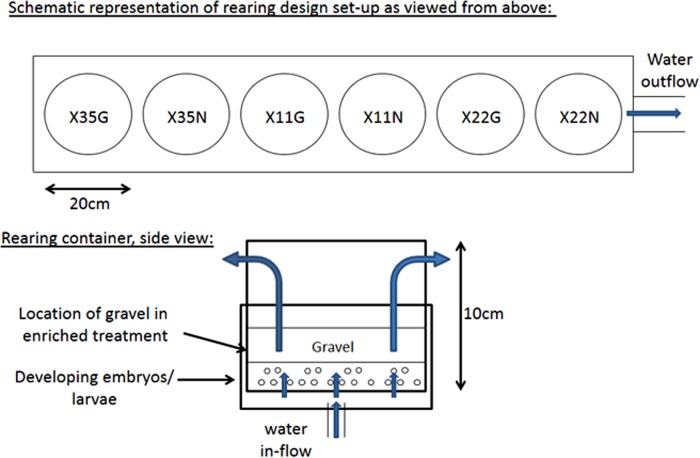
Schematic representation of rearing design used to expose Atlantic salmon juveniles from three families (X35, X11, X22) to a traditional hatchery environment i.e. unenriched, without gravel substrate (N), or a hatchery environment enriched with gravel (G). The experiment was implemented at the Ocean Sciences Centre, Memorial University of Newfoundland, St. John’s, Newfoundland, Canada, during the fall-winter 2011–2012.

We used plastic cylindrical buckets measuring 15 cm deep and 20 cm in diameter to rear the family groups. The bottom of each bucket was perforated with 5 mm holes and covered with mesh screening to allow water upwelling through the bucket. A series of 4 equally spaced 1.3 cm holes covered with mesh screening were located 13 cm above the container bottom to allow for continuous flow-through (Fig. 1). Each incubation container sat within a similarly sized bucket having a water inlet tube entering the center bottom. There was a 4 cm deep space between the perforated bottom of the incubation container and that of the bucket that housed it. All containers were covered with lids to ensure similarly dark environments during incubation. Ambient flow-through water (sand filtered but otherwise untreated) from the local Sugarloaf Brook system was provided at a rate of 1.9L/min to each incubation chamber.

At emergence (i.e. when juveniles emerged from the gravel substrate, which coincides with the start of exogenous feeding in nature), during April 23–30 2012, we terminated the experiment, and haphazardly collected a total of five “unenriched” and five “enriched” juveniles from each of the three families (*N* = 15 unenriched juveniles; *N* = 15 enriched juveniles, in total). Since juveniles from the unenriched environment were reared without gravel substrate, we utilized the families reared in the enriched treatment to assess emergence time. Emergence occurred at ca. 745–785 accumulated degree-days. The juveniles were euthanized using 300mg/L buffered MS-222 (Sigma-Aldrich, Oakville, ON, Canada) and weighed and measured for fork-length. Heads were then severed from the body, directly posterior to the operculum, immersed in RNAlater, and incubated at 4°C overnight (~40mg tissue:1.5mL RNAlater; Life Technologies Inc., Burlington, ON, Canada). The samples were subsequently stored at -80°C. By assessing gene transcription profiles in whole heads, we ensured that all regions of the brain were included in our sample (Fig. A in S1 File), while also reducing the contribution of skeletal muscle transcripts, which are likely to predominate in samples of whole larvae undergoing rapid development and growth [26,27]. Besides brain, other structures including the eyes and heart were included in the sample (Fig. A in S1 File). All experiments were conducted in strict accordance with guidelines provided by the Canadian Council on Animal Care and were approved by Memorial University’s Animal Care Committee (protocol 12–18-IF).

### RNA isolation, amplification, and labelling

Whole heads were removed from RNAlater, blotted dry, and then homogenized using Kontes’ disposable pestles and a hand-held homogenizer (Thermo Fisher Scientific, Wilmington, DE, USA) in 1.5mL TRI Reagent (Sigma-Aldrich). Total RNA was isolated following the manufacturer’s instructions. Isolated RNA was treated with RNAse-free DNase-I (QIAGEN, Mississauga, ON, Canada) and purified using the RNeasy MinElute kit (QIAGEN), according to the manufacturer’s instructions. We assessed RNA quantity/purity on a NanoDrop 1000 (Thermo Fisher Scientific); 260/280 and 260/230 ratios averaged 1.98 (range: 1.82–2) and 2.17 (range: 1.9–2.35), respectively. RNA isolates were also visualized on 1% agarose gels stained with ethidium bromide (Life Technologies Inc.) to ensure integrity of the 18S and 28S ribosomal RNA bands.

Anti-sense RNA (aRNA) was amplified and fluorescently-labelled using the Amino Allyl MessageAmp^TM^ II aRNA amplification kit (Ambion, Life Technologies Inc.) and Cy5 and Cy3 mono-reactive dyes (GE Healthcare, Mississauga, ON, Canada), according to the manufacturer’s instructions. One μg of total RNA was used as starting material for the aRNA amplifications. Twenty μg of each “experimental” aRNA sample (i.e. from each of the head samples) was then labelled with Cy5 dye. A “reference” sample composed of equal contributions of each of the total RNA samples was also amplified, and 20 μg of reference aRNA was labelled with Cy3 dye; also see [28]. Following labelling, the samples were again measured on the NanoDrop 1000 to determine final aRNA concentration and the efficiency of dye incorporation.

### Microarray hybridization and experiments

The head transcriptomes of juveniles reared in enriched and unenriched environments were compared using the Agilent 4×44K salmonid microarray (Agilent Technologies Inc., Mississauga, ON, Canada) designed by cGRASP [24,29]. We used a reference design to obtain estimates of mRNA transcript abundance for each individual based on hybridization to the microarray; each Cy5-labelled experimental aRNA sample was pooled with the Cy3-labelled reference sample and then hybridized to the array. The hybridization level (i.e. fluorescent signal) of each experimental sample was then evaluated as a ratio relative to the common Cy3-labelled sample [30]. The hybridization mixture for each array was prepared using Agilent’s Gene Expression Hybridization Kit, according to the manufacturer’s instructions (Agilent Technologies Inc.). Hybridizations were performed overnight in a microarray hybridization oven at 65°C which oscillated at 10 revolutions per minute (RPM). Following hybridization, arrays were washed and dried following the manufacturer's instructions.

### Microarray data acquisition and analysis

The microarrays were scanned at 5μm resolution using the ScanArray Gx Plus scanner and ScanExpress v4.0 software (Perkin-Elmer Life Sciences, Waltham, MA, USA). Scanning protocols followed [28]. TIFF images for each channel (Cy5, Cy3) were exported from ScanExpress and signal intensity data extracted from the images using Imagene v9.0 (BioDiscovery, El Segundo, CA, USA). Poor quality spots on each array were manually flagged in Imagene.

We used the Bioconductor package “mArray” as implemented in R to remove flagged spots from the dataset and log_2_-transform and Loess-normalize signal intensity data for each of the 44K grids on a microarray slide. As in [31], probe signal intensities that were lower (undetected signal) than the average of the median background signal ±2 SD were removed and marked as “N/A”. R scripts were adapted from those described in [31] and are reported in the S1 File.

We used Significance Analysis of Microarrays (SAM; [32]), as implemented in R in the “siggenes” package [33], to test for differing patterns of gene transcript (mRNA) abundance in juveniles from the two rearing environments. Analyses conducted in SAM incorporated a permutation-based correction for multiple hypothesis testing using the false discovery rate (FDR) [32]. Our results are reported at a FDR = 5% except where indicated. The SAM analysis excluded probes for which a signal was undetected on more than 25% of the arrays examined (i.e. 8 of 30 arrays; see [28]). This resulted in a final dataset consisting of 21,117 microarray probes (of the microarray containing ~44K probes). We then used the EM array method of the LSimpute algorithm to impute any missing values for the retained probes [34,35]. Again, detailed R scripts were adapted from those reported in [31] and are reported in the S1 File.

Our experiment includes two factors that could influence patterns of gene transcript abundance: rearing environment (treatment) and family (and the tank it was reared in), with a sample size of five juveniles from each family/treatment combination. A comparison of analysis methods for microarray data suggested that datasets with sample sizes of five or fewer are poorly classified using ANOVA-based methods [36]. Thus, we elected to investigate patterns of gene transcription in juveniles from our two rearing environments in SAM in two ways: first, we compared the transcription profiles of all 15 juveniles from the enriched to the 15 juveniles from the unenriched environment, regardless of family, in a single analysis. Second, the gene transcription profiles of juveniles from the enriched and unenriched environments within each of the families were compared.

Following identification of a candidate list of responsive genes in SAM, we used hierarchical clustering, implemented in Genesis [37], to visually examine similarities in transcription profiles among juveniles. Relative gene transcript abundance estimates were median-centered in Genesis, and then clustered using Pearson correlations across experiments (i.e. each juvenile = experiment). This gene list was also used as the basis of Gene Ontology (GO) term enrichment analysis, conducted in Blast2GO [38]. We annotated our gene list (test set) using a BLASTx alignment of the array’s expressed sequence tags/contigs against the NCBI nr database and mapped the results to GO terms. In Blast2GO we utilized a minimum expect (i.e. threshold) value (E-value) of 10^-6^, and the default HSP cut-off of 33 to identify significant GO terms. A Fisher’s exact test was then used to examine whether GO terms in our test set were over- or underrepresented compared to the representation of GO terms found on the 44K microarray (reference set) following annotation using human terms. We elected to compare our significantly differentially transcribed genes to annotated genes on the entire array because we were interested in identifying overrepresented functions in head tissues compared to the suite of all possible biological functions represented on the array [39]. We identified significant GO terms using a FDR < 5%.

Finally, principal components analysis (PCA) was also used to partition variation in gene transcription among juveniles at all 21,117 retained microarray probes. The PCA was run in the R package ade4TkGUI [40].

### Morphological variation

The body mass of juveniles conformed to a normal distribution (Shapiro-Wilk W: P > 0.05) so we used analysis of covariance (ANCOVA) to examine the influence of family and rearing environment on juvenile body mass. The model included fork-length as a covariate. Tests of normality and ANCOVA were conducted in JMP v. 10 (SAS Institute, Cary, NC) with a critical α = 0.05.

## Results

### Morphological variation and rearing environment

**Fig 2:**
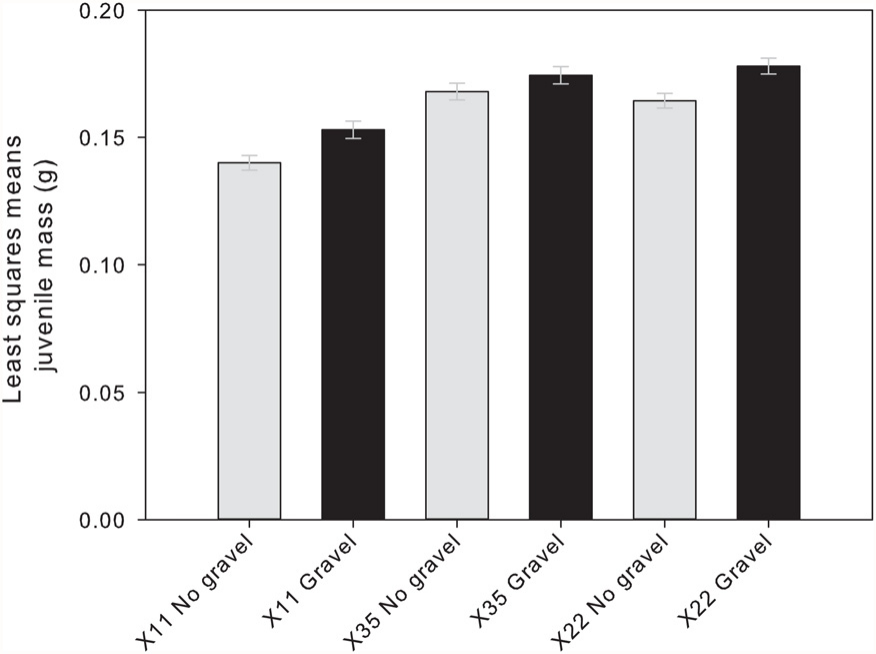
Body mass of juvenile Atlantic salmon from each of three families reared in an unenriched hatchery environment (grey bars) or a hatchery environment enriched with gravel (black bars). Body mass is shown as a least squares means (LSM) estimate derived from ANCOVA, which included fork length as a covariate in the model. Error bars show LSM body mass ± 1 SE.

Juveniles from the enriched environment were significantly heavier than their full sibs reared in the unenriched environment, regardless of family (*P* = 0.0002; Table 2, Fig. 2). We also observed a significant effect of family on body mass: juveniles from the X35 and X22 families were significantly heavier than the X11 juveniles (*P* < 0.0001; Table 2, Fig. 2).

**Table 2:** Results of ANCOVA examining the influence of family and rearing environment on Atlantic salmon juvenile body mass. Fork length was included in the model as a covariate. The null hypothesis that each model factor’s effect on body mass was zero was tested with the F-statistic. Model and error degrees of freedom (DF) are indicated for the model as are DF for each factor. P-values falling below the critical α (0.05) are bolded.

		DF	F	P
Model		6,23	36.34	<**0.001**
	Length	1	22.68	<**0.001**
	Family	2	38.03	<**0.001**
	Rearing environment	1	19.76	**<0.001**
	Family × rearing environment	2	0.09	0.341

### Transcriptional response to rearing environment

A total of 808 genes were significantly differentially transcribed by juveniles reared in the unenriched versus enriched environments (FDR of 5%; gene list and fold-changes reported in Table A in S1 File). Of these genes, 674 of 808 (83%) were successfully annotated using BLASTx. The majority of the genes responded similarly across the three families, with most genes relatively upregulated in juveniles from the unenriched environment (Table 3, Table A in S1 File). The top 10 differentially up- and downregulated genes, ranked by fold-change (enriched/unenriched environment), are shown in Table 3. The top upregulated gene in the enriched environment was protein-glutamine gamma-glutamyltransferase e-like (8.6-fold), whereas the top downregulated gene was myosin light polypeptide 4 (16.7-fold; Table 3).

**Table 3:** List of the top ten differentially transcribed genes (for overall fold-change) in Atlantic salmon reared in each of the enriched and unenriched hatchery environments. The analysis of relative gene transcript abundance among juveniles from each of three families (X11, X22, X35) was performed using the cGRASP-designed 4×44K microarray. Significantly differentially transcribed genes were identified using a FDR = 5%. Fold-changes (enriched/unenriched) in gene transcript abundance are shown across all families and separately for each of the three families. Only genes that were successfully annotated using BLASTx are shown. Up to five GO terms associated with each gene are also indicated.

	Probe ID	Best BLASTx hit^a^	All families fold-change^b^	Fold-change(X11)	Fold-change(X22)	Fold-change(X35)	GO terms^c^
Relatively upregulated enriched	C067R138	protein-glutamine gamma-glutamyltransferase e-like	8.55	4.21	20.72	2.21	Peptide cross-linking (P); protein-glutamine gamma-glutamyltransferase activity (F); metal ion binding (F)
	C204R103	cytochrome p450 2m1	4.23	5.37	4.99	3.06	Heme binding (F); iron ion binding (F); oxidoreductase activity (F); organic acid metabolic process (P); small molecular metabolic process (P)
	C237R040	PREDICTED: hypothetical protein LOC324610	2.87	3.40	2.66	2.58	-
	C205R145	claudin-10-like	2.41	3.92	1.38	2.48	Structural molecule activity (F); cell junction (C); plasma membrane (C)
	C211R110	complement receptor type 1-like	2.26	1.99	2.87	2.12	Receptor activity (F)
	C051R138	hypothetical protein MTR_5g050970	2.05	2.22	1.92	2.09	-
	C211R161	metalloproteinase inhibitor 2 precursor	2.05	2.76	2.51	1.32	Metalloendopeptidase inhibitor activity (F); metal ion binding (F); negative regulation of endopeptidase activity (P); extracellular region (C)
	C026R035	protein phosphatase 1 regulatory subunit 14b	1.90	2.82	1.43	1.52	Regulation of phosphorylation (P); protein phosphatase inhibitor activity (F); protein phosphatase type 1 regulator activity (F)
	C001R030	metalloproteinase inhibitor 2 precursor	1.89	2.12	2.02	1.60	Metalloendopeptidase inhibitor activity (F); metal ion binding (F); negative regulation of endopeptidase activity (P); extracellular region (C)
	C155R133	gtpase imap family member 7	1.87	2.20	1.40	2.49	GTP binding (F); biological process (P); cellular component (C)
Relatively upregulated unenriched	C152R152*	ependymin-1 precursor	0.46	0.20	0.57	0.69	Cell-matrix adhesion (P); calcium ion binding (F); extracellular region (C)
	C147R072	asph protein	0.46	0.25	0.51	0.78	Negative regulation of cell proliferation (P); face morphogenesis (P); limb morphogenesis (P); endoplasmic reticulum membrane (C); oxidoreductase activity (F)
	C228R108	ttd non-photosensitive 1 protein homolog	0.46	0.25	0.49	0.95	None
	C233R135	myosin heavy chain	0.45	0.33	0.68	0.46	Metabolic process (P); muscle cell development (P); motor activity (F); myosin complex (C); plasma membrane (P)
	C211R037	ubiquitin thioesterase partial	0.44	0.23	0.43	0.79	Cell surface (C)
	C113R135	regulator of chromosome condensation	0.44	0.23	0.60	0.75	Nuclear membrane (C); spindle assembly (P); nucleosomal DNA binding (F); mitosis (P); regulation of S phase of mitotic cell cycle (P)
	C020R131	phosphatidylinositol transfer protein beta isoform isoform 2	0.31	0.22	0.46	0.42	Lipid binding (F); in utero embryonic development (P); phosphatidylinositol transported activity (F); Golgi apparatus (C); phospholipid binding (F)
	C176R119	apolipoprotein a-i precursor	0.15	0.16	0.07	0.59	Lipid metabolic process (P); lipid transport (P); cholesterol metabolic process (P); cholesterol transport (P); lipid binding (F)
	C251R080	myosin regulatory light chain atrial isoform	0.10	0.06	0.12	0.13	Calcium ion binding (F); dendritic spine (C); heart morphogenesis (P); atrial cardiac myofibril assembly (P); cardiac muscle tissue development (P)
	C086R103	myosin light polypeptide 4	0.06	0.04	0.08	0.08	Calcium ion binding (F); sarcomere organization (P); heart morphogenesis (P); ventricular cardiac myofibril assembly (P); cardiac muscle tissue development (P)

^a^Significantly differentially transcribed genes were annotated in Blast2GO using the BLASTx algorithm; the best BLASTx hit (E-value < 10^-6^) is presented.

^b^ Fold-changes are presented as output from siggenes.

^c^Up to five examples of GO terms mapped to each microarray probe in Blast2GO are indicated. The associated GO category, biological process (P), molecular function (F), and cellular component (C), is also indicated for each term in parentheses.

*While this gene was initially annotated as ependymin-1, further analyses suggest that contig C152R152 is chimeric (data not shown).

**Fig 3:**
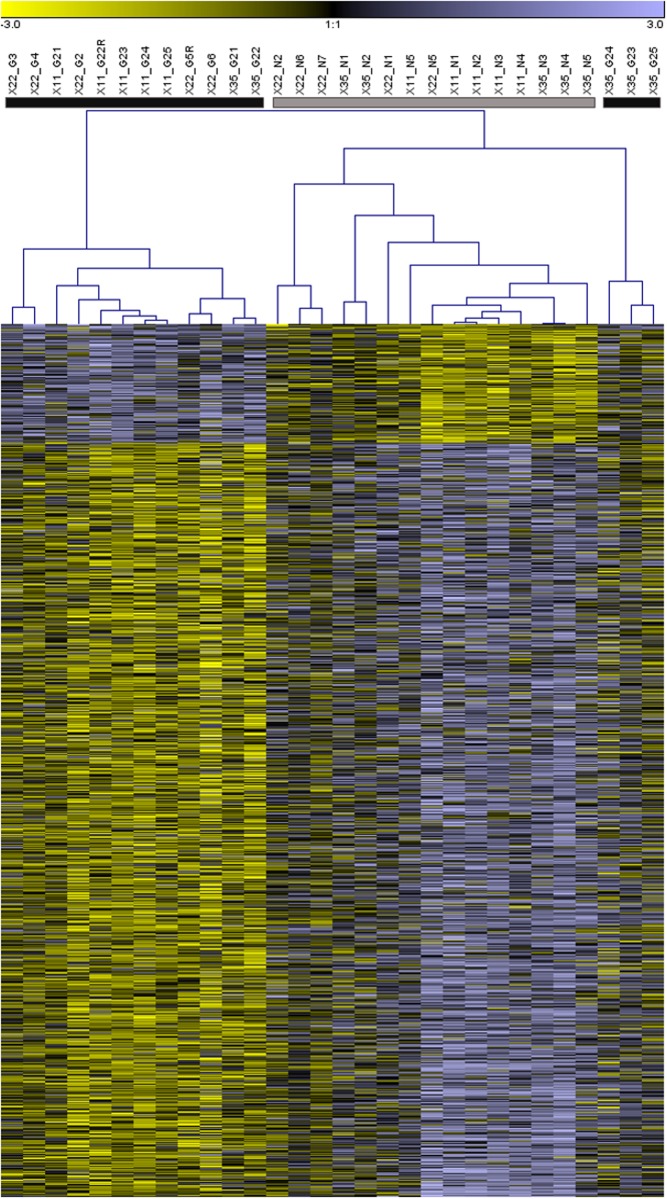
Hierarchical clustering of Atlantic salmon gene transcription profiles following juvenile rearing in unenriched and enriched hatchery environments. The heatmap depicts clustering of all 808 differentially transcribed genes, with the transcription profile of each individual represented by a column in the map. Grey and black color bars located above the columns correspond to individuals reared in unenriched and enriched environments, respectively. Above these bars, each profile is also labeled by family (X11, X22, X35), whether the individual was reared with gravel (G) or without gravel (N), and by individual identification number. For a given gene (i.e. row in heatmap) higher and lower gene transcript abundance, relative to the common reference sample, is indicated in purple and yellow, respectively.

The hierarchical clustering analysis revealed three main clusters of individuals with similar transcription profiles. Individuals from each of the unenriched and enriched environments predominantly clustered together, except for three “enriched” individuals from family X35 that clustered together and more closely to juveniles from the unenriched environment (Fig. 3). The cluster analysis also demonstrated that most of the differentially transcribed genes were relatively upregulated in juveniles from the unenriched environment (Fig. 3, Table A in S1 File).

### GO term enrichment in differentially transcribed genes

Within the list of 808 differentially transcribed genes, a total of 184 GO terms were significantly over- or underrepresented. The majority of the terms, 115 of 184 (63%), were associated with biological processes, including the terms “muscle cell development”, “muscle cell differentiation”, “cardiac myofibril assembly”, “M phase of mitotic cell cycle”, “mitosis”, and “cell cycle phase”, which were represented by more than 30 microarray features each (Table B in S1 File). We have summarized a selection of enriched GO terms associated with muscle, heart, cell cycle, and mitosis in Table 4. In Table C in the S1 File, we have also included a list of the 122 genes associated with the GO term “cell cycle” and the 63 genes associated with the term “heart development”. Fold-changes in transcript abundance at the genes annotated to “cell cycle” and “heart development” were similar among families in response to rearing environment (Table C in S1 File).

**Table 4:** Subset of overrepresented Gene Ontology (GO) terms linked to the biological processes mitosis/cell cycle and heart development. Overrepresented GO terms were identified through enrichment analysis, conducted in Blast2GO, of the 808 genes differentially transcribed by juvenile Atlantic salmon reared in unenriched and enriched hatchery environments. The total number of genes (i.e. microarray features) associated with each of the GO terms is indicated. Significance of the Fisher’s exact test (FDR < 5%) of GO term overrepresentation is also shown.

GO ID	GO Term	FDR	No. Genes	Representation
GO:0048738	cardiac muscle tissue development	7.02E-09	38	over
GO:0055003	cardiac myofibril assembly	1.24E-08	18	over
GO:0000279	M phase	1.38E-07	66	over
GO:0007507	heart development	2.49E-07	63	over
GO:0055013	cardiac muscle cell development	2.63E-07	19	over
GO:0055006	cardiac cell development	3.35E-07	20	over
GO:0022402	cell cycle process	9.22E-07	106	over
GO:0000087	M phase of mitotic cell cycle	1.2E-06	50	over
GO:0022403	cell cycle phase	1.31E-06	90	over
GO:0007067	mitosis	1.02E-05	46	over
GO:0000280	nuclear division	1.03E-05	46	over
GO:0000278	mitotic cell cycle	1.42E-05	86	over
GO:0060048	cardiac muscle contraction	2.08E-05	19	over
GO:0007049	cell cycle	5.29E-05	122	over
GO:0003015	heart process	6.35E-05	31	over
GO:0060047	heart contraction	6.35E-05	31	over
GO:0007059	chromosome segregation	6.35E-05	25	over
GO:0055007	cardiac muscle cell differentiation	0.00011	19	over
GO:0003007	heart morphogenesis	0.012801	30	over

**Fig 4:**
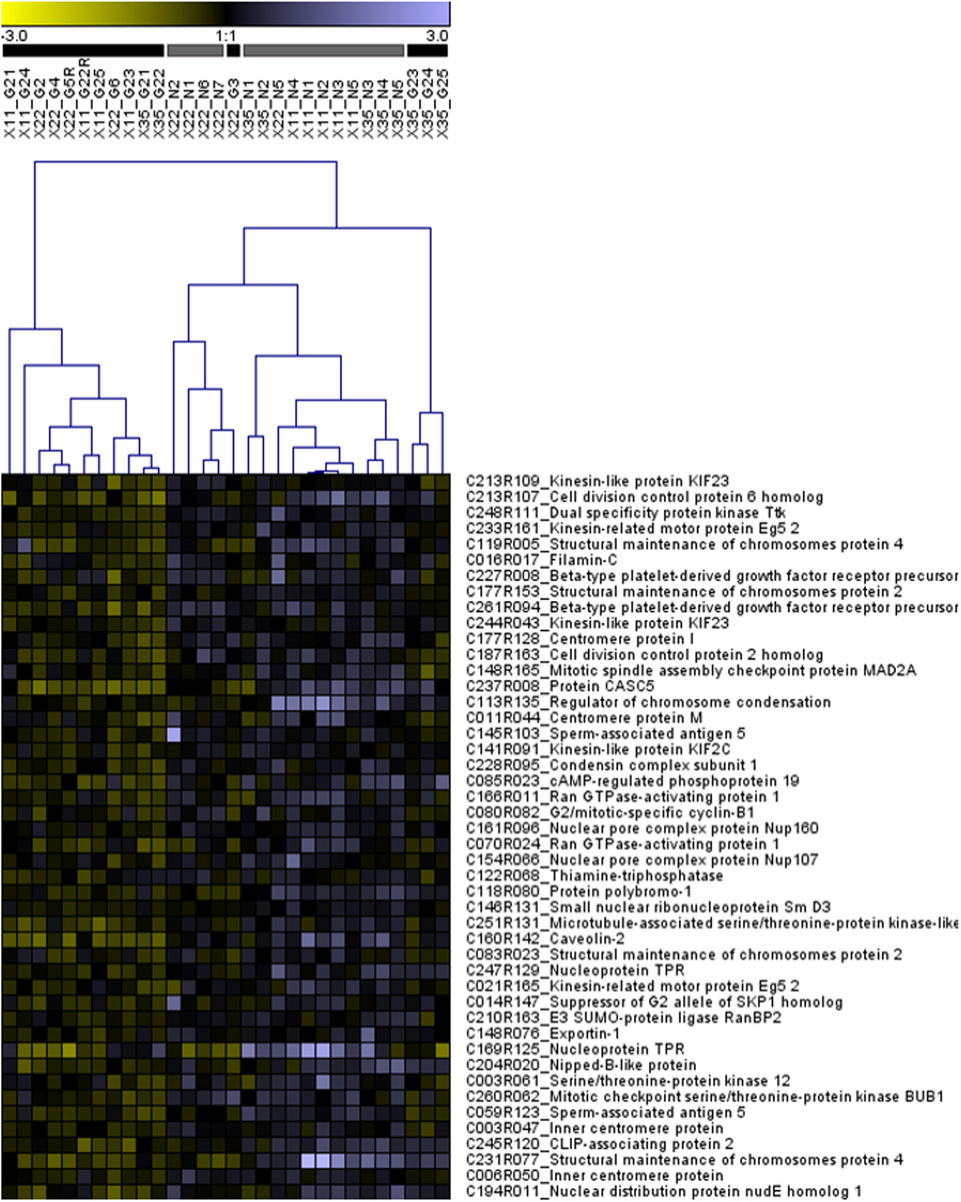
Hierarchical clustering of 46 genes associated with the GO term “mitosis” that were differentially transcribed by salmon juveniles reared in enriched and unenriched hatchery environments (also see Table C in S1 File). Grey and black color bars located above the columns correspond to individuals reared in unenriched and enriched hatchery environments, respectively. Above these bars, each profile is labeled by family (X11, X22, X35), whether the individual was reared in the enriched “gravel” (G) or unenriched “no gravel” (N) treatment, and by individual identification number. For a given gene (i.e. row in heatmap) higher and lower gene transcript abundance, relative to the common reference sample, is indicated in purple and yellow, respectively.

Given the over-representation of GO terms associated with mitosis in our gene list, we conducted a separate hierarchical clustering analysis of all 46 genes associated with the “mitosis” GO term (Table 4, Fig. 4). As in the analysis of all 808 genes, three major clusters of juveniles formed; the first cluster was entirely comprised of juveniles reared in the enriched environment and the second was predominantly comprised of juveniles reared in the unenriched environment, except for one “enriched” juvenile from family X22 that clustered with the latter group (Fig. 4). The third cluster comprised the same three “enriched” juveniles from family X35 that formed a separate cluster in the analysis of all 808 differentially transcribed genes (Fig. 4).

In addition to biological processes, 45 GO terms associated with cellular components were highly represented (i.e. > 30 microarray features) in our gene list. Several were linked to muscle cell components, including “sarcomere”, “myofibril”, and “contractile fiber” (Table B in S1 File). Twenty-four GO terms were associated with molecular function including “structural constituent of muscle”, “protein kinase C binding”, and “structural molecule activity”.

### Gene transcription response across families

Our independent analyses of the three families revealed considerable variation in the degree of gene transcription response to rearing environment. Families X22 and X11 showed 17 and 3445 significantly differentially transcribed genes (FDR < 5%), respectively, and only three genes in common: metalloproteinase inhibitor 2 precursor (Probe ID = C211R161), cd276 antigen (Probe ID = C010R015), and T-cell surface glycoprotein cd5-like (Probe ID = C260R032). Metalloproteinase inhibitor 2 precursor was relatively upregulated, whereas cd276 antigen and t-cell surface glycoprotein cd5-like were relatively downregulated under the enriched conditions (Table D in S1 File). A full list of differentially transcribed genes in each of the three families is reported Table D in S1 File.

The gene transcription patterns of X35 juveniles reared under enriched and unenriched hatchery environments did not differ significantly at our threshold FDR of 5%. However, 13 genes were marginally significantly differentially transcribed (FDR = 8%) by juveniles from the two rearing environments, and of these genes, six were also identified as differentially transcribed by juveniles from family X11 (Table A, D in S1 File). These genes included c4b-binding protein alpha chain precursor (Probe ID = C211R110; upregulated in enriched environment), sperm-associated antigen 5 (Probe ID = C059R123; downregulated in enriched environment), dep domain containing protein 1a isoform 2 (Probe ID = C153R058; downregulated in enriched environment), heterogeneous nuclear ribonucleoprotein l (Probe ID = C018R137; downregulated in enriched environment), prostaglandin E synthase 3 (Probe ID = C237R046; downregulated in enriched environment), and a hypothetical protein LOC324610 (Probe ID = C237R040; upregulated in enriched environment; Table A, D in S1 File). There were no (marginally significantly) differentially transcribed genes in common between families X35 and X22.

In all three families, the majority of the differentially transcribed genes were relatively downregulated in the juveniles from the enriched environment compared to juveniles from the unenriched environment. In families X22 and X35, only 1 of 17 (6%) and 3 of 13 (23%) differentially transcribed genes were upregulated in juveniles reared in the enriched environment (Table D in S1 File). For family X11, 1402 of 3445 (41%) differentially transcribed genes were upregulated in juveniles from the enriched environment (Table D in S1 File).

**Fig 5:**
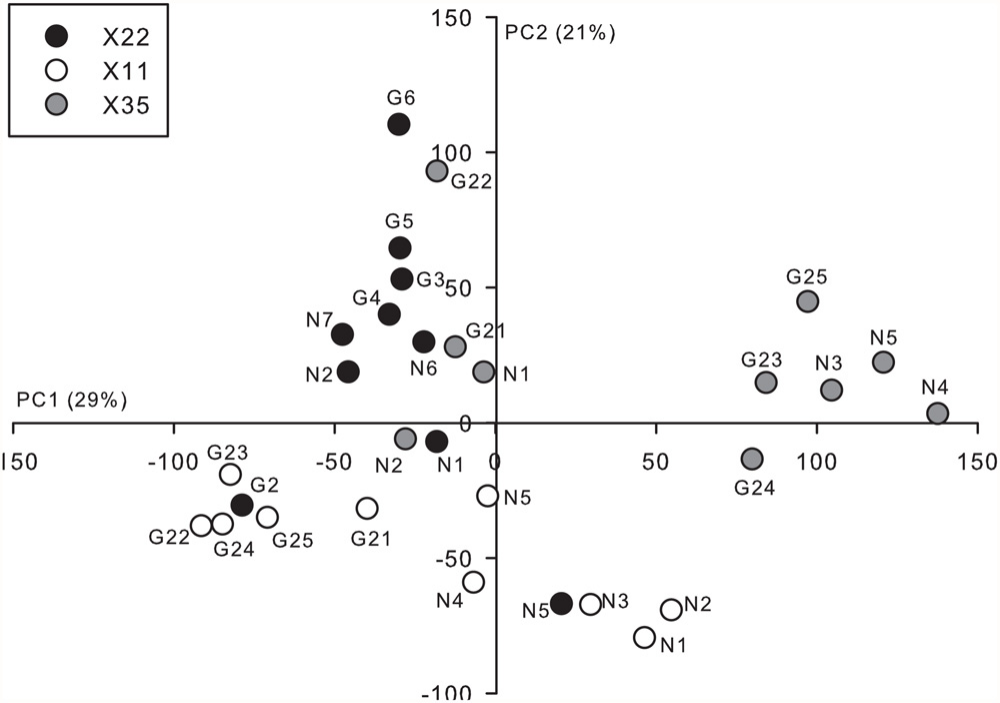
Principal components analysis of the head transcription profiles of Atlantic salmon juveniles exposed to either an unenriched or enriched hatchery rearing environment during early development. Principal component (PC) scores for individuals from family X35, X11, and X22, are shown by the grey, open, and black circles, respectively. The points are also labeled by individual ID and whether they were reared in a hatchery environment with (G) or without (N) the addition of gravel substrate for environmental enrichment. PC axes 1 and 2 explained 29% and 21% of the respective variation in relative gene transcript abundance observed among the juveniles.

The first two axes arising from the PCA explained 50% (PC1 = 29%, PC2 = 21%) of the variation in gene transcript abundance observed among juveniles from the three families (Fig. 5). There appeared to be greater variation among individuals from family X35 compared to families X11 and X22. Specifically, six X35 juveniles grouped into their own cluster, exhibiting relatively high scores along PC1; the other four juveniles grouped together near the origin of PC1 and PC2. The cluster of X35 individuals with high PC1 scores was composed of three “unenriched” and three “enriched” juveniles and thus, does not appear to be related to rearing environment. It is possible that this high degree of intra-family variation could have limited our ability to detect relatively smaller changes in gene transcript abundance related to rearing environment. In contrast to the patterns observed for family X35, the transcription patterns of individuals from families X11 and X22 were relatively continuously distributed along the component axes. Interestingly, for family X11, all five juveniles reared in the enriched environment clustered together. There also appeared to be some separation of X22 juveniles reared in the unenriched and enriched environments along PC2 (Fig. 5).

## Discussion

In this study, we utilized a powerful split-family breeding design to examine the influence of environmental enrichment on juvenile Atlantic salmon growth and gene transcription. Following several weeks of exposure to a traditional or experimentally enriched hatchery environment, we observed clear differences in juvenile size; juveniles from the enriched environment were heavier, even after controlling for body length, than their full sibs from the unenriched environment. Previous studies of salmonids have also demonstrated differences in growth and condition related to environmental enrichment, likely driven by the differing energetic requirements of juveniles reared with and without structure [18,41,42]. Though we were unable to investigate activity levels as part of this study, the absence of structural support in the unenriched environment may have led to more frequent swimming by juveniles in order to maintain vertical stability [42–44], potentially decreasing the efficiency of yolk-sac utilization [45].

In contrast to their relatively consistent growth responses to rearing environment, the three families responded distinctly at the gene transcription level. One of the families (X11) exhibited an extreme response to rearing environment, with 16% (3,445/21,117) of genes showing significantly different transcription patterns in full sibs reared in the unenriched compared to enriched environment. Full sibs from the other two families (X22 and X35) exhibited relatively few significantly differentially transcribed genes (< 20) when reared in the different environments. We did not design our experiment to formally test for an influence of parental origin or population on patterns of gene transcription in developing salmon. Each family was reared in a single enriched and unenriched replicate, and thus it is not possible to tease apart family from replicate effects. However, the use of farmed (domestic) × wild salmon versus wild-only fish in the weakly (X22 and X35) and strongly responsive crosses (X11), respectively, points to a potential role for genetic differences between wild and farmed populations as a driver of transcriptomic response to rearing environment [46–50]. Domesticated salmon have undergone artificial (directional) selection over multiple generations for marketable traits, such a large body size, which is likely to alter gene expression profiles [4,47,51,52], and indeed the two families derived from farm origin fathers (X22 and X35) grew more compared to the offspring of wild origin male and female parents, regardless of rearing environment. The influence of population/parental effects on the gene transcription responses of salmon to environmental enrichment requires further exploration under a more formal quantitative genetic framework. However, our results preliminarily suggest that genotype × environment effects influence the head transcriptome of developing salmon, and from an applied perspective, suggest that these effects may impact the efficacy of environmental enrichment programs during captive-rearing.

It is also possible that apparent differences in gene transcription response to rearing environment among families are, in part, a consequence of low power related to limited sample sizes. Small sample sizes and the issue of low power are common in microarray studies, as they are costly and laborious to conduct [36]. By analysing the transcription profiles of all 30 juvenile salmon in a single analysis we were able to evaluate a generalized response to rearing environment. Overall, the combined analysis of all three families identified 808 genes with altered transcription patterns between rearing environments. The gene transcription patterns of juveniles reared in the enriched compared to unenriched environments are reported for each of the three families in Table A in the S1 File and Fig. 3, both of which demonstrate a generally consistent response by the families to rearing environment.

Within this gene list, our GO term enrichment analysis identified 184 terms over-, or less commonly, underrepresented in the head transcriptome. Among the top overrepresented terms, based on highly significant FDRs and number of differentially transcribed genes (i.e. > 30 microarray features) associated with each term, were those associated with muscle cell growth and development (e.g. sarcomere, myofibril) and cardiac muscle/heart development. Also, several of the most overexpressed genes in juveniles from unenriched environments were associated with muscle/heart/limb development (e.g. myosin heavy chain, asph protein, myosin regulatory light chain atrial isoform, myosin light polypeptide 4) and face and limb morphogenesis (asph protein). While the head tissues were isolated in a consistent manner from salmon from each of the rearing environments, it is possible that differences the size or developmental stage of salmon (and associated allometric effects) from each of the rearing environments could contribute to differences in muscle and organ tissue-associated transcripts in head tissues.

However, several other genes associated with growth and development were found to be differentially transcribed in the head tissues of juveniles from the enriched and unenriched rearing environments. A gene associated with lipid metabolism (apolipoprotein a-i precursor) was relatively upregulated by juveniles from the unenriched environment, suggesting that environmental enrichment leads to differing lipid utilization by salmon juveniles [53]. Also, two microarray features annotated to metalloproteinase inhibitor 2 precursor (also known as tissue inhibitor of matrix metalloproteinase 2) were relatively upregulated by juveniles from the enriched environment. Tissue inhibitor of matrix metalloproteinase 2 (TIMP2) is transcribed in a variety of tissues in fishes, including brain, blood, and muscle [54]. TIMP2’s encoded protein inhibits the activity of matrix metalloproteinases, proteinases that degrade extracellular matrix macromolecules i.e. the building blocks of the cellular environments produced during development and morphogenesis [55]. It is therefore possible that the relative upregulation of TIMP2 in salmon from enriched environments facilitates investment into growth and underlies altered tissue turnover and remodelling compared to juveniles from the unenriched environment. These results point to multiple genomic regions potentially underlying altered growth and morphological variation exhibited by salmonid juveniles from enriched and unenriched hatchery environments [53].

The fish brain, particularly during development, can be highly responsive (plastic) in the face of environmental variation [18,21,22,56]. A gene that was preliminarily annotated as ependymin-1 precursor was one of the top upregulated genes in juveniles from the unenriched environment, although further bioinformatic analysis showed this microarray feature to be a potential chimera (data not shown). Nevertheless, five other microarray features annotated to ependymin were differentially transcribed by juveniles from family X11 (Table S1), most relatively upregulated (C027R097, C175R070, C005R058, C210R115) and one downregulated (C015R062) in the enriched environment. Ependymin proteins are produced in the meningeal cells of teleosts [57], and their expression in fish brains has been linked to aggressive behaviors [57], acclimation to cold and dark environments [58,59], and long term memory [59]. Differential ependymin gene transcription may therefore reflect behavioral modification or acclimation to physical differences in enriched and unenriched environments. Additional qPCR-based experiments are planned to investigate the ubiquity of differential ependymin gene paralog regulation in response to differences in hatchery rearing environments.

Previous work in nine-spine stickleback (*Pungitius pungitius*) and Atlantic salmon has shown that individuals reared in enriched compared to unenriched environments develop larger optic tecta in the brain [18,60]. Moreover, Atlantic salmon from enriched environments have been shown to upregulate a gene linked to neurogenesis [22]. In this study, we found that two genes encoding for myelin and lymphocyte proteins (MAL)—which, in mammals, are ubiquitously expressed by Schwann and oligodendrocyte cells and are involved in the maintenance of the myelin sheath in the central and peripheral nervous system [61]—were relatively upregulated in juvenile salmon from the enriched environments. We also observed differences in transcript abundance between juveniles from enriched and unenriched environments at several other genes associated with brain development: e.g. schwannomin-interacting protein 1-like, cerebellin, and neuritin, suggesting that multiple genomic regions associated with brain development are affected by environmental enrichment. Future work is needed to determine whether these altered gene transcription patterns are conserved throughout development and/or possibly translate into differing behaviors or cognitive abilities [22].

Our work adds to a growing number of studies that have demonstrated clear impacts, at multiple biological scales, of rearing animals in captive conditions that deviate from those experienced in natural environments. In addition to documenting differences in growth related to rearing environment, we uncovered several candidate genes potentially underlying behavioral variation and differences in neurological development. Further studies are now needed to examine whether environmental enrichment influences the performance of salmon throughout the life-cycle [62], and whether such interventions can be harnessed to improve the efficacy of conservation and restoration efforts involving captive-rearing programs. Given that salmon are philopatric (most individuals return to their natal stream to spawn), a study that combines experimental exposure to enriched and traditional hatchery rearing environments with subsequent genetic pedigree-based investigation of adult recrutiment from each of the treatments could be used to quantify the efficacy of enrichment across salmonid life-cycle.

## Data Archiving

Gene transcription datasets have been uploaded to the GEO archive (#GSE63982).

## Supporting Information

S1 File (DOCX)
