# Supplementary material for: Transcriptomic Responses of Atlantic Salmon (Salmo salar) to Environmental Enrichment during Juvenile Rearing
Source: PLoS One. 2015 Mar 5;10(3):e0118378. doi: 10.1371/journal.pone.0118378 (PMC4350989; doi:10.1371/journal.pone.0118378)
Supplement: S1 File — (DOCX) [file pone.0118378.s001.docx]

Supporting Information

Supplemental Methods

R-Scripts used for microarray image analysis and analysis of gene transcription profiles of salmon juveniles from enriched and unenriched hatchery rearing envrionments.

Microarray data acquisition (scripts adapted from Booman et al. 2011)

1. Images exported from the ScanArray Gx Plus scanner and ScanExpress v4.0 software were visualized using Imagene. In Imagene, manual flagging was used to exclude spots of poor quality.
2. Further processing of the data was done in R:

# Script based on 'script 110327.R'

# Script written and executed over multiple days January 24-25 2013

# Load marray package

# Read in array info to make object of class marrayInfo

targets_fam_trmt_melissa <- read.marrayInfo(fname="targets_fam_trmt.txt",info.id=NULL, labels=NULL, notes="targets_fam_trmt_melissa", sep="\t", skip=0, quote="\"")

# Read in gal file info indicating Name and ID columns and columns containing layout info to make object of class marrayInfo

galinfoAgilent <- read.Galfile("025055_D_20090817_AID.gal", path=".", info.id=c("Name","Annotation ID","ID","ControlType"), layout.id=c(Block="Block", Row="Row", Column="Column"), labels="Name", notes="", sep="\t", skip=NULL, ncolumns=1)

# Create vector of array names

arrayNames <- targets_fam_trmt_melissa@maLabels

# Read in, summarize and display intensity data for all arrays

mrawMedian <- read.marrayRaw(fnames=arrayNames, path=".", name.Gf="Signal Median 2", name.Gb="Background Median 2", name.Rf="Signal Median 1", name.Rb="Background Median 1", name.W="Flag", layout=galinfoAgilent$layout, gnames=galinfoAgilent$gnames, targets=targets_fam_trmt_melissa, notes=NULL, skip=NULL, sep="\t", quote="\"", DEBUG=FALSE)

summary(mrawMedian)

# Check if order of intensities and target are the same

checkTargetInfo(mrawMedian)

# Remove control spots

mrawMedianNoControl <- subset(mrawMedian,!(mrawMedian@maGnames@maInfo$ControlType %in% c("ignore","pos","neg")))

# Calculate summary stats and cutoff levels

# Create separate object for this, and remove manual flagged spots (flag 1)

# Manually flagged spots are removed to prevent blowing up the background SD because of dust etc.

mrawMedianStats <- mrawMedianNoControl

for (i in 1:32){

mrawMedianStats@maGf[,i][mrawMedianStats@maW[,i]==1]<-NA

mrawMedianStats@maGb[,i][mrawMedianStats@maW[,i]==1]<-NA

mrawMedianStats@maRf[,i][mrawMedianStats@maW[,i]==1]<-NA

mrawMedianStats@maRb[,i][mrawMedianStats@maW[,i]==1]<-NA

}

# Calculate average (avg), standard deviation (sd), signal/background ratio (sbr) and cutoffs

avgGf <- colMeans(mrawMedianStats@maGf, na.rm=TRUE)

avgGb <- colMeans(mrawMedianStats@maGb, na.rm=TRUE)

avgRf <- colMeans(mrawMedianStats@maRf, na.rm=TRUE)

avgRb <- colMeans(mrawMedianStats@maRb, na.rm=TRUE)

sdGf <- apply(mrawMedianStats@maGf,2,sd, na.rm=TRUE)

sdGb <- apply(mrawMedianStats@maGb,2,sd, na.rm=TRUE)

sdRf <- apply(mrawMedianStats@maRf,2,sd, na.rm=TRUE)

sdRb <- apply(mrawMedianStats@maRb,2,sd, na.rm=TRUE)

sbrG <- avgGf/avgGb

sbrR <- avgRf/avgRb

cutoffs <- matrix(0,32,2)

for (i in 1:32){

cutoffs[i,1] <- avgGb[i] + (2*sdGb[i])

cutoffs[i,2] <- avgRb[i] + (2*sdRb[i])

}

# To export cutoffs into text file, add column and row names

cutoffsExport <- cbind(arrayNames,cutoffs)

cutoffsExport <- rbind(c('ArrayID','Green cutoff','Red cutoff'),cutoffsExport)

write.table(cutoffsExport,"cutoff levels.txt",quote=FALSE,sep="\t")

# To export summary statistics to text file, add column and row names

statsExport <- cbind(avgGf,sdGf,avgRf,sdRf,avgGb,sdGb,avgRb,sdRb,sbrG,sbrR)

write.table(statsExport,"summary statistics.txt",quote=FALSE,sep="\t")

# Continue with normalizing dataset, using the mrawMedianNoControl object

# (which has only control spots removed)

# Normalize using printtip Loess

mnormMedianNoControl <- maNormMain(mrawMedianNoControl,echo=TRUE)

summary(mnormMedianNoControl)

# Plot boxplots to check normalization

for (i in 1:32){

pdf(paste("RawMedianBoxPlots",targets_fam_trmt_melissa@maInfo[i,2],".pdf", sep=""), paper="letter")

boxplot(mrawMedianNoControl[, i], xvar = "maPrintTip", yvar = "maM")

dev.off()

pdf(paste("NormMedianBoxPlots",targets_fam_trmt_melissa@maInfo[i,2],".pdf", sep=""), paper="letter")

boxplot(mnormMedianNoControl[, i], xvar = "maPrintTip", yvar = "maM")

dev.off()

}

pdf("RawMedianBoxPlotsAllArrays.pdf", paper="letter")

boxplot(mrawMedianNoControl, yvar = "maM")

dev.off()

pdf("NormMedianBoxPlotsAllArrays.pdf", paper="letter")

boxplot(mnormMedianNoControl, yvar = "maM")

dev.off()

# Plot scatterplots; example MA plots with LowessLines

for (i in 1:32){

pdf(paste("RawMedianScatterPlot",targets_fam_trmt_melissa@maInfo[i,2],".pdf", sep=""), paper="letter")

defs <- maDefaultPar(mrawMedianNoControl[, i], x = "maA", y = "maM", z = "maPrintTip")

legend.func <- do.call("maLegendLines", defs$def.legend)

lines.func <- do.call("maLowessLines", c(list(TRUE, f = 0.3), defs$def.lines))

plot(mrawMedianNoControl[, i], xvar = "maA", yvar = "maM", zvar = "maPrintTip", lines.func, text.func = maText(), legend.func)

dev.off()

pdf(paste("NormMedianScatterPlot",targets_fam_trmt_melissa@maInfo[i,2],".pdf", sep=""), paper="letter")

defs <- maDefaultPar(mnormMedianNoControl[, i], x = "maA", y = "maM", z = "maPrintTip")

legend.func <- do.call("maLegendLines", defs$def.legend)

lines.func <- do.call("maLowessLines", c(list(TRUE, f = 0.3), defs$def.lines))

plot(mnormMedianNoControl[, i], xvar = "maA", yvar = "maM", zvar = "maPrintTip", lines.func, text.func = maText(), legend.func)

dev.off()

}

# Create array data where values below specific cutoff are replaced by NA after normalization

# Create different datasets

# And one with only cutoff for Green used (i.e. to prevent loss of genes that are switched off in a subgroup of samples)

mnormCutoffBoth <- mnormMedianNoControl

for (i in 1:91){

removeG <- mrawMedianNoControl@maGf[,i]<cutoffs[i,1]

removeR <- mrawMedianNoControl@maRf[,i]<cutoffs[i,2]

mnormCutoffBoth@maM[,i][removeG]<-NA

mnormCutoffBoth@maM[,i][removeR]<-NA

mnormCutoffBoth@maA[,i][is.na(mnormCutoffBoth@maM[,i])]<-NA

}

mnormCutoffGreen <- mnormMedianNoControl

for (i in 1:32){

removeG <- mrawMedianNoControl@maGf[,i]<cutoffs[i,1]

mnormCutoffGreen@maM[,i][removeG]<-NA

mnormCutoffGreen@maA[,i][is.na(mnormCutoffGreen@maM[,i])]<-NA

}

# Make a count of the Imagene quality flags remaining after removal of controls

# (since spots below cutoff are not removed but rather replaced by NA, a count after cutoff levels is useless)

flags <- mrawMedianNoControl@maW

flags1 <- flags == 1

flags2 <- flags == 2

flags3 <- flags == 3

flagcounts <- matrix(0,32,3)

flagcounts[,1] <- apply(flags1,2,sum)

flagcounts[,2] <- apply(flags2,2,sum)

flagcounts[,3] <- apply(flags3,2,sum)

write.table(flagcounts,"flagcounts after control removal.txt",quote=FALSE,sep="\t")

# Remove all flagged spots

mnormCutoffBothrmFlagAll <- mnormCutoffBoth

for (i in 1:1){

mnormCutoffBothrmFlagAll@maM[,i][mnormCutoffBothrmFlagAll@maW[,i]==1]<-NA

[mnormCutoffBothrmFlagAll@maW[,i]==2]<-NA

mnormCutoffBothrmFlagAll@maA[,i][is.na(mnormCutoffBothrmFlagAll@maM[,i])]<-NA

}

mnormCutoffBothrmFlagAll.table <- cbind(mnormCutoffBothrmFlagAll@maGnames@maInfo$ID,mnormCutoffBothrmFlagAll@maM)

write.table(mnormCutoffBothrmFlagAll.table,"mnormCutoffBothrmFlagAll_ratios.txt",quote=FALSE,sep="\t")

mnormCutoffGreenrmFlagAll <- mnormCutoffGreen

for (i in 1:32){

mnormCutoffGreenrmFlagAll@maM[,i][mnormCutoffGreenrmFlagAll@maW[,i]==2]<-NA

mnormCutoffGreenrmFlagAll@maA[,i][is.na(mnormCutoffGreenrmFlagAll@maM[,i])]<-NA

}

for (i in 1:32){

mnormCutoffGreenrmFlagAll@maM[,i][mnormCutoffGreenrmFlagAll@maW[,i]==1]<-NA

mnormCutoffGreenrmFlagAll@maA[,i][is.na(mnormCutoffGreenrmFlagAll@maM[,i])]<-NA

}

mnormCutoffGreenrmFlagAll.table <- cbind(mnormCutoffGreenrmFlagAll@maGnames@maInfo$ID,mnormCutoffGreenrmFlagAll@maM)

write.table(mnormCutoffGreenrmFlagAll.table,"mnormCutoffGreenrmFlagAll_ratios.txt",quote=FALSE,sep="\t")

# Make new tables removing spots with >=25% NA (32 arrays, 8 NA, so 8 and smaller allowed)

mnormCutoffGreenrmFlagAllNACount <- rowSums(is.na(mnormCutoffGreenrmFlagAll.table))

mnormCutoffGreen.NA <- cbind(mnormCutoffGreenrmFlagAll.table,mnormCutoffGreenrmFlagAllNACount)

write.table(mnormCutoffGreen.NA,file="mnormCutoffGreenNACount.txt",quote=FALSE,sep="\t")

mnormCutoffGreen.full <- read.table("mnormCutoffGreenNACount.txt",header=TRUE,sep="\t",row.names=1,fill=TRUE)

mnormCutoffGreen.NA25 <- subset(mnormCutoffGreen.full,mnormCutoffGreen.full[,34]<9)

mnormCutoffGreen.NA25 <- mnormCutoffGreen.NA25[,1:33]

write.table(mnormCutoffGreen.NA25,"Green_avg_NACutoff_25percent.txt",quote=FALSE,sep="\t")

# Impute missing data using EMarray from LSimpute applet

# Adapt LSimpute commands to new files and folders

# Do not forget to change text files first to insert a tab at the beginning and remove './' and '.txt'

Siggenes analysis (scripts adapted from Booman et al. 2011)

1. As part of the previous script, all probes for which the log_2_ ratio was missing (NA) in more than 25% of arrays were removed from the normalized and thresholded log transcription data. This resulted in a final dataset comprised of 21,117 probes.
2. Missing data in the new dataset were imputed using the EM_array algorithm from the LSimpute package as described by Bø et al., *Nucleic Acids Research* 2004, 32:e34.
3. Imputed data were read into R and two-class comparison analysis was performed with the package ‘siggenes’:

#Read in imputed data

table.melissa.avg.25.imputed.full <- read.table("Green_avg_NACutoff_25percent_imputed_EMarray_repl_rem.txt",header=TRUE,sep="\t",row.names=1,fill=TRUE)

table.25imputed_all_families_NG_vs_G <- cbind(table.melissa.avg.25.imputed.full[,1:5],table.melissa.avg.25.imputed.full[,11:15],table.melissa.avg.25.imputed.full[,21:25],table.melissa.avg.25.imputed.full[,6:10],table.melissa.avg.25.imputed.full[,16:20],table.melissa.avg.25.imputed.full[,26:30])

write.table(table.25imputed_all_families_NG_vs_G, "all_families_NG_vs_G_25_percent_imputed.txt",quote=FALSE, sep="\t")

#assign classes to each treatment zero is control (No gravel) and 1 is treatment (Gravel) in this case and which is analogous to U and I respectively in Fig.1 from Tusher et al. 2001

#Run SAM class comparison in siggenes

all_families_data<- table.25imputed_all_families_NG_vs_G

class.melissa <- rep(c(0,1),c(15,15))

all_families.out <- sam(all_families_data,class.melissa,method=d.stat,var.equal=FALSE,B=2000,B.more=0.1,B.max=30000,med=TRUE,gene.names=dimnames(all_families_data)[[1]],use.dm=FALSE,R.unlog=TRUE,na.replace=FALSE,rand=12345)

#View SAM output: all_families.out

summary(all_families.out)

summary(all_families.out,delta=seq(0.5,1.2,0.1))

plot(all_families.out)

plot(all_families.out,0.9)

sum.all_families <- summary(all_families.out,0.9)

siggenes.melissa <- sum.all_families@mat.sig

write.table(siggenes.melissa,"all_families_averaged_FDR_0p012_255.txt",quote=FALSE,sep="\t")

Figure A. Midsaggital histological cross-section of a juvenile Atlantic salmon head at emergence. The dashed line in the picture indicates where the head was severed from the body in our study of gene transcription patterns in juvenile salmon. The location of the heart, regions of the brain, and muscle tissue are also indicated. Picture provided by J. Winkowski and I. Fleming.


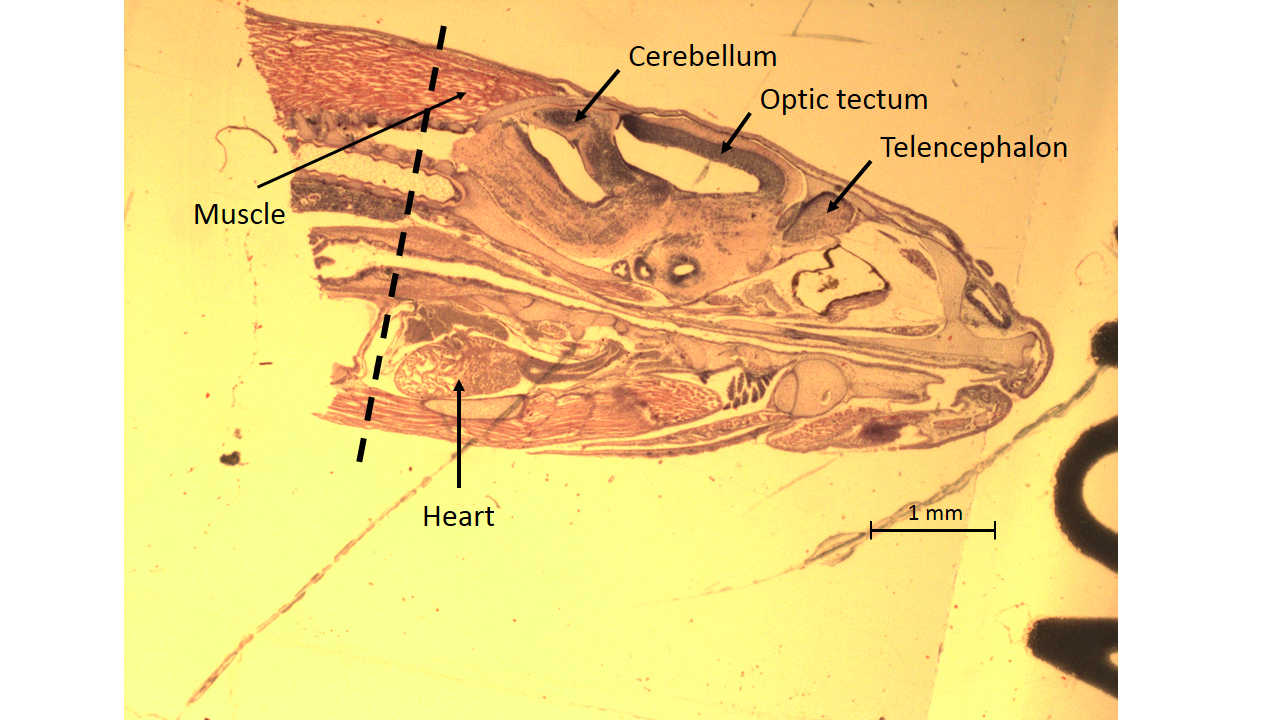


Table A. Atlantic salmon microarray probes identified as differentially transcribed between juveniles reared in enriched and unenriched (traditional) hatchery environments. The results shown follow the analysis of gene transcription patterns across the three families (X11, X22, X35) in a total of 15 complex and 15 simple individuals using the cGRASP-designed Agilent 4×44K salmon gene microarray. Differentially transcribed genes were identified using a FDR of 5% in siggenes. The fold-change in gene transcription levels found in enriched/unenriched environments are indicated.

| Probe ID | Best BLASTx hit^*^ | Fold-change  (all families) | Fold-change  (X11) | Fold-change  (X22) | Fold-change  (X35) |
| --- | --- | --- | --- | --- | --- |
| C067R138 | protein-glutamine gamma-glutamyltransferase e-like | 8.55 | 4.21 | 20.72 | 2.21 |
| C204R103 | cytochrome p450 2m1 | 4.23 | 5.37 | 4.99 | 3.06 |
| C237R040 | PREDICTED: hypothetical protein LOC324610 | 2.87 | 3.40 | 2.66 | 2.58 |
| C205R145 | claudin-10-like | 2.41 | 3.92 | 1.38 | 2.48 |
| C211R110 | complement receptor type 1-like | 2.26 | 1.99 | 2.87 | 2.12 |
| C258R138 | UNKNOWN | 2.13 | 6.88 | 1.30 | 1.39 |
| C051R138 | hypothetical protein MTR_5g050970 | 2.05 | 2.22 | 1.92 | 2.09 |
| C211R161 | metalloproteinase inhibitor 2 precursor | 2.05 | 2.76 | 2.51 | 1.32 |
| C026R035 | protein phosphatase 1 regulatory subunit 14b | 1.90 | 2.82 | 1.43 | 1.52 |
| C001R030 | metalloproteinase inhibitor 2 precursor | 1.89 | 2.12 | 2.02 | 1.60 |
| C155R133 | gtpase imap family member 7 | 1.87 | 2.20 | 1.40 | 2.49 |
| C166R069 | uroplakin-1a | 1.81 | 2.10 | 1.49 | 2.26 |
| C172R070 | cytochrome p450 2m1 | 1.80 | 1.79 | 1.62 | 2.02 |
| C135R135 | ornithine decarboxylase antizyme | 1.78 | 1.75 | 2.11 | 1.41 |
| C215R100 | transmembrane protein 106b | 1.77 | 2.60 | 1.21 | 1.73 |
| C036R160 | atpase h+ transporting v1 subunit g isoform 1 | 1.76 | 2.61 | 1.78 | 1.05 |
| C258R048 | zinc finger protein zic 4 | 1.75 | 2.29 | 1.13 | 2.26 |
| C085R160 | phosphorylase b kinase gamma catalytic testis liver isoform | 1.73 | 2.05 | 1.77 | 1.33 |
| C022R025 | udp-n-acetylhexosamine pyrophosphorylase-like | 1.73 | 2.53 | 1.44 | 1.35 |
| C124R148 | myelin and lymphocyte protein | 1.72 | 1.74 | 2.23 | 1.33 |
| C104R034 | migration and invasion enhancer 1 | 1.71 | 2.13 | 1.79 | 1.34 |
| C024R096 | hypoxanthine-guanine phosphoribosyltransferase | 1.69 | 2.32 | 1.36 | 1.54 |
| C025R093 | protein eva-1 homolog c-like | 1.67 | 1.78 | 1.65 | 1.58 |
| C051R009 | protein fam181a-like | 1.66 | 2.69 | 1.08 | 1.64 |
| C066R122 | vacuolar proton pump subunit e 1 | 1.65 | 1.86 | 1.78 | 1.27 |
| C177R030 | ras-related c3 botulinum toxin substrate 1 | 1.65 | 1.78 | 1.59 | 1.55 |
| C045R110 | purpurin precursor | 1.62 | 1.74 | 1.77 | 1.44 |
| C147R126 | prodynorphin precursor | 1.62 | 2.37 | 1.53 | 1.18 |
| C076R102 | cyclin-g2-like | 1.60 | 1.82 | 1.43 | 1.56 |
| C032R048 | bcl2 adenovirus e1b 19 kda protein-interacting protein 3 | 1.59 | 2.01 | 1.48 | 1.42 |
| C255R108 | peptidyl-prolyl cis-trans isomerase c | 1.56 | 1.51 | 1.37 | 1.94 |
| C257R146 | embigin precursor | 1.55 | 2.57 | 0.99 | 1.79 |
| C182R144 | pyridoxal kinase-like | 1.55 | 2.61 | 1.04 | 1.50 |
| C088R165 | UNKNOWN | 1.55 | 2.34 | 1.10 | 1.39 |
| C128R109 | dynein light chain cytoplasmic | 1.54 | 1.91 | 1.41 | 1.29 |
| C086R164 | teashirt homolog 1 | 1.53 | 1.74 | 1.05 | 2.32 |
| C078R052 | integral membrane protein 2c | 1.53 | 1.70 | 1.34 | 1.58 |
| C083R166 | sodium-coupled neutral amino acid transporter 7-like | 1.53 | 1.80 | 1.55 | 1.23 |
| C138R056 | envoplakin-like | 1.52 | 1.84 | 1.28 | 1.49 |
| C025R107 | zinc finger protein 512b | 1.52 | 1.01 | 1.48 | 2.08 |
| C157R083 | UNKNOWN | 1.51 | 1.28 | 1.67 | 1.63 |
| C138R082 | cerebellin-1-like | 1.50 | 2.43 | 1.39 | 1.07 |
| C081R073 | phosphatidylinositol transfer protein alpha isoform | 1.50 | 1.87 | 1.33 | 1.36 |
| C147R039 | neuritin | 1.50 | 1.83 | 1.20 | 1.57 |
| C195R042 | gdp-mannose dehydratase | 1.49 | 1.87 | 1.26 | 1.43 |
| C196R170 | myelin and lymphocyte protein | 1.48 | 1.29 | 2.04 | 1.27 |
| C023R126 | 5 -amp-activated protein kinase subunit beta-1 | 1.48 | 1.69 | 1.39 | 1.36 |
| C069R110 | UNKNOWN | 1.47 | 1.74 | 1.18 | 1.58 |
| C196R097 | bccip homolog | 1.47 | 1.61 | 1.50 | 1.32 |
| C141R027 | transcription factor sox-2 | 1.46 | 1.70 | 1.36 | 1.38 |
| C086R033 | gamma-crystallin m2-like | 1.46 | 1.31 | 1.72 | 1.37 |
| C122R157 | aspartyl asparaginyl beta-hydroxylase-like | 1.46 | 1.28 | 1.70 | 1.41 |
| C262R103 | transcription factor jun-d | 1.45 | 2.52 | 1.15 | 1.13 |
| C082R123 | polycomb complex protein bmi-1-a | 1.45 | 1.79 | 1.18 | 1.39 |
| C091R079 | probable g-protein coupled receptor 88 | 1.45 | 1.64 | 1.55 | 1.18 |
| C075R036 | envoplakin-like | 1.44 | 1.27 | 1.94 | 1.28 |
| C113R065 | chromosome 11 open reading frame 87 | 1.44 | 1.57 | 1.44 | 1.29 |
| C243R121 | peroxisome proliferator-activated receptor beta | 1.44 | 1.75 | 1.47 | 1.18 |
| C155R148 | synaptic vesicle 2-related protein | 1.43 | 1.51 | 1.62 | 1.19 |
| C064R164 | schwannomin-interacting protein 1-like | 1.43 | 1.78 | 1.29 | 1.29 |
| C028R051 | claudin-4 | 1.42 | 1.74 | 1.31 | 1.26 |
| C120R154 | ras-related protein rab-25 | 1.42 | 1.38 | 1.49 | 1.37 |
| C023R052 | hydroxyacylglutathione hydrolase | 1.41 | 1.66 | 1.15 | 1.51 |
| C048R158 | scel protein | 1.40 | 1.57 | 1.22 | 1.43 |
| C024R036 | proteasome assembly chaperone 1 | 1.39 | 1.46 | 1.31 | 1.41 |
| C032R036 | btb poz domain-containing protein kctd5 | 1.39 | 1.64 | 1.24 | 1.27 |
| C207R079 | hcls1-associated protein x-1 | 1.38 | 1.47 | 1.33 | 1.35 |
| C080R142 | guanine nucleotide-binding protein subunit alpha-14-like | 1.38 | 1.28 | 1.65 | 1.23 |
| C023R047 | calpain-2 catalytic subunit-like | 1.38 | 1.55 | 1.27 | 1.32 |
| C128R096 | UNKNOWN | 1.37 | 1.42 | 1.26 | 1.46 |
| C042R122 | UNKNOWN | 1.37 | 1.28 | 1.50 | 1.32 |
| C238R108 | hydroxyacylglutathione hydrolase | 1.37 | 1.55 | 1.19 | 1.41 |
| C181R097 | transmembrane protein 199 | 1.37 | 1.53 | 1.29 | 1.31 |
| C032R066 | UNKNOWN | 1.36 | 1.23 | 1.63 | 1.25 |
| C207R063 | UNKNOWN | 1.36 | 1.53 | 1.35 | 1.21 |
| C136R151 | UNKNOWN | 1.36 | 1.16 | 1.41 | 1.55 |
| C089R127 | adipocyte plasma membrane-associated protein | 1.35 | 1.46 | 1.23 | 1.38 |
| C154R165 | cyclin-dependent kinase 4 inhibitor b | 1.35 | 1.56 | 1.29 | 1.23 |
| C074R167 | protein manbal | 1.35 | 1.61 | 1.35 | 1.14 |
| C002R100 | pol-like protein | 1.35 | 1.46 | 1.34 | 1.25 |
| C084R110 | hypothetical protein PCYB_123530 | 1.35 | 1.25 | 1.35 | 1.44 |
| C131R046 | n-acylneuraminate cytidylyltransferase | 1.34 | 1.43 | 1.23 | 1.40 |
| C135R166 | insulinoma-associated protein 1-like | 1.33 | 1.36 | 1.49 | 1.15 |
| C043R087 | low quality protein: ornithine decarboxylase antizyme 2 | 1.33 | 1.25 | 1.28 | 1.48 |
| C185R128 | calpain (m ii) large subunit | 1.33 | 1.49 | 1.37 | 1.14 |
| C031R158 | plasminogen activator inhibitor 1 rna-binding protein isoform 4 | 1.32 | 1.50 | 1.36 | 1.14 |
| C078R140 | p53 apoptosis effector related to pmp-22 | 1.32 | 1.16 | 1.53 | 1.26 |
| C048R097 | transmembrane protein 50a | 1.32 | 1.68 | 1.15 | 1.19 |
| C118R132 | cyclin i | 1.32 | 1.44 | 1.27 | 1.23 |
| C188R163 | UNKNOWN | 1.32 | 1.37 | 1.34 | 1.25 |
| C203R062 | scel protein | 1.32 | 1.67 | 1.07 | 1.30 |
| C080R079 | cyclin-dependent kinase 4 inhibitor b | 1.32 | 1.55 | 1.15 | 1.29 |
| C068R076 | dopachrome tautomerase | 1.32 | 1.23 | 1.55 | 1.21 |
| C115R053 | udp-n-acetylhexosamine pyrophosphorylase-like | 1.31 | 1.24 | 1.58 | 1.14 |
| C030R156 | phosphorylase b kinase gamma catalytic skeletal muscle isoform | 1.31 | 1.20 | 1.34 | 1.39 |
| C224R065 | envoplakin-like | 1.30 | 1.47 | 1.32 | 1.17 |
| C138R068 | small g protein signaling modulator 1-like | 1.30 | 1.45 | 1.16 | 1.32 |
| C031R128 | transmembrane and coiled-coil domain-containing protein 1 | 1.30 | 1.40 | 1.23 | 1.28 |
| C184R075 | protein fam3c-like | 1.30 | 1.31 | 1.31 | 1.27 |
| C195R152 | scel protein | 1.30 | 1.51 | 1.08 | 1.34 |
| C050R102 | upf0414 transmembrane protein c20orf30 homolog | 1.29 | 1.47 | 1.32 | 1.11 |
| C030R160 | translocator protein | 1.28 | 1.40 | 1.28 | 1.18 |
| C215R130 | ras-related protein rab-25 | 1.28 | 1.53 | 1.15 | 1.20 |
| C062R103 | UNKNOWN | 1.27 | 1.22 | 1.27 | 1.33 |
| C219R070 | transmembrane protein 60 | 1.27 | 1.39 | 1.20 | 1.20 |
| C103R116 | transmembrane emp24 domain-containing protein 4 | 1.27 | 1.41 | 1.23 | 1.19 |
| C135R126 | UNKNOWN | 1.26 | 1.20 | 1.22 | 1.38 |
| C045R094 | mitochondrial fission process protein 1 | 1.26 | 1.31 | 1.13 | 1.34 |
| C105R094 | dehydrogenase reductase sdr family member 1 | 1.24 | 1.36 | 1.23 | 1.14 |
| C168R138 | sodium- and chloride-dependent taurine transporter | 1.23 | 1.19 | 1.24 | 1.27 |
| C229R087 | mrna turnover protein 4 homolog | 0.87 | 0.84 | 0.87 | 0.89 |
| C150R077 | rwd domain-containing protein 1 | 0.86 | 0.80 | 0.85 | 0.94 |
| C154R123 | wd repeat domain 46 | 0.86 | 0.92 | 0.84 | 0.82 |
| C253R055 | 26s protease regulatory subunit 8 | 0.85 | 0.76 | 0.89 | 0.91 |
| C115R023 | wd repeat-containing protein 75 | 0.85 | 0.87 | 0.81 | 0.86 |
| C139R132 | dna (cytosine-5)-methyltransferase 1-like | 0.85 | 0.86 | 0.86 | 0.82 |
| C146R131 | small nuclear ribonucleoprotein sm d3 | 0.84 | 0.79 | 0.89 | 0.86 |
| C161R096 | nuclear pore complex protein nup160 | 0.84 | 0.74 | 0.93 | 0.87 |
| C075R094 | phosphoribosylaminoimidazole phosphoribosylaminoimidazole succinocarboxamide synthetase | 0.84 | 0.80 | 0.89 | 0.84 |
| C144R110 | UNKNOWN | 0.84 | 0.70 | 0.98 | 0.85 |
| C232R114 | translation initiation factor eif-2b subunit delta-like | 0.84 | 0.72 | 0.85 | 0.95 |
| C231R113 | ribosomal rna small subunit methyltransferase nep1-like | 0.83 | 0.71 | 0.87 | 0.95 |
| C107R039 | 39s ribosomal protein mitochondrial precursor | 0.83 | 0.74 | 0.87 | 0.90 |
| C144R089 | upf0609 protein c4orf27 homolog | 0.83 | 0.71 | 0.93 | 0.86 |
| C151R102 | UKNOWN | 0.83 | 0.77 | 0.89 | 0.83 |
| C059R097 | UNKNOWN | 0.83 | 0.75 | 1.01 | 0.73 |
| C012R051 | steroid receptor rna activator 1 | 0.83 | 0.69 | 0.94 | 0.90 |
| C182R064 | transposable element tcb1 transposase | 0.83 | 0.70 | 0.83 | 0.96 |
| C129R072 | adenylosuccinate lyase | 0.83 | 0.75 | 0.88 | 0.86 |
| C101R057 | delta-like protein b-like | 0.83 | 0.87 | 0.81 | 0.80 |
| C223R091 | lamin-b receptor | 0.83 | 0.63 | 1.01 | 0.87 |
| C132R061 | dna (cytosine-5)-methyltransferase 1 | 0.83 | 0.76 | 0.88 | 0.85 |
| C159R165 | atp synthase-coupling factor mitochondrial precursor | 0.83 | 0.92 | 0.82 | 0.76 |
| C101R107 | h aca ribonucleoprotein complex subunit 1 | 0.83 | 0.72 | 0.94 | 0.83 |
| C149R114 | general transcription factor 3c polypeptide 6 | 0.82 | 0.77 | 0.93 | 0.79 |
| C229R153 | endoplasmin precursor | 0.82 | 0.76 | 0.84 | 0.87 |
| C153R084 | atp-dependent rna helicase dhx8 | 0.82 | 0.64 | 1.02 | 0.86 |
| C205R011 | methylcrotonoyl- carboxylase beta mitochondrial | 0.82 | 0.82 | 0.72 | 0.94 |
| C042R030 | tyrosine-protein kinase 223-like | 0.82 | 0.63 | 0.89 | 0.96 |
| C026R007 | protein disulfide-isomerase a5-like | 0.82 | 0.69 | 0.88 | 0.93 |
| C147R098 | tyrosine--trna cytoplasmic | 0.82 | 0.77 | 0.85 | 0.84 |
| C155R025 | cell adhesion molecule 4-like | 0.82 | 0.71 | 0.87 | 0.90 |
| C237R086 | guanine nucleotide-binding 3-like | 0.82 | 0.82 | 0.85 | 0.80 |
| C148R030 | nostrin isoform 1 | 0.82 | 0.84 | 0.84 | 0.78 |
| C056R087 | deoxycytidine kinase | 0.82 | 1.00 | 0.71 | 0.77 |
| C148R076 | exportin-1 | 0.82 | 0.79 | 0.87 | 0.80 |
| C057R087 | transposable element tcb1 transposase | 0.82 | 0.65 | 0.95 | 0.88 |
| C131R041 | cell differentiation protein rcd1 homolog | 0.82 | 0.75 | 0.83 | 0.90 |
| C170R082 | zinc finger protein 62 homolog | 0.81 | 0.75 | 0.80 | 0.89 |
| C163R052 | UNKNOWN | 0.81 | 0.68 | 0.76 | 1.01 |
| C182R067 | transcriptional repressor ctcf-like | 0.81 | 0.74 | 0.90 | 0.81 |
| C221R081 | myosin light chain 3 | 0.81 | 0.87 | 0.75 | 0.83 |
| C090R023 | eukaryotic initiation factor 4a-iii | 0.81 | 0.77 | 0.91 | 0.77 |
| C007R133 | survival motor neuron protein 1 | 0.81 | 0.73 | 0.90 | 0.81 |
| C122R116 | UNKNOWN | 0.81 | 0.79 | 0.75 | 0.88 |
| C011R073 | UNKNOWN | 0.81 | 0.66 | 0.96 | 0.84 |
| C030R063 | UNKNOWN | 0.81 | 0.83 | 0.90 | 0.71 |
| C259R138 | nedd4-like e3 ubiquitin-protein ligase wwp2 | 0.81 | 0.78 | 0.65 | 1.02 |
| C086R088 | acidic leucine-rich nuclear phosphoprotein 32 family member a | 0.81 | 0.63 | 0.88 | 0.90 |
| C131R042 | monocarboxylate transporter 2-like | 0.81 | 0.68 | 0.96 | 0.82 |
| C123R043 | prickle-like protein 2 | 0.81 | 0.79 | 0.87 | 0.77 |
| C258R130 | protein smg8-like | 0.81 | 0.69 | 0.86 | 0.90 |
| C157R093 | peptidyl-trna hydrolase mitochondrial precursor | 0.81 | 0.74 | 0.97 | 0.75 |
| C194R147 | gmp reductase 1 | 0.81 | 0.81 | 0.72 | 0.90 |
| C259R081 | proliferating cell nuclear antigen | 0.81 | 0.79 | 0.81 | 0.82 |
| C189R165 | UNKNOWN | 0.81 | 0.59 | 0.94 | 0.92 |
| C200R099 | pre-rrna-processing protein tsr1 homolog | 0.81 | 0.73 | 0.85 | 0.85 |
| C111R137 | gmp reductase 1 | 0.80 | 0.68 | 0.84 | 0.89 |
| C210R031 | tubulin alpha-1c chain- partial | 0.80 | 0.76 | 0.76 | 0.90 |
| C199R164 | rrp12-like protein | 0.80 | 0.92 | 0.82 | 0.69 |
| C146R082 | rna-binding protein 28 isoform 2 | 0.80 | 0.78 | 0.81 | 0.82 |
| C034R093 | transmembrane and tpr repeat-containing protein 4 | 0.80 | 0.68 | 1.02 | 0.71 |
| C089R012 | u5 small nuclear ribonucleoprotein 40 kda protein | 0.80 | 0.92 | 0.67 | 0.80 |
| C165R091 | protein emsy | 0.80 | 0.62 | 0.85 | 0.94 |
| C122R068 | ttnl protein | 0.80 | 0.76 | 0.75 | 0.90 |
| C148R099 | sjchgc04011 protein | 0.80 | 0.74 | 0.75 | 0.91 |
| C109R127 | placenta-specific protein 9-like | 0.80 | 0.68 | 0.89 | 0.86 |
| C230R124 | nucleolar protein 14 | 0.80 | 0.70 | 0.93 | 0.80 |
| C080R163 | importin subunit alpha-2 | 0.80 | 0.70 | 1.02 | 0.69 |
| C200R070 | eh domain-binding protein 1-like protein 1 | 0.80 | 0.85 | 0.62 | 0.92 |
| C204R141 | plastin-2 isoform 1 | 0.80 | 0.81 | 0.76 | 0.82 |
| C244R043 | kinesin-like protein kif23 | 0.80 | 0.79 | 0.80 | 0.81 |
| C157R127 | cytochrome c oxidase subunit mitochondrial precursor | 0.80 | 0.73 | 0.82 | 0.84 |
| C029R115 | translational activator gcn1 | 0.80 | 0.70 | 0.91 | 0.80 |
| C162R085 | coiled-coil domain-containing protein 43 | 0.80 | 0.78 | 0.85 | 0.77 |
| C165R010 | poly | 0.80 | 0.73 | 0.72 | 0.96 |
| C227R073 | lipoprotein lipase | 0.80 | 0.65 | 0.92 | 0.85 |
| C199R161 | neuronal growth regulator 1-like | 0.80 | 0.79 | 0.83 | 0.78 |
| C031R022 | sorbitol dehydrogenase | 0.80 | 0.79 | 0.86 | 0.75 |
| C186R059 | reticulon 2 | 0.80 | 0.90 | 0.69 | 0.82 |
| C208R015 | cytochrome b-c1 complex subunit mitochondrial precursor | 0.80 | 0.88 | 0.64 | 0.87 |
| C251R045 | cyclin-dependent kinases regulatory subunit 2 | 0.80 | 0.81 | 0.78 | 0.81 |
| C097R015 | dna replication licensing factor mcm3 | 0.80 | 1.01 | 0.69 | 0.73 |
| C008R125 | lysine-specific histone demethylase 1a-like | 0.80 | 0.65 | 0.81 | 0.95 |
| C201R019 | protein mon2 homolog | 0.80 | 0.78 | 0.73 | 0.87 |
| C208R145 | glutamate-rich wd repeat containing 1 | 0.80 | 0.60 | 0.99 | 0.83 |
| C186R110 | homeodomain protein 2b | 0.80 | 0.77 | 0.84 | 0.78 |
| C154R024 | UNKNOWN | 0.80 | 0.73 | 0.71 | 0.92 |
| C236R160 | cytochrome c oxidase polypeptide vic-2 | 0.79 | 0.70 | 0.81 | 0.87 |
| C177R153 | structural maintenance of chromosomes protein 2 | 0.79 | 0.86 | 0.79 | 0.74 |
| C094R091 | n-alpha-acetyltransferase auxiliary subunit | 0.79 | 0.67 | 0.81 | 0.92 |
| C234R130 | dna mismatch repair protein msh6 isoform 2 | 0.79 | 0.88 | 0.81 | 0.71 |
| C162R042 | cleavage and polyadenylation specificity factor subunit 6 | 0.79 | 0.76 | 0.81 | 0.81 |
| C161R086 | small subunit processome component 20 homolog | 0.79 | 0.61 | 0.72 | 1.03 |
| C186R097 | ctp synthase 1-like | 0.79 | 0.74 | 0.78 | 0.84 |
| C078R131 | proteasome subunit beta type-7 precursor | 0.79 | 0.68 | 1.02 | 0.74 |
| C194R076 | targeting protein for xklp2 | 0.79 | 0.70 | 0.91 | 0.78 |
| C202R064 | nucleolar protein 6-like | 0.79 | 0.88 | 0.77 | 0.73 |
| C210R163 | e3 sumo-protein ligase 2 | 0.79 | 0.78 | 0.90 | 0.71 |
| C236R099 | nuclear autoantigenic sperm protein | 0.79 | 0.88 | 0.81 | 0.69 |
| C216R118 | interferon-induced guanylate-binding protein 1 | 0.79 | 0.80 | 0.74 | 0.83 |
| C045R038 | beta-parvin isoform x1 | 0.79 | 0.78 | 0.75 | 0.84 |
| C265R142 | sjchgc04011 protein | 0.79 | 0.59 | 0.76 | 1.02 |
| C112R036 | dna repair protein xrcc1-like | 0.79 | 0.78 | 0.85 | 0.73 |
| C106R091 | UNKNOWN | 0.79 | 0.80 | 0.85 | 0.73 |
| C089R007 | 39s ribosomal protein mitochondrial-like | 0.79 | 0.75 | 0.78 | 0.85 |
| C162R077 | PREDICTED: uncharacterized protein LOC101166494 | 0.79 | 0.75 | 0.84 | 0.78 |
| C038R017 | novel protein e-cadherin | 0.79 | 0.75 | 0.91 | 0.73 |
| C202R116 | UNKNOWN | 0.79 | 0.65 | 0.88 | 0.86 |
| C167R146 | cd83 | 0.79 | 0.61 | 0.95 | 0.84 |
| C064R063 | cytoplasmic dynein 2 light intermediate chain 1-like | 0.79 | 0.76 | 0.71 | 0.90 |
| C266R147 | importin 5 | 0.79 | 0.67 | 0.70 | 1.02 |
| C005R123 | sjchgc09650 protein | 0.79 | 0.59 | 0.90 | 0.91 |
| C159R170 | UNKNOWN | 0.79 | 0.63 | 0.87 | 0.86 |
| C165R160 | 60s ribosomal protein l22-like 1-like | 0.79 | 0.81 | 0.69 | 0.85 |
| C164R145 | enhancer of zeste homolog 2 (enx-1) isoform cra_a | 0.79 | 0.60 | 1.01 | 0.80 |
| C007R074 | zw10 interactor-like | 0.79 | 0.87 | 0.85 | 0.62 |
| C090R110 | ribose-5-phosphate isomerase | 0.79 | 0.90 | 0.75 | 0.70 |
| C098R134 | bis(5 -nucleosyl)-tetraphosphatase | 0.79 | 0.70 | 0.86 | 0.80 |
| C099R059 | 40s ribosomal protein s13 | 0.78 | 0.80 | 0.80 | 0.76 |
| C226R078 | epidermal growth factor-like protein 7-like | 0.78 | 0.74 | 0.81 | 0.80 |
| C243R073 | trimeric intracellular cation channel type a | 0.78 | 0.80 | 0.75 | 0.81 |
| C003R029 | multidrug resistance-associated protein 1-like | 0.78 | 0.62 | 0.88 | 0.89 |
| C114R077 | 14 kda phosphohistidine phosphatase | 0.78 | 0.77 | 0.78 | 0.80 |
| C194R145 | 60s ribosomal protein l3-like | 0.78 | 0.91 | 0.66 | 0.80 |
| C243R102 | g-protein coupled receptor 182 | 0.78 | 0.60 | 1.05 | 0.73 |
| C011R098 | tropomodulin 4 | 0.78 | 0.74 | 0.77 | 0.84 |
| C183R051 | cysteine-rich protein 1 | 0.78 | 0.93 | 0.67 | 0.77 |
| C169R012 | UNKNOWN | 0.78 | 0.54 | 0.74 | 1.10 |
| C172R088 | eukaryotic translation initiation factor 4e | 0.78 | 0.70 | 0.95 | 0.73 |
| C083R023 | structural maintenance of chromosomes protein 2 | 0.78 | 0.92 | 0.73 | 0.68 |
| C075R131 | uracil nucleotide cysteinyl leukotriene receptor-like | 0.78 | 0.65 | 0.69 | 1.05 |
| C174R122 | UNKNOWN | 0.78 | 0.86 | 0.71 | 0.78 |
| C258R070 | sjoegren syndrome scleroderma autoantigen 1 | 0.78 | 0.64 | 0.80 | 0.92 |
| C120R007 | glutaminyl-peptide cyclotransferase-like | 0.78 | 0.79 | 0.68 | 0.88 |
| C019R161 | probable c- | 0.78 | 0.85 | 0.88 | 0.62 |
| C142R018 | UNKNOWN | 0.78 | 0.67 | 0.72 | 0.94 |
| C141R102 | UNKNOWN | 0.78 | 0.52 | 0.92 | 0.89 |
| C169R045 | protein fam212a-like | 0.78 | 0.73 | 0.76 | 0.86 |
| C190R020 | ribonucleoside-diphosphate reductase large subunit | 0.78 | 0.97 | 0.62 | 0.80 |
| C042R058 | cytochrome c oxidase subunit vib isoform 1 | 0.78 | 0.67 | 0.99 | 0.76 |
| C259R019 | UNKNOWN | 0.78 | 0.79 | 0.77 | 0.78 |
| C176R096 | dna-directed rna polymerases and iii subunit rpabc5 | 0.78 | 0.64 | 0.88 | 0.86 |
| C184R019 | UKNOWN | 0.78 | 0.61 | 0.92 | 0.86 |
| C039R055 | aspartic acid-rich protein aspolin2 | 0.78 | 0.62 | 0.93 | 0.82 |
| C229R033 | exosome complex exonuclease rrp44 | 0.78 | 0.73 | 0.68 | 0.93 |
| C028R034 | nuclear transport factor 2 | 0.78 | 0.63 | 0.93 | 0.87 |
| C238R056 | zinc finger protein 503 | 0.78 | 0.78 | 0.81 | 0.73 |
| C228R155 | calsequestrin-1-like | 0.78 | 0.76 | 0.70 | 0.87 |
| C013R120 | zinc finger protein 703-like | 0.78 | 0.50 | 0.93 | 0.95 |
| C167R128 | protein-glutamine gamma-glutamyltransferase 2 | 0.78 | 0.76 | 0.76 | 0.82 |
| C266R135 | hydroxyacid oxidase 2 | 0.78 | 0.90 | 0.63 | 0.84 |
| C252R104 | hemoglobin subunit beta-1 | 0.78 | 0.67 | 0.73 | 0.95 |
| C030R134 | UKNOWN | 0.78 | 0.61 | 0.90 | 0.85 |
| C160R007 | UKNOWN | 0.78 | 0.75 | 0.73 | 0.86 |
| C200R114 | transitional endoplasmic reticulum atpase-like | 0.78 | 0.59 | 0.78 | 0.97 |
| C021R165 | kinesin-like protein kif11-like | 0.78 | 0.70 | 0.94 | 0.72 |
| C229R095 | UKNOWN | 0.78 | 0.62 | 0.88 | 0.85 |
| C236R133 | UNKNOWN | 0.78 | 0.61 | 0.76 | 0.93 |
| C200R073 | drebrin-like protein | 0.78 | 0.67 | 0.72 | 0.95 |
| C172R133 | epidermis-type lipoxygenase 3-like | 0.77 | 0.62 | 0.77 | 0.93 |
| C199R053 | disabled homolog 2-interacting | 0.77 | 0.68 | 0.83 | 0.82 |
| C266R068 | malcavernin-like protein | 0.77 | 0.53 | 0.76 | 1.06 |
| C131R120 | calumenin isoform x1 | 0.77 | 0.60 | 0.90 | 0.88 |
| C008R116 | beta-catenin-like protein 1 | 0.77 | 0.77 | 0.80 | 0.76 |
| C255R041 | UNKNOWN | 0.77 | 0.70 | 0.90 | 0.74 |
| C121R162 | nicotinamide riboside kinase 2 | 0.77 | 0.65 | 0.80 | 0.88 |
| C103R142 | nidogen-1 | 0.77 | 0.78 | 0.77 | 0.76 |
| C092R009 | low quality protein: dedicator of cytokinesis protein 10-like | 0.77 | 0.71 | 0.70 | 0.92 |
| C128R054 | nucleolar protein 14 | 0.77 | 0.70 | 0.86 | 0.78 |
| C243R052 | protein unc-79 homolog | 0.77 | 0.74 | 0.69 | 0.90 |
| C252R069 | laminin subunit alpha-2 | 0.77 | 0.71 | 0.75 | 0.85 |
| C158R131 | interferon-induced guanylate-binding protein 1 | 0.77 | 0.75 | 0.73 | 0.84 |
| C155R100 | noggin-3 precursor | 0.77 | 0.74 | 0.80 | 0.79 |
| C016R017 | filamin-c-like isoform 1 | 0.77 | 0.72 | 0.72 | 0.90 |
| C012R050 | small glutamine-rich tetratricopeptide repeat-containing protein alpha | 0.77 | 0.59 | 0.91 | 0.86 |
| C017R158 | thioredoxin-like protein 1 | 0.77 | 0.78 | 0.89 | 0.67 |
| C234R065 | wd repeat-containing protein 34 | 0.77 | 0.72 | 0.78 | 0.81 |
| C118R070 | intracellular hyaluronan-binding protein 4 | 0.77 | 0.82 | 0.74 | 0.75 |
| C071R100 | interleukin-1 receptor accessory protein isoform x2 | 0.77 | 0.85 | 0.69 | 0.77 |
| C206R043 | probable atp-dependent rna helicase ddx27 | 0.77 | 0.79 | 0.83 | 0.70 |
| C013R156 | receptor-type tyrosine-protein phosphatase c-like | 0.77 | 0.80 | 0.68 | 0.82 |
| C147R067 | PREDICTED: ankyrin-1-like | 0.77 | 0.70 | 0.70 | 0.92 |
| C070R024 | ran gtpase-activating protein 1-like | 0.77 | 0.69 | 0.92 | 0.75 |
| C053R114 | doublesex- and mab-3-related transcription factor a1 | 0.77 | 0.76 | 0.92 | 0.65 |
| C240R151 | conserved oligomeric golgi complex subunit partial | 0.77 | 0.69 | 0.77 | 0.85 |
| C065R122 | rho-related gtp-binding protein | 0.77 | 0.65 | 0.82 | 0.83 |
| C175R071 | protein transport protein sec61 subunit gamma | 0.77 | 0.69 | 0.83 | 0.79 |
| C248R116 | phenylalanine--trna ligase alpha subunit | 0.77 | 0.64 | 0.88 | 0.82 |
| C132R050 | enterin neuropeptide | 0.77 | 0.77 | 0.69 | 0.88 |
| C147R022 | 28s ribosomal protein mitochondrial | 0.77 | 0.78 | 0.66 | 0.86 |
| C103R092 | rotatin | 0.77 | 0.51 | 1.02 | 0.85 |
| C209R016 | UNKNOWN | 0.77 | 0.92 | 0.67 | 0.75 |
| C063R090 | centromere protein u-like | 0.77 | 0.64 | 1.05 | 0.60 |
| C154R066 | nuclear pore complex protein nup107 | 0.77 | 0.72 | 0.81 | 0.78 |
| C234R112 | dihydrolipoyllysine-residue acetyltransferase component of pyruvate dehydrogenase mitochondrial | 0.77 | 0.69 | 0.89 | 0.73 |
| C262R052 | interferon-induced guanylate-binding protein 1 | 0.77 | 0.86 | 0.59 | 0.89 |
| C107R060 | u6 snrna-associated sm-like protein lsm5 | 0.77 | 0.68 | 0.83 | 0.80 |
| C014R147 | suppressor of g2 allele of skp1 homolog | 0.77 | 0.75 | 0.62 | 0.98 |
| C155R168 | nuclear pore complex protein nup205 | 0.76 | 0.74 | 0.85 | 0.70 |
| C200R015 | UNKNOWN | 0.76 | 0.79 | 0.58 | 0.95 |
| C253R040 | forkhead box protein d1-like | 0.76 | 0.78 | 0.77 | 0.74 |
| C199R103 | UNKNOWN | 0.76 | 0.71 | 0.78 | 0.80 |
| C200R045 | dna polymerase alpha subunit b | 0.76 | 0.93 | 0.66 | 0.72 |
| C179R059 | UNKNOWN | 0.76 | 0.90 | 0.74 | 0.64 |
| C180R083 | tropomodulin 4 | 0.76 | 0.75 | 0.70 | 0.84 |
| C161R031 | breast cancer type 2 susceptibility | 0.76 | 0.69 | 0.77 | 0.83 |
| C141R091 | kinesin-like protein kif2c | 0.76 | 0.85 | 0.66 | 0.77 |
| C187R119 | tetratricopeptide repeat protein 4-like | 0.76 | 0.61 | 0.83 | 0.87 |
| C118R021 | UNKNOWN | 0.76 | 0.69 | 0.70 | 0.85 |
| C149R123 | nexilin | 0.76 | 0.88 | 0.68 | 0.75 |
| C013R130 | zinc finger mym-type protein 1-like | 0.76 | 0.65 | 0.86 | 0.79 |
| C207R017 | UNKNOWN | 0.76 | 0.90 | 0.71 | 0.70 |
| C056R089 | troponin slow skeletal and cardiac muscles | 0.76 | 0.74 | 0.91 | 0.66 |
| C099R054 | ribosome production factor 2 homolog | 0.76 | 0.68 | 0.89 | 0.71 |
| C014R141 | dna-directed rna polymerase i subunit rpa43 | 0.76 | 0.69 | 0.71 | 0.89 |
| C124R010 | mitochondrial inner membrane protease subunit 1 | 0.76 | 0.68 | 0.73 | 0.88 |
| C187R133 | methionine synthase reductase | 0.76 | 0.55 | 0.91 | 0.82 |
| C229R119 | protection of telomeres protein 1 | 0.76 | 0.71 | 0.88 | 0.71 |
| C248R102 | 26s proteasome non-atpase regulatory subunit 2 | 0.76 | 0.68 | 0.76 | 0.84 |
| C263R100 | xin actin-binding repeat-containing protein 1-like | 0.76 | 0.54 | 0.79 | 0.96 |
| C224R160 | intraflagellar transport protein 88 homolog | 0.76 | 0.67 | 0.87 | 0.77 |
| C147R120 | tetratricopeptide repeat protein 40 | 0.76 | 0.72 | 0.55 | 1.02 |
| C096R085 | ras-interacting protein 1 | 0.76 | 0.61 | 0.99 | 0.75 |
| C255R076 | UNKNOWN | 0.76 | 0.70 | 0.66 | 0.90 |
| C204R057 | lariat debranching enzyme | 0.76 | 0.95 | 0.73 | 0.65 |
| C177R137 | 40s ribosomal protein s10 | 0.76 | 0.76 | 0.75 | 0.76 |
| C194R011 | nuclear distribution protein nude homolog 1-like | 0.76 | 0.67 | 0.72 | 0.89 |
| C124R049 | UNKNOWN | 0.76 | 0.50 | 0.95 | 0.89 |
| C120R070 | endothelin-converting enzyme 2 | 0.76 | 0.81 | 0.84 | 0.61 |
| C118R086 | nad mitochondrial-like | 0.76 | 0.72 | 0.81 | 0.74 |
| C206R024 | dihydrofolate reductase | 0.75 | 0.83 | 0.84 | 0.61 |
| C227R025 | regulator of g-protein signaling 5-like protein | 0.75 | 0.86 | 0.71 | 0.69 |
| C098R135 | reticulon 2 | 0.75 | 0.64 | 0.82 | 0.82 |
| C159R044 | epidermis-type lipoxygenase 3-like | 0.75 | 0.72 | 0.67 | 0.87 |
| C128R056 | UNKNOWN | 0.75 | 0.55 | 0.80 | 0.99 |
| C072R096 | UKNOWN | 0.75 | 0.65 | 0.92 | 0.74 |
| C248R039 | UNKNOWN | 0.75 | 0.69 | 0.66 | 0.94 |
| C099R013 | 4-hydroxyphenylpyruvate dioxygenase-like protein | 0.75 | 0.70 | 0.72 | 0.85 |
| C264R020 | translation machinery-associated protein 16 | 0.75 | 0.91 | 0.66 | 0.73 |
| C111R078 | UKNOWN | 0.75 | 0.67 | 0.68 | 0.90 |
| C243R144 | haus augmin-like complex subunit 6-like | 0.75 | 0.69 | 0.75 | 0.81 |
| C264R125 | nicotinamide riboside kinase 2 | 0.75 | 0.70 | 0.83 | 0.73 |
| C010R028 | dna topoisomerase 2-alpha | 0.75 | 0.78 | 0.77 | 0.69 |
| C205R170 | nebulin-related-anchoring protein | 0.75 | 0.55 | 0.97 | 0.79 |
| C083R044 | lim-domain binding factor 3 | 0.75 | 0.74 | 0.68 | 0.82 |
| C079R070 | dna topoisomerase 2-binding protein 1 | 0.75 | 0.58 | 1.01 | 0.74 |
| C059R005 | trimeric intracellular cation channel type a | 0.75 | 0.95 | 0.58 | 0.75 |
| C149R046 | transcription elongation regulator 1-like | 0.75 | 0.67 | 0.66 | 0.94 |
| C147R007 | UNKNOWN | 0.75 | 0.64 | 0.76 | 0.83 |
| C077R044 | UNKNOWN | 0.75 | 0.58 | 0.85 | 0.85 |
| C266R051 | nucleolar protein 14 | 0.75 | 0.83 | 0.65 | 0.78 |
| C254R073 | allograft inflammatory factor 1 | 0.75 | 0.67 | 0.63 | 0.90 |
| C068R013 | immunoglobulin light chain | 0.75 | 0.79 | 0.56 | 0.91 |
| C169R059 | dnaj homolog subfamily c member 25-like | 0.75 | 0.59 | 0.85 | 0.87 |
| C085R023 | camp-regulated phosphoprotein 19 | 0.75 | 0.59 | 0.86 | 0.78 |
| C004R153 | tropomodulin 4 | 0.75 | 0.67 | 0.75 | 0.84 |
| C179R095 | cdkn2aip n-terminal-like protein | 0.75 | 0.72 | 0.81 | 0.73 |
| C052R082 | elongation factor 2 | 0.75 | 0.72 | 0.67 | 0.85 |
| C172R050 | protein cip2a homolog | 0.75 | 0.80 | 0.78 | 0.67 |
| C209R138 | enhancer of rudimentary homolog | 0.75 | 0.61 | 0.89 | 0.78 |
| C118R149 | UNKNOWN | 0.75 | 0.62 | 0.84 | 0.79 |
| C124R008 | protein canopy homolog 4 | 0.74 | 0.66 | 0.70 | 0.94 |
| C063R091 | homologous-pairing protein 2 homolog | 0.74 | 0.52 | 0.85 | 0.89 |
| C159R115 | cytochrome c oxidase subunit 4 isoform mitochondrial precursor | 0.74 | 0.67 | 0.74 | 0.82 |
| C181R067 | reticulon 2b | 0.74 | 0.73 | 0.63 | 0.92 |
| C244R015 | homeobox protein 3ab | 0.74 | 0.69 | 0.62 | 0.91 |
| C001R099 | UNKNOWN | 0.74 | 0.59 | 0.73 | 0.90 |
| C003R047 | inner centromere protein | 0.74 | 0.75 | 0.73 | 0.75 |
| C107R105 | cellular retinoic acid-binding protein 2-like | 0.74 | 0.62 | 1.03 | 0.66 |
| C006R050 | inner centromere protein | 0.74 | 0.82 | 0.64 | 0.79 |
| C072R014 | cholesteryl ester transfer protein | 0.74 | 0.81 | 0.51 | 0.94 |
| C258R021 | UNKNOWN | 0.74 | 0.78 | 0.66 | 0.81 |
| C129R093 | UKNOWN | 0.74 | 0.57 | 1.19 | 0.62 |
| C151R012 | taxilin beta muscle-derived protein 77-like | 0.74 | 1.03 | 0.46 | 0.84 |
| C073R133 | UNKNOWN | 0.74 | 0.80 | 0.65 | 0.77 |
| C055R090 | rna-binding protein 38-like | 0.74 | 0.60 | 0.79 | 0.89 |
| C261R017 | collagen alpha-3 chain-like | 0.74 | 0.65 | 0.75 | 0.82 |
| C142R098 | UKNOWN | 0.74 | 0.62 | 0.69 | 0.90 |
| C211R109 | UKNOWN | 0.74 | 0.76 | 0.69 | 0.78 |
| C157R015 | aspartyl aminopeptidase | 0.74 | 0.75 | 0.61 | 0.87 |
| C092R066 | interferon-related developmental regulator 2 | 0.74 | 0.63 | 0.97 | 0.68 |
| C207R016 | 39s ribosomal protein mitochondrial | 0.74 | 0.85 | 0.78 | 0.63 |
| C063R052 | major egg antigen | 0.74 | 0.70 | 0.71 | 0.83 |
| C206R027 | UNKNOWN | 0.74 | 0.64 | 0.75 | 0.83 |
| C193R017 | general transcription factor iie subunit 1 | 0.74 | 0.63 | 0.68 | 0.91 |
| C263R062 | ox-2 membrane glyco | 0.74 | 0.97 | 0.78 | 0.48 |
| C252R074 | targeting protein for xklp2 | 0.74 | 0.72 | 0.83 | 0.66 |
| C094R014 | nuclear transport factor 2 | 0.74 | 0.72 | 0.71 | 0.79 |
| C074R053 | troponin slow skeletal and cardiac muscles | 0.74 | 0.61 | 0.98 | 0.69 |
| C021R144 | UNKNOWN | 0.74 | 0.59 | 0.80 | 0.82 |
| C075R111 | acyl- synthetase family member mitochondrial | 0.74 | 0.60 | 0.92 | 0.72 |
| C143R060 | UNKNOWN | 0.74 | 0.48 | 0.76 | 0.95 |
| C116R096 | 60s ribosomal protein l22-like 1-like | 0.74 | 0.79 | 0.67 | 0.73 |
| C216R131 | triadin | 0.74 | 0.61 | 0.84 | 0.77 |
| C031R021 | lumican | 0.74 | 0.46 | 0.80 | 1.02 |
| C250R019 | small subunit processome component 20 homolog | 0.74 | 0.65 | 0.64 | 0.88 |
| C054R036 | tsc22 domain family protein 3-like | 0.73 | 0.66 | 0.72 | 0.84 |
| C176R122 | UNKNOWN | 0.73 | 0.64 | 0.86 | 0.74 |
| C167R086 | rrna-processing protein fcf1 homolog | 0.73 | 0.78 | 0.74 | 0.70 |
| C166R008 | low quality protein: coagulation factor viii-like | 0.73 | 0.78 | 0.58 | 0.84 |
| C102R168 | vasorin-like isoform x1 | 0.73 | 0.44 | 1.02 | 0.84 |
| C201R166 | solute carrier family facilitated glucose transporter member 3-like | 0.73 | 0.60 | 0.86 | 0.75 |
| C259R025 | mrna turnover protein 4 homolog | 0.73 | 0.76 | 0.71 | 0.73 |
| C066R097 | nicotinamide riboside kinase 2 | 0.73 | 0.53 | 0.94 | 0.81 |
| C234R113 | chromosome-associated kinesin kif4a | 0.73 | 0.60 | 0.96 | 0.70 |
| C202R083 | fatty acid-binding intestinal | 0.73 | 0.63 | 1.00 | 0.66 |
| C228R095 | condensin complex subunit 1-like | 0.73 | 0.87 | 0.69 | 0.66 |
| C159R030 | sulfotransferase 6b1 | 0.73 | 0.62 | 0.76 | 0.85 |
| C125R071 | set and mynd domain-containing protein 1-like isoform 1 | 0.73 | 0.69 | 0.64 | 0.85 |
| C255R047 | origin recognition complex subunit 5 | 0.73 | 0.71 | 0.82 | 0.67 |
| C232R031 | UNKNOWN | 0.73 | 0.60 | 0.56 | 0.96 |
| C255R040 | lim-domain binding factor 3 | 0.73 | 0.87 | 0.66 | 0.69 |
| C260R058 | transmembrane protein 88-like | 0.73 | 0.55 | 0.79 | 0.87 |
| C115R138 | myosin binding protein cardiac | 0.73 | 0.75 | 0.72 | 0.71 |
| C229R063 | von willebrand factor d and egf domain-containing | 0.73 | 0.81 | 0.64 | 0.74 |
| C168R019 | krueppel-like factor 11-like | 0.73 | 0.59 | 0.73 | 0.87 |
| C157R071 | dna-directed rna polymerase i subunit rpa1 | 0.73 | 0.61 | 0.80 | 0.80 |
| C119R005 | structural maintenance of chromosomes protein 4 | 0.73 | 0.75 | 0.65 | 0.79 |
| C201R116 | dna repair protein rad51 homolog 1 | 0.73 | 0.76 | 0.85 | 0.58 |
| C155R037 | basal cell adhesion molecule precursor | 0.73 | 0.64 | 0.75 | 0.80 |
| C138R131 | von willebrand factor d and egf domain-containing | 0.73 | 0.75 | 0.75 | 0.69 |
| C091R016 | probable c- | 0.73 | 0.78 | 0.62 | 0.79 |
| C077R054 | transmembrane protein 131-like | 0.73 | 0.44 | 0.92 | 0.89 |
| C245R120 | clip-associating protein 2-like | 0.73 | 0.44 | 0.90 | 0.91 |
| C144R062 | nadp-dependent malic mitochondrial-like | 0.73 | 0.69 | 0.66 | 0.85 |
| C166R011 | ran gtpase-activating protein 1-like | 0.73 | 0.67 | 0.66 | 0.88 |
| C129R146 | catenin beta-1 | 0.73 | 0.72 | 0.75 | 0.72 |
| C202R057 | nuclear autoantigenic sperm protein (histone-binding) | 0.73 | 0.68 | 0.82 | 0.70 |
| C230R011 | neutral alpha-glucosidase ab-like | 0.73 | 0.70 | 0.63 | 0.87 |
| C077R015 | UNKNOWN | 0.73 | 0.46 | 0.82 | 1.01 |
| C021R009 | lim-domain binding factor 3 | 0.73 | 0.66 | 0.60 | 0.94 |
| C144R158 | trans- -enoyl- reductase-like | 0.73 | 0.66 | 0.62 | 0.88 |
| C214R057 | UNKNOWN | 0.73 | 0.66 | 0.79 | 0.73 |
| C248R111 | dual specificity protein kinase ttk | 0.73 | 0.70 | 0.67 | 0.83 |
| C142R031 | transforming growth factor-beta-induced protein ig-h3 | 0.73 | 0.62 | 0.81 | 0.77 |
| C018R118 | fermitin family homolog 1 | 0.73 | 0.46 | 0.93 | 0.94 |
| C213R105 | UNKNOWN | 0.72 | 0.65 | 0.78 | 0.76 |
| C162R130 | protein regulator of cytokinesis 1 | 0.72 | 0.67 | 0.92 | 0.60 |
| C232R021 | zinc finger protein 207 | 0.72 | 0.58 | 0.79 | 0.83 |
| C100R109 | 26s proteasome complex subunit dss1 | 0.72 | 0.68 | 0.73 | 0.77 |
| C230R162 | cofilin-2 | 0.72 | 0.56 | 0.78 | 0.85 |
| C056R050 | polyadenylate-binding protein 4 | 0.72 | 0.77 | 0.71 | 0.70 |
| C231R007 | UNKNOWN | 0.72 | 0.63 | 0.71 | 0.82 |
| C141R170 | tonsoku-like protein | 0.72 | 0.65 | 0.62 | 0.88 |
| C244R099 | UNKNOWN | 0.72 | 0.54 | 0.68 | 0.91 |
| C207R024 | UKNOWN | 0.72 | 0.71 | 0.63 | 0.84 |
| C176R058 | disabled homolog 2 isoform 1 | 0.72 | 0.73 | 0.77 | 0.69 |
| C039R082 | perforin-1-like | 0.72 | 0.61 | 0.69 | 0.83 |
| C158R046 | myomesin-3 | 0.72 | 0.62 | 0.64 | 0.90 |
| C199R109 | small nuclear ribonucleoprotein f | 0.72 | 0.67 | 0.88 | 0.67 |
| C151R067 | musculoskeletal embryonic nuclear protein 1 | 0.72 | 0.71 | 0.62 | 0.88 |
| C244R012 | t-box 1 | 0.72 | 0.46 | 0.78 | 0.98 |
| C049R111 | acetolactate synthase-like protein | 0.72 | 0.73 | 0.87 | 0.63 |
| C106R090 | high choriolytic enzyme 1 precursor | 0.72 | 0.57 | 0.80 | 0.85 |
| C238R116 | ankyrin repeat domain-containing protein 1-like | 0.72 | 0.63 | 0.85 | 0.68 |
| C048R037 | UNKNOWN | 0.72 | 0.87 | 0.90 | 0.49 |
| C158R037 | histone-arginine methyltransferase carm1 | 0.72 | 0.73 | 0.61 | 0.81 |
| C135R063 | zinc finger protein 135-like | 0.72 | 0.56 | 0.90 | 0.78 |
| C090R149 | UNKNOWN | 0.72 | 0.51 | 0.84 | 0.83 |
| C125R039 | laminin subunit beta-2-like | 0.72 | 0.69 | 0.63 | 0.84 |
| C103R023 | UKNOWN | 0.72 | 0.41 | 0.83 | 0.92 |
| C085R011 | UKNOWN | 0.72 | 0.90 | 0.38 | 0.88 |
| C233R161 | kinesin-like protein kif11 | 0.72 | 0.78 | 0.78 | 0.62 |
| C260R098 | procollagen c-endopeptidase enhancer 2-like | 0.72 | 0.63 | 0.86 | 0.69 |
| C021R155 | smoothelin-like protein 2-like | 0.72 | 0.51 | 0.79 | 0.85 |
| C120R115 | rho-related gtp-binding protein | 0.72 | 0.70 | 0.72 | 0.73 |
| C047R128 | protein apcdd1-like | 0.72 | 0.53 | 1.08 | 0.69 |
| C169R048 | heat shock cognate 71 kda | 0.72 | 0.71 | 0.56 | 0.90 |
| C171R162 | ubiquitin-like modifier-activating enzyme 1-like | 0.72 | 0.46 | 0.88 | 0.83 |
| C177R128 | UKNOWN | 0.72 | 0.94 | 0.82 | 0.47 |
| C198R157 | UNKNOWN | 0.72 | 0.58 | 0.67 | 0.88 |
| C204R020 | nipped-b-like protein | 0.72 | 0.62 | 0.74 | 0.78 |
| C123R129 | fish virus induced trim protein | 0.72 | 0.55 | 0.87 | 0.81 |
| C260R062 | mitotic checkpoint serine threonine-protein kinase bub1 | 0.72 | 0.61 | 0.84 | 0.70 |
| C178R073 | dep domain-containing protein 7-like | 0.72 | 0.83 | 0.89 | 0.52 |
| C154R042 | UNKNOWN | 0.71 | 0.62 | 0.71 | 0.82 |
| C062R074 | kinesin-like protein kif20b | 0.71 | 0.88 | 0.61 | 0.70 |
| C186R121 | complement c4-like | 0.71 | 0.35 | 0.95 | 0.96 |
| C181R064 | guanidinoacetate n-methyltransferase | 0.71 | 0.75 | 0.61 | 0.78 |
| C120R016 | liprin-beta-2- partial | 0.71 | 0.57 | 0.83 | 0.75 |
| C122R078 | UNKNOWN | 0.71 | 0.39 | 1.02 | 0.89 |
| C021R017 | cytoskeleton-associated protein 2 | 0.71 | 0.66 | 0.79 | 0.71 |
| C232R006 | UNKNOWN | 0.71 | 0.72 | 0.55 | 0.89 |
| C224R013 | forkhead box protein n3-like | 0.71 | 0.54 | 0.72 | 0.88 |
| C056R114 | heat repeat-containing protein 3 | 0.71 | 0.61 | 0.78 | 0.78 |
| C122R121 | tyrosyl-dna phosphodiesterase 2-like | 0.71 | 0.54 | 0.83 | 0.80 |
| C209R137 | UNKNOWN | 0.71 | 0.57 | 0.79 | 0.73 |
| C238R022 | histone-arginine methyltransferase carm1-like | 0.71 | 0.60 | 0.81 | 0.76 |
| C026R169 | fanconi anemia group d2 | 0.71 | 0.67 | 0.83 | 0.67 |
| C148R122 | protein ect2-like | 0.71 | 0.77 | 0.81 | 0.57 |
| C108R169 | solute carrier family 30 (zinc transporter) member 1 | 0.71 | 0.64 | 0.83 | 0.71 |
| C165R019 | sodium channel subunit beta-1-like | 0.71 | 0.58 | 0.71 | 0.89 |
| C104R068 | myozenin 1 | 0.71 | 0.59 | 0.70 | 0.85 |
| C264R138 | nucleosome-remodeling factor subunit bptf | 0.71 | 0.49 | 0.77 | 0.90 |
| C233R116 | transferrin precursor | 0.71 | 0.73 | 0.49 | 0.97 |
| C245R019 | pleckstrin homology domain-containing family m member 1 | 0.71 | 0.62 | 0.64 | 0.83 |
| C066R037 | thrombospondin-3 isoform 3 | 0.71 | 0.59 | 0.77 | 0.77 |
| C148R165 | mitotic spindle assembly checkpoint protein mad2a | 0.71 | 0.75 | 0.97 | 0.46 |
| C155R170 | homeobox protein hox-b2a-like | 0.71 | 0.58 | 0.89 | 0.70 |
| C137R058 | inhibitor of nuclear factor kappa-b kinase-interacting protein isoform 1 | 0.71 | 0.58 | 0.76 | 0.83 |
| C018R027 | UNKNOWN | 0.71 | 0.76 | 0.71 | 0.64 |
| C112R163 | serine threonine-protein kinase 35-like | 0.70 | 0.69 | 0.73 | 0.69 |
| C138R017 | b-cell receptor-associated protein 29 | 0.70 | 0.71 | 0.56 | 0.83 |
| C154R064 | macrophage receptor marco | 0.70 | 0.65 | 0.72 | 0.73 |
| C184R049 | translation initiation factor eif-2b subunit delta-like | 0.70 | 0.47 | 0.97 | 0.86 |
| C161R007 | forkhead box protein l1-like | 0.70 | 0.72 | 0.57 | 0.87 |
| C118R080 | protein polybromo-1 | 0.70 | 0.57 | 0.73 | 0.81 |
| C250R023 | probable phospholipid-transporting atpase vd-like | 0.70 | 0.48 | 0.80 | 0.86 |
| C020R012 | solute carrier family 41 member 3-like isoform 1 | 0.70 | 0.80 | 0.55 | 0.75 |
| C194R062 | mitochondrial nadh-ubiquinone oxidoreductase 75 kda subunit | 0.70 | 0.43 | 1.08 | 0.85 |
| C221R097 | far upstream element-binding protein 2-like | 0.70 | 0.45 | 0.79 | 0.89 |
| C241R063 | cysteine and histidine-rich domain-containing protein 1-like | 0.70 | 0.78 | 0.55 | 0.79 |
| C150R052 | 40s ribosomal protein s10 | 0.70 | 0.60 | 0.67 | 0.88 |
| C169R060 | UNKNOWN | 0.70 | 0.67 | 0.62 | 0.81 |
| C176R150 | non-syndromic hearing impairment protein 5 | 0.70 | 0.66 | 0.68 | 0.74 |
| C263R149 | cd3 epsilon | 0.70 | 0.80 | 0.57 | 0.75 |
| C138R160 | lim-domain binding factor 3 | 0.70 | 0.58 | 0.78 | 0.75 |
| C142R032 | cysteine and histidine-rich domain-containing protein 1-like | 0.70 | 0.72 | 0.57 | 0.80 |
| C140R003 | lysozyme g | 0.70 | 0.65 | 0.64 | 0.79 |
| C213R109 | kinesin-like protein kif23 | 0.70 | 0.91 | 0.61 | 0.60 |
| C122R033 | whey acidic protein precursor | 0.70 | 0.71 | 0.48 | 0.87 |
| C203R016 | n-acetyltransferase esco2-like | 0.70 | 0.89 | 0.61 | 0.64 |
| C125R129 | plastin-1 | 0.70 | 0.46 | 0.89 | 0.85 |
| C102R043 | myozenin 1-like | 0.69 | 0.67 | 0.58 | 0.85 |
| C232R118 | rna-binding protein fus | 0.69 | 0.43 | 0.88 | 0.85 |
| C224R128 | UNKNOWN | 0.69 | 0.67 | 0.81 | 0.61 |
| C183R018 | UNKNOWN | 0.69 | 0.63 | 0.52 | 0.90 |
| C188R033 | zinc finger and scan domain-containing protein 25-like isoform x1 | 0.69 | 0.52 | 0.82 | 0.78 |
| C260R027 | tetratricopeptide repeat protein 39a-like | 0.69 | 0.48 | 0.64 | 0.95 |
| C110R010 | actin-related protein 2 3 complex subunit 2 | 0.69 | 0.58 | 0.61 | 1.01 |
| C205R023 | dna mismatch repair protein msh2-like | 0.69 | 0.98 | 0.56 | 0.55 |
| C166R010 | plac8-like protein 1 | 0.69 | 0.62 | 0.53 | 0.86 |
| C249R065 | ankyrin repeat and socs box protein 14-like | 0.69 | 0.53 | 0.74 | 0.81 |
| C020R060 | nck-associated protein 1-like | 0.69 | 0.42 | 0.83 | 0.95 |
| C251R131 | serine threonine-protein kinase greatwall-like | 0.69 | 0.55 | 0.73 | 0.79 |
| C043R036 | splicing factor 3b subunit 1 | 0.69 | 0.51 | 0.89 | 0.79 |
| C157R055 | UNKNOWN | 0.69 | 0.51 | 0.65 | 0.89 |
| C045R067 | transporter-associated with antigen processing 1 | 0.69 | 0.60 | 0.76 | 0.72 |
| C136R130 | telomeric repeat-binding factor 2 | 0.69 | 0.39 | 0.91 | 0.81 |
| C207R092 | nuclear transport factor 2 | 0.69 | 0.55 | 0.86 | 0.75 |
| C154R022 | non-syndromic hearing impairment protein 5 | 0.69 | 0.60 | 0.58 | 0.84 |
| C153R133 | UNKNOWN | 0.69 | 0.52 | 0.83 | 0.76 |
| C118R101 | protein-tyrosine kinase 2-beta-like | 0.69 | 0.51 | 0.74 | 0.83 |
| C156R042 | dna-directed rna polymerases and iii subunit rpabc4 | 0.69 | 0.56 | 0.87 | 0.70 |
| C159R032 | rotatin | 0.69 | 0.53 | 0.69 | 0.84 |
| C213R078 | UNKNOWN | 0.69 | 0.51 | 0.77 | 0.81 |
| C074R021 | UNKNOWN | 0.69 | 0.64 | 0.64 | 0.79 |
| C170R129 | atp-dependent dna helicase q4 | 0.69 | 0.51 | 0.81 | 0.75 |
| C187R163 | cell division control protein 2 homolog | 0.68 | 0.69 | 0.75 | 0.61 |
| C039R096 | myogenin | 0.68 | 0.44 | 0.92 | 0.73 |
| C029R028 | adenylosuccinate lyase | 0.68 | 0.52 | 0.71 | 0.93 |
| C027R031 | UNKNOWN | 0.68 | 0.74 | 0.62 | 0.70 |
| C257R035 | atp-binding cassette sub-family f member 3-like | 0.68 | 0.93 | 0.60 | 0.54 |
| C233R016 | pcna-associated factor | 0.68 | 0.73 | 0.66 | 0.65 |
| C178R159 | alpha-aminoadipic semialdehyde dehydrogenase | 0.68 | 0.45 | 0.86 | 0.77 |
| C030R055 | atpase family aaa domain-containing protein 2 | 0.68 | 0.63 | 0.75 | 0.68 |
| C099R026 | esophageal cancer-related gene 4 protein precursor | 0.68 | 0.67 | 0.59 | 0.80 |
| C229R120 | centrosomal protein of 135 kda | 0.68 | 0.37 | 0.71 | 0.98 |
| C175R061 | cytochrome c oxidase polypeptide vic-2 | 0.68 | 0.49 | 0.78 | 0.89 |
| C249R024 | lim-domain binding factor 3 | 0.68 | 0.80 | 0.71 | 0.53 |
| C076R085 | isotocin precursor | 0.68 | 0.71 | 0.90 | 0.47 |
| C135R074 | kelch repeat and btb domain-containing protein 10-like | 0.68 | 0.58 | 0.67 | 0.79 |
| C215R147 | UKNOWN | 0.68 | 0.56 | 0.84 | 0.71 |
| C221R127 | tripartite motif-containing protein 16-like | 0.68 | 0.61 | 0.45 | 0.94 |
| C181R072 | filamin a-interacting protein 1-like | 0.68 | 0.55 | 0.72 | 0.77 |
| C064R015 | UNKNOWN | 0.68 | 0.43 | 0.66 | 0.90 |
| C020R126 | dual specificity protein kinase ttk-like | 0.68 | 0.57 | 0.76 | 0.74 |
| C056R003 | traf family member-associated nf-kappa-b activator-like | 0.68 | 1.06 | 0.42 | 0.63 |
| C071R076 | zz-type zinc finger-containing protein 3 | 0.68 | 0.43 | 0.77 | 0.93 |
| C118R014 | t-complex protein 1 subunit theta | 0.68 | 0.51 | 0.85 | 0.73 |
| C073R026 | dna damage-inducible transcript 4-like | 0.68 | 0.69 | 0.71 | 0.61 |
| C233R147 | myb-binding protein 1a | 0.67 | 0.62 | 0.72 | 0.68 |
| C137R035 | very long-chain specific acyl- mitochondrial isoform 1 | 0.67 | 0.53 | 0.62 | 0.91 |
| C116R024 | gonadotropin-releasing hormone | 0.67 | 0.50 | 0.93 | 0.74 |
| C014R096 | 2 -deoxynucleoside 5 -phosphate n-hydrolase 1 | 0.67 | 0.89 | 0.61 | 0.58 |
| C062R112 | phosphatidylinositol- -bisphosphate 3-kinase catalytic subunit gamma isoform-like | 0.67 | 0.57 | 0.63 | 0.81 |
| C112R081 | e3 ubiquitin-protein ligase rnf130 | 0.67 | 0.55 | 0.81 | 0.69 |
| C084R013 | cleavage and polyadenylation specificity factor subunit 7 | 0.67 | 0.57 | 0.62 | 0.88 |
| C185R072 | adenylosuccinate lyase | 0.67 | 0.60 | 0.60 | 0.89 |
| C137R043 | UNKNOWN | 0.67 | 0.41 | 0.59 | 1.34 |
| C094R043 | trans- -dihydrobenzene- -diol dehydrogenase-like | 0.67 | 0.44 | 0.92 | 0.80 |
| C161R081 | atp-binding cassette sub-family b member 9-like | 0.67 | 0.49 | 0.79 | 0.74 |
| C071R116 | UNKNOWN | 0.67 | 0.47 | 0.80 | 0.88 |
| C063R169 | dna mismatch repair protein msh2-like | 0.67 | 0.56 | 0.79 | 0.68 |
| C059R123 | sperm-associated antigen 5 | 0.67 | 0.69 | 0.82 | 0.53 |
| C080R082 | cyclin b | 0.67 | 0.85 | 0.62 | 0.55 |
| C147R057 | alpha cardiac muscle 1 | 0.67 | 0.52 | 0.70 | 0.82 |
| C111R080 | tumor necrosis factor ligand superfamily member 13b | 0.67 | 0.53 | 0.95 | 0.65 |
| C010R015 | cd276 antigen-like | 0.67 | 0.70 | 0.52 | 0.80 |
| C165R066 | rho gtpase-activating protein 24 isoform 2 | 0.67 | 0.60 | 0.68 | 0.74 |
| C094R030 | UNKNOWN | 0.67 | 0.69 | 0.74 | 0.60 |
| C245R046 | mucosa associated lymphoid tissue lymphoma translocation gene 1 | 0.67 | 0.55 | 0.72 | 0.77 |
| C263R056 | neuralized-like protein 2 | 0.67 | 0.75 | 0.49 | 0.76 |
| C221R134 | inter-alpha-trypsin inhibitor heavy chain h3-like | 0.67 | 0.38 | 0.75 | 0.90 |
| C084R169 | a2 protein | 0.67 | 0.43 | 0.80 | 0.87 |
| C052R059 | UNKNOWN | 0.67 | 0.56 | 0.63 | 0.80 |
| C040R096 | UNKNOWN | 0.67 | 0.42 | 0.78 | 0.89 |
| C186R010 | eukaryotic peptide chain release factor subunit 1 | 0.67 | 0.51 | 0.64 | 0.96 |
| C019R035 | f-box wd repeat-containing protein 5 | 0.67 | 0.54 | 0.63 | 0.84 |
| C099R123 | phosducin-like protein | 0.66 | 0.46 | 0.90 | 0.72 |
| C148R007 | rho-related gtp-binding protein | 0.66 | 0.85 | 0.43 | 0.78 |
| C072R114 | UKNOWN | 0.66 | 0.51 | 0.80 | 0.74 |
| C219R038 | UKNOWN | 0.66 | 0.53 | 0.64 | 0.76 |
| C094R169 | myomesin-1 isoform 1 | 0.66 | 0.48 | 0.56 | 0.94 |
| C227R008 | platelet-derived growth factor receptor beta | 0.66 | 0.81 | 0.45 | 0.81 |
| C107R024 | hypothetical protein BRAFLDRAFT_86387 | 0.66 | 0.42 | 0.75 | 0.93 |
| C011R044 | centromere protein m | 0.66 | 0.60 | 0.72 | 0.68 |
| C175R023 | rna binding protein fox-1 homolog 1-like | 0.66 | 0.55 | 0.62 | 0.84 |
| C058R127 | desmin | 0.66 | 0.58 | 0.50 | 0.86 |
| C229R038 | heat shock protein hsp 90-alpha 1-like | 0.66 | 0.63 | 0.78 | 0.58 |
| C111R089 | UKNOWN | 0.66 | 0.54 | 0.64 | 0.78 |
| C145R103 | sperm-associated antigen 5 | 0.66 | 0.97 | 0.44 | 0.72 |
| C012R013 | cardiac muscle alpha actin 1 | 0.66 | 0.45 | 0.79 | 0.85 |
| C156R098 | beta-taxilin | 0.66 | 0.64 | 0.52 | 0.81 |
| C135R061 | immunoglobulin superfamily member 10 isoform 1 | 0.66 | 0.42 | 0.69 | 0.86 |
| C182R034 | si:dkeyp- protein | 0.66 | 0.61 | 0.62 | 0.74 |
| C223R122 | heat shock protein beta-7 | 0.66 | 0.46 | 0.75 | 0.83 |
| C088R014 | UNKNOWN | 0.66 | 0.42 | 0.72 | 0.92 |
| C160R061 | protein unc-45 homolog b | 0.66 | 0.74 | 0.56 | 0.68 |
| C088R033 | macrophage receptor marco | 0.65 | 0.67 | 0.57 | 0.69 |
| C003R061 | aurora kinase b-like | 0.65 | 0.63 | 0.67 | 0.67 |
| C156R013 | nuclear transport factor 2 | 0.65 | 0.60 | 0.59 | 0.80 |
| C140R150 | UNKNOWN | 0.65 | 0.59 | 0.66 | 0.69 |
| C130R051 | prostaglandin e synthase 3 | 0.65 | 0.61 | 0.67 | 0.67 |
| C035R018 | acidic leucine-rich nuclear phosphoprotein 32 family member b | 0.65 | 0.62 | 0.61 | 0.71 |
| C079R047 | smad nuclear-interacting protein 1 | 0.65 | 0.42 | 1.08 | 0.68 |
| C178R138 | UNKNOWN | 0.65 | 0.42 | 0.82 | 0.79 |
| C014R159 | ephrin type-a receptor 2-like | 0.65 | 0.65 | 0.60 | 0.69 |
| C004R141 | UNKNOWN | 0.65 | 0.41 | 0.87 | 0.72 |
| C165R018 | ubiquitin carboxyl-terminal hydrolase 28-like | 0.65 | 0.44 | 0.50 | 1.03 |
| C161R016 | UKNOWN | 0.65 | 0.53 | 0.78 | 0.65 |
| C238R137 | bchain human vinculin head (1-258) in complex with human vinculin tail (879-1066) | 0.65 | 0.35 | 0.79 | 0.81 |
| C208R126 | UNKNOWN | 0.65 | 0.42 | 1.16 | 0.62 |
| C183R130 | UNKNOWN | 0.65 | 0.49 | 0.56 | 0.78 |
| C261R094 | platelet-derived growth factor receptor beta | 0.65 | 0.58 | 0.59 | 0.81 |
| C116R033 | kelch repeat and btb domain-containing protein 12-like | 0.65 | 0.40 | 0.82 | 0.79 |
| C155R048 | UNKNOWN | 0.65 | 0.68 | 0.64 | 0.61 |
| C146R035 | condensin-2 complex subunit g2 | 0.65 | 0.52 | 0.71 | 0.74 |
| C259R049 | leucine-rich repeat and wd repeat-containing protein 1 | 0.65 | 0.70 | 0.56 | 0.70 |
| C179R074 | gmp reductase 1 | 0.64 | 0.60 | 0.66 | 0.67 |
| C157R130 | UNKNOWN | 0.64 | 0.56 | 0.83 | 0.62 |
| C254R151 | UKNOWN | 0.64 | 0.56 | 0.68 | 0.72 |
| C110R033 | UKNOWN | 0.64 | 0.66 | 0.57 | 0.69 |
| C103R046 | UNKNOWN | 0.64 | 0.54 | 0.70 | 0.73 |
| C071R019 | t-complex protein 1 subunit theta | 0.64 | 0.41 | 0.86 | 0.79 |
| C123R012 | UNKNOWN | 0.64 | 0.60 | 0.61 | 0.74 |
| C006R012 | UNKNOWN | 0.64 | 0.47 | 0.54 | 0.96 |
| C256R034 | lim-domain binding factor 3 | 0.64 | 0.45 | 0.81 | 0.68 |
| C247R129 | nucleoprotein tpr | 0.64 | 0.59 | 0.53 | 0.78 |
| C013R125 | glutathione peroxidase 4a | 0.64 | 0.43 | 0.76 | 0.88 |
| C246R044 | chromosome transmission fidelity protein 8 homolog | 0.64 | 0.53 | 0.76 | 0.62 |
| C099R024 | ap-3 complex subunit delta-1 | 0.64 | 0.44 | 0.71 | 0.95 |
| C173R066 | muscle-related coiled-coil | 0.64 | 0.53 | 0.70 | 0.68 |
| C084R051 | myomesin 2 | 0.64 | 0.53 | 0.61 | 0.79 |
| C017R024 | p2y purinoceptor 8 | 0.64 | 0.57 | 0.45 | 0.82 |
| C002R144 | myoblast determination protein 2 | 0.64 | 0.37 | 0.82 | 0.94 |
| C114R088 | alpha-actinin-2 isoform 2 | 0.64 | 0.42 | 0.86 | 0.77 |
| C174R024 | UNKNOWN | 0.64 | 0.50 | 0.58 | 0.91 |
| C165R011 | abnormal spindle-like microcephaly-associated protein homolog | 0.64 | 0.77 | 0.51 | 0.62 |
| C099R030 | pogo transposable element with krab domain-like | 0.64 | 0.36 | 0.77 | 0.86 |
| C057R043 | disintegrin and metalloproteinase domain-containing protein 33 | 0.64 | 0.42 | 0.82 | 0.74 |
| C239R104 | kinesin-like protein kif20b-like | 0.63 | 0.57 | 0.69 | 0.65 |
| C082R133 | UNKNOWN | 0.63 | 0.39 | 0.87 | 0.75 |
| C100R002 | matrix-remodeling-associated protein 8-like | 0.63 | 0.45 | 0.84 | 0.73 |
| C093R148 | dna polymerase delta catalytic subunit-like | 0.63 | 0.62 | 0.65 | 0.63 |
| C108R133 | alpha-ketoglutarate-dependent dioxygenase alkb homolog 2 | 0.63 | 0.50 | 0.97 | 0.55 |
| C149R067 | selenoprotein p | 0.63 | 0.53 | 0.72 | 0.65 |
| C114R022 | novel protein vertebrate nebulin | 0.63 | 0.41 | 0.74 | 0.80 |
| C125R109 | acidic coiled-coil containing protein 3 | 0.63 | 0.69 | 0.66 | 0.53 |
| C253R006 | tripartite motif-containing protein 16-like | 0.63 | 0.68 | 0.34 | 0.89 |
| C221R048 | upf0193 protein evg1 isoform 2 | 0.63 | 0.40 | 1.14 | 0.62 |
| C137R106 | UNKNOWN | 0.63 | 0.38 | 0.73 | 0.84 |
| C074R025 | gonadotropin-releasing hormone | 0.62 | 0.56 | 0.95 | 0.52 |
| C201R124 | sumo-activating enzyme subunit 2 | 0.62 | 0.49 | 0.89 | 0.56 |
| C128R044 | PREDICTED: uncharacterized protein C1orf112 homolog isoform X1 | 0.62 | 0.43 | 0.93 | 0.63 |
| C009R091 | fibulin-1 precursor | 0.62 | 0.43 | 0.73 | 0.83 |
| C065R038 | nadh dehydrogenase subunit 3 | 0.62 | 0.39 | 0.69 | 0.92 |
| C160R062 | mothers against decapentaplegic homolog 1 | 0.62 | 0.33 | 0.85 | 0.94 |
| C157R060 | ataxin-2-like isoform x4 | 0.62 | 0.38 | 0.65 | 0.90 |
| C065R162 | set and mynd domain-containing protein 1-like isoform 2 | 0.62 | 0.68 | 0.51 | 0.68 |
| C179R010 | light chain 12 | 0.62 | 0.43 | 0.57 | 0.99 |
| C118R056 | tyrosine-protein kinase transforming protein fps-like | 0.62 | 0.35 | 0.96 | 0.78 |
| C153R058 | dep domain-containing protein 1a isoform 2 | 0.62 | 0.50 | 1.02 | 0.46 |
| C164R085 | prolyl endopeptidase-like | 0.62 | 0.50 | 0.76 | 0.65 |
| C228R010 | integrator complex subunit 4 | 0.62 | 0.55 | 0.56 | 0.73 |
| C016R137 | thyroglobulin | 0.62 | 0.68 | 0.61 | 0.58 |
| C237R008 | protein casc5-like | 0.61 | 0.49 | 0.56 | 0.82 |
| C204R156 | swi snf-related matrix-associated actin-dependent regulator of chromatin subfamily a-like protein 1 | 0.61 | 0.66 | 0.69 | 0.51 |
| C062R015 | UNKNOWN | 0.61 | 0.59 | 0.43 | 0.82 |
| C169R125 | nucleoprotein tpr | 0.61 | 0.49 | 0.76 | 0.68 |
| C203R027 | complement component c1q receptor | 0.61 | 0.43 | 0.52 | 0.97 |
| C256R075 | sarcosine mitochondrial | 0.61 | 0.53 | 0.56 | 0.70 |
| C213R107 | cell division control protein 6 homolog | 0.61 | 0.45 | 0.84 | 0.63 |
| C241R010 | t-cell surface glycoprotein cd3 zeta chain precursor | 0.61 | 0.55 | 0.52 | 0.71 |
| C153R037 | alpha cardiac muscle 1 | 0.61 | 0.54 | 0.52 | 0.77 |
| C152R011 | collagen alpha-1 chain | 0.61 | 0.52 | 0.53 | 0.75 |
| C146R110 | myosin binding protein cardiac | 0.61 | 0.62 | 0.49 | 0.69 |
| C073R043 | tetraspanin-3-like isoform 1 | 0.61 | 0.28 | 0.96 | 0.72 |
| C233R067 | protein dpy-30 homolog | 0.61 | 0.41 | 0.98 | 0.66 |
| C145R100 | myosin heavy chain | 0.61 | 0.46 | 0.64 | 0.74 |
| C118R069 | UNKNOWN | 0.60 | 0.37 | 0.72 | 0.91 |
| C264R139 | desmin | 0.60 | 0.54 | 0.48 | 0.76 |
| C061R059 | protein kinase c and casein kinase substrate in neurons protein 2-like | 0.60 | 0.38 | 1.03 | 0.75 |
| C089R008 | myosin-binding protein fast-type-like | 0.60 | 0.53 | 0.41 | 0.89 |
| C206R039 | UNKNOWN | 0.60 | 0.49 | 0.74 | 0.59 |
| C217R085 | UNKNOWN | 0.60 | 0.39 | 0.65 | 0.75 |
| C118R155 | s-antigen partial | 0.60 | 0.57 | 0.81 | 0.52 |
| C018R051 | endoplasmin- partial | 0.60 | 0.62 | 0.70 | 0.45 |
| C167R014 | PREDICTED: uncharacterized protein LOC101486179 | 0.60 | 0.54 | 0.43 | 0.76 |
| C046R150 | heat shock protein beta-1 | 0.60 | 0.57 | 0.52 | 0.74 |
| C241R059 | matrilin-2 precursor | 0.60 | 0.42 | 0.61 | 0.82 |
| C047R111 | myotilin isoform 1 | 0.60 | 0.25 | 1.10 | 0.76 |
| C252R013 | c14orf159 protein | 0.60 | 0.39 | 0.41 | 1.02 |
| C099R019 | UNKNOWN | 0.60 | 0.61 | 0.50 | 0.67 |
| C236R043 | interferon inducible mx protein | 0.59 | 0.53 | 0.83 | 0.48 |
| C067R162 | cyclic amp-dependent transcription factor atf-4 | 0.59 | 0.38 | 0.67 | 0.77 |
| C148R140 | e3 ubiquitin-protein ligase synoviolin-like | 0.59 | 0.29 | 0.70 | 0.89 |
| C084R162 | c-c motif chemokine 19 precursor | 0.59 | 0.60 | 0.39 | 0.67 |
| C246R074 | zinc finger protein 51 | 0.59 | 0.55 | 0.63 | 0.63 |
| C090R041 | fatty-acid amide hydrolase 2 | 0.59 | 0.54 | 0.64 | 0.60 |
| C263R059 | ubiquitin-associated protein 2 | 0.59 | 0.35 | 0.65 | 0.81 |
| C072R005 | UNKNOWN | 0.59 | 0.58 | 0.41 | 0.73 |
| C264R084 | protein fam117a-like | 0.59 | 0.56 | 0.47 | 0.73 |
| C139R165 | actinin alpha 2 | 0.59 | 0.41 | 0.77 | 0.72 |
| C013R148 | UNKNOWN | 0.58 | 0.61 | 0.56 | 0.58 |
| C030R046 | prostaglandin e synthase 3 | 0.58 | 0.61 | 0.48 | 0.64 |
| C047R107 | lymphocyte-specific protein tyrosine kinase | 0.58 | 0.37 | 0.78 | 0.71 |
| C198R110 | rrna methyltransferase mitochondrial | 0.58 | 0.36 | 0.62 | 0.74 |
| C106R087 | myocilin-like | 0.58 | 0.49 | 0.59 | 0.66 |
| C135R056 | actin-binding rho-activating | 0.58 | 0.39 | 0.49 | 0.81 |
| C193R122 | lymphocyte-specific protein tyrosine kinase | 0.58 | 0.26 | 1.01 | 0.65 |
| C224R069 | tho complex subunit 5 homolog | 0.58 | 0.31 | 0.91 | 0.68 |
| C023R031 | mhc class ii alpha partial | 0.58 | 0.41 | 0.66 | 0.65 |
| C125R078 | svil protein | 0.57 | 0.39 | 0.69 | 0.85 |
| C163R131 | myogenin | 0.57 | 0.55 | 0.67 | 0.52 |
| C227R065 | nadh dehydrogenase | 0.57 | 0.46 | 1.03 | 0.49 |
| C057R111 | cysteine and glycine-rich protein 3 | 0.57 | 0.44 | 0.62 | 0.65 |
| C064R032 | UNKNOWN | 0.57 | 0.53 | 0.48 | 0.70 |
| C257R041 | UNKNOWN | 0.57 | 0.57 | 0.47 | 0.66 |
| C138R009 | UNKNOWN | 0.57 | 0.26 | 0.63 | 0.98 |
| C052R118 | UNKNOWN | 0.57 | 0.29 | 1.17 | 0.52 |
| C169R047 | myocilin-like | 0.57 | 0.30 | 0.77 | 0.67 |
| C240R162 | alpha cardiac muscle 1 | 0.56 | 0.51 | 0.74 | 0.51 |
| C252R157 | troponin slow skeletal and cardiac muscles | 0.56 | 0.42 | 0.77 | 0.59 |
| C172R117 | calcineurin b homologous protein 2 | 0.56 | 0.42 | 0.62 | 0.70 |
| C260R032 | t-cell surface glycoprotein cd5-like | 0.56 | 0.49 | 0.22 | 0.84 |
| C064R027 | myogenic factor 5 | 0.56 | 0.47 | 0.55 | 0.66 |
| C124R109 | UNKNOWN | 0.56 | 0.44 | 0.48 | 0.75 |
| C013R158 | set and mynd domain-containing protein 1-like isoform 2 | 0.56 | 0.55 | 0.53 | 0.62 |
| C098R103 | fibroblast growth factor 1 | 0.56 | 0.42 | 0.66 | 0.65 |
| C200R010 | t-cell receptor gamma | 0.56 | 0.59 | 0.27 | 0.82 |
| C077R041 | yth domain family protein 2-like | 0.56 | 0.34 | 0.59 | 0.90 |
| C073R023 | cysteine and glycine-rich protein 3 | 0.56 | 0.52 | 0.51 | 0.65 |
| C107R140 | UKNOWN | 0.55 | 0.39 | 0.66 | 0.68 |
| C213R128 | asc-type amino acid transporter 1-like | 0.55 | 0.43 | 0.60 | 0.65 |
| C243R086 | UNKNOWN | 0.55 | 0.40 | 0.43 | 0.76 |
| C226R125 | isoform cra_a | 0.55 | 0.41 | 0.56 | 0.61 |
| C044R040 | ubiquitin-associated protein 1-like | 0.54 | 0.26 | 0.94 | 0.61 |
| C011R090 | serine threonine-protein kinase pak 2-like | 0.54 | 0.24 | 1.39 | 0.58 |
| C160R142 | caveolin-2-like | 0.54 | 0.40 | 0.61 | 0.63 |
| C063R031 | ras gtpase-activating-like protein iqgap3-like | 0.54 | 0.33 | 0.44 | 0.96 |
| C254R062 | inter-alpha-trypsin inhibitor heavy chain h3 | 0.54 | 0.36 | 0.59 | 0.74 |
| C242R008 | UNKNOWN | 0.53 | 0.35 | 0.45 | 0.88 |
| C062R056 | rna binding protein fox-1 homolog 1-like | 0.53 | 0.44 | 0.48 | 0.78 |
| C116R108 | mkl myocardin-like protein 1 | 0.53 | 0.30 | 0.75 | 0.60 |
| C260R055 | myosin binding protein cardiac | 0.53 | 0.39 | 0.62 | 0.63 |
| C266R127 | set and mynd domain-containing protein 1-like isoform 2 | 0.52 | 0.67 | 0.41 | 0.54 |
| C238R102 | beta-crystallin b3 | 0.52 | 0.29 | 0.78 | 0.62 |
| C206R009 | UNKNOWN | 0.52 | 0.38 | 0.46 | 0.72 |
| C237R046 | prostaglandin e synthase 3 | 0.51 | 0.44 | 0.75 | 0.36 |
| C101R089 | thimet oligopeptidase | 0.51 | 0.30 | 0.97 | 0.60 |
| C050R154 | UNKNOWN | 0.51 | 0.27 | 0.63 | 0.77 |
| C010R106 | UNKNOWN | 0.51 | 0.27 | 0.68 | 0.83 |
| C231R077 | structural maintenance of chromosomes protein 4 | 0.51 | 0.29 | 0.71 | 0.77 |
| C025R008 | tropomyosin 4 | 0.51 | 0.48 | 0.49 | 0.58 |
| C246R022 | inter-alpha-trypsin inhibitor heavy chain h3-like | 0.51 | 0.40 | 0.45 | 0.68 |
| C100R025 | telomere-associated protein rif1 isoform x2 | 0.50 | 0.47 | 0.53 | 0.52 |
| C157R006 | solute carrier family 43 member 3-like | 0.50 | 0.54 | 0.36 | 0.59 |
| C200R008 | parvalbumin-7-like isoform x1 | 0.49 | 0.42 | 0.35 | 0.68 |
| C132R101 | replication factor c subunit 3 | 0.49 | 0.15 | 1.38 | 0.62 |
| C197R155 | UNKNOWN | 0.49 | 0.40 | 0.58 | 0.51 |
| C131R010 | UNKNOWN | 0.48 | 0.34 | 0.53 | 0.59 |
| C244R017 | cysteine and glycine-rich protein 3 | 0.48 | 0.43 | 0.46 | 0.56 |
| C103R085 | e3 ubiquitin-protein ligase trim21-like | 0.48 | 0.24 | 0.56 | 0.66 |
| C164R059 | protein | 0.47 | 0.11 | 0.88 | 0.83 |
| C094R067 | UNKNOWN | 0.47 | 0.25 | 0.60 | 0.64 |
| C214R036 | heat shock protein beta-1 | 0.47 | 0.66 | 0.28 | 0.52 |
| C152R152^a^ | ependymin-1 precursor | 0.46 | 0.20 | 0.57 | 0.69 |
| C147R072 | asph protein | 0.46 | 0.25 | 0.51 | 0.78 |
| C228R108 | ttd non-photosensitive 1 protein homolog | 0.46 | 0.25 | 0.49 | 0.95 |
| C233R135 | myosin heavy chain | 0.45 | 0.33 | 0.68 | 0.46 |
| C211R037 | ubiquitin thioesterase partial | 0.44 | 0.23 | 0.43 | 0.79 |
| C113R135 | regulator of chromosome condensation | 0.44 | 0.23 | 0.60 | 0.75 |
| C074R049 | UNKNOWN | 0.43 | 0.30 | 0.54 | 0.49 |
| C020R131 | phosphatidylinositol transfer protein beta isoform isoform 2 | 0.31 | 0.22 | 0.46 | 0.42 |
| C176R119 | apolipoprotein a-i precursor | 0.15 | 0.16 | 0.07 | 0.59 |
| C251R080 | myosin regulatory light chain atrial isoform | 0.10 | 0.06 | 0.12 | 0.13 |
| C086R103 | myosin light polypeptide 4 | 0.06 | 0.04 | 0.08 | 0.08 |

^*^Significantly differentially transcribed genes were annotated in Blast2GO using the BLASTx algorithm and the best BLASTx hit (E-value < 10^-6^) is presented.

^a^ While this gene was initially annotated as ependymin-1, further analyses suggest that microarray contig C152R152 is chimeric (data not shown).

Table B. Overrepresented Gene Ontology (GO) terms associated with the 808 genes differentially transcribed between juvenile Atlantic salmon reared in unenriched and enriched hatchery environments. Significantly over- or under-represented GO terms were identified using a Fisher’s exact test in Blast2GO. Gene parent ontology category (P: biological process; C: cellular component; F: molecular function) and the number of genes (i.e. microarray features) associated with each of the GO terms is indicated. Significance of the Fisher’s exact test (assessed at < 5% FDR) of over- or under-representation of the GO term in the test gene set compared to the reference gene set is also shown.

| GO ID | GO Term | GO Category | FDR | No. Genes | Representation |
| --- | --- | --- | --- | --- | --- |
| GO:0030017 | sarcomere | C | 1.23E-12 | 51 | over |
| GO:0030016 | myofibril | C | 1.23E-12 | 54 | over |
| GO:0044449 | contractile fiber part | C | 1.23E-12 | 52 | over |
| GO:0043292 | contractile fiber | C | 5.91E-12 | 54 | over |
| GO:0030239 | myofibril assembly | P | 5.9E-11 | 32 | over |
| GO:0031674 | I band | C | 9E-11 | 36 | over |
| GO:0055001 | muscle cell development | P | 1.29E-10 | 49 | over |
| GO:0055002 | striated muscle cell development | P | 2.42E-10 | 46 | over |
| GO:0031032 | actomyosin structure organization | P | 4.72E-10 | 32 | over |
| GO:0042692 | muscle cell differentiation | P | 5.9E-09 | 62 | over |
| GO:0048738 | cardiac muscle tissue development | P | 7.02E-09 | 38 | over |
| GO:0055003 | cardiac myofibril assembly | P | 1.24E-08 | 18 | over |
| GO:0030018 | Z disc | C | 1.66E-08 | 29 | over |
| GO:0061061 | muscle structure development | P | 1.79E-08 | 76 | over |
| GO:0051146 | striated muscle cell differentiation | P | 7.35E-08 | 52 | over |
| GO:0010927 | cellular component assembly involved in morphogenesis | P | 1.11E-07 | 36 | over |
| GO:0000279 | M phase | P | 1.38E-07 | 66 | over |
| GO:0008307 | structural constituent of muscle | F | 1.47E-07 | 22 | over |
| GO:0031143 | pseudopodium | C | 1.74E-07 | 12 | over |
| GO:0007507 | heart development | P | 2.49E-07 | 63 | over |
| GO:0055013 | cardiac muscle cell development | P | 2.63E-07 | 19 | over |
| GO:0055006 | cardiac cell development | P | 3.35E-07 | 20 | over |
| GO:0060537 | muscle tissue development | P | 4.5E-07 | 58 | over |
| GO:0014706 | striated muscle tissue development | P | 8.4E-07 | 56 | over |
| GO:0022402 | cell cycle process | P | 9.22E-07 | 106 | over |
| GO:0007517 | muscle organ development | P | 1.08E-06 | 58 | over |
| GO:0000087 | M phase of mitotic cell cycle | P | 1.2E-06 | 50 | over |
| GO:0022403 | cell cycle phase | P | 1.31E-06 | 90 | over |
| GO:0045214 | sarcomere organization | P | 1.31E-06 | 20 | over |
| GO:0030049 | muscle filament sliding | P | 1.38E-06 | 20 | over |
| GO:0033275 | actin-myosin filament sliding | P | 2.03E-06 | 20 | over |
| GO:0070252 | actin-mediated cell contraction | P | 3.44E-06 | 21 | over |
| GO:0006936 | muscle contraction | P | 4.25E-06 | 43 | over |
| GO:0007010 | cytoskeleton organization | P | 4.58E-06 | 92 | over |
| GO:0048285 | organelle fission | P | 1E-05 | 48 | over |
| GO:0007067 | mitosis | P | 1.02E-05 | 46 | over |
| GO:0000280 | nuclear division | P | 1.03E-05 | 46 | over |
| GO:0043232 | intracellular non-membrane-bounded organelle | C | 1.13E-05 | 248 | over |
| GO:0043228 | non-membrane-bounded organelle | C | 1.13E-05 | 248 | over |
| GO:0000278 | mitotic cell cycle | P | 1.42E-05 | 86 | over |
| GO:0003012 | muscle system process | P | 1.88E-05 | 44 | over |
| GO:0060048 | cardiac muscle contraction | P | 2.08E-05 | 19 | over |
| GO:0007049 | cell cycle | P | 5.29E-05 | 122 | over |
| GO:0030048 | actin filament-based movement | P | 6.15E-05 | 22 | over |
| GO:0003015 | heart process | P | 6.35E-05 | 31 | over |
| GO:0060047 | heart contraction | P | 6.35E-05 | 31 | over |
| GO:0007059 | chromosome segregation | P | 6.35E-05 | 25 | over |
| GO:0005856 | cytoskeleton | C | 8.02E-05 | 128 | over |
| GO:0009888 | tissue development | P | 8.06E-05 | 131 | over |
| GO:0000967 | rRNA 5'-end processing | P | 9.03E-05 | 5 | over |
| GO:0000480 | endonucleolytic cleavage in 5'-ETS of tricistronic rRNA transcript (SSU-rRNA, 5.8S rRNA, LSU-rRNA) | P | 9.03E-05 | 5 | over |
| GO:0000472 | endonucleolytic cleavage to generate mature 5'-end of SSU-rRNA from (SSU-rRNA, 5.8S rRNA, LSU-rRNA) | P | 9.03E-05 | 5 | over |
| GO:0034471 | ncRNA 5'-end processing | P | 9.03E-05 | 5 | over |
| GO:0000775 | chromosome, centromeric region | C | 9.15E-05 | 26 | over |
| GO:0055007 | cardiac muscle cell differentiation | P | 0.00011 | 19 | over |
| GO:0030029 | actin filament-based process | P | 0.00014 | 61 | over |
| GO:0008092 | cytoskeletal protein binding | F | 0.000152 | 71 | over |
| GO:0030688 | preribosome, small subunit precursor | C | 0.000162 | 5 | over |
| GO:0044427 | chromosomal part | C | 0.000162 | 58 | over |
| GO:0048513 | organ development | P | 0.00018 | 200 | over |
| GO:0030036 | actin cytoskeleton organization | P | 0.000206 | 57 | over |
| GO:0005694 | chromosome | C | 0.000242 | 64 | over |
| GO:0030274 | LIM domain binding | F | 0.000315 | 7 | over |
| GO:0006941 | striated muscle contraction | P | 0.000388 | 23 | over |
| GO:0035051 | cardiac cell differentiation | P | 0.000404 | 22 | over |
| GO:0051983 | regulation of chromosome segregation | P | 0.000492 | 10 | over |
| GO:0048747 | muscle fiber development | P | 0.00053 | 25 | over |
| GO:0031034 | myosin filament assembly | P | 0.000539 | 10 | over |
| GO:0000966 | RNA 5'-end processing | P | 0.000665 | 5 | over |
| GO:0016020 | membrane | C | 0.000732 | 207 | under |
| GO:0060538 | skeletal muscle organ development | P | 0.000741 | 36 | over |
| GO:0055008 | cardiac muscle tissue morphogenesis | P | 0.000841 | 18 | over |
| GO:0048644 | muscle organ morphogenesis | P | 0.000841 | 19 | over |
| GO:0044085 | cellular component biogenesis | P | 0.000869 | 139 | over |
| GO:0005859 | muscle myosin complex | C | 0.000937 | 9 | over |
| GO:0005643 | nuclear pore | C | 0.000999 | 17 | over |
| GO:0072359 | circulatory system development | P | 0.001284 | 80 | over |
| GO:0072358 | cardiovascular system development | P | 0.001284 | 80 | over |
| GO:0060415 | muscle tissue morphogenesis | P | 0.001284 | 18 | over |
| GO:0000776 | kinetochore | C | 0.001307 | 19 | over |
| GO:0005865 | striated muscle thin filament | C | 0.001499 | 14 | over |
| GO:0031033 | myosin filament organization | P | 0.001499 | 10 | over |
| GO:0016460 | myosin II complex | C | 0.001638 | 10 | over |
| GO:0005080 | protein kinase C binding | F | 0.001736 | 13 | over |
| GO:0010669 | epithelial structure maintenance | P | 0.001996 | 8 | over |
| GO:0032971 | regulation of muscle filament sliding | P | 0.002332 | 6 | over |
| GO:0006996 | organelle organization | P | 0.002482 | 161 | over |
| GO:0019901 | protein kinase binding | F | 0.002625 | 44 | over |
| GO:0000236 | mitotic prometaphase | P | 0.002692 | 15 | over |
| GO:0071690 | cardiac muscle myosin thick filament assembly | P | 0.002796 | 4 | over |
| GO:0019900 | kinase binding | F | 0.002836 | 48 | over |
| GO:0014866 | skeletal myofibril assembly | P | 0.002887 | 10 | over |
| GO:0007519 | skeletal muscle tissue development | P | 0.004007 | 33 | over |
| GO:0005198 | structural molecule activity | F | 0.004241 | 58 | over |
| GO:0010032 | meiotic chromosome condensation | P | 0.004303 | 4 | over |
| GO:0014705 | C zone | C | 0.004654 | 3 | over |
| GO:0000819 | sister chromatid segregation | P | 0.004923 | 12 | over |
| GO:0000447 | endonucleolytic cleavage in ITS1 to separate SSU-rRNA from 5.8S rRNA and LSU-rRNA from tricistronic rRNA transcript (SSU-rRNA, 5.8S rRNA, LSU-rRNA) | P | 0.006405 | 5 | over |
| GO:0019855 | calcium channel inhibitor activity | F | 0.006405 | 5 | over |
| GO:0044089 | positive regulation of cellular component biogenesis | P | 0.006429 | 8 | over |
| GO:0017022 | myosin binding | F | 0.006983 | 12 | over |
| GO:0046930 | pore complex | C | 0.007541 | 17 | over |
| GO:0031090 | organelle membrane | C | 0.007541 | 68 | under |
| GO:0016459 | myosin complex | C | 0.007541 | 14 | over |
| GO:0045132 | meiotic chromosome segregation | P | 0.007541 | 7 | over |
| GO:0045445 | myoblast differentiation | P | 0.009075 | 12 | over |
| GO:0016657 | oxidoreductase activity, acting on NADH or NADPH, nitrogenous group as acceptor | F | 0.010116 | 3 | over |
| GO:0003920 | GMP reductase activity | F | 0.010116 | 3 | over |
| GO:0030692 | Noc4p-Nop14p complex | C | 0.010116 | 3 | over |
| GO:0048769 | sarcomerogenesis | P | 0.011275 | 6 | over |
| GO:0000070 | mitotic sister chromatid segregation | P | 0.011529 | 11 | over |
| GO:0051096 | positive regulation of helicase activity | P | 0.011897 | 4 | over |
| GO:0000226 | microtubule cytoskeleton organization | P | 0.012123 | 36 | over |
| GO:0003007 | heart morphogenesis | P | 0.012801 | 30 | over |
| GO:0000796 | condensin complex | C | 0.012876 | 5 | over |
| GO:0032040 | small-subunit processome | C | 0.012876 | 5 | over |
| GO:0000479 | endonucleolytic cleavage of tricistronic rRNA transcript (SSU-rRNA, 5.8S rRNA, LSU-rRNA) | P | 0.012876 | 5 | over |
| GO:0000478 | endonucleolytic cleavage involved in rRNA processing | P | 0.012876 | 5 | over |
| GO:0000469 | cleavage involved in rRNA processing | P | 0.012876 | 5 | over |
| GO:0015629 | actin cytoskeleton | C | 0.01376 | 45 | over |
| GO:0048646 | anatomical structure formation involved in morphogenesis | P | 0.014465 | 128 | over |
| GO:0071670 | smooth muscle cell chemotaxis | P | 0.014832 | 4 | over |
| GO:0007062 | sister chromatid cohesion | P | 0.015028 | 9 | over |
| GO:0001533 | cornified envelope | C | 0.01528 | 6 | over |
| GO:0032991 | macromolecular complex | C | 0.01528 | 246 | over |
| GO:0043234 | protein complex | C | 0.016561 | 211 | over |
| GO:0044430 | cytoskeletal part | C | 0.01675 | 91 | over |
| GO:0030689 | Noc complex | C | 0.01675 | 3 | over |
| GO:0071840 | cellular component organization or biogenesis | P | 0.01711 | 263 | over |
| GO:0035994 | response to muscle stretch | P | 0.017395 | 5 | over |
| GO:0044428 | nuclear part | C | 0.018189 | 196 | over |
| GO:0032405 | MutLalpha complex binding | F | 0.018189 | 4 | over |
| GO:0032135 | DNA insertion or deletion binding | F | 0.018189 | 4 | over |
| GO:0071897 | DNA biosynthetic process | P | 0.018189 | 8 | over |
| GO:0016571 | histone methylation | P | 0.018189 | 13 | over |
| GO:0055010 | ventricular cardiac muscle tissue morphogenesis | P | 0.018189 | 12 | over |
| GO:0035019 | somatic stem cell maintenance | P | 0.019612 | 9 | over |
| GO:0010564 | regulation of cell cycle process | P | 0.019709 | 45 | over |
| GO:0008200 | ion channel inhibitor activity | F | 0.020332 | 6 | over |
| GO:0007051 | spindle organization | P | 0.020527 | 18 | over |
| GO:0003779 | actin binding | F | 0.021132 | 38 | over |
| GO:0003229 | ventricular cardiac muscle tissue development | P | 0.021492 | 12 | over |
| GO:0005863 | striated muscle myosin thick filament | C | 0.022191 | 4 | over |
| GO:0006200 | ATP catabolic process | P | 0.022191 | 25 | over |
| GO:0000466 | maturation of 5.8S rRNA from tricistronic rRNA transcript (SSU-rRNA, 5.8S rRNA, LSU-rRNA) | P | 0.02581 | 5 | over |
| GO:0005721 | centromeric heterochromatin | C | 0.02581 | 5 | over |
| GO:0000462 | maturation of SSU-rRNA from tricistronic rRNA transcript (SSU-rRNA, 5.8S rRNA, LSU-rRNA) | P | 0.02581 | 5 | over |
| GO:0007267 | cell-cell signaling | P | 0.02601 | 20 | under |
| GO:0048741 | skeletal muscle fiber development | P | 0.027079 | 18 | over |
| GO:0032982 | myosin filament | C | 0.030051 | 7 | over |
| GO:0005768 | endosome | C | 0.030995 | 9 | under |
| GO:0048468 | cell development | P | 0.033736 | 117 | over |
| GO:0007076 | mitotic chromosome condensation | P | 0.033871 | 6 | over |
| GO:0048856 | anatomical structure development | P | 0.033878 | 261 | over |
| GO:0048739 | cardiac muscle fiber development | P | 0.037095 | 6 | over |
| GO:0070626 | (S)-2-(5-amino-1-(5-phospho-D-ribosyl)imidazole-4-carboxamido)succinate AMP-lyase (fumarate-forming) activity | F | 0.037095 | 3 | over |
| GO:0004018 | N6-(1,2-dicarboxyethyl)AMP AMP-lyase (fumarate-forming) activity | F | 0.037095 | 3 | over |
| GO:0031260 | pseudopodium membrane | C | 0.037364 | 2 | over |
| GO:0048731 | system development | P | 0.037364 | 230 | over |
| GO:0030686 | 90S preribosome | C | 0.037682 | 5 | over |
| GO:0031023 | microtubule organizing center organization | P | 0.037682 | 12 | over |
| GO:0007098 | centrosome cycle | P | 0.037865 | 9 | over |
| GO:0044431 | Golgi apparatus part | C | 0.039215 | 10 | under |
| GO:0005884 | actin filament | C | 0.0407 | 15 | over |
| GO:0008016 | regulation of heart contraction | P | 0.040885 | 18 | over |
| GO:0030490 | maturation of SSU-rRNA | P | 0.042061 | 5 | over |
| GO:0031672 | A band | C | 0.04291 | 10 | over |
| GO:0016248 | channel inhibitor activity | F | 0.04291 | 6 | over |
| GO:0007052 | mitotic spindle organization | P | 0.045086 | 12 | over |
| GO:0022607 | cellular component assembly | P | 0.045086 | 119 | over |
| GO:0006189 | 'de novo' IMP biosynthetic process | P | 0.046067 | 4 | over |
| GO:0032404 | mismatch repair complex binding | F | 0.046067 | 4 | over |
| GO:0010831 | positive regulation of myotube differentiation | P | 0.046067 | 4 | over |
| GO:0051095 | regulation of helicase activity | P | 0.046067 | 4 | over |
| GO:0007268 | synaptic transmission | P | 0.046862 | 11 | under |
| GO:0051297 | centrosome organization | P | 0.046982 | 11 | over |
| GO:0012506 | vesicle membrane | C | 0.046982 | 4 | under |
| GO:0030261 | chromosome condensation | P | 0.046982 | 8 | over |
| GO:0032357 | oxidized purine DNA binding | F | 0.046982 | 3 | over |
| GO:0032301 | MutSalpha complex | C | 0.046982 | 3 | over |
| GO:0032143 | single thymine insertion binding | F | 0.046982 | 3 | over |
| GO:0032142 | single guanine insertion binding | F | 0.046982 | 3 | over |
| GO:0032139 | dinucleotide insertion or deletion binding | F | 0.046982 | 3 | over |
| GO:0032356 | oxidized DNA binding | F | 0.046982 | 3 | over |

Table C. Atlantic salmon microarray probes identified as differentially transcribed by juveniles reared in enriched and unenriched hatchery environments and that corresponded to the GO terms “heart development” and “cell cycle” in a Blast2GO gene enrichment analysis. Differentially transcribed genes were identified using a FDR of 5% in siggenes. The fold-change in gene transcription levels found in enriched/unenriched hatchery environments are indicated and reported for all families and for each of the three examined families individually.

| GO Term | Probe ID | Best BLASTx hit* | Fold-change  (all families) | Fold-change  (X22) | Fold-change  (X11) | Fold-change  (X35) |
| --- | --- | --- | --- | --- | --- | --- |
| Heart development (GO:0007507) | C045R110 | Purpurin precursor | 1.62 | 1.77 | 1.74 | 1.44 |
|  | C080R142 | Guanine nucleotide-binding protein alpha-14 subunit | 1.38 | 1.65 | 1.28 | 1.23 |
|  | C184R075 | Protein FAM3C precursor | 1.30 | 1.31 | 1.31 | 1.27 |
|  | C101R057 | Delta-like protein B precursor | 0.83 | 0.81 | 0.87 | 0.80 |
|  | C259R081 | Proliferating cell nuclear antigen | 0.81 | 0.81 | 0.79 | 0.82 |
|  | C122R068 | Thiamine-triphosphatase | 0.80 | 0.75 | 0.76 | 0.90 |
|  | C045R038 | Beta-parvin | 0.79 | 0.75 | 0.78 | 0.84 |
|  | C183R051 | Cysteine-rich protein 1 | 0.78 | 0.67 | 0.93 | 0.77 |
|  | C184R019 | FK506-binding protein 1B | 0.78 | 0.92 | 0.61 | 0.86 |
|  | C266R068 | Malcavernin | 0.77 | 0.76 | 0.53 | 1.06 |
|  | C155R100 | Noggin-3 precursor | 0.77 | 0.80 | 0.74 | 0.79 |
|  | C149R123 | Nexilin | 0.76 | 0.68 | 0.88 | 0.75 |
|  | C056R089 | Troponin C, slow skeletal and cardiac muscles | 0.76 | 0.91 | 0.74 | 0.66 |
|  | C224R160 | Intraflagellar transport protein 88 homolog | 0.76 | 0.87 | 0.67 | 0.77 |
|  | C120R070 | Endothelin-converting enzyme 2 | 0.76 | 0.84 | 0.81 | 0.61 |
|  | C206R024 | Dihydrofolate reductase | 0.75 | 0.84 | 0.83 | 0.61 |
|  | C083R044 | LIM domain-binding protein 3 | 0.75 | 0.68 | 0.74 | 0.82 |
|  | C181R067 | NOGO isoform A2 | 0.74 | 0.63 | 0.73 | 0.92 |
|  | C142R098 | LIM domain-binding protein 3 | 0.74 | 0.69 | 0.62 | 0.90 |
|  | C074R053 | Troponin C, slow skeletal and cardiac muscles | 0.74 | 0.98 | 0.61 | 0.69 |
|  | C125R071 | SET and MYND domain-containing protein 1 | 0.73 | 0.64 | 0.69 | 0.85 |
|  | C255R040 | LIM domain-binding protein 3 | 0.73 | 0.66 | 0.87 | 0.69 |
|  | C115R138 | Myosin-binding protein C, cardiac-type | 0.73 | 0.72 | 0.75 | 0.71 |
|  | C129R146 | Catenin beta-1 | 0.73 | 0.75 | 0.72 | 0.72 |
|  | C021R009 | LIM domain-binding protein 3 | 0.73 | 0.60 | 0.66 | 0.94 |
|  | C244R012 | T-box transcription factor TBX1 | 0.72 | 0.78 | 0.46 | 0.98 |
|  | C238R116 | Ankyrin repeat domain-containing protein 1 | 0.72 | 0.85 | 0.63 | 0.68 |
|  | C085R011 | RuvB-like 2 | 0.72 | 0.38 | 0.90 | 0.88 |
|  | C204R020 | Nipped-B-like protein | 0.72 | 0.74 | 0.62 | 0.78 |
|  | C118R080 | Protein polybromo-1 | 0.70 | 0.73 | 0.57 | 0.81 |
|  | C138R160 | LIM domain-binding protein 3 | 0.70 | 0.78 | 0.58 | 0.75 |
|  | C203R016 | N-acetyltransferase ESCO1 | 0.70 | 0.61 | 0.89 | 0.64 |
|  | C020R060 | Nck-associated protein 1-like | 0.69 | 0.83 | 0.42 | 0.95 |
|  | C187R163 | Cell division control protein 2 homolog | 0.68 | 0.75 | 0.69 | 0.61 |
|  | C249R024 | LIM domain-binding protein 3 | 0.68 | 0.71 | 0.80 | 0.53 |
|  | C147R057 | Actin, alpha cardiac muscle 1 | 0.67 | 0.70 | 0.52 | 0.82 |
|  | C227R008 | Beta-type platelet-derived growth factor receptor precursor | 0.66 | 0.45 | 0.81 | 0.81 |
|  | C175R023 | Fox-1 homolog-like protein 1 | 0.66 | 0.62 | 0.55 | 0.84 |
|  | C012R013 | Actin, alpha cardiac muscle 1 | 0.66 | 0.79 | 0.45 | 0.85 |
|  | C160R061 | UNC45 homolog B | 0.66 | 0.56 | 0.74 | 0.68 |
|  | C261R094 | Beta-type platelet-derived growth factor receptor precursor | 0.65 | 0.59 | 0.58 | 0.81 |
|  | C256R034 | LIM domain-binding protein 3 | 0.64 | 0.81 | 0.45 | 0.68 |
|  | C173R066 | PTRF/SDPR family protein | 0.64 | 0.70 | 0.53 | 0.68 |
|  | C009R091 | Fibulin-1 precursor | 0.62 | 0.73 | 0.43 | 0.83 |
|  | C160R062 | Mothers against decapentaplegic homolog 5 | 0.62 | 0.85 | 0.33 | 0.94 |
|  | C065R162 | SET and MYND domain-containing protein 1 | 0.62 | 0.51 | 0.68 | 0.68 |
|  | C153R037 | Actin, alpha cardiac muscle 1 | 0.61 | 0.52 | 0.54 | 0.77 |
|  | C146R110 | Myosin-binding protein C, cardiac-type | 0.61 | 0.49 | 0.62 | 0.69 |
|  | C145R100 | Myosin-6 | 0.61 | 0.64 | 0.46 | 0.74 |
|  | C057R111 | Cysteine and glycine-rich protein 3 | 0.57 | 0.62 | 0.44 | 0.65 |
|  | C240R162 | Actin, larval muscle | 0.56 | 0.74 | 0.51 | 0.51 |
|  | C252R157 | Troponin C, slow skeletal and cardiac muscles | 0.56 | 0.77 | 0.42 | 0.59 |
|  | C013R158 | SET and MYND domain-containing protein 1 | 0.56 | 0.53 | 0.55 | 0.62 |
|  | C098R103 | Putative heparin-binding growth factor 1 | 0.56 | 0.66 | 0.42 | 0.65 |
|  | C073R023 | Cysteine and glycine-rich protein 3 | 0.56 | 0.51 | 0.52 | 0.65 |
|  | C062R056 | Fox-1 homolog-like protein 1 | 0.53 | 0.48 | 0.44 | 0.78 |
|  | C260R055 | Myosin-binding protein C, cardiac-type | 0.53 | 0.62 | 0.39 | 0.63 |
|  | C266R127 | SET and MYND domain-containing protein 1 | 0.52 | 0.41 | 0.67 | 0.54 |
|  | C025R008 | Tropomyosin alpha-4 chain | 0.51 | 0.49 | 0.48 | 0.58 |
|  | C244R017 | Cysteine and glycine-rich protein 3 | 0.48 | 0.46 | 0.43 | 0.56 |
|  | C233R135 | Myosin-2 | 0.45 | 0.68 | 0.33 | 0.46 |
|  | C251R080 | Myosin regulatory light chain 2, atrial isoform | 0.10 | 0.12 | 0.06 | 0.13 |
|  | C086R103 | Myosin light chain 4 | 0.06 | 0.08 | 0.04 | 0.08 |
| Cell cycle (GO:0007049) | C211R161 | Metalloproteinase inhibitor 2 precursor | 2.05 | 2.51 | 2.76 | 1.32 |
|  | C076R102 | Cyclin-G2 | 1.60 | 1.43 | 1.82 | 1.56 |
|  | C128R109 | Dynein light chain 2, cytoplasmic | 1.54 | 1.41 | 1.91 | 1.29 |
|  | C023R126 | 5'-AMP-activated protein kinase subunit beta-1 | 1.48 | 1.39 | 1.69 | 1.36 |
|  | C196R097 | Protein BCCIP homolog | 1.47 | 1.50 | 1.61 | 1.32 |
|  | C141R027 | Transcription factor Sox-2 | 1.46 | 1.36 | 1.70 | 1.38 |
|  | C154R165 | Cyclin-dependent kinase 4 inhibitor B | 1.35 | 1.29 | 1.56 | 1.23 |
|  | C080R079 | Cyclin-dependent kinase 4 inhibitor B | 1.32 | 1.15 | 1.55 | 1.29 |
|  | C253R055 | 26S protease regulatory subunit 8 | 0.85 | 0.89 | 0.76 | 0.91 |
|  | C146R131 | Small nuclear ribonucleoprotein Sm D3 | 0.84 | 0.89 | 0.79 | 0.86 |
|  | C161R096 | Nuclear pore complex protein Nup160 | 0.84 | 0.93 | 0.74 | 0.87 |
|  | C075R094 | Multifunctional protein ADE2 | 0.84 | 0.89 | 0.80 | 0.84 |
|  | C107R039 | 39S ribosomal protein L41, mitochondrial precursor | 0.83 | 0.87 | 0.74 | 0.90 |
|  | C237R086 | Guanine nucleotide-binding protein-like 3 | 0.82 | 0.85 | 0.82 | 0.80 |
|  | C148R076 | Exportin-1 | 0.82 | 0.87 | 0.79 | 0.80 |
|  | C182R067 | Transcriptional repressor CTCF | 0.81 | 0.90 | 0.74 | 0.81 |
|  | C086R088 | Acidic leucine-rich nuclear phosphoprotein 32 family member A | 0.81 | 0.88 | 0.63 | 0.90 |
|  | C259R081 | Proliferating cell nuclear antigen | 0.81 | 0.81 | 0.79 | 0.82 |
|  | C122R068 | Thiamine-triphosphatase | 0.80 | 0.75 | 0.76 | 0.90 |
|  | C080R163 | Importin subunit alpha-2 | 0.80 | 1.02 | 0.70 | 0.69 |
|  | C244R043 | Kinesin-like protein KIF23 | 0.80 | 0.80 | 0.79 | 0.81 |
|  | C251R045 | Cyclin-dependent kinases regulatory subunit 2 | 0.80 | 0.78 | 0.81 | 0.81 |
|  | C097R015 | DNA replication licensing factor MCM3 | 0.80 | 0.69 | 1.01 | 0.73 |
|  | C177R153 | Structural maintenance of chromosomes protein 2 | 0.79 | 0.79 | 0.86 | 0.74 |
|  | C234R130 | DNA mismatch repair protein Msh6 | 0.79 | 0.81 | 0.88 | 0.71 |
|  | C078R131 | Proteasome subunit beta type-7 precursor | 0.79 | 1.02 | 0.68 | 0.74 |
|  | C194R076 | Targeting protein for Xklp2 | 0.79 | 0.91 | 0.70 | 0.78 |
|  | C210R163 | E3 SUMO-protein ligase RanBP2 | 0.79 | 0.90 | 0.78 | 0.71 |
|  | C236R099 | Protein HGV2 | 0.79 | 0.81 | 0.88 | 0.69 |
|  | C164R145 | Enhancer of zeste homolog 2 | 0.79 | 1.01 | 0.60 | 0.80 |
|  | C172R088 | Eukaryotic translation initiation factor 4E | 0.78 | 0.95 | 0.70 | 0.73 |
|  | C083R023 | Structural maintenance of chromosomes protein 2 | 0.78 | 0.73 | 0.92 | 0.68 |
|  | C190R020 | Ribonucleoside-diphosphate reductase large subunit | 0.78 | 0.62 | 0.97 | 0.80 |
|  | C200R114 | Transitional endoplasmic reticulum ATPase | 0.78 | 0.78 | 0.59 | 0.97 |
|  | C021R165 | Kinesin-related motor protein Eg5 2 | 0.78 | 0.94 | 0.70 | 0.72 |
|  | C016R017 | Filamin-C | 0.77 | 0.72 | 0.72 | 0.90 |
|  | C013R156 | Leukocyte common antigen precursor | 0.77 | 0.68 | 0.80 | 0.82 |
|  | C070R024 | Ran GTPase-activating protein 1 | 0.77 | 0.92 | 0.69 | 0.75 |
|  | C063R090 | Centromere protein U | 0.77 | 1.05 | 0.64 | 0.60 |
|  | C154R066 | Nuclear pore complex protein Nup107 | 0.77 | 0.81 | 0.72 | 0.78 |
|  | C014R147 | Suppressor of G2 allele of SKP1 homolog | 0.77 | 0.62 | 0.75 | 0.98 |
|  | C200R045 | DNA polymerase subunit alpha B | 0.76 | 0.66 | 0.93 | 0.72 |
|  | C141R091 | Kinesin-like protein KIF2C | 0.76 | 0.66 | 0.85 | 0.77 |
|  | C248R102 | 26S proteasome non-ATPase regulatory subunit 2 | 0.76 | 0.76 | 0.68 | 0.84 |
|  | C194R011 | Nuclear distribution protein nudE homolog 1 | 0.76 | 0.72 | 0.67 | 0.89 |
|  | C206R024 | Dihydrofolate reductase | 0.75 | 0.84 | 0.83 | 0.61 |
|  | C159R044 | Arachidonate 12-lipoxygenase, 12R type | 0.75 | 0.67 | 0.72 | 0.87 |
|  | C243R144 | Protein FAM29A | 0.75 | 0.75 | 0.69 | 0.81 |
|  | C010R028 | DNA topoisomerase 2-alpha | 0.75 | 0.77 | 0.78 | 0.69 |
|  | C079R070 | DNA topoisomerase 2-binding protein 1 | 0.75 | 1.01 | 0.58 | 0.74 |
|  | C254R073 | Allograft inflammatory factor 1 | 0.75 | 0.63 | 0.67 | 0.90 |
|  | C085R023 | cAMP-regulated phosphoprotein 19 | 0.75 | 0.86 | 0.59 | 0.78 |
|  | C209R138 | Enhancer of rudimentary homolog | 0.75 | 0.89 | 0.61 | 0.78 |
|  | C063R091 | Homologous-pairing protein 2 homolog | 0.74 | 0.85 | 0.52 | 0.89 |
|  | C003R047 | Inner centromere protein | 0.74 | 0.73 | 0.75 | 0.75 |
|  | C006R050 | Inner centromere protein | 0.74 | 0.64 | 0.82 | 0.79 |
|  | C055R090 | RNA-binding protein 38 | 0.74 | 0.79 | 0.60 | 0.89 |
|  | C252R074 | Targeting protein for Xklp2 | 0.74 | 0.83 | 0.72 | 0.66 |
|  | C228R095 | Condensin complex subunit 1 | 0.73 | 0.69 | 0.87 | 0.66 |
|  | C255R047 | Origin recognition complex subunit 5 | 0.73 | 0.82 | 0.71 | 0.67 |
|  | C168R019 | Transforming growth factor-beta-inducible early growth response protein 3 | 0.73 | 0.73 | 0.59 | 0.87 |
|  | C119R005 | Structural maintenance of chromosomes protein 4 | 0.73 | 0.65 | 0.75 | 0.79 |
|  | C201R116 | DNA repair protein RAD51 homolog B | 0.73 | 0.85 | 0.76 | 0.58 |
|  | C245R120 | CLIP-associating protein 2 | 0.73 | 0.90 | 0.44 | 0.91 |
|  | C166R011 | Ran GTPase-activating protein 1 | 0.73 | 0.66 | 0.67 | 0.88 |
|  | C129R146 | Catenin beta-1 | 0.73 | 0.75 | 0.72 | 0.72 |
|  | C202R057 | Nuclear autoantigenic sperm protein | 0.73 | 0.82 | 0.68 | 0.70 |
|  | C248R111 | Dual specificity protein kinase Ttk | 0.73 | 0.67 | 0.70 | 0.83 |
|  | C162R130 | Protein regulator of cytokinesis 1 | 0.72 | 0.92 | 0.67 | 0.60 |
|  | C233R161 | Kinesin-related motor protein Eg5 2 | 0.72 | 0.78 | 0.78 | 0.62 |
|  | C169R048 | Heat shock cognate 71 kDa protein | 0.72 | 0.56 | 0.71 | 0.90 |
|  | C177R128 | Centromere protein I | 0.72 | 0.82 | 0.94 | 0.47 |
|  | C204R020 | Nipped-B-like protein | 0.72 | 0.74 | 0.62 | 0.78 |
|  | C260R062 | Mitotic checkpoint serine/threonine-protein kinase BUB1 | 0.72 | 0.84 | 0.61 | 0.70 |
|  | C062R074 | M-phase phosphoprotein 1 | 0.71 | 0.61 | 0.88 | 0.70 |
|  | C021R017 | Cytoskeleton-associated protein 2 | 0.71 | 0.79 | 0.66 | 0.71 |
|  | C224R013 | Forkhead box protein N3 | 0.71 | 0.72 | 0.54 | 0.88 |
|  | C026R169 | Fanconi anemia group D2 protein | 0.71 | 0.83 | 0.67 | 0.67 |
|  | C148R122 | Protein ECT2 | 0.71 | 0.81 | 0.77 | 0.57 |
|  | C148R165 | Mitotic spindle assembly checkpoint protein MAD2A | 0.71 | 0.97 | 0.75 | 0.46 |
|  | C118R080 | Protein polybromo-1 | 0.70 | 0.73 | 0.57 | 0.81 |
|  | C241R063 | Cysteine and histidine-rich domain-containing protein 1 | 0.70 | 0.55 | 0.78 | 0.79 |
|  | C142R032 | Cysteine and histidine-rich domain-containing protein 1 | 0.70 | 0.57 | 0.72 | 0.80 |
|  | C213R109 | Kinesin-like protein KIF23 | 0.70 | 0.61 | 0.91 | 0.60 |
|  | C203R016 | N-acetyltransferase ESCO1 | 0.70 | 0.61 | 0.89 | 0.64 |
|  | C205R023 | DNA mismatch repair protein MSH2 | 0.69 | 0.56 | 0.98 | 0.55 |
|  | C251R131 | Microtubule-associated serine/threonine-protein kinase-like | 0.69 | 0.73 | 0.55 | 0.79 |
|  | C170R129 | ATP-dependent DNA helicase Q4 | 0.69 | 0.81 | 0.51 | 0.75 |
|  | C187R163 | Cell division control protein 2 homolog | 0.68 | 0.75 | 0.69 | 0.61 |
|  | C233R016 | PCNA-associated factor | 0.68 | 0.66 | 0.73 | 0.65 |
|  | C099R026 | Esophageal cancer-related gene 4 protein precursor | 0.68 | 0.59 | 0.67 | 0.80 |
|  | C229R120 | Centrosomal protein of 135 kDa | 0.68 | 0.71 | 0.37 | 0.98 |
|  | C020R126 | Dual specificity protein kinase Ttk | 0.68 | 0.76 | 0.57 | 0.74 |
|  | C233R147 | Myb-binding protein 1A-like protein | 0.67 | 0.72 | 0.62 | 0.68 |
|  | C161R081 | Multidrug resistance protein 1 | 0.67 | 0.79 | 0.49 | 0.74 |
|  | C063R169 | DNA mismatch repair protein Msh2 | 0.67 | 0.79 | 0.56 | 0.68 |
|  | C059R123 | Sperm-associated antigen 5 | 0.67 | 0.82 | 0.69 | 0.53 |
|  | C080R082 | G2/mitotic-specific cyclin-B1 | 0.67 | 0.62 | 0.85 | 0.55 |
|  | C019R035 | F-box/WD repeat-containing protein 5 | 0.67 | 0.63 | 0.54 | 0.84 |
|  | C227R008 | Beta-type platelet-derived growth factor receptor precursor | 0.66 | 0.45 | 0.81 | 0.81 |
|  | C011R044 | Centromere protein M | 0.66 | 0.72 | 0.60 | 0.68 |
|  | C145R103 | Sperm-associated antigen 5 | 0.66 | 0.44 | 0.97 | 0.72 |
|  | C003R061 | Serine/threonine-protein kinase 12 | 0.65 | 0.67 | 0.63 | 0.67 |
|  | C165R018 | Ubiquitin carboxyl-terminal hydrolase 28 | 0.65 | 0.50 | 0.44 | 1.03 |
|  | C261R094 | Beta-type platelet-derived growth factor receptor precursor | 0.65 | 0.59 | 0.58 | 0.81 |
|  | C259R049 | Leucine-rich repeat and WD repeat-containing protein 1 | 0.65 | 0.56 | 0.70 | 0.70 |
|  | C247R129 | Nucleoprotein TPR | 0.64 | 0.53 | 0.59 | 0.78 |
|  | C246R044 | Chromosome transmission fidelity protein 8 | 0.64 | 0.76 | 0.53 | 0.62 |
|  | C165R011 | Abnormal spindle-like microcephaly-associated protein | 0.64 | 0.51 | 0.77 | 0.62 |
|  | C239R104 | M-phase phosphoprotein 1 | 0.63 | 0.69 | 0.57 | 0.65 |
|  | C093R148 | DNA polymerase delta catalytic subunit | 0.63 | 0.65 | 0.62 | 0.63 |
|  | C125R109 | CPEB-associated factor Maskin | 0.63 | 0.66 | 0.69 | 0.53 |
|  | C118R056 | Proto-oncogene tyrosine-protein kinase FER | 0.62 | 0.96 | 0.35 | 0.78 |
|  | C237R008 | Protein CASC5 | 0.61 | 0.56 | 0.49 | 0.82 |
|  | C204R156 | SWI/SNF-related matrix-associated actin-dependent regulator of chromatin subfamily A-like protein 1 | 0.61 | 0.69 | 0.66 | 0.51 |
|  | C169R125 | Nucleoprotein TPR | 0.61 | 0.76 | 0.49 | 0.68 |
|  | C213R107 | Cell division control protein 6 homolog | 0.61 | 0.84 | 0.45 | 0.63 |
|  | C160R142 | Caveolin-2 | 0.54 | 0.61 | 0.40 | 0.63 |
|  | C231R077 | Structural maintenance of chromosomes protein 4 | 0.51 | 0.71 | 0.29 | 0.77 |
|  | C100R025 | Telomere-associated protein RIF1 | 0.50 | 0.53 | 0.47 | 0.52 |
|  | C132R101 | Replication factor C subunit 3 | 0.49 | 1.38 | 0.15 | 0.62 |
|  | C113R135 | Regulator of chromosome condensation | 0.44 | 0.60 | 0.23 | 0.75 |

^*^Significantly differentially transcribed genes were annotated in Blast2GO using the BLASTx algorithm and the best BLASTx hit (E-value < 10^-6^) is presented.

Table D. Atlantic salmon microarray probes showing differing patterns of gene transcription in juvenile salmon reared in unenriched and enriched environments. Gene transcription patterns were examined using the cGRASP-designed 4×44K salmon gene microarray and the analysis conducted separately for each of three families, X11, X22, and X35, using SAM. Results are shown for family X22 and X11 at a FDR of 5% and for family X35 at a FDR of 8%. The fold-change in gene transcription levels found in enriched compared to unenriched habitats are indicated.

| Family | Probe ID | Best Blastx hit* | Fold-change  (enriched/unenriched) |
| --- | --- | --- | --- |
| X22 | C211R161^§^ | metalloproteinase inhibitor 2 precursor | 2.51 |
|  | C200R070 | eh domain-binding protein 1-like protein 1 | 0.62 |
|  | C028R014 | c3 and pzp-like alpha-2-macroglobulin domain-containing protein 8-like | 0.62 |
|  | C246R012 | serine protease htra1 precursor | 0.61 |
|  | C065R063 | UNKNOWN | 0.60 |
|  | C109R011 | outer dense fiber protein 3-like protein 2-like | 0.59 |
|  | C102R006 | cklf-like marvel transmembrane domain-containing protein 7 | 0.58 |
|  | C138R017 | b-cell receptor-associated protein 29 | 0.56 |
|  | C010R015^§^ | cd276 antigen | 0.52 |
|  | C233R116 | transferrin precursor | 0.49 |
|  | C149R037 | shugoshin-like 1 | 0.45 |
|  | C148R007 | rho-related gtp-binding protein | 0.43 |
|  | C118R012 | probable d-lactate mitochondrial-like | 0.42 |
|  | C085R011 | ruvb-like 2 | 0.38 |
|  | C194R001 | transcription termination factor 1-like | 0.32 |
|  | C260R032^§^ | t-cell surface glycoprotein cd5-like | 0.22 |
|  | C173R009 | atp-dependent rna helicase ddx1 | 0.15 |
| X11 | C258R138 | UNKNOWN | 6.88 |
|  | C076R113 | protein-glutamine gamma-glutamyltransferase 5-like | 5.80 |
|  | C204R103 | cytochrome p450 2m1 | 5.37 |
|  | C067R138 | protein-glutamine gamma-glutamyltransferase e-like | 4.21 |
|  | C168R047 | chordin-like protein 2-like | 4.08 |
|  | C205R145 | claudin-10-like | 3.92 |
|  | C178R155 | type i cytoskeletal 13-like | 3.90 |
|  | C243R111 | mhc class i antigen | 3.78 |
|  | C108R097 | motile sperm domain-containing protein 2 | 3.76 |
|  | C177R022 | ras-like protein family member 11b | 3.75 |
|  | C087R147 | leucine-rich glioma-inactivated protein 1 precursor | 3.59 |
|  | C249R164 | UNKNOWN | 3.56 |
|  | C185R152 | nucleophosmin | 3.48 |
|  | C262R042 | serine threonine protein kinase | 3.45 |
|  | C237R040^‡^ | PREDICTED: hypothetical protein LOC324610 | 3.40 |
|  | C182R007 | tripartite motif-containing protein 35-like | 3.35 |
|  | C079R001 | UNKNOWN | 3.33 |
|  | C169R151 | homeobox protein dlx-1 | 3.28 |
|  | C091R002 | tax1-binding protein 1 homolog b-like | 3.26 |
|  | C215R090 | UNKNOWN | 3.19 |
|  | C071R117 | ras-related protein rab-7a | 3.14 |
|  | C069R141 | protein atp1b4-like | 3.11 |
|  | C039R004 | secreted frizzled-related protein 1 precursor | 3.09 |
|  | C154R016 | follistatin-related protein 3 | 3.07 |
|  | C198R002 | sodium potassium-transporting atpase subunit beta-3-like | 3.05 |
|  | C005R155 | fatty acid binding protein h6-isoform | 3.05 |
|  | C132R081 | ring finger protein 13 | 3.00 |
|  | C148R124 | protein c-ets-2 | 2.99 |
|  | C081R005 | e3 ubiquitin-protein ligase rfwd2 | 2.98 |
|  | C085R046 | protein tyrosine phosphatase type iva 1 | 2.96 |
|  | C170R024 | calcium-binding protein 39-like | 2.96 |
|  | C060R004 | cation-independent mannose-6-phosphate receptor | 2.95 |
|  | C082R055 | cytochrome c oxidase subunit 1 | 2.92 |
|  | C035R081 | transmembrane emp24 domain-containing protein 4 | 2.91 |
|  | C179R004 | transcription factor hes-5-like | 2.91 |
|  | C266R141 | elongation of very long chain fatty acids protein 4-like | 2.86 |
|  | C026R035 | protein phosphatase 1 regulatory subunit 14b | 2.82 |
|  | C012R016 | ankyrin repeat domain-containing protein 22 | 2.80 |
|  | C185R057 | heme oxygenase | 2.80 |
|  | C034R024 | v-type proton atpase 21 kda proteolipid subunit-like | 2.77 |
|  | C211R161^§^ | metalloproteinase inhibitor 2 precursor | 2.76 |
|  | C106R002 | seizure protein 6 homolog | 2.76 |
|  | C071R051 | heme oxygenase | 2.74 |
|  | C154R021 | hypothetical protein SGRA_3282 | 2.73 |
|  | C184R005 | msx1 partial | 2.73 |
|  | C203R098 | septin 5 | 2.71 |
|  | C183R003 | carbohydrate sulfotransferase 12-like | 2.70 |
|  | C181R010 | serine threonine-protein kinase pim-3 | 2.69 |
|  | C051R009 | protein fam181a-like | 2.69 |
|  | C210R133 | lysosomal thioesterase ppt2-a-like | 2.68 |
|  | C238R158 | transmembrane protein 127 | 2.67 |
|  | C120R101 | UNKNOWN | 2.66 |
|  | C235R030 | protein disulfide-isomerase a6 | 2.65 |
|  | C202R136 | collagen type iv alpha-3-binding | 2.65 |
|  | C154R002 | cofilin-2 | 2.64 |
|  | C211R164 | mhc class i | 2.64 |
|  | C175R004 | lymphoid-specific helicase | 2.63 |
|  | C036R160 | atpase h+ transporting v1 subunit g isoform 1 | 2.61 |
|  | C182R144 | pyridoxal kinase-like | 2.61 |
|  | C265R026 | elongation factor 1-gamma | 2.60 |
|  | C215R100 | transmembrane protein 106b | 2.60 |
|  | C087R027 | lim and sh3 domain protein 1 | 2.59 |
|  | C196R032 | protein fam76b | 2.58 |
|  | C261R019 | matrin 3-like | 2.58 |
|  | C257R146 | embigin precursor | 2.57 |
|  | C124R005 | phospholipid transfer protein precursor | 2.55 |
|  | C145R007 | UNKNOWN | 2.54 |
|  | C251R005 | dna chr wayne state university expressed | 2.54 |
|  | C084R127 | forkhead box protein b1 | 2.54 |
|  | C194R139 | heterogeneous nuclear ribonucleoprotein a1 | 2.54 |
|  | C048R030 | stomatin-like protein 2 | 2.54 |
|  | C022R025 | udp-n-acetylhexosamine pyrophosphorylase-like | 2.53 |
|  | C070R087 | v-type proton atpase subunit c 1-a-like | 2.53 |
|  | C221R033 | ras-related protein rab-3a | 2.53 |
|  | C262R103 | transcription factor jun-d | 2.52 |
|  | C037R004 | chromobox protein homolog 8-like | 2.52 |
|  | C069R069 | ras-related protein ral-b | 2.51 |
|  | C216R003 | ubiquitin carboxyl-terminal hydrolase 12 | 2.51 |
|  | C201R046 | erlin-2 precursor | 2.50 |
|  | C266R133 | wd repeat and socs box-containing protein 2 | 2.48 |
|  | C217R035 | ras-related and estrogen-regulated growth inhibitor-like | 2.48 |
|  | C250R164 | von willebrand factor type egf and pentraxin domain-containing protein 1-like | 2.47 |
|  | C044R019 | catenin beta-1 | 2.45 |
|  | C250R149 | wd repeat and socs box-containing protein 2 | 2.45 |
|  | C032R163 | casein kinase i isoform alpha | 2.45 |
|  | C053R002 | ammonium transporter rh type c 1-like | 2.44 |
|  | C125R006 | protein dispatched homolog 2 | 2.44 |
|  | C134R083 | synaptosomal-associated protein 25 | 2.44 |
|  | C120R054 | ras-related protein rab-10 | 2.44 |
|  | C263R039 | neuroplastin precursor | 2.43 |
|  | C255R093 | transgelin-3 | 2.43 |
|  | C109R004 | ap-1 complex subunit sigma-3 | 2.43 |
|  | C138R082 | cerebellin-1-like | 2.43 |
|  | C048R170 | homeobox protein orthopedia b-like isoform 2 | 2.42 |
|  | C247R004 | upf0488 protein c8orf33 homolog | 2.42 |
|  | C225R166 | zinc finger protein zpr1 | 2.42 |
|  | C066R112 | glutamate receptor 2 isoform x1 | 2.41 |
|  | C237R002 | heterogeneous nuclear ribonucleoprotein u-like protein 1 | 2.41 |
|  | C177R157 | tyrosine-protein kinase frk | 2.40 |
|  | C175R096 | sarcoplasmic endoplasmic reticulum calcium atpase 2 | 2.40 |
|  | C244R146 | protein ndrg2-like | 2.40 |
|  | C118R006 | germ cell-less 1 | 2.40 |
|  | C180R050 | o-phosphoseryl-trna selenium transferase | 2.39 |
|  | C030R163 | neuronal-specific septin-3 | 2.39 |
|  | C172R058 | pituitary homeobox 2 isoform 1 | 2.39 |
|  | C144R105 | v-type proton atpase subunit s1-like | 2.39 |
|  | C190R127 | retinoic acid receptor rxr-beta-a | 2.38 |
|  | C193R041 | mitochondrial import receptor subunit tom40 homolog | 2.38 |
|  | C111R060 | pantothenate kinase 1 | 2.37 |
|  | C112R019 | transcription factor jun-d | 2.37 |
|  | C164R063 | alanyl-trna cytoplasmic | 2.37 |
|  | C184R003 | water dikinase 1 | 2.37 |
|  | C032R027 | brain protein 44 | 2.37 |
|  | C019R080 | zinc finger protein c3h1 type-like 2 | 2.37 |
|  | C147R126 | prodynorphin precursor | 2.37 |
|  | C263R137 | iroquois-class homeodomain protein irx-3 | 2.36 |
|  | C139R059 | ankyrin repeat domain-containing protein 9-like | 2.36 |
|  | C183R086 | n-acylneuraminate cytidylyltransferase | 2.36 |
|  | C159R155 | heterogeneous nuclear ribonucleoprotein r | 2.35 |
|  | C195R164 | UNKNOWN | 2.35 |
|  | C198R063 | ankyrin repeat family a protein 2 | 2.34 |
|  | C088R165 | UNKNOWN | 2.34 |
|  | C051R146 | transcription factor sox-14 | 2.34 |
|  | C169R002 | phosphogluconate dehydrogenase | 2.34 |
|  | C197R026 | grainyhead-like protein 3 homolog | 2.33 |
|  | C024R096 | hypoxanthine-guanine phosphoribosyltransferase | 2.32 |
|  | C223R127 | dead box atp-dependent rna | 2.32 |
|  | C145R138 | UNKNOWN | 2.31 |
|  | C266R046 | trna-splicing ligase homolog | 2.31 |
|  | C104R055 | protein disulfide-isomerase a3-like | 2.31 |
|  | C213R005 | UNKNOWN | 2.31 |
|  | C172R005 | lysozyme c ii precursor | 2.30 |
|  | C041R151 | protein sco1 mitochondrial-like | 2.30 |
|  | C258R048 | UNKNOWN | 2.29 |
|  | C250R007 | u4 tri-snrnp-associated protein 2 isoform 2 | 2.28 |
|  | C217R156 | zinc finger protein 161 homolog | 2.28 |
|  | C248R158 | fxyd domain-containing ion transport regulator 6 | 2.28 |
|  | C039R087 | 5 -3 exoribonuclease 2 | 2.28 |
|  | C210R044 | biglycan precursor | 2.28 |
|  | C084R142 | n-acylglucosamine 2-epimerase-like | 2.27 |
|  | C090R150 | glycoprotein m6a | 2.26 |
|  | C189R078 | dehydrogenase reductase sdr family member 1 | 2.26 |
|  | C205R152 | matrilin-4 | 2.26 |
|  | C142R144 | iroquois-class homeodomain protein irx-3 | 2.25 |
|  | C093R130 | lanosterol 14-alpha demethylase-like | 2.25 |
|  | C018R117 | glycogen liver form | 2.24 |
|  | C124R044 | claudin-4 | 2.24 |
|  | C044R163 | protein disulfide-isomerase precursor | 2.24 |
|  | C170R001 | UNKNOWN | 2.24 |
|  | C026R095 | sin3 histone deacetylase corepressor complex component sds3 | 2.24 |
|  | C232R150 | UNKNOWN | 2.24 |
|  | C150R011 | kelch-like protein 26 | 2.23 |
|  | C206R153 | probable palmitoyltransferase zdhhc12-like | 2.23 |
|  | C040R018 | rho-related gtp-binding | 2.22 |
|  | C051R138 | hypothetical protein MTR_5g050970 | 2.22 |
|  | C198R026 | btb poz domain-containing protein kctd17 | 2.22 |
|  | C081R116 | protein phosphatase 1g | 2.22 |
|  | C075R168 | dual specificity mitogen-activated protein kinase kinase 4-like | 2.21 |
|  | C223R012 | protein atonal homolog 1-like | 2.21 |
|  | C089R062 | hydroxyacylglutathione hydrolase | 2.21 |
|  | C178R004 | UNKNOWN | 2.21 |
|  | C068R154 | pleiotrophic factor-alpha-2 precursor | 2.20 |
|  | C155R133 | gtpase imap family member 7 | 2.20 |
|  | C042R094 | leptin receptor gene-related protein | 2.19 |
|  | C171R108 | 28s ribosomal protein mitochondrial-like | 2.19 |
|  | C048R162 | 72 kda type iv collagenase | 2.19 |
|  | C017R103 | sodium potassium-transporting atpase subunit beta-233 | 2.19 |
|  | C240R004 | claudin-5-like | 2.19 |
|  | C021R089 | eukaryotic translation initiation factor 1b | 2.19 |
|  | C112R086 | UNKNOWN | 2.19 |
|  | C222R006 | tomoregulin-1-like | 2.19 |
|  | C150R003 | calcium calmodulin-dependent protein kinase ii inhibitor 2 | 2.19 |
|  | C053R047 | hig1 domain family member 1a | 2.19 |
|  | C190R032 | sun domain-containing protein 1-like | 2.18 |
|  | C135R099 | luc7-like protein 3 | 2.18 |
|  | C253R111 | sox3 | 2.18 |
|  | C052R119 | 28 kda heat- and acid-stable phosphoprotein | 2.18 |
|  | C256R101 | spermine oxidase-like | 2.18 |
|  | C023R111 | UNKNOWN | 2.17 |
|  | C167R154 | headcase protein homolog | 2.17 |
|  | C039R050 | zinc finger cchc domain-containing protein 4 | 2.17 |
|  | C015R121 | heat shock protein hsp 90-alpha | 2.17 |
|  | C246R112 | protein fam45a | 2.17 |
|  | C142R035 | heme-binding protein 1 | 2.17 |
|  | C244R004 | dna damage-inducible transcript 4-like | 2.16 |
|  | C066R007 | metalloproteinase inhibitor 2 precursor | 2.16 |
|  | C159R075 | amyloid beta a4 | 2.16 |
|  | C195R122 | cd59 glycoprotein precursor | 2.16 |
|  | C079R156 | spindlin-1 | 2.16 |
|  | C202R085 | transcription factor sox-11 | 2.15 |
|  | C187R065 | UNKNOWN | 2.15 |
|  | C117R102 | myelin expression factor 2 | 2.14 |
|  | C051R003 | UNKNOWN | 2.14 |
|  | C220R164 | sphingosine 1-phosphate receptor 1 | 2.14 |
|  | C161R093 | serine threonine-protein phosphatase 2a 65 kda regulatory subunit a beta isoform-like | 2.14 |
|  | C233R121 | UNKNOWN | 2.14 |
|  | C080R151 | zinc finger protein 346-like | 2.14 |
|  | C104R034 | migration and invasion enhancer 1 | 2.13 |
|  | C192R001 | protein mal2 | 2.13 |
|  | C197R056 | low quality protein: ankyrin-3-like | 2.13 |
|  | C101R141 | toll-interacting protein | 2.13 |
|  | C061R124 | ubiquitin carboxyl-terminal hydrolase 46 | 2.13 |
|  | C045R014 | sex comb on midleg-like protein 4-like | 2.13 |
|  | C246R005 | zinc finger protein c3h1 type-like 1 | 2.13 |
|  | C120R004 | PREDICTED: hypothetical protein LOC100699190 | 2.12 |
|  | C244R145 | microtubule-associated protein 1a | 2.12 |
|  | C187R170 | ap-3 complex subunit mu-2 | 2.12 |
|  | C180R005 | translocon-associated protein subunit delta precursor | 2.12 |
|  | C159R071 | fibroblast growth factor 13 | 2.12 |
|  | C188R044 | syndecan-4 | 2.12 |
|  | C213R141 | geranylgeranyl diphosphate synthase 1 | 2.12 |
|  | C147R115 | coatomer subunit gamma-2 | 2.12 |
|  | C018R006 | zinc finger protein 25 | 2.11 |
|  | C175R016 | gdnf family receptor alpha-1-like | 2.11 |
|  | C205R133 | cub and lccl domain containing 2- partial | 2.11 |
|  | C265R147 | protein fam76b | 2.11 |
|  | C105R004 | adipocyte plasma membrane-associated protein | 2.11 |
|  | C249R017 | UNKNOWN | 2.11 |
|  | C056R146 | zinc finger protein 135- partial | 2.11 |
|  | C260R064 | pyridoxal kinase-like | 2.10 |
|  | C175R128 | novel alpha-globin | 2.10 |
|  | C166R069 | uroplakin-1a | 2.10 |
|  | C073R097 | transcription factor jun-b-like | 2.09 |
|  | C035R062 | neurolin-like cell adhesion molecule | 2.09 |
|  | C219R078 | hexosaminidase d | 2.09 |
|  | C058R115 | o-phosphoseryl-trna selenium transferase | 2.09 |
|  | C039R005 | aspartate beta-hydroxylase domain-containing protein 2 | 2.09 |
|  | C143R012 | ef-hand domain-containing family member a1-like | 2.09 |
|  | C095R124 | galectin-8-like | 2.09 |
|  | C069R056 | UNKNOWN | 2.08 |
|  | C131R128 | na k atpase alpha subunit isoform 1c | 2.08 |
|  | C223R103 | diphosphomevalonate decarboxylase | 2.08 |
|  | C248R160 | tetraspanin-7 | 2.08 |
|  | C077R062 | loc100127300 protein | 2.08 |
|  | C254R089 | tropomyosin alpha-3 chain | 2.08 |
|  | C038R003 | ubiquinone biosynthesis protein mitochondrial precursor | 2.07 |
|  | C023R155 | mothers against decapentaplegic homolog 7-like | 2.06 |
|  | C083R075 | programmed cell death protein 4 isoform 1 | 2.06 |
|  | C021R065 | enoyl- hydratase domain-containing protein mitochondrial-like | 2.06 |
|  | C164R036 | tsc22 domain member 1 | 2.06 |
|  | C142R105 | n -dimethylarginine dimethylaminohydrolase 2 | 2.06 |
|  | C060R028 | cd209 antigen-like | 2.06 |
|  | C139R049 | mitochondrial precursor | 2.05 |
|  | C062R120 | disulfide-isomerase a6 precursor | 2.05 |
|  | C140R158 | charged multivesicular body protein 3 | 2.05 |
|  | C133R143 | involucrin-like isoform x3 | 2.05 |
|  | C053R044 | pleiotrophic factor-alpha-2 precursor | 2.05 |
|  | C016R072 | nucleolysin tiar | 2.05 |
|  | C013R057 | synaptosomal-associated protein 25 | 2.05 |
|  | C199R049 | multiple inositol polyphosphate phosphatase 1-like | 2.05 |
|  | C085R160 | phosphorylase b kinase gamma catalytic testis liver isoform | 2.05 |
|  | C051R140 | secreted frizzled-related protein 3-like | 2.05 |
|  | C022R015 | mbd3 protein | 2.05 |
|  | C148R111 | sideroflexin-4 | 2.05 |
|  | C091R089 | type-1 angiotensin ii receptor-associated | 2.04 |
|  | C202R062 | selenophosphate synthetase 2 | 2.04 |
|  | C263R060 | alpha-n-acetylgalactosaminide alpha-sialyltransferase 3-like | 2.04 |
|  | C152R041 | UNKNOWN | 2.04 |
|  | C200R163 | UNKNOWN | 2.04 |
|  | C056R005 | estrogen receptor binding site 9 | 2.03 |
|  | C167R083 | v-type proton atpase subunit s1-like | 2.03 |
|  | C059R041 | chloride intracellular channel protein 1 | 2.02 |
|  | C085R070 | rna binding protein with multiple splicing 2 | 2.02 |
|  | C096R075 | mothers against decapentaplegic homolog 3 isoform 2 | 2.02 |
|  | C225R065 | transmembrane protein 59-like | 2.02 |
|  | C203R023 | UNKNOWN | 2.02 |
|  | C205R001 | n-lysine methyltransferase setd8-a-like | 2.02 |
|  | C047R087 | protein sec13 homolog isoform 1 | 2.02 |
|  | C042R075 | UNKNOWN | 2.02 |
|  | C160R072 | tripartite motif-containing protein 35-like | 2.02 |
|  | C182R004 | cd63 antigen | 2.02 |
|  | C248R006 | 26s protease regulatory subunit 6a | 2.01 |
|  | C011R036 | leucine-rich glioma-inactivated protein 1 precursor | 2.01 |
|  | C032R048 | bcl2 adenovirus e1b 19 kda protein-interacting protein 3 | 2.01 |
|  | C200R139 | septin 10 | 2.01 |
|  | C193R045 | elongation of very long chain fatty acids protein 6-like | 2.01 |
|  | C171R165 | upf0368 protein cxorf26-like | 2.01 |
|  | C151R001 | u1 small nuclear ribonucleoprotein c | 2.01 |
|  | C035R139 | claudin-like protein zf-a89 | 2.01 |
|  | C153R160 | cell cycle control protein 50b | 2.01 |
|  | C063R015 | na k atpase alpha subunit isoform 1c | 2.00 |
|  | C027R094 | echinoderm microtubule associated protein like 1 | 2.00 |
|  | C158R098 | rhomboid-related protein 2 | 2.00 |
|  | C077R036 | cell division cycle protein 123 homolog | 2.00 |
|  | C258R084 | ras-related protein rap-2c | 2.00 |
|  | C126R082 | UNKNOWN | 2.00 |
|  | C198R087 | alpha-synuclein | 2.00 |
|  | C172R123 | fish virus induced trim protein | 1.99 |
|  | C179R046 | serine threonine-protein kinase sgk3 | 1.99 |
|  | C154R104 | cytosolic non-specific dipeptidase | 1.99 |
|  | C007R077 | u4 tri-snrnp-associated protein 2 isoform 2 | 1.99 |
|  | C200R067 | nuclear transcription factor y subunit alpha-like | 1.99 |
|  | C024R083 | UNKNOWN | 1.99 |
|  | C035R055 | repressor of rna polymerase iii transcription maf1 homolog | 1.99 |
|  | C211R110^‡^ | c4b-binding protein alpha chain precursor | 1.99 |
|  | C208R062 | daz-associated protein 1 | 1.98 |
|  | C256R110 | beta-galactosyltransferase 1 | 1.98 |
|  | C178R006 | calpain small subunit 1 | 1.98 |
|  | C158R041 | zinc finger protein zpr1 | 1.98 |
|  | C119R045 | glutaminase kidney mitochondrial-like | 1.98 |
|  | C081R087 | nucleolysin tia-1 isoform p40 isoform 2 | 1.98 |
|  | C048R135 | max protein | 1.97 |
|  | C176R102 | antizyme inhibitor 1 | 1.97 |
|  | C122R045 | hcls1-associated protein x-1 | 1.97 |
|  | C035R042 | v-maf musculoaponeurotic fibrosarcoma oncogene-like | 1.97 |
|  | C074R132 | e3 ubiquitin-protein ligase rnf146-like | 1.96 |
|  | C127R002 | septin-6 isoform 1 | 1.96 |
|  | C207R078 | nf-kappa-b inhibitor-interacting ras-like protein 2 | 1.96 |
|  | C008R077 | prostaglandin e synthase 3 | 1.96 |
|  | C246R120 | claudin-like protein zf-a89 | 1.96 |
|  | C238R011 | adenosine kinase | 1.96 |
|  | C193R062 | mitochondrial glutamate carrier 1-like | 1.96 |
|  | C070R056 | tumor-associated calcium signal transducer 2 precursor | 1.96 |
|  | C117R159 | spermidine synthase | 1.96 |
|  | C084R102 | beta-glucuronidase precursor | 1.96 |
|  | C093R080 | tgf-beta receptor type-2-like | 1.96 |
|  | C106R060 | t-cell acute lymphocytic leukemia protein 2 | 1.95 |
|  | C047R023 | calcium release-activated calcium channel protein 1 | 1.95 |
|  | C246R145 | UNKNOWN | 1.95 |
|  | C222R034 | serine threonine-protein phosphatase 2a 65 kda regulatory subunit a beta isoform-like | 1.95 |
|  | C165R131 | death-associated protein 1 | 1.95 |
|  | C209R002 | wd repeat-containing protein 82 | 1.95 |
|  | C251R060 | c4b-binding protein alpha chain precursor | 1.94 |
|  | C025R088 | arrestin domain-containing protein 2 | 1.94 |
|  | C167R129 | polycomb complex protein bmi-1 | 1.94 |
|  | C089R143 | wd repeat-containing protein 92 | 1.94 |
|  | C057R004 | maspardin isoform 1 | 1.94 |
|  | C048R103 | nucleosome assembly protein 1-like 1 | 1.94 |
|  | C209R004 | cd209 antigen-like protein a | 1.94 |
|  | C108R105 | isovaleryl coenzyme a dehydrogenase | 1.94 |
|  | C079R071 | profilin-2 | 1.93 |
|  | C062R129 | inhibitor of growth protein 5 | 1.93 |
|  | C166R060 | ras-related protein rab-3a | 1.93 |
|  | C216R002 | zinc finger protein 850-like | 1.93 |
|  | C021R131 | akt-interacting protein | 1.93 |
|  | C201R001 | plastin-2 isoform 1 | 1.93 |
|  | C157R099 | low quality protein: cell cycle control protein 50a | 1.92 |
|  | C088R127 | protein yipf1-like | 1.92 |
|  | C161R139 | neurogenic differentiation factor 2 | 1.92 |
|  | C227R087 | protein atp1b4-like | 1.92 |
|  | C024R163 | elav-like protein 1 | 1.92 |
|  | C122R001 | retinoic acid receptor rxr-beta-a | 1.92 |
|  | C206R080 | intraflagellar transport protein 52 homolog | 1.92 |
|  | C201R149 | UNKNOWN | 1.92 |
|  | C197R044 | solute carrier family 35 member f1 | 1.92 |
|  | C097R156 | pyridoxal phosphate phosphatase | 1.91 |
|  | C263R038 | UNKNOWN | 1.91 |
|  | C005R151 | probable bax inhibitor 1 | 1.91 |
|  | C164R004 | ino80 complex subunit e | 1.91 |
|  | C193R076 | UNKNOWN | 1.91 |
|  | C240R056 | myosin regulatory light chain smooth muscle isoform | 1.91 |
|  | C128R109 | dynein light chain cytoplasmic | 1.91 |
|  | C015R021 | cytochrome b5 | 1.91 |
|  | C031R067 | heterogeneous nuclear ribonucleoprotein q | 1.90 |
|  | C209R041 | u4 tri-snrnp-associated protein 2 isoform 2 | 1.90 |
|  | C241R024 | epithelial membrane protein 2 | 1.90 |
|  | C264R151 | succinate dehydrogenase | 1.90 |
|  | C194R022 | general transcription factor ii-i repeat domain-containing protein 1-like | 1.90 |
|  | C012R108 | prostaglandin e synthase 3 | 1.90 |
|  | C010R058 | g protein-activated inward rectifier potassium channel 3 | 1.90 |
|  | C240R105 | fxyd domain-containing ion transport regulator 6 precursor | 1.90 |
|  | C218R064 | fish virus induced trim protein | 1.90 |
|  | C104R163 | epoxide hydrolase 1-like | 1.89 |
|  | C182R027 | heat shock protein hsp 90-alpha | 1.89 |
|  | C021R083 | keratin 8 | 1.89 |
|  | C055R055 | guanine nucleotide-binding protein g g g subunit beta-1-like | 1.89 |
|  | C213R030 | eukaryotic translation initiation factor 3 subunit g | 1.88 |
|  | C203R131 | UNKNOWN | 1.88 |
|  | C239R088 | complexin-1 | 1.88 |
|  | C195R055 | rna-binding protein 12b | 1.88 |
|  | C237R056 | torsin-1b | 1.88 |
|  | C145R037 | probable alpha-ketoglutarate-dependent dioxygenase abh7-like | 1.88 |
|  | C137R144 | riboflavin kinase | 1.88 |
|  | C148R154 | cop9 signalosome complex subunit 2 isoform 1 | 1.88 |
|  | C101R117 | UNKNOWN | 1.88 |
|  | C073R082 | UNKNOWN | 1.88 |
|  | C048R100 | UNKNOWN | 1.88 |
|  | C030R053 | growth associated protein 43 | 1.88 |
|  | C231R149 | calcineurin subunit b type 1 | 1.88 |
|  | C147R029 | sun domain-containing protein 1 | 1.87 |
|  | C117R002 | atp-dependent rna helicase tdrd9-like | 1.87 |
|  | C216R004 | high affinity camp-specific 3 -cyclic phosphodiesterase 7a | 1.87 |
|  | C258R035 | sal-like protein 4 | 1.87 |
|  | C065R021 | probable bax inhibitor 1 | 1.87 |
|  | C116R085 | mitochondrial-like | 1.87 |
|  | C081R073 | phosphatidylinositol transfer protein alpha isoform | 1.87 |
|  | C012R079 | transcription factor hes-5-like | 1.87 |
|  | C195R042 | gdp-mannose dehydratase | 1.87 |
|  | C005R076 | cell growth regulator with ring finger domain protein 1 | 1.87 |
|  | C193R034 | diphthamide biosynthesis protein 2-like | 1.87 |
|  | C118R084 | gtp-binding nuclear protein ran | 1.87 |
|  | C083R120 | protein kinase c iota type | 1.87 |
|  | C212R032 | vesicle transport protein got1b | 1.87 |
|  | C243R149 | cerebellin- partial | 1.87 |
|  | C011R155 | zinc finger ran-binding domain-containing protein 2 | 1.86 |
|  | C066R122 | vacuolar proton pump subunit e 1 | 1.86 |
|  | C089R121 | ring finger protein 11 | 1.86 |
|  | C177R090 | protein mal2 | 1.86 |
|  | C194R037 | sparc precursor | 1.86 |
|  | C054R144 | transport and golgi organization 2 homolog | 1.86 |
|  | C174R012 | unconventional myosin-ic | 1.86 |
|  | C214R127 | glutamate decarboxylase 1-like | 1.86 |
|  | C232R100 | neuronal calcium sensor 1 | 1.86 |
|  | C081R160 | acidic fibroblast growth factor intracellular-binding | 1.86 |
|  | C109R033 | uv excision repair protein rad23 homolog a-like | 1.86 |
|  | C225R155 | protein fam60a-like | 1.85 |
|  | C065R151 | double-strand-break repair protein rad21 homolog | 1.85 |
|  | C040R034 | wd repeat-containing protein 1 | 1.85 |
|  | C035R050 | UNKNOWN | 1.85 |
|  | C121R086 | caspase 3 | 1.85 |
|  | C250R065 | ig kappa chain v-iv region b17 precursor | 1.85 |
|  | C225R168 | fish virus induced trim protein | 1.85 |
|  | C190R001 | epithelial membrane protein 3-like | 1.85 |
|  | C244R136 | glutamate decarboxylase 1 | 1.85 |
|  | C043R134 | kinesin heavy chain isoform 5a isoform 2 | 1.85 |
|  | C230R006 | btb poz domain-containing protein 6 | 1.84 |
|  | C170R106 | cysteine mitochondrial | 1.84 |
|  | C061R167 | UNKNOWN | 1.84 |
|  | C220R076 | n-terminal ef-hand calcium-binding protein 1-like | 1.84 |
|  | C104R043 | sh3 domain-containing protein 19-like | 1.84 |
|  | C143R026 | UNKNOWN | 1.84 |
|  | C191R075 | ubiquitin-like modifier-activating enzyme 5 | 1.84 |
|  | C244R038 | multiple inositol polyphosphate phosphatase 1-like | 1.84 |
|  | C174R032 | swi snf-related matrix-associated actin-dependent regulator of chromatin subfamily e member 1-related | 1.84 |
|  | C246R138 | tumor-associated calcium signal transducer 2 precursor | 1.84 |
|  | C144R036 | lim homeobox protein lhx9 isoform x1 | 1.84 |
|  | C160R170 | 40s ribosomal protein sa | 1.84 |
|  | C035R135 | cln3 protein | 1.84 |
|  | C134R084 | septin isoform cra_a | 1.84 |
|  | C053R121 | UNKNOWN | 1.84 |
|  | C215R086 | protein mab-21-like 1 | 1.84 |
|  | C138R056 | envoplakin-like | 1.84 |
|  | C060R006 | hemoglobin subunit beta-1 | 1.84 |
|  | C119R140 | run and fyve domain containing 3 | 1.83 |
|  | C109R067 | transmembrane 9 superfamily member 3 | 1.83 |
|  | C210R131 | histone -like | 1.83 |
|  | C254R126 | nicotinamide mononucleotide adenylyltransferase 2 | 1.83 |
|  | C147R039 | neuritin | 1.83 |
|  | C179R138 | protein fam60a-like | 1.83 |
|  | C262R069 | sodium-coupled neutral amino acid transporter 2 | 1.83 |
|  | C067R055 | proliferation-associated protein 2g4-like | 1.83 |
|  | C094R116 | death-associated protein kinase 3 | 1.83 |
|  | C076R127 | adp-ribosylation factor-like protein 9 | 1.83 |
|  | C015R103 | methylosome protein 50 | 1.83 |
|  | C258R045 | osteoblast specific factor | 1.83 |
|  | C038R022 | electron transfer flavoprotein-ubiquinone mitochondrial-like | 1.83 |
|  | C143R089 | sam domain and hd domain-containing protein 1 | 1.82 |
|  | C096R039 | follistatin-related protein 1 precursor | 1.82 |
|  | C062R155 | ornithine decarboxylase antizyme 1 | 1.82 |
|  | C049R073 | protein kinase c and casein kinase substrate in neurons 3 | 1.82 |
|  | C221R021 | nuclear prelamin a recognition factor | 1.82 |
|  | C043R082 | protein mab-21-like 2 | 1.82 |
|  | C194R070 | spermine oxidase-like | 1.82 |
|  | C244R034 | phosphatidylinositol transfer protein beta isoform isoform 1 | 1.82 |
|  | C076R102 | cyclin-g2-like | 1.82 |
|  | C164R125 | cd99 antigen precursor | 1.82 |
|  | C168R046 | transposable element tcb1 transposase | 1.82 |
|  | C204R070 | vacuolar protein sorting-associated protein 41 homolog | 1.82 |
|  | C127R145 | glutamate receptor 2 isoform x1 | 1.82 |
|  | C168R036 | UNKNOWN | 1.82 |
|  | C179R034 | cop9 signalosome complex subunit 2 isoform 1 | 1.82 |
|  | C083R107 | solute carrier family 7 (cationic amino acid y+ system) member 3 | 1.82 |
|  | C021R127 | matrix metalloproteinase 2 | 1.82 |
|  | C053R036 | actin-related protein 2 | 1.82 |
|  | C218R126 | wd repeat domain-containing protein 83 | 1.82 |
|  | C069R041 | 14-3-3 protein gamma | 1.81 |
|  | C041R006 | UNKNOWN | 1.81 |
|  | C170R168 | casein kinase i isoform alpha | 1.81 |
|  | C072R052 | mitochondrial inner membrane protease subunit 2-like | 1.81 |
|  | C043R020 | UNKNOWN | 1.81 |
|  | C037R006 | UNKNOWN | 1.81 |
|  | C073R105 | beta- -galactosyltransferase 2-like | 1.81 |
|  | C113R034 | calpain-9 isoform 3 | 1.81 |
|  | C176R005 | protein farnesyltransferase subunit beta-like | 1.81 |
|  | C036R048 | protein max-like isoform 3 | 1.81 |
|  | C031R083 | long-chain-fatty-acid-- ligase acsbg2-like | 1.81 |
|  | C187R103 | lipoma hmgic fusion partner-like 3 | 1.81 |
|  | C039R151 | akt-interacting protein | 1.81 |
|  | C107R064 | u4 u6 small nuclear ribonucleoprotein prp31-like | 1.81 |
|  | C117R012 | peptidyl-prolyl cis-trans isomerase-like 4 | 1.80 |
|  | C155R073 | serine threonine-protein phosphatase 2a 55 kda regulatory subunit b delta isoform | 1.80 |
|  | C141R110 | tropomyosin alpha-4 chain isoform 8 | 1.80 |
|  | C216R079 | protein set-like | 1.80 |
|  | C058R138 | ring finger and spry domain-containing protein 1 | 1.80 |
|  | C244R101 | atp-sensitive inward rectifier potassium channel 11-like | 1.80 |
|  | C065R158 | membrane-associated progesterone receptor component 1 | 1.80 |
|  | C130R075 | coup transcription factor 1 | 1.80 |
|  | C079R106 | golgin subfamily a member 7 | 1.80 |
|  | C073R062 | ring finger protein 223-like isoform x1 | 1.80 |
|  | C184R151 | probable peptidyl-trna hydrolase | 1.80 |
|  | C053R137 | guanine nucleotide-binding protein g g g subunit gamma-3 | 1.80 |
|  | C250R018 | protein-coding gene cg057 like protein | 1.80 |
|  | C036R027 | pith domain-containing protein 1-like | 1.80 |
|  | C177R020 | apoptosis regulator bcl-x | 1.80 |
|  | C264R071 | v-type proton atpase subunit d 1 | 1.80 |
|  | C174R039 | homeobox protein dlx-1 | 1.80 |
|  | C172R070 | cytochrome p450 2m1 | 1.79 |
|  | C082R123 | polycomb complex protein bmi-1-a | 1.79 |
|  | C054R088 | tubulin beta-2a chain | 1.79 |
|  | C266R103 | oocyte zinc finger protein 6-like | 1.79 |
|  | C108R066 | nedd4 family-interacting protein 1 | 1.79 |
|  | C036R074 | novel protein | 1.79 |
|  | C256R109 | heat shock protein 70 | 1.79 |
|  | C151R038 | cell surface protein | 1.79 |
|  | C172R138 | kh domain- rna- signal transduction-associated protein 1-like | 1.79 |
|  | C193R140 | cystinosin | 1.79 |
|  | C148R103 | methylosome protein 50 | 1.79 |
|  | C199R003 | phosphatidylinositol 4-kinase type 2-alpha | 1.79 |
|  | C041R160 | ras-related protein rab-11a | 1.79 |
|  | C089R133 | insulin-like growth factor-binding protein 7-like | 1.79 |
|  | C012R069 | choline ethanolaminephosphotransferase 1 | 1.79 |
|  | C261R067 | UNKNOWN | 1.78 |
|  | C179R060 | ubiquitin-conjugating enzyme e2 d2 | 1.78 |
|  | C177R030 | ras-related c3 botulinum toxin substrate 1 (rho small gtp binding protein rac1) | 1.78 |
|  | C178R100 | btb poz domain-containing protein kctd5 | 1.78 |
|  | C111R044 | seizure protein 6 homolog | 1.78 |
|  | C064R164 | schwannomin-interacting protein 1-like | 1.78 |
|  | C200R084 | glutathione transferase omega-1 | 1.78 |
|  | C197R027 | peptidyl-prolyl cis-trans isomerase fkbp4 | 1.78 |
|  | C035R071 | cyclin-dependent kinase 5 activator 1-like | 1.78 |
|  | C017R044 | cerebellin- partial | 1.78 |
|  | C133R097 | myotubularin-related protein 13-like | 1.78 |
|  | C154R140 | alpha- -sialyltransferase st8sia v | 1.78 |
|  | C085R067 | cell division cycle-associated protein 4-like | 1.78 |
|  | C222R075 | cyclin-g2-like | 1.78 |
|  | C216R005 | protein hexim1 | 1.78 |
|  | C254R057 | casein kinase i isoform alpha | 1.78 |
|  | C005R051 | 5 -3 exoribonuclease 2 | 1.78 |
|  | C146R020 | tubulin beta chain-like | 1.78 |
|  | C244R049 | prothymosin alpha | 1.78 |
|  | C085R138 | vacuolar protein sorting-associated protein 28 homolog | 1.77 |
|  | C083R101 | sry-box containing gene 11a | 1.77 |
|  | C054R162 | UNKNOWN | 1.77 |
|  | C093R073 | calcium calmodulin-dependent protein kinase iv | 1.77 |
|  | C187R048 | copine-2 | 1.77 |
|  | C096R128 | bcl2 antagonist of cell death | 1.77 |
|  | C141R151 | growth arrest and dna damage-inducible protein gadd45 beta | 1.77 |
|  | C127R006 | mid1-interacting protein 1-b-like | 1.77 |
|  | C163R124 | kh domain- rna- signal transduction-associated protein 1-like | 1.77 |
|  | C211R019 | myosin-9-like | 1.77 |
|  | C250R040 | secretogranin-2 precursor | 1.77 |
|  | C081R019 | uncharacterized loc100912373 | 1.77 |
|  | C215R022 | gamma-aminobutyric acid a receptor alpha 1 | 1.77 |
|  | C007R036 | glutamate receptor u1-like | 1.77 |
|  | C039R102 | probable atp-dependent rna helicase ddx49 | 1.77 |
|  | C149R126 | c6orf64 homolog | 1.77 |
|  | C062R022 | e3 ubiquitin-protein ligase rnf185 | 1.77 |
|  | C214R086 | neuronal membrane glycoprotein m6-b-like | 1.76 |
|  | C011R162 | biglycan precursor | 1.76 |
|  | C212R001 | loc100137634 protein | 1.76 |
|  | C069R109 | gpi-anchor transamidase-like | 1.76 |
|  | C199R067 | retinol dehydrogenase 12 | 1.76 |
|  | C143R154 | transcription factor sox-21 | 1.76 |
|  | C170R046 | solute carrier family 25 member 40 | 1.76 |
|  | C109R044 | acidic fibroblast growth factor intracellular-binding | 1.76 |
|  | C186R054 | actin-related protein 3 | 1.76 |
|  | C023R069 | glutaminase kidney mitochondrial-like | 1.76 |
|  | C212R037 | asparagine-trna cytoplasmic | 1.76 |
|  | C097R012 | ras-related c3 botulinum toxin substrate 1 (rho small gtp binding protein rac1) | 1.76 |
|  | C145R143 | UNKNOWN | 1.76 |
|  | C087R160 | adenylosuccinate synthetase isozyme 2 | 1.76 |
|  | C215R001 | UNKNOWN | 1.76 |
|  | C012R071 | s-methyl-5 -thioadenosine phosphorylase | 1.76 |
|  | C249R094 | sialic acid synthase | 1.76 |
|  | C262R041 | nad-dependent deacetylase sirtuin-2 | 1.76 |
|  | C197R104 | serine incorporator 1-like | 1.76 |
|  | C200R071 | protein max-like isoform 3 | 1.76 |
|  | C197R114 | peroxisomal coenzyme a diphosphatase nudt7 | 1.76 |
|  | C140R007 | protein crumbs homolog 3-like | 1.76 |
|  | C091R055 | mpn domain-containing protein | 1.75 |
|  | C085R137 | translocon-associated protein subunit alpha-like | 1.75 |
|  | C092R099 | lipopolysaccharide-induced tumor necrosis factor-alpha factor homolog | 1.75 |
|  | C175R001 | choline transporter-like protein 2-like | 1.75 |
|  | C031R110 | microspherule protein 1 | 1.75 |
|  | C135R135 | ornithine decarboxylase antizyme | 1.75 |
|  | C180R109 | serine threonine-protein phosphatase 2a catalytic subunit alpha isoform | 1.75 |
|  | C078R149 | dual specificity protein phosphatase 6 | 1.75 |
|  | C085R077 | cystinosin | 1.75 |
|  | C070R079 | tubulin alpha-1a chain | 1.75 |
|  | C243R121 | peroxisome proliferator-activated receptor beta | 1.75 |
|  | C031R072 | adenylate kinase isoenzyme 6-like | 1.75 |
|  | C177R166 | ring finger protein 122 | 1.75 |
|  | C215R109 | calmodulin | 1.75 |
|  | C121R100 | protein yipf3 | 1.75 |
|  | C096R003 | vacuolar protein sorting-associated protein 41 homolog | 1.74 |
|  | C167R153 | calreticulin precursor | 1.74 |
|  | C069R110 | UNKNOWN | 1.74 |
|  | C192R157 | metalloreductase steap2 | 1.74 |
|  | C117R058 | cadherin-1 isoform 1 | 1.74 |
|  | C232R001 | epididymal secretory protein e1 precursor | 1.74 |
|  | C247R162 | actin-related protein 10 | 1.74 |
|  | C121R058 | claudin-4 | 1.74 |
|  | C216R145 | pescadillo homolog | 1.74 |
|  | C236R146 | alpha-enolase-like isoform 1 | 1.74 |
|  | C028R051 | claudin-4 | 1.74 |
|  | C182R085 | short branched chain specific acyl- mitochondrial | 1.74 |
|  | C177R045 | beta- partial | 1.74 |
|  | C091R069 | synaptosomal-associated protein 25 | 1.74 |
|  | C022R135 | serine hydrolase-like | 1.74 |
|  | C086R164 | teashirt homolog 1 | 1.74 |
|  | C019R119 | ccaat enhancer-binding protein delta | 1.73 |
|  | C226R075 | adp-ribosylation factor-like protein 4a | 1.73 |
|  | C073R164 | regulator of nonsense transcripts 3b | 1.73 |
|  | C051R075 | protein fam89a | 1.73 |
|  | C084R005 | vesicle-associated membrane protein-associated protein b-like | 1.73 |
|  | C214R055 | high mobility group protein b3 | 1.73 |
|  | C192R050 | transmembrane protein 120b | 1.73 |
|  | C226R143 | 26s proteasome non-atpase regulatory subunit 9 | 1.73 |
|  | C112R151 | elmo domain-containing protein 2 | 1.73 |
|  | C087R114 | sodium channel subunit beta-3 | 1.73 |
|  | C193R026 | annexin a13 | 1.73 |
|  | C102R002 | annexin a2-a | 1.73 |
|  | C067R103 | reticulon 4 | 1.73 |
|  | C021R167 | UNKNOWN | 1.73 |
|  | C084R083 | splicing arginine serine-rich 2 | 1.73 |
|  | C231R075 | eukaryotic initiation factor 4a-i | 1.73 |
|  | C141R149 | nitrilase homolog 1-like | 1.73 |
|  | C103R106 | asporin isoform 2 | 1.72 |
|  | C007R104 | denn domain-containing protein 2d | 1.72 |
|  | C071R142 | neuronal migration protein doublecortin isoform 2 | 1.72 |
|  | C177R025 | matrin 3-like | 1.72 |
|  | C202R078 | jnk1 mapk8-associated membrane protein isoform 1 | 1.72 |
|  | C229R008 | methyltransferase ddb_g0268948-like | 1.72 |
|  | C168R163 | mannoside acetylglucosaminyltransferase isoform cra_a | 1.72 |
|  | C232R008 | cation transport regulator-like protein 1 | 1.72 |
|  | C039R075 | atp synthase mitochondrial f1 complex assembly factor 2 | 1.72 |
|  | C016R005 | uba-like domain-containing protein 1 | 1.72 |
|  | C189R045 | ankyrin repeat domain-containing protein 10-like | 1.72 |
|  | C113R002 | UNKNOWN | 1.72 |
|  | C121R089 | ubiquitin-conjugating enzyme e2 b | 1.72 |
|  | C211R168 | zinc finger protein 3 | 1.72 |
|  | C159R069 | inositol polyphosphate 1-phosphatase | 1.72 |
|  | C200R033 | UNKNOWN | 1.72 |
|  | C186R111 | protein ndrg3-like | 1.72 |
|  | C210R115 | ependymin precursor | 1.71 |
|  | C211R005 | gastrotropin | 1.71 |
|  | C186R064 | claudin-5-like | 1.71 |
|  | C007R122 | regulator of g-protein signaling 3-like | 1.71 |
|  | C168R064 | protein sec13 homolog | 1.71 |
|  | C014R030 | cell cycle control protein 50b | 1.71 |
|  | C108R131 | zinc finger and btb domain-containing protein 17 | 1.71 |
|  | C203R039 | cofilin-2 | 1.71 |
|  | C226R013 | adp-ribosylation factor-like protein 2-binding protein | 1.71 |
|  | C046R136 | btb poz domain-containing protein kctd9 | 1.71 |
|  | C080R070 | UNKNOWN | 1.71 |
|  | C196R098 | rna-binding protein 39 | 1.71 |
|  | C175R161 | desmoplakin isoform x1 | 1.71 |
|  | C052R090 | calcium-regulated heat stable protein 1 | 1.71 |
|  | C140R106 | threonine-trna cytoplasmic | 1.71 |
|  | C149R053 | probable rrna-processing protein ebp2 | 1.71 |
|  | C097R097 | poly | 1.71 |
|  | C161R011 | cd99 antigen precursor | 1.70 |
|  | C014R020 | histone h5a | 1.70 |
|  | C072R057 | something about silencing protein 10 | 1.70 |
|  | C266R090 | protein lunapark-b | 1.70 |
|  | C078R043 | 45 kda calcium-binding protein | 1.70 |
|  | C156R020 | cofilin-2 | 1.70 |
|  | C077R161 | heterochromatin protein binding protein isoform cra_a | 1.70 |
|  | C078R052 | integral membrane protein 2c | 1.70 |
|  | C240R067 | zinc finger protein zic 3 | 1.70 |
|  | C154R068 | regulator of g-protein signaling 3-like | 1.70 |
|  | C057R132 | kv channel-interacting protein 1 isoform 4 | 1.70 |
|  | C235R046 | heterogeneous nuclear ribonucleoprotein r | 1.70 |
|  | C148R003 | thymidine cytosolic | 1.70 |
|  | C086R158 | katnb1-like protein 1 | 1.70 |
|  | C217R021 | aryl hydrocarbon receptor nuclear translocator 2 | 1.70 |
|  | C005R149 | guanine nucleotide-binding protein g subunit alpha | 1.70 |
|  | C141R027 | transcription factor sox-2 | 1.70 |
|  | C009R149 | UNKNOWN | 1.70 |
|  | C079R041 | vacuolar protein sorting-associated protein 33b | 1.70 |
|  | C023R044 | heterogeneous nuclear ribonucleoprotein a1 | 1.69 |
|  | C059R078 | translin | 1.69 |
|  | C112R147 | calcium calmodulin-dependent protein kinase ii inhibitor 1 | 1.69 |
|  | C211R001 | calmodulin | 1.69 |
|  | C086R100 | PREDICTED: hypothetical protein LOC100002065 | 1.69 |
|  | C148R052 | monocyte to macrophage differentiation protein | 1.69 |
|  | C023R126 | 5 -amp-activated protein kinase subunit beta-1 | 1.69 |
|  | C057R003 | inositol-3-phosphate synthase 1 | 1.69 |
|  | C067R010 | occludin b | 1.69 |
|  | C114R069 | mitochondrial atp synthase gamma chain | 1.69 |
|  | C182R066 | calreticulin precursor | 1.69 |
|  | C071R132 | UNKNOWN | 1.69 |
|  | C244R148 | histidine triad nucleotide-binding protein 3 | 1.69 |
|  | C171R008 | cornifelin homolog a | 1.69 |
|  | C038R060 | 39s ribosomal protein mitochondrial | 1.69 |
|  | C175R123 | bro1 domain-containing protein brox | 1.69 |
|  | C237R006 | h aca ribonucleoprotein complex subunit 2-like protein | 1.69 |
|  | C040R107 | pleckstrin homology domain-containing family f member 1 | 1.69 |
|  | C208R002 | actin-related protein 2 3 complex subunit 3 | 1.69 |
|  | C240R041 | nucleolysin tia-1 isoform p40 isoform 2 | 1.68 |
|  | C126R103 | epoxide hydrolase 1-like | 1.68 |
|  | C217R118 | stannin | 1.68 |
|  | C104R026 | serine arginine-rich splicing factor 11-like | 1.68 |
|  | C240R097 | glutathione transferase omega-1 | 1.68 |
|  | C220R094 | elav-like protein 1 | 1.68 |
|  | C134R096 | vacuolar protein sorting-associated protein 41 homolog | 1.68 |
|  | C135R154 | metalloproteinase inhibitor 3-like | 1.68 |
|  | C103R033 | interleukin-4 receptor subunit alpha | 1.68 |
|  | C225R039 | ribose-phosphate pyrophosphokinase 2 | 1.68 |
|  | C238R029 | spindle and kinetochore-associated protein 2 | 1.68 |
|  | C087R074 | transmembrane protein 54 isoform 2 | 1.68 |
|  | C185R023 | nucleolar gtp-binding protein 1 | 1.68 |
|  | C010R098 | inhibitor of growth protein 2 | 1.68 |
|  | C048R097 | transmembrane protein 50a | 1.68 |
|  | C261R014 | protein | 1.68 |
|  | C183R048 | casein kinase ii subunit beta | 1.68 |
|  | C195R020 | rhombotin-1 | 1.67 |
|  | C231R106 | membrane protein mlc1-like | 1.67 |
|  | C031R047 | zinc finger protein 596-like | 1.67 |
|  | C169R037 | zinc finger protein 280d | 1.67 |
|  | C244R002 | UNKNOWN | 1.67 |
|  | C188R055 | synaptosomal-associated protein 23 | 1.67 |
|  | C255R014 | neuron-specific protein family member 1 | 1.67 |
|  | C252R159 | nicotinamide riboside kinase 2 | 1.67 |
|  | C205R104 | repulsive guidance molecule a precursor | 1.67 |
|  | C216R115 | bsg protein | 1.67 |
|  | C233R087 | map kinase-activated protein kinase 2 | 1.67 |
|  | C250R160 | cystathionine beta-synthase-like | 1.67 |
|  | C203R062 | scel protein | 1.67 |
|  | C095R034 | calcyphosin-like protein | 1.67 |
|  | C261R155 | splicing factor 3a subunit 3 | 1.67 |
|  | C227R082 | protein fam19a2 | 1.67 |
|  | C237R087 | mannose-1-phosphate guanyltransferase alpha-a | 1.67 |
|  | C014R099 | udp-glucose 4-epimerase | 1.67 |
|  | C216R126 | superoxide dismutase | 1.67 |
|  | C087R011 | nicolin-1 | 1.67 |
|  | C188R145 | pyridoxal phosphate phosphatase phospho2 | 1.67 |
|  | C128R106 | actin-related protein 2 | 1.66 |
|  | C138R034 | serine threonine-protein kinase rio1 | 1.66 |
|  | C034R147 | glyoxylate reductase hydroxypyruvate reductase | 1.66 |
|  | C023R052 | hydroxyacylglutathione hydrolase | 1.66 |
|  | C255R075 | signal peptidase complex catalytic subunit sec11a | 1.66 |
|  | C225R136 | voltage-dependent anion-selective channel protein 2 | 1.66 |
|  | C071R040 | methyltransferase-like protein 5 | 1.66 |
|  | C138R059 | wd repeat and socs box-containing protein 2 | 1.66 |
|  | C229R074 | ist1 homolog | 1.66 |
|  | C146R013 | srp72 protein | 1.66 |
|  | C187R075 | UNKNOWN | 1.66 |
|  | C014R080 | prostaglandin e synthase 3 | 1.66 |
|  | C082R009 | dehydrogenase reductase sdr family member 11 | 1.66 |
|  | C127R051 | purine nucleoside phosphorylase-like | 1.66 |
|  | C112R097 | calcyclin-binding protein | 1.66 |
|  | C151R083 | casein kinase i isoform epsilon-like | 1.66 |
|  | C093R089 | ccr4-not transcription complex subunit 7 | 1.66 |
|  | C126R072 | tetratricopeptide repeat protein mitochondrial | 1.65 |
|  | C040R003 | haus augmin-like complex subunit 8-like | 1.65 |
|  | C194R128 | p53 apoptosis effector related to pmp-22 | 1.65 |
|  | C006R031 | dual specificity mitogen-activated protein kinase kinase 4-like | 1.65 |
|  | C133R146 | 14-3-3 protein beta alpha | 1.65 |
|  | C216R102 | 4f2 cell-surface antigen heavy chain | 1.65 |
|  | C074R159 | btb poz domain-containing protein kctd17 | 1.65 |
|  | C182R118 | follistatin-related protein 1 precursor | 1.65 |
|  | C249R023 | calpain small subunit 1 | 1.65 |
|  | C020R096 | oocyte zinc finger protein 6-like | 1.65 |
|  | C113R122 | nuclease harbi1 | 1.65 |
|  | C096R028 | lysosomal protective protein | 1.65 |
|  | C190R048 | n-acetyltransferase nat13 | 1.65 |
|  | C114R149 | protein set-like | 1.65 |
|  | C161R128 | septin 5 | 1.65 |
|  | C079R100 | ring finger protein 122 | 1.65 |
|  | C186R046 | nf-kappa-b inhibitor-interacting ras-like protein 2 | 1.65 |
|  | C264R056 | sept8a protein | 1.65 |
|  | C151R024 | alpha-enolase | 1.65 |
|  | C054R089 | ap-1 complex subunit sigma-2 | 1.65 |
|  | C016R014 | receptor expression-enhancing protein 5 | 1.65 |
|  | C159R026 | active breakpoint cluster region-related protein isoform 4 | 1.64 |
|  | C227R023 | dehydrogenase reductase sdr family member 12-like | 1.64 |
|  | C192R120 | synapsin-2-like isoform 1 | 1.64 |
|  | C050R160 | glutamate decarboxylase 1 | 1.64 |
|  | C221R115 | swi snf-related matrix-associated actin-dependent regulator of chromatin subfamily e member 1-related | 1.64 |
|  | C245R113 | zinc finger protein 576 | 1.64 |
|  | C197R107 | uridine-cytidine kinase 1 | 1.64 |
|  | C081R018 | UNKNOWN | 1.64 |
|  | C075R031 | protein fam199x | 1.64 |
|  | C023R075 | transmembrane emp24 domain-containing protein 3 precursor | 1.64 |
|  | C052R075 | zinc finger protein 25 | 1.64 |
|  | C053R065 | polyadenylate-binding protein 2 | 1.64 |
|  | C091R079 | probable g-protein coupled receptor 88 | 1.64 |
|  | C255R113 | transmembrane protein 9b precursor | 1.64 |
|  | C025R016 | UNKNOWN | 1.64 |
|  | C032R036 | btb poz domain-containing protein kctd5 | 1.64 |
|  | C097R119 | l-seryl-trna kinase | 1.64 |
|  | C207R080 | lysosomal protective protein | 1.64 |
|  | C118R004 | UNKNOWN | 1.64 |
|  | C205R088 | rna-binding protein musashi homolog 1 | 1.64 |
|  | C010R135 | adaptin ear-binding coat-associated protein 1 | 1.64 |
|  | C005R058 | ependymin precursor | 1.64 |
|  | C185R060 | uv excision repair protein rad23 homolog b | 1.64 |
|  | C061R161 | ubiquitin carboxyl-terminal hydrolase isozyme l3 | 1.64 |
|  | C187R024 | tetratricopeptide repeat protein 32 | 1.63 |
|  | C196R072 | protein fam50a | 1.63 |
|  | C039R021 | c-x-c motif chemokine 14 precursor | 1.63 |
|  | C140R157 | probable hydrolase pnkd isoform 2 | 1.63 |
|  | C093R095 | ribosome biogenesis protein nsa2 homolog | 1.63 |
|  | C083R135 | guanine nucleotide-binding protein g subunit alpha-2 | 1.63 |
|  | C071R090 | casein kinase i isoform alpha isoform 1 | 1.63 |
|  | C175R003 | loc553515 partial | 1.63 |
|  | C067R005 | transmembrane protease serine 9-like | 1.63 |
|  | C061R129 | actin-related protein 2 | 1.63 |
|  | C195R089 | ras-related and estrogen-regulated growth inhibitor | 1.63 |
|  | C220R128 | adp-ribosylation factor 1 | 1.63 |
|  | C079R035 | probable saccharopine dehydrogenase | 1.63 |
|  | C069R043 | hig1 domain family member 1a | 1.63 |
|  | C159R049 | hematological and neurological expressed 1 | 1.63 |
|  | C162R086 | 26s protease regulatory subunit 6b isoform 1 | 1.63 |
|  | C074R100 | septin-9-like isoform x2 | 1.63 |
|  | C092R059 | eukaryotic initiation factor 4a-ii | 1.63 |
|  | C222R158 | basic leucine zipper and w2 domain-containing protein 1-a-like | 1.62 |
|  | C094R026 | oxidative stress responsive 1 | 1.62 |
|  | C073R114 | t-complex protein 1 subunit delta | 1.62 |
|  | C069R144 | ring finger protein 4 | 1.62 |
|  | C143R119 | purpurin precursor | 1.62 |
|  | C028R044 | protein lunapark-b | 1.62 |
|  | C172R139 | platelet receptor gi24 | 1.62 |
|  | C218R079 | v-type proton atpase 116 kda subunit a isoform 1-like isoform 2 | 1.62 |
|  | C050R027 | protein lin-7 homolog c | 1.62 |
|  | C243R154 | PREDICTED: hypothetical protein LOC100698742 | 1.62 |
|  | C074R084 | probable palmitoyltransferase zdhhc16-like | 1.62 |
|  | C120R116 | eukaryotic peptide chain release factor subunit 1 | 1.62 |
|  | C078R056 | mitochondrial precursor | 1.62 |
|  | C112R139 | zinc finger ran-binding domain-containing protein 2 | 1.62 |
|  | C084R047 | lysophospholipase i | 1.62 |
|  | C154R168 | transmembrane protein 59-like | 1.62 |
|  | C048R094 | dehydrogenase reductase sdr family member 11-like | 1.62 |
|  | C057R130 | upf0468 protein c16orf80 homolog | 1.62 |
|  | C106R010 | caspase 3 | 1.62 |
|  | C145R165 | 3 -5 exoribonuclease 1-like | 1.62 |
|  | C147R069 | atpase family aaa domain-containing protein 1 | 1.62 |
|  | C057R148 | rho guanine nucleotide exchange factor 4 | 1.62 |
|  | C162R021 | protein arginine n-methyltransferase 5 isoform 1 | 1.62 |
|  | C145R034 | nucleosome assembly protein 1-like 1 | 1.62 |
|  | C264R142 | tight junction protein zo-1-like | 1.61 |
|  | C047R034 | ras-related protein rab-2a | 1.61 |
|  | C010R150 | protein sys1 homolog | 1.61 |
|  | C196R097 | bccip homolog | 1.61 |
|  | C103R078 | protein mal2 | 1.61 |
|  | C142R151 | fish virus induced trim protein | 1.61 |
|  | C025R081 | multivesicular body subunit 12a | 1.61 |
|  | C201R005 | type ii keratin e3 | 1.61 |
|  | C069R103 | exocyst complex component 1 | 1.61 |
|  | C209R067 | gap junction beta-4 protein | 1.61 |
|  | C166R106 | serine threonine-protein phosphatase pp1-gamma catalytic subunit | 1.61 |
|  | C067R160 | UNKNOWN | 1.61 |
|  | C156R002 | circularly permutated ras protein 1-like | 1.61 |
|  | C040R125 | homeobox protein dlx-3 | 1.61 |
|  | C058R067 | transcription initiation factor tfiid subunit 10 | 1.61 |
|  | C174R015 | cap-specific mrna (nucleoside-2 -o-)-methyltransferase 1 | 1.61 |
|  | C017R025 | beta-catenin-interacting protein 1 | 1.61 |
|  | C074R167 | protein manbal | 1.61 |
|  | C207R100 | dcn1-like protein 5 | 1.61 |
|  | C150R009 | heat shock 70 kda protein 4l | 1.61 |
|  | C204R046 | tcf3 fusion partner homolog | 1.61 |
|  | C199R025 | serine threonine-protein phosphatase 2a 65 kda regulatory subunit a beta isoform-like | 1.61 |
|  | C039R032 | phosphogluconate dehydrogenase | 1.61 |
|  | C069R127 | UNKNOWN | 1.60 |
|  | C174R051 | cytochrome b-c1 complex subunit 10 | 1.60 |
|  | C146R061 | UNKNOWN | 1.60 |
|  | C106R080 | insulin-like growth factor binding protein 5 | 1.60 |
|  | C061R103 | vesicular glutamate transporter 1-like | 1.60 |
|  | C032R007 | translocon-associated protein subunit alpha-like | 1.60 |
|  | C096R080 | palmitoyltransferase zdhhc3-like | 1.60 |
|  | C168R130 | beta-glucuronidase-like | 1.60 |
|  | C258R155 | transcription initiation factor tfiid subunit 10 | 1.60 |
|  | C226R020 | UNKNOWN | 1.60 |
|  | C259R150 | cytochrome c oxidase subunit viia-related mitochondrial precursor | 1.60 |
|  | C228R005 | probable g-protein coupled receptor 116 | 1.60 |
|  | C051R123 | tetraspanin-7 | 1.60 |
|  | C092R117 | dna chr wayne state university expressed | 1.60 |
|  | C172R085 | mki67 fha domain-interacting nucleolar phospho | 1.60 |
|  | C259R070 | receptor expression-enhancing protein 5 | 1.60 |
|  | C039R084 | rad51-associated protein 1 | 1.60 |
|  | C021R027 | histone h5a | 1.60 |
|  | C096R044 | regulator of g-protein signaling 5-like | 1.60 |
|  | C159R100 | rna methyltransferase like 1 | 1.60 |
|  | C026R074 | transcription initiation factor tfiid subunit 7 | 1.60 |
|  | C072R127 | beta- partial | 1.60 |
|  | C114R059 | bccip homolog | 1.60 |
|  | C263R035 | charged multivesicular body protein 2a | 1.60 |
|  | C223R038 | delta-6 fatty acyl desaturase | 1.60 |
|  | C088R162 | endoplasmic reticulum resident protein 44 | 1.60 |
|  | C214R069 | inositol monophosphatase 2 | 1.59 |
|  | C226R072 | isoform a | 1.59 |
|  | C194R054 | glutathione s-transferase | 1.59 |
|  | C088R139 | ubiquitin carboxyl-terminal hydrolase 47 | 1.59 |
|  | C248R022 | wnt1-inducible-signaling pathway protein 1-like | 1.59 |
|  | C248R166 | glycogen liver form | 1.59 |
|  | C195R092 | fructose- -bisphosphatase tigar b-like | 1.59 |
|  | C218R159 | UNKNOWN | 1.59 |
|  | C188R035 | hyaluronan and proteoglycan link protein 1 | 1.59 |
|  | C263R085 | adp-ribosylation factor 1 | 1.59 |
|  | C098R105 | nicotinamide riboside kinase 2 | 1.59 |
|  | C112R116 | UNKNOWN | 1.59 |
|  | C226R146 | u2 small nuclear ribonucleoprotein a | 1.59 |
|  | C141R030 | arrestin-c-like | 1.59 |
|  | C266R075 | asparagine-trna cytoplasmic | 1.59 |
|  | C246R123 | spry domain-containing protein 7 isoform 1 | 1.59 |
|  | C185R024 | rna-binding protein nob1 | 1.59 |
|  | C115R006 | farnesyl pyrophosphate synthase | 1.59 |
|  | C071R122 | ras-related gtp-binding protein a | 1.59 |
|  | C016R043 | calpain-1 catalytic subunit-like | 1.59 |
|  | C018R088 | phosphoethanolamine n-methyltransferase 3 | 1.59 |
|  | C203R089 | kinesin light chain 4 | 1.59 |
|  | C075R032 | surfeit locus protein 6 | 1.58 |
|  | C216R024 | dnaj homolog subfamily a member mitochondrial isoform 2 | 1.58 |
|  | C063R030 | tumor-associated calcium signal transducer 2 precursor | 1.58 |
|  | C094R097 | btb poz domain-containing protein kctd17 | 1.58 |
|  | C112R088 | 26s proteasome non-atpase regulatory subunit 7 | 1.58 |
|  | C024R015 | hcls1-binding protein 3 | 1.58 |
|  | C222R106 | lysine-specific demethylase 5b-like | 1.58 |
|  | C081R161 | stathmin | 1.58 |
|  | C095R007 | kiaa1143 homolog | 1.58 |
|  | C147R046 | prefoldin subunit 4 | 1.58 |
|  | C030R142 | btb poz domain-containing protein kctd20 | 1.58 |
|  | C240R118 | atp-citrate synthase-like | 1.58 |
|  | C041R118 | lipid phosphate phosphatase-related protein type 1 | 1.58 |
|  | C065R084 | selenoprotein 1 | 1.58 |
|  | C092R064 | ras-related protein rab-27a | 1.58 |
|  | C084R155 | necap endocytosis associated 2 | 1.58 |
|  | C173R029 | zinc finger ran-binding domain-containing protein 2 | 1.58 |
|  | C022R055 | glucosamine 6-phosphate n-acetyltransferase | 1.58 |
|  | C215R031 | dmx-like protein 2-like | 1.58 |
|  | C173R060 | neighbor of cox4 | 1.58 |
|  | C261R113 | protein kish-a | 1.58 |
|  | C017R145 | histidine kinase- dna gyrase b- and hsp90-like domain containing protein | 1.58 |
|  | C078R127 | 28 kda heat- and acid-stable phosphoprotein | 1.58 |
|  | C112R150 | kxdl motif-containing protein 1 | 1.58 |
|  | C109R051 | inhibitor of growth protein 4 | 1.58 |
|  | C180R157 | mediator of rna polymerase ii transcription subunit 29 | 1.57 |
|  | C082R091 | acidic coiled-coil containing protein 3 | 1.57 |
|  | C054R157 | small ubiquitin-related modifier 3 precursor | 1.57 |
|  | C185R081 | transmembrane 4 l6 family member 1-like | 1.57 |
|  | C245R010 | protein pelota homolog | 1.57 |
|  | C120R088 | krr1 small subunit processome component homolog | 1.57 |
|  | C240R094 | o-phosphoseryl-trna selenium transferase | 1.57 |
|  | C139R135 | carbonyl reductase | 1.57 |
|  | C140R044 | iroquois-class homeodomain protein irx-5 | 1.57 |
|  | C015R080 | eh domain-containing protein 1 | 1.57 |
|  | C048R158 | scel protein | 1.57 |
|  | C074R138 | abhydrolase domain-containing protein 4 | 1.57 |
|  | C061R058 | ras-related protein rab-27b | 1.57 |
|  | C210R085 | general transcription factor iie subunit 2 | 1.57 |
|  | C218R083 | h+ v1 subunit h | 1.57 |
|  | C151R158 | transforming protein precursor | 1.57 |
|  | C238R026 | solute carrier family 35 member b1 | 1.57 |
|  | C015R083 | zinc finger protein 420-like | 1.57 |
|  | C181R051 | stathmin | 1.57 |
|  | C030R149 | serine threonine-protein phosphatase 2a 55 kda regulatory subunit b delta isoform-like | 1.57 |
|  | C009R089 | peroxisomal -dienoyl- reductase | 1.57 |
|  | C177R056 | gem-associated protein 8-like | 1.57 |
|  | C264R017 | cd59 glycoprotein precursor | 1.57 |
|  | C030R133 | death-associated 1-a | 1.57 |
|  | C113R065 | chromosome 11 open reading frame 87 | 1.57 |
|  | C210R057 | inhibitor of growth member 5 | 1.57 |
|  | C039R048 | dna-directed rna polymerase ii subunit rpb11-a | 1.56 |
|  | C194R168 | proteasome activator complex subunit 3 | 1.56 |
|  | C036R041 | cathepsin d | 1.56 |
|  | C022R027 | trafficking protein particle complex subunit 2-like protein | 1.56 |
|  | C202R040 | schwannomin-interacting protein 1 | 1.56 |
|  | C196R108 | UNKNOWN | 1.56 |
|  | C177R058 | probable ergosterol biosynthetic protein 28 | 1.56 |
|  | C245R038 | UNKNOWN | 1.56 |
|  | C248R031 | tho complex subunit 3 | 1.56 |
|  | C170R165 | biglycan isoform 2 | 1.56 |
|  | C246R076 | ubiquitin-conjugating enzyme e2 d2 | 1.56 |
|  | C135R092 | adp-ribosylation factor-like protein 5a | 1.56 |
|  | C029R069 | rna-binding protein 39 | 1.56 |
|  | C162R064 | galectin-related protein b-like | 1.56 |
|  | C063R006 | tetraspanin-7 | 1.56 |
|  | C205R083 | cop9 signalosome complex subunit 2 isoform 1 | 1.56 |
|  | C192R042 | alpha-aminoadipic semialdehyde dehydrogenase | 1.56 |
|  | C078R042 | heat shock 70 kda protein 4-like | 1.56 |
|  | C019R145 | protein tmed8 | 1.56 |
|  | C067R019 | cop9 signalosome complex subunit 7a | 1.56 |
|  | C057R031 | chaperonin containing subunit 7 | 1.56 |
|  | C223R043 | protein max-like isoform 3 | 1.56 |
|  | C041R055 | partner of y14 and mago | 1.55 |
|  | C180R008 | vacuolar protein sorting-associated protein 28 homolog | 1.55 |
|  | C080R135 | transcription initiation factor tfiid subunit 4 | 1.55 |
|  | C239R049 | sorting and assembly machinery component 50 homolog | 1.55 |
|  | C091R129 | small nuclear ribonucleoprotein sm d2 | 1.55 |
|  | C187R166 | mixed lineage kinase domain-like | 1.55 |
|  | C238R108 | hydroxyacylglutathione hydrolase | 1.55 |
|  | C089R095 | rna-binding protein musashi homolog 2 | 1.55 |
|  | C023R047 | calpain-2 catalytic subunit-like | 1.55 |
|  | C042R106 | serine incorporator 1 | 1.55 |
|  | C213R040 | protein njmu-r1 | 1.55 |
|  | C225R080 | translational activator of cytochrome c oxidase 1-like | 1.55 |
|  | C097R050 | epidermal retinol dehydrogenase 2-like | 1.55 |
|  | C082R029 | serine arginine-rich splicing factor 11-like | 1.55 |
|  | C040R016 | UNKNOWN | 1.55 |
|  | C062R096 | protein fam114a2-like | 1.55 |
|  | C225R010 | carboxypeptidase e | 1.55 |
|  | C048R080 | dual specificity protein phosphatase 22-a-like | 1.55 |
|  | C194R132 | n-acetylglucosamine-1-phosphotransferase subunit gamma precursor | 1.55 |
|  | C080R079 | cyclin-dependent kinase 4 inhibitor b | 1.55 |
|  | C196R038 | heterogeneous nuclear ribonucleoprotein h | 1.55 |
|  | C175R070 | ependymin precursor | 1.55 |
|  | C204R009 | uba-like domain-containing protein 1 | 1.55 |
|  | C243R047 | integral membrane protein 2b | 1.54 |
|  | C072R098 | profilin-2 | 1.54 |
|  | C005R042 | v-type proton atpase subunit f | 1.54 |
|  | C253R165 | mrna export factor | 1.54 |
|  | C263R125 | phytanoyl- hydroxylase-interacting protein | 1.54 |
|  | C221R019 | rna binding protein with multiple splicing 2 | 1.54 |
|  | C074R069 | gamma-glutamyl hydrolase | 1.54 |
|  | C111R100 | transcription factor vbp-like | 1.54 |
|  | C045R006 | ap-1 complex subunit sigma-3 | 1.54 |
|  | C104R072 | iron-responsive element-binding protein 2 | 1.54 |
|  | C086R157 | 28s ribosomal protein mitochondrial-like | 1.54 |
|  | C233R048 | mitochondrial-processing peptidase subunit beta-like | 1.54 |
|  | C170R037 | calcipressin-1 | 1.54 |
|  | C090R152 | 14-3-3 protein beta alpha-2 | 1.54 |
|  | C255R154 | 26s protease regulatory subunit 8 | 1.54 |
|  | C172R025 | gdp-mannose dehydratase | 1.54 |
|  | C027R097 | ependymin precursor | 1.54 |
|  | C163R032 | splicing arginine serine-rich 6 | 1.54 |
|  | C172R131 | testis derived transcript | 1.54 |
|  | C183R117 | elongation factor 2 | 1.54 |
|  | C171R007 | er membrane protein complex subunit 1-like | 1.54 |
|  | C187R136 | cobw domain-containing protein 2-like | 1.54 |
|  | C175R062 | probable phospholipid-transporting atpase iia-like | 1.53 |
|  | C215R130 | ras-related protein rab-25 | 1.53 |
|  | C116R019 | protein sco1 mitochondrial-like | 1.53 |
|  | C223R119 | eukaryotic translation initiation factor 3 subunit l-like | 1.53 |
|  | C226R129 | nad h dehydrogenase | 1.53 |
|  | C025R064 | atp-binding cassette sub-family e member 1 | 1.53 |
|  | C181R097 | transmembrane protein 199 | 1.53 |
|  | C047R127 | glycylpeptide n-tetradecanoyltransferase 1 | 1.53 |
|  | C085R130 | kunitz-type protease inhibitor 1-like | 1.53 |
|  | C069R075 | interleukin-13 receptor subunit alpha-2-like | 1.53 |
|  | C163R010 | ras-related protein rab-3b | 1.53 |
|  | C204R116 | ump-cmp kinase | 1.53 |
|  | C116R118 | septin-7 isoform 1 | 1.53 |
|  | C122R070 | inactive dipeptidyl peptidase 10-like | 1.53 |
|  | C109R041 | mitochondrial 2-oxoglutarate malate carrier protein | 1.53 |
|  | C083R037 | cbp p300-interacting transactivator 2 | 1.53 |
|  | C207R063 | UNKNOWN | 1.53 |
|  | C197R138 | mixed lineage kinase domain-like | 1.53 |
|  | C035R124 | alpha-mannosyl-glycoprotein 2-beta-n-acetylglucosaminyltransferase | 1.53 |
|  | C028R061 | transmembrane protein 68 | 1.53 |
|  | C015R093 | serine protease 27-like | 1.53 |
|  | C143R145 | methyl-binding domain protein 3 | 1.53 |
|  | C251R100 | myb sant-like dna-binding domain-containing protein 3-like isoform x1 | 1.53 |
|  | C230R122 | transmembrane protein 68 | 1.53 |
|  | C202R166 | nop58 protein | 1.53 |
|  | C006R127 | UNKNOWN | 1.53 |
|  | C058R091 | leucine-rich repeat-containing protein 58-like | 1.53 |
|  | C097R120 | ddb1- and cul4-associated factor 13 | 1.53 |
|  | C186R135 | actin-related protein 2 | 1.52 |
|  | C220R160 | dehydrogenase reductase sdr family member 13-like | 1.52 |
|  | C092R127 | UNKNOWN | 1.52 |
|  | C220R066 | sarcolemmal membrane-associated | 1.52 |
|  | C047R073 | phosphoribosyl pyrophosphate synthase-associated protein 1 | 1.52 |
|  | C151R128 | breakpoint cluster region protein | 1.52 |
|  | C154R036 | methyltransferase-like protein 5 | 1.52 |
|  | C012R158 | coiled-coil domain-containing protein 71 | 1.52 |
|  | C071R011 | lysosomal-associated transmembrane protein 4a | 1.52 |
|  | C138R054 | protein kish-a | 1.52 |
|  | C092R037 | endothelial differentiation-related factor 1 | 1.52 |
|  | C213R054 | cyclin-dependent kinase 4 inhibitor b | 1.52 |
|  | C179R155 | solute carrier family 31 (copper transporters) member 1 | 1.52 |
|  | C205R049 | biogenesis of lysosome-related organelles complex 1 subunit 1 | 1.52 |
|  | C146R012 | mitochondrial uncoupling protein 2 | 1.52 |
|  | C184R082 | regulator of g-protein signaling 20 | 1.52 |
|  | C198R104 | redox-regulatory protein fam213a | 1.52 |
|  | C071R039 | queuine trna-ribosyltransferase | 1.52 |
|  | C111R002 | integral membrane protein 2b | 1.51 |
|  | C075R068 | adp-ribosylation factor 4 | 1.51 |
|  | C264R102 | phosphoribosyl pyrophosphate synthase-associated protein 1 | 1.51 |
|  | C036R029 | charged multivesicular body protein 2b | 1.51 |
|  | C266R108 | plasma alpha-l-fucosidase | 1.51 |
|  | C015R122 | swi snf matrix actin dependent regulator of subfamily member 1 | 1.51 |
|  | C102R079 | e3 ubiquitin-protein ligase hectd1 | 1.51 |
|  | C065R130 | UNKNOWN | 1.51 |
|  | C122R120 | cd9 antigen | 1.51 |
|  | C148R073 | methyl- -binding domain protein 3 | 1.51 |
|  | C221R096 | s-methyl-5 -thioadenosine phosphorylase | 1.51 |
|  | C140R032 | palmitoyltransferase zdhhc15 | 1.51 |
|  | C195R134 | UNKNOWN | 1.51 |
|  | C096R108 | natterin-like protein | 1.51 |
|  | C217R164 | dna-binding protein inhibitor id-1 | 1.51 |
|  | C098R054 | anti-apoptotic protein nr13 | 1.51 |
|  | C102R125 | bcl2 adenovirus e1b 19 kda protein-interacting protein 3-like | 1.51 |
|  | C059R006 | collagen alpha-1 chain | 1.51 |
|  | C262R073 | cell cycle progression protein 1 | 1.51 |
|  | C155R142 | nedd4 family-interacting protein 1 | 1.51 |
|  | C022R008 | ubiquitin carboxyl-terminal hydrolase isozyme l1 | 1.51 |
|  | C036R076 | vacuolar protein sorting-associated protein 26b-like | 1.51 |
|  | C136R108 | homolog subfamily c member 12 | 1.51 |
|  | C155R148 | synaptic vesicle 2-related protein | 1.51 |
|  | C212R013 | septin 6 | 1.51 |
|  | C067R022 | activated rna polymerase ii transcriptional coactivator p15 | 1.51 |
|  | C215R082 | novel protein | 1.51 |
|  | C127R081 | UNKNOWN | 1.51 |
|  | C085R080 | UNKNOWN | 1.51 |
|  | C072R008 | tomoregulin-1-like | 1.51 |
|  | C068R110 | ectonucleoside triphosphate diphosphohydrolase 2 | 1.51 |
|  | C047R041 | zinc finger protein 2 homolog | 1.51 |
|  | C079R079 | zinc finger protein 830 | 1.51 |
|  | C019R054 | cathepsin l | 1.51 |
|  | C195R152 | scel protein | 1.51 |
|  | C132R086 | bola-like protein 3-like | 1.51 |
|  | C243R123 | heat shock protein 67b2 | 1.51 |
|  | C110R020 | v-type proton atpase subunit s1-like | 1.50 |
|  | C042R010 | cystathionine gamma-lyase | 1.50 |
|  | C216R067 | probable hydrolase pnkd-like | 1.50 |
|  | C176R098 | ewing sarcoma breakpoint region 1b | 1.50 |
|  | C067R034 | dynein heavy chain axonemal-like | 1.50 |
|  | C202R010 | guanine nucleotide-binding 3 | 1.50 |
|  | C141R031 | peptidyl-prolyl cis-trans isomerase h | 1.50 |
|  | C253R124 | stathmin-like 3 | 1.50 |
|  | C019R095 | daz-associated protein 2 | 1.50 |
|  | C052R105 | lupus la protein homolog | 1.50 |
|  | C005R114 | elmo domain-containing protein 2 | 1.50 |
|  | C031R158 | plasminogen activator inhibitor 1 rna-binding protein isoform 4 | 1.50 |
|  | C252R149 | nedd4 family-interacting protein 1 | 1.50 |
|  | C224R081 | protein fam98b | 1.50 |
|  | C143R111 | protein fam219b | 1.50 |
|  | C184R073 | lamina-associated polypeptide isoforms beta gamma | 1.50 |
|  | C201R007 | epidermal growth factor-like protein 6 | 1.50 |
|  | C215R081 | amphiphysin-like isoform x9 | 1.50 |
|  | C235R012 | protein max-like isoform 3 | 1.50 |
|  | C096R009 | ovarian cancer-associated gene 2 protein homolog | 1.50 |
|  | C064R008 | tetraspanin-9 | 1.50 |
|  | C213R081 | katnb1-like protein 1 | 1.49 |
|  | C065R149 | chibby homolog 1 | 1.49 |
|  | C062R081 | UNKNOWN | 1.49 |
|  | C097R051 | hiv tat-specific factor 1 homolog | 1.49 |
|  | C116R129 | protein manbal | 1.49 |
|  | C200R006 | bccip homolog | 1.49 |
|  | C081R001 | ets-related transcription factor elf-3 | 1.49 |
|  | C073R072 | mediator of rna polymerase ii transcription subunit 29 | 1.49 |
|  | C089R100 | n-terminal asparagine amidohydrolase | 1.49 |
|  | C161R159 | UNKNOWN | 1.49 |
|  | C172R087 | ras-related c3 botulinum toxin substrate 1 (rho small gtp binding protein rac1) | 1.49 |
|  | C237R136 | v-type proton atpase proteolipid subunit-like protein | 1.49 |
|  | C188R167 | UNKNOWN | 1.49 |
|  | C075R115 | zinc finger protein zic 5-like | 1.49 |
|  | C082R056 | jumping translocation breakpoint | 1.49 |
|  | C082R150 | heme transporter hrg1 | 1.49 |
|  | C028R137 | protein phosphatase catalytic gamma isoform | 1.49 |
|  | C091R023 | lanosterol 14-alpha demethylase-like | 1.49 |
|  | C095R105 | serine threonine-protein kinase pim-1-like | 1.49 |
|  | C097R083 | twisted gastrulation protein homolog 1 | 1.49 |
|  | C188R079 | coiled-coil domain-containing protein 23 | 1.48 |
|  | C188R140 | max-like protein x | 1.48 |
|  | C086R085 | chromobox protein homolog 1-like | 1.48 |
|  | C226R105 | coatomer subunit delta | 1.48 |
|  | C032R033 | proteasome subunit alpha type-1 | 1.48 |
|  | C069R112 | programmed cell death protein 10 | 1.48 |
|  | C092R122 | retinitis pigmentosa 9 protein homolog | 1.48 |
|  | C167R141 | actin-related protein 3 | 1.48 |
|  | C219R055 | scan domain-containing protein 3-like | 1.48 |
|  | C170R113 | nucleolin-like isoform 2 | 1.48 |
|  | C241R107 | zinc finger protein 2 homolog | 1.48 |
|  | C179R150 | homeobox protein dlx-2 | 1.48 |
|  | C089R053 | histone-binding protein rbbp4 | 1.48 |
|  | C202R022 | nfu1 iron-sulfur cluster scaffold mitochondrial | 1.48 |
|  | C055R051 | calmodulin | 1.48 |
|  | C060R144 | protein max-like isoform 3 | 1.48 |
|  | C210R072 | centrin-1 | 1.48 |
|  | C251R007 | tetraspanin-8 | 1.48 |
|  | C083R080 | transmembrane protein 9b | 1.48 |
|  | C228R097 | protein fam115-like | 1.48 |
|  | C173R086 | calumenin isoform 2 | 1.48 |
|  | C019R106 | cop9 signalosome complex subunit 6 | 1.48 |
|  | C092R167 | t-complex protein 1 subunit gamma | 1.48 |
|  | C051R159 | actin-related protein 3 | 1.47 |
|  | C191R103 | ldlr chaperone mesd | 1.47 |
|  | C207R079 | hcls1-associated protein x-1 | 1.47 |
|  | C069R079 | transmembrane emp24 domain-containing protein 4 | 1.47 |
|  | C113R072 | chemokine-like receptor 1-like | 1.47 |
|  | C143R165 | annexin a2-a | 1.47 |
|  | C256R136 | eukaryotic translation initiation factor 3 subunit j | 1.47 |
|  | C090R167 | growth arrest and dna damage-inducible protein gadd45 beta | 1.47 |
|  | C210R055 | nedd4 family-interacting protein 1 | 1.47 |
|  | C087R106 | mitochondrial mimitin | 1.47 |
|  | C179R005 | v-type proton atpase proteolipid subunit-like protein | 1.47 |
|  | C061R101 | eukaryotic translation initiation factor 3 subunit k | 1.47 |
|  | C224R065 | envoplakin-like | 1.47 |
|  | C025R133 | UNKNOWN | 1.47 |
|  | C186R109 | cyclin-dependent kinase inhibitor 1c | 1.47 |
|  | C197R083 | UNKNOWN | 1.47 |
|  | C015R078 | translation initiation factor eif-2b subunit gamma-like | 1.47 |
|  | C081R124 | cysteine and glycine-rich protein 2 | 1.47 |
|  | C091R071 | beta-catenin-like protein 1 | 1.47 |
|  | C060R016 | programmed cell death protein 10 | 1.47 |
|  | C258R141 | UNKNOWN | 1.47 |
|  | C057R159 | glutathione s-transferase a | 1.47 |
|  | C062R006 | cystathionine gamma-lyase | 1.47 |
|  | C106R071 | myosin regulatory light chain smooth muscle isoform | 1.47 |
|  | C118R143 | tubulin-specific chaperone a | 1.46 |
|  | C259R074 | casein kinase ii subunit beta | 1.46 |
|  | C086R121 | 40s ribosomal protein s5 | 1.46 |
|  | C231R024 | cat eye syndrome critical region protein 5 homolog | 1.46 |
|  | C153R119 | ras-related protein rab-27a | 1.46 |
|  | C024R036 | proteasome assembly chaperone 1 | 1.46 |
|  | C048R096 | protein set-like | 1.46 |
|  | C064R020 | bromodomain-containing protein 3 | 1.46 |
|  | C041R026 | mitotic-spindle organizing protein 1 | 1.46 |
|  | C216R105 | glutathione s-transferase theta-1 | 1.46 |
|  | C112R055 | 2-oxoglutarate and iron-dependent oxygenase domain-containing protein 1 | 1.46 |
|  | C171R095 | phosphoserine phosphatase | 1.46 |
|  | C138R129 | cathepsin l | 1.46 |
|  | C091R101 | cysteine-rich protein 2 | 1.46 |
|  | C082R155 | tm2 domain-containing protein 2 | 1.46 |
|  | C264R036 | unknown | 1.46 |
|  | C174R009 | UNKNOWN | 1.46 |
|  | C089R127 | adipocyte plasma membrane-associated protein | 1.46 |
|  | C091R108 | histone -like | 1.46 |
|  | C114R058 | phosphate carrier mitochondrial precursor | 1.46 |
|  | C114R138 | methyltransferase-like protein 6 | 1.46 |
|  | C167R045 | claudin domain-containing protein 1-like | 1.46 |
|  | C162R095 | microfibrillar-associated protein 1 | 1.46 |
|  | C160R127 | beta-hexosaminidase subunit beta-like | 1.46 |
|  | C103R127 | protein btg1 | 1.46 |
|  | C071R153 | UNKNOWN | 1.46 |
|  | C022R039 | low quality protein: methionine-r-sulfoxide reductase b1 | 1.46 |
|  | C098R033 | homolog subfamily c member 12 | 1.45 |
|  | C222R095 | t-complex protein 1 subunit alpha | 1.45 |
|  | C059R036 | heat shock cognate 70 kda protein | 1.45 |
|  | C138R068 | small g protein signaling modulator 1-like | 1.45 |
|  | C104R085 | calpain-2 catalytic subunit-like | 1.45 |
|  | C088R129 | adp-ribosylation factor-like protein 2-binding protein | 1.45 |
|  | C058R147 | calpain-9 isoform 3 | 1.45 |
|  | C190R117 | transmembrane and tpr repeat-containing protein 4 | 1.45 |
|  | C193R137 | UNKNOWN | 1.45 |
|  | C127R065 | ap-1 complex subunit mu-2 | 1.45 |
|  | C185R119 | raf proto-oncogene serine threonine-protein kinase | 1.45 |
|  | C028R084 | guanine nucleotide-binding protein g subunit alpha-1 | 1.45 |
|  | C242R107 | rrna-processing protein utp23 homolog | 1.45 |
|  | C174R154 | serine incorporator 1 | 1.45 |
|  | C019R081 | programmed cell death protein 10 | 1.45 |
|  | C059R058 | mortality factor 4-like protein 1 | 1.45 |
|  | C229R066 | UNKNOWN | 1.45 |
|  | C019R159 | dna-binding protein inhibitor id-1 | 1.45 |
|  | C220R142 | UNKNOWN | 1.45 |
|  | C134R093 | src substrate protein p85-like | 1.44 |
|  | C199R116 | serine threonine-protein phosphatase 2a catalytic subunit alpha isoform | 1.44 |
|  | C215R162 | protein set | 1.44 |
|  | C088R157 | secretory carrier-associated membrane protein 4 | 1.44 |
|  | C118R132 | cyclin i | 1.44 |
|  | C175R058 | pax-6 protein | 1.44 |
|  | C247R077 | calmodulin | 1.44 |
|  | C185R068 | x-box binding protein 1 | 1.44 |
|  | C142R084 | peroxiredoxin-1 | 1.44 |
|  | C082R117 | transmembrane protein 109-like | 1.44 |
|  | C140R046 | fun14 domain-containing protein 2 | 1.44 |
|  | C070R045 | beta-2-microglobulin precursor | 1.44 |
|  | C007R108 | neurogenic differentiation factor 2-like | 1.44 |
|  | C225R104 | UNKNOWN | 1.44 |
|  | C180R156 | protein pelota homolog | 1.44 |
|  | C253R038 | lamin-b receptor | 1.44 |
|  | C153R044 | interferon-induced guanylate-binding protein 1 | 1.44 |
|  | C122R036 | actin-related protein 2 3 complex subunit 1a | 1.44 |
|  | C135R080 | UNKNOWN | 1.44 |
|  | C244R024 | argininosuccinate synthase | 1.44 |
|  | C184R017 | nucleolar gtp-binding protein 1 | 1.44 |
|  | C169R036 | choline transporter-like protein 2-like | 1.44 |
|  | C154R142 | histone | 1.43 |
|  | C194R105 | actin-related protein 2 3 complex subunit 3 | 1.43 |
|  | C075R117 | trafficking protein particle complex subunit 3 | 1.43 |
|  | C203R166 | aurora kinase b | 1.43 |
|  | C073R148 | c-type natriuretic peptide 1 precursor | 1.43 |
|  | C134R102 | retinaldehyde-binding protein 1-like | 1.43 |
|  | C035R031 | protein fam219a isoform x1 | 1.43 |
|  | C058R160 | cystatin precursor | 1.43 |
|  | C100R124 | transmembrane protein 50a | 1.43 |
|  | C079R086 | UNKNOWN | 1.43 |
|  | C234R079 | tight junction protein zo-2-like | 1.43 |
|  | C042R014 | signal peptidase complex catalytic subunit sec11a | 1.43 |
|  | C212R170 | prepro-thyrotropin-releasing hormone | 1.43 |
|  | C225R159 | 14-3-3 protein zeta delta | 1.43 |
|  | C023R008 | UNKNOWN | 1.43 |
|  | C251R024 | sialic acid synthase | 1.43 |
|  | C057R083 | UNKNOWN | 1.43 |
|  | C053R020 | dual specificity protein kinase clk4 | 1.43 |
|  | C106R069 | neuroendocrine protein 7b2 | 1.43 |
|  | C190R015 | chromosome 6 open reading frame 106 | 1.42 |
|  | C092R070 | mitochondrial-processing peptidase subunit beta-like | 1.42 |
|  | C129R086 | dolichyl-diphosphooligosaccharide-protein glycosyltransferase 48 kda subunit-like | 1.42 |
|  | C243R008 | homeobox protein otx2 | 1.42 |
|  | C170R110 | krueppel-like factor 6 | 1.42 |
|  | C063R129 | translationally-controlled tumor protein | 1.42 |
|  | C138R146 | scinderin like a | 1.42 |
|  | C189R035 | n-lysine methyltransferase setd8-a-like | 1.42 |
|  | C150R072 | purine nucleoside phosphorylase-like | 1.42 |
|  | C113R163 | riboflavin-binding protein | 1.42 |
|  | C039R103 | UNKNOWN | 1.42 |
|  | C136R077 | probable ergosterol biosynthetic protein 28 | 1.42 |
|  | C089R033 | sideroflexin 2 | 1.42 |
|  | C140R121 | chromobox protein homolog 1 | 1.42 |
|  | C063R126 | glutathione s-transferase a | 1.42 |
|  | C226R110 | paired box protein pax-6 isoform 2 | 1.41 |
|  | C148R048 | glycerophosphodiester phosphodiesterase domain-containing protein 1 | 1.41 |
|  | C117R097 | dna-binding protein inhibitor id-1 | 1.41 |
|  | C182R097 | translation initiation factor eif-2b subunit alpha | 1.41 |
|  | C096R026 | transmembrane protein 60 | 1.41 |
|  | C059R055 | isovaleryl coenzyme a dehydrogenase | 1.41 |
|  | C220R120 | transmembrane protein 9b | 1.41 |
|  | C174R079 | splicing arginine serine-rich 2 | 1.41 |
|  | C255R032 | occludin b | 1.41 |
|  | C265R107 | UNKNOWN | 1.41 |
|  | C042R019 | tumor necrosis factor receptor superfamily member 1a precursor | 1.41 |
|  | C209R092 | androgen-dependent tfpi-regulating protein | 1.41 |
|  | C190R097 | er lumen protein retaining receptor 3 | 1.41 |
|  | C144R053 | vacuolar protein sorting-associated protein 11 homolog | 1.41 |
|  | C059R048 | n-acetylgalactosaminyltransferase 7-like | 1.41 |
|  | C103R116 | transmembrane emp24 domain-containing protein 4 | 1.41 |
|  | C116R008 | adenylate kinase isoenzyme 6-like | 1.41 |
|  | C035R109 | 28s ribosomal protein mitochondrial-like | 1.41 |
|  | C204R099 | annexin a11 | 1.41 |
|  | C158R156 | gtpase imap family member 7-like | 1.41 |
|  | C057R094 | endothelial differentiation-related factor 1 | 1.41 |
|  | C256R148 | UNKNOWN | 1.40 |
|  | C151R170 | beta- -galactosyltransferase 1 | 1.40 |
|  | C146R057 | rhombotin-1 | 1.40 |
|  | C245R110 | UNKNOWN | 1.40 |
|  | C175R165 | 15-hydroxyprostaglandin dehydrogenase | 1.40 |
|  | C140R114 | 116 kda u5 small nuclear ribonucleoprotein component | 1.40 |
|  | C098R169 | cyclin-d1-binding protein 1 homolog | 1.40 |
|  | C180R149 | lim zinc-binding domain-containing nebulette isoform 2 | 1.40 |
|  | C148R105 | rab gtpase-binding effector protein 2 | 1.40 |
|  | C200R092 | ras gtpase-activating protein-binding protein 1-like | 1.40 |
|  | C069R096 | protein cwc15 homolog | 1.40 |
|  | C043R093 | phosphatidylinositol n-acetylglucosaminyltransferase subunit p | 1.40 |
|  | C192R069 | nuclear receptor subfamily 1 group d member 2-like | 1.40 |
|  | C190R017 | trna selenocysteine 1-associated protein 1-like | 1.40 |
|  | C220R161 | malonyl-acyl carrier protein mitochondrial | 1.39 |
|  | C213R085 | poly polymerase 12 | 1.39 |
|  | C229R107 | ubiquitin-conjugating enzyme e2 d4 | 1.39 |
|  | C090R155 | sialic acid synthase | 1.39 |
|  | C169R158 | rna 3 -terminal phosphate cyclase | 1.39 |
|  | C042R044 | cadherin type n-cadherin protein | 1.39 |
|  | C095R068 | phosphoglycerate mutase 1 | 1.39 |
|  | C170R155 | atlastin-3 | 1.39 |
|  | C092R091 | kinesin-associated protein 3 | 1.39 |
|  | C014R064 | x-box binding protein 1 | 1.39 |
|  | C187R068 | splicing arginine serine-rich 2 | 1.39 |
|  | C191R091 | nucleolar rna helicase 2-like | 1.39 |
|  | C170R019 | apoptotic protease-activating factor 1-like | 1.39 |
|  | C095R091 | gpn-loop gtpase 1-like | 1.39 |
|  | C171R036 | er lumen protein retaining receptor 2 | 1.38 |
|  | C205R046 | nucleolar gtp-binding protein 1 | 1.38 |
|  | C067R108 | tubulin-specific chaperone e | 1.38 |
|  | C144R073 | cathepsin z-like | 1.38 |
|  | C078R012 | 4f2 cell-surface antigen heavy chain | 1.38 |
|  | C142R057 | riboflavin transporter 2-like | 1.38 |
|  | C178R013 | hydroxymethylglutaryl- mitochondrial-like | 1.38 |
|  | C189R069 | lysyl-trna synthetase | 1.38 |
|  | C254R162 | splicing arginine serine-rich 3 | 1.38 |
|  | C251R052 | calmodulin | 1.38 |
|  | C033R014 | retinol dehydrogenase 14-like | 1.37 |
|  | C178R139 | aspartate mitochondrial-like | 1.37 |
|  | C264R046 | h aca ribonucleoprotein complex subunit 3 | 1.37 |
|  | C094R064 | comm domain-containing protein 7 | 1.37 |
|  | C182R063 | translocon-associated protein subunit delta precursor | 1.37 |
|  | C229R058 | transmembrane protein 93 | 1.37 |
|  | C093R009 | 26s protease regulatory subunit 6b isoform 1 | 1.37 |
|  | C204R126 | pleiotrophic factor-alpha-2 precursor | 1.37 |
|  | C170R065 | myelin transcription factor 1-like | 1.36 |
|  | C074R091 | UNKNOWN | 1.36 |
|  | C072R069 | transmembrane emp24 domain-containing protein 10 precursor | 1.36 |
|  | C168R086 | uv excision repair protein rad23 homolog a isoform 1 | 1.36 |
|  | C078R065 | adenosine deaminase | 1.36 |
|  | C191R108 | er lumen protein retaining receptor 3 | 1.36 |
|  | C068R020 | calcium calmodulin-dependent protein kinase ii inhibitor 2 | 1.36 |
|  | C084R112 | alpha-aspartyl dipeptidase | 1.36 |
|  | C056R016 | glutathione s-transferase theta-1 | 1.36 |
|  | C083R152 | UNKNOWN | 1.36 |
|  | C070R142 | serine threonine-protein kinase sgk1-like | 1.36 |
|  | C080R072 | armadillo repeat-containing protein 6-like | 1.36 |
|  | C179R162 | dual specificity protein phosphatase 14 | 1.36 |
|  | C097R093 | u3 small nucleolar rna-interacting protein 2 | 1.36 |
|  | C089R075 | ran-binding protein 3-like | 1.36 |
|  | C231R097 | transcription initiation factor tfiid subunit 10 | 1.35 |
|  | C045R105 | cytochrome b-c1 complex subunit 10 | 1.35 |
|  | C087R045 | sjchgc03036 protein | 1.35 |
|  | C238R054 | leucine-rich glioma-inactivated protein 1 precursor | 1.35 |
|  | C027R100 | comm domain-containing protein 3 | 1.35 |
|  | C090R049 | dehydrogenase reductase sdr family member 11 | 1.35 |
|  | C088R087 | zgc:174935 protein | 1.35 |
|  | C095R071 | protein kish-b | 1.35 |
|  | C218R136 | retinol dehydrogenase 3 | 1.34 |
|  | C201R094 | protein fam134a-like | 1.34 |
|  | C027R045 | UNKNOWN | 1.34 |
|  | C097R066 | myotubularin-related protein 7 | 1.34 |
|  | C155R123 | x-box binding protein 1 | 1.34 |
|  | C189R102 | eukaryotic translation initiation factor 3 subunit 6 | 1.34 |
|  | C171R053 | mam domain-containing protein 2 | 1.33 |
|  | C257R024 | ezrin isoform 2 | 1.33 |
|  | C197R096 | cop9 signalosome complex subunit 5 | 1.33 |
|  | C114R052 | voltage-dependent anion-selective channel protein 2-like | 1.33 |
|  | C099R073 | zinc finger protein 185 isoform 6 | 1.32 |
|  | C076R007 | coiled-coil domain-containing protein 94 | 1.32 |
|  | C202R165 | transcription factor | 1.32 |
|  | C189R140 | glutathione s-transferase theta-1 | 1.32 |
|  | C073R056 | phytanoyl- hydroxylase-interacting protein | 1.32 |
|  | C138R100 | UNKNOWN | 1.32 |
|  | C234R085 | e3 ubiquitin-protein ligase arih1-like | 1.30 |
|  | C150R026 | transposable element tcb2 transposase | 1.29 |
|  | C091R091 | eukaryotic translation initiation factor 3 subunit j | 1.25 |
|  | C043R105 | proactivator polypeptide precursor | 0.80 |
|  | C078R114 | low density lipoprotein receptor adapter protein 1 | 0.79 |
|  | C073R068 | ss18-like protein 2 | 0.78 |
|  | C001R059 | glycogen muscle form | 0.78 |
|  | C121R042 | xaa-pro aminopeptidase 1 | 0.78 |
|  | C234R122 | hig1 domain family member 2a | 0.78 |
|  | C231R083 | nedd4 family-interacting protein 2 | 0.78 |
|  | C059R084 | transmembrane protein 182 | 0.78 |
|  | C045R038 | beta-parvin-like isoform x1 | 0.78 |
|  | C124R077 | kinase d-interacting substrate of 220 kda-like | 0.77 |
|  | C097R154 | g kinase-anchoring protein 1 | 0.77 |
|  | C181R144 | general transcription factor 3c polypeptide 6 | 0.77 |
|  | C164R046 | amyotrophic lateral sclerosis 2 chromosomal region candidate gene 4 protein | 0.77 |
|  | C153R142 | branched-chain-amino-acid cytosolic-like | 0.77 |
|  | C152R101 | cytochrome b-c1 complex subunit mitochondrial-like | 0.77 |
|  | C015R047 | gdp-l-fucose synthetase | 0.77 |
|  | C139R083 | UNKNOWN | 0.77 |
|  | C001R052 | cap-gly domain-containing linker protein 1 | 0.77 |
|  | C155R106 | ubiquitin conjugation factor e4 a | 0.77 |
|  | C046R069 | sjchgc03018 protein | 0.77 |
|  | C101R045 | UNKNOWN | 0.76 |
|  | C130R104 | transcription factor 20 isoform 2 | 0.76 |
|  | C259R025 | mrna turnover protein 4 homolog | 0.76 |
|  | C241R069 | interleukin-1 receptor-associated kinase 3 | 0.76 |
|  | C233R124 | dnaj homolog subfamily c member 15 | 0.76 |
|  | C253R055 | 26s protease regulatory subunit 8 | 0.76 |
|  | C152R165 | heterogeneous nuclear ribonucleoprotein k | 0.76 |
|  | C229R153 | endoplasmin precursor | 0.76 |
|  | C154R098 | UNKNOWN | 0.76 |
|  | C121R027 | ribosomal protein s6 kinase beta-1-like | 0.76 |
|  | C084R049 | UNKNOWN | 0.76 |
|  | C130R070 | matrin-3 | 0.76 |
|  | C192R128 | allograft inflammatory factor 1 | 0.76 |
|  | C095R021 | c-c motif chemokine 20-like | 0.76 |
|  | C082R028 | UNKNOWN | 0.76 |
|  | C232R041 | UNKNOWN | 0.76 |
|  | C131R116 | phosphorylase b kinase regulatory subunit beta-like | 0.76 |
|  | C190R116 | deoxyhypusine hydroxylase | 0.75 |
|  | C030R035 | coiled-coil-helix-coiled-coil-helix domain containing 2 variant 1 | 0.75 |
|  | C252R092 | UNKNOWN | 0.75 |
|  | C045R100 | na k atpase alpha subunit isoform 1c | 0.75 |
|  | C014R130 | UNKNOWN | 0.75 |
|  | C016R052 | casein kinase i isoform delta | 0.75 |
|  | C026R129 | hypoxia-inducible factor 1 alpha | 0.75 |
|  | C219R075 | atpase mitochondrial precursor | 0.75 |
|  | C170R082 | zinc finger protein 62 homolog | 0.75 |
|  | C126R089 | UNKNOWN | 0.75 |
|  | C083R028 | ets translocation variant 4 | 0.75 |
|  | C143R140 | UNKNOWN | 0.75 |
|  | C059R097 | UNKNOWN | 0.75 |
|  | C037R080 | UNKNOWN | 0.75 |
|  | C219R071 | ubiquitin-conjugating enzyme e2 d2 | 0.75 |
|  | C133R012 | cat eye syndrome critical region protein 5 homolog | 0.75 |
|  | C173R100 | UNKNOWN | 0.75 |
|  | C064R082 | UNKNOWN | 0.75 |
|  | C158R131 | interferon-induced guanylate-binding protein 1 | 0.75 |
|  | C051R012 | transposable element tcb1 transposase | 0.75 |
|  | C131R041 | cell differentiation protein rcd1 homolog | 0.75 |
|  | C229R088 | cdgsh iron-sulfur domain-containing protein 2 | 0.75 |
|  | C103R108 | coiled-coil domain-containing protein 51-like | 0.75 |
|  | C164R086 | tether containing ubx domain for glut4 | 0.75 |
|  | C211R107 | peptidyl-trna hydrolase mitochondrial | 0.75 |
|  | C117R098 | sphingomyelin phosphodiesterase 4 | 0.75 |
|  | C096R068 | succinyl- ligase | 0.75 |
|  | C040R115 | transmembrane protein 237a-like | 0.75 |
|  | C155R071 | thrombospondin 2 | 0.74 |
|  | C074R096 | chromosome-associated kinesin kif4a | 0.74 |
|  | C182R067 | transcriptional repressor ctcf-like | 0.74 |
|  | C143R106 | swi snf-related matrix-associated actin-dependent regulator of chromatin subfamily a containing dead h box 1 isoform 2 | 0.74 |
|  | C107R039 | 39s ribosomal protein mitochondrial precursor | 0.74 |
|  | C116R045 | hypoxia up-regulated protein 1-like | 0.74 |
|  | C053R015 | UNKNOWN | 0.74 |
|  | C094R017 | tfiih basal transcription factor complex helicase xpd subunit isoform x1 | 0.74 |
|  | C160R059 | mitogen-activated protein kinase kinase kinase kinase 3-like isoform 2 | 0.74 |
|  | C092R036 | protein-tyrosine sulfotransferase 1 | 0.74 |
|  | C154R121 | rna-binding protein 4b | 0.74 |
|  | C013R059 | macrophage-capping protein | 0.74 |
|  | C112R095 | UNKNOWN | 0.74 |
|  | C112R096 | dna topoisomerase 1 | 0.74 |
|  | C112R077 | camp-dependent protein kinase type ii-alpha regulatory subunit-like | 0.74 |
|  | C112R070 | intron-binding protein aquarius | 0.74 |
|  | C011R098 | tropomodulin-4 | 0.74 |
|  | C200R141 | tpm1 protein | 0.74 |
|  | C053R019 | UNKNOWN | 0.74 |
|  | C161R096 | nuclear pore complex protein nup160 | 0.74 |
|  | C201R084 | membrane-spanning 4-domains subfamily a member 4a | 0.74 |
|  | C111R069 | protoheme ix mitochondrial-like | 0.74 |
|  | C177R068 | mannose-6-phosphate isomerase-like | 0.74 |
|  | C072R039 | UNKNOWN | 0.74 |
|  | C148R072 | UNKNOWN | 0.74 |
|  | C070R109 | UNKNOWN | 0.74 |
|  | C228R094 | UNKNOWN | 0.74 |
|  | C157R093 | peptidyl-trna hydrolase mitochondrial precursor | 0.74 |
|  | C266R131 | myeloid differentiation factor 88 | 0.74 |
|  | C166R036 | UNKNOWN | 0.74 |
|  | C017R121 | transmembrane protein 134-like | 0.74 |
|  | C175R139 | cation-independent mannose-6-phosphate receptor | 0.74 |
|  | C121R043 | neurofilament light polypeptide-like | 0.74 |
|  | C186R097 | ctp synthase 1-like | 0.74 |
|  | C159R019 | sororin | 0.74 |
|  | C063R011 | pls3 protein | 0.74 |
|  | C035R142 | cleft lip and palate transmembrane protein 1-like protein | 0.74 |
|  | C125R130 | endothelin-converting enzyme 2-like | 0.74 |
|  | C140R017 | major facilitator superfamily domain-containing protein 5 | 0.74 |
|  | C173R150 | UNKNOWN | 0.74 |
|  | C250R054 | gdp dissociation inhibitor 2 | 0.74 |
|  | C214R162 | kin of irre-like protein 1-like | 0.74 |
|  | C016R164 | UNKNOWN | 0.74 |
|  | C028R058 | ubiquitin-conjugating enzyme e2 d2 | 0.73 |
|  | C197R076 | UNKNOWN | 0.73 |
|  | C078R022 | cellular nucleic acid-binding protein | 0.73 |
|  | C133R136 | protein yipf5 | 0.73 |
|  | C145R083 | UNKNOWN | 0.73 |
|  | C108R029 | UNKNOWN | 0.73 |
|  | C169R045 | protein fam212a-like | 0.73 |
|  | C157R110 | protein kish-a | 0.73 |
|  | C191R124 | developmentally-regulated gtp-binding protein 1 | 0.73 |
|  | C174R155 | 26s proteasome non-atpase regulatory subunit 3 | 0.73 |
|  | C180R140 | UNKNOWN | 0.73 |
|  | C154R024 | UNKNOWN | 0.73 |
|  | C209R139 | homocysteine-responsive endoplasmic reticulum-resident ubiquitin-like domain member 2 | 0.73 |
|  | C060R042 | arginyl-trna-protein transferase 1 | 0.73 |
|  | C157R127 | cytochrome c oxidase subunit mitochondrial precursor | 0.73 |
|  | C229R033 | exosome complex exonuclease rrp44 | 0.73 |
|  | C014R125 | ubiquitin-conjugating enzyme e2 g1 | 0.73 |
|  | C207R072 | uridine 5 -monophosphate synthase | 0.73 |
|  | C188R115 | UNKNOWN | 0.73 |
|  | C040R121 | neurochondrin isoform 1 | 0.73 |
|  | C139R127 | UNKNOWN | 0.73 |
|  | C119R095 | UNKNOWN | 0.73 |
|  | C075R082 | UNKNOWN | 0.73 |
|  | C098R046 | UNKNOWN | 0.73 |
|  | C104R078 | cleavage stimulation factor subunit 3 | 0.73 |
|  | C184R101 | UNKNOWN | 0.73 |
|  | C034R074 | oocyte zinc finger protein 20 | 0.73 |
|  | C100R094 | ring finger protein 170 | 0.73 |
|  | C097R085 | transmembrane protein 39b | 0.73 |
|  | C010R104 | gamma b (breast cancer-specific protein 1) | 0.73 |
|  | C240R060 | component of gems 4 | 0.73 |
|  | C007R133 | survival motor neuron protein 1 | 0.73 |
|  | C140R088 | lysosomal thioesterase ppt2-a-like | 0.73 |
|  | C245R100 | fructose -bisphosphatase 1-like | 0.73 |
|  | C178R061 | rna-binding protein musashi homolog 2 isoform 1 | 0.73 |
|  | C129R010 | ribosomal protein partial | 0.73 |
|  | C111R160 | slit-robo rho gtpase-activating protein 3-like isoform x2 | 0.73 |
|  | C074R125 | e3 ubiquitin-protein ligase march6 | 0.73 |
|  | C157R166 | sodium potassium-transporting atpase subunit beta-233 | 0.73 |
|  | C075R063 | bmp-binding endothelial regulator protein | 0.73 |
|  | C031R117 | nuclear receptor coactivator 6-like | 0.73 |
|  | C160R107 | vasculin-like protein 1 | 0.73 |
|  | C229R126 | regulation of nuclear pre-mrna domain-containing protein 1b | 0.73 |
|  | C144R068 | ran-specific gtpase-activating protein | 0.73 |
|  | C043R081 | cullin-3 isoform 2 | 0.72 |
|  | C215R079 | calpain-3 isoform 3 | 0.72 |
|  | C157R158 | UNKNOWN | 0.72 |
|  | C075R079 | splicing factor 3a subunit 1 isoform 1 | 0.72 |
|  | C131R090 | rho guanine nucleotide exchange factor 15-like | 0.72 |
|  | C109R093 | transposase | 0.72 |
|  | C101R107 | h aca ribonucleoprotein complex subunit 1 | 0.72 |
|  | C075R021 | phytanoyl- dioxygenase domain-containing protein 1 | 0.72 |
|  | C151R058 | v-set and transmembrane domain-containing protein 2-like | 0.72 |
|  | C030R012 | UNKNOWN | 0.72 |
|  | C229R019 | eukaryotic translation initiation factor 2 subunit 1 | 0.72 |
|  | C040R043 | UNKNOWN | 0.72 |
|  | C113R032 | UNKNOWN | 0.72 |
|  | C160R104 | UNKNOWN | 0.72 |
|  | C240R072 | replication protein a 14 kda subunit | 0.72 |
|  | C205R156 | UNKNOWN | 0.72 |
|  | C252R074 | targeting protein for xklp2 | 0.72 |
|  | C171R089 | UNKNOWN | 0.72 |
|  | C172R098 | lysyl-trna synthetase | 0.72 |
|  | C255R123 | UNKNOWN | 0.72 |
|  | C135R134 | dedicator of cytokinesis protein 2 | 0.72 |
|  | C171R059 | UNKNOWN | 0.72 |
|  | C081R147 | UNKNOWN | 0.72 |
|  | C081R106 | nadh dehydrogenase | 0.72 |
|  | C133R044 | UNKNOWN | 0.72 |
|  | C167R022 | atp-binding cassette sub-family d member 3 isoform 2 | 0.72 |
|  | C108R010 | e3 ubiquitin-protein ligase bre1a | 0.72 |
|  | C172R142 | g-protein-signaling modulator 2 | 0.72 |
|  | C106R056 | triadin | 0.72 |
|  | C126R044 | UNKNOWN | 0.72 |
|  | C234R087 | myocyte-specific enhancer factor 2c-like isoform 2 | 0.72 |
|  | C182R121 | coagulation factor xiii a chain | 0.72 |
|  | C074R104 | cytochrome c oxidase subunit vib isoform 1 | 0.72 |
|  | C111R056 | cytochrome c oxidase subunit vib isoform 1 | 0.72 |
|  | C065R065 | UNKNOWN | 0.72 |
|  | C127R128 | b-cell cll lymphoma 9 protein | 0.72 |
|  | C232R114 | translation initiation factor eif-2b subunit delta-like | 0.72 |
|  | C200R100 | tropomyosin 4 | 0.72 |
|  | C236R151 | UNKNOWN | 0.72 |
|  | C221R094 | UNKNOWN | 0.72 |
|  | C168R120 | polyhomeotic-like protein 1-like | 0.72 |
|  | C013R122 | UNKNOWN | 0.72 |
|  | C103R132 | keratinocyte-associated transmembrane protein 2-like | 0.72 |
|  | C075R073 | UNKNOWN | 0.72 |
|  | C015R138 | signal recognition particle 72 kda protein | 0.72 |
|  | C150R071 | protein kinase c beta type-like | 0.72 |
|  | C118R086 | nad mitochondrial-like | 0.72 |
|  | C230R126 | proteasome subunit alpha type-6 | 0.72 |
|  | C123R079 | UNKNOWN | 0.72 |
|  | C252R069 | laminin subunit alpha-2 | 0.71 |
|  | C229R119 | protection of telomeres protein 1 | 0.71 |
|  | C214R165 | cytochrome b | 0.71 |
|  | C003R097 | focal adhesion kinase partial | 0.71 |
|  | C121R065 | transmembrane protein 97 | 0.71 |
|  | C196R081 | rna-binding protein 40 | 0.71 |
|  | C117R126 | UNKNOWN | 0.71 |
|  | C260R141 | pantophysin | 0.71 |
|  | C053R033 | related to transposase | 0.71 |
|  | C149R117 | UNKNOWN | 0.71 |
|  | C229R065 | squalene synthase | 0.71 |
|  | C019R143 | target of egr1 protein 1 | 0.71 |
|  | C177R120 | t-complex protein 1 subunit gamma isoform 1 | 0.71 |
|  | C231R113 | ribosomal rna small subunit methyltransferase nep1-like | 0.71 |
|  | C162R114 | cell division cycle protein 27 homolog | 0.71 |
|  | C097R137 | protein fam167a-like | 0.71 |
|  | C171R066 | transmembrane protein c7orf23 homolog | 0.71 |
|  | C155R025 | cell adhesion molecule 4-like | 0.71 |
|  | C120R045 | UNKNOWN | 0.71 |
|  | C229R070 | UNKNOWN | 0.71 |
|  | C046R169 | integrin-linked protein kinase | 0.71 |
|  | C130R088 | protein zwilch homolog | 0.71 |
|  | C015R042 | proteasome maturation protein | 0.71 |
|  | C125R027 | thioredoxin-like protein 1 | 0.71 |
|  | C144R089 | upf0609 protein c4orf27 homolog | 0.71 |
|  | C169R048 | heat shock cognate 71 kda | 0.71 |
|  | C204R088 | PREDICTED: uncharacterized protein C9orf152-like | 0.71 |
|  | C141R018 | UNKNOWN | 0.71 |
|  | C259R042 | eukaryotic translation initiation factor 4e type 2 | 0.71 |
|  | C229R035 | leucine-rich repeat flightless-interacting protein 2 | 0.71 |
|  | C063R095 | c-x-c motif chemokine 10 precursor | 0.71 |
|  | C162R158 | 26s proteasome non-atpase regulatory subunit 10 | 0.71 |
|  | C044R022 | protein fem-1 homolog c-like | 0.71 |
|  | C046R009 | dermatan 4 sulfotransferase 1 | 0.71 |
|  | C139R096 | cdk5 regulatory subunit associated protein 1-like 1 | 0.71 |
|  | C149R127 | tropomyosin alpha-1 chain isoform 4 | 0.71 |
|  | C165R058 | UNKNOWN | 0.71 |
|  | C068R133 | homeobox protein otx5-like | 0.71 |
|  | C230R035 | UNKNOWN | 0.71 |
|  | C164R011 | origin recognition complex subunit 3-like | 0.71 |
|  | C066R062 | carboxy-terminal domain rna polymerase ii polypeptide a small phosphatase 1-like | 0.71 |
|  | C007R091 | arf-gap with sh3 ank repeat and ph domain-containing protein 2-like | 0.71 |
|  | C199R146 | UNKNOWN | 0.71 |
|  | C203R124 | histone-lysine n-methyltransferase ezh2-like | 0.71 |
|  | C260R080 | ubiquitin-protein ligase e3a-like | 0.71 |
|  | C244R115 | UNKNOWN | 0.71 |
|  | C112R112 | UNKNOWN | 0.71 |
|  | C092R009 | low quality protein: dedicator of cytokinesis protein 10-like | 0.71 |
|  | C255R047 | origin recognition complex subunit 5 | 0.71 |
|  | C236R033 | UNKNOWN | 0.71 |
|  | C127R087 | twinfilin-1 | 0.71 |
|  | C109R024 | sjchgc04011 protein | 0.71 |
|  | C059R016 | transmembrane protein 129 | 0.71 |
|  | C089R139 | usp6 n-terminal-like | 0.71 |
|  | C142R125 | UNKNOWN | 0.71 |
|  | C006R065 | atp-binding cassette sub-family f member 2 | 0.71 |
|  | C086R070 | UNKNOWN | 0.71 |
|  | C167R088 | ubiquitin-associated protein 2-like isoform x1 | 0.71 |
|  | C145R062 | f-box lrr-repeat protein 17 | 0.71 |
|  | C064R035 | dual specificity protein phosphatase 14 | 0.71 |
|  | C253R043 | acyl- dehydrogenase -like | 0.71 |
|  | C087R012 | UNKNOWN | 0.71 |
|  | C157R038 | mannosyltransferase-like | 0.71 |
|  | C215R121 | ceramide kinase-like protein isoform 2 | 0.71 |
|  | C108R041 | UNKNOWN | 0.71 |
|  | C119R059 | tyrosine-protein kinase btk | 0.71 |
|  | C144R159 | prefoldin subunit 4 | 0.71 |
|  | C219R149 | UNKNOWN | 0.71 |
|  | C250R114 | polyadenylate-binding protein 4 | 0.70 |
|  | C230R124 | nucleolar protein 14 | 0.70 |
|  | C177R146 | UNKNOWN | 0.70 |
|  | C169R043 | nad-dependent deacetylase sirtuin-5 | 0.70 |
|  | C194R076 | targeting protein for xklp2 | 0.70 |
|  | C058R017 | UNKNOWN | 0.70 |
|  | C187R117 | golgi resident protein gcp60 | 0.70 |
|  | C169R164 | exportin-2 | 0.70 |
|  | C157R131 | steroid receptor rna activator 1 | 0.70 |
|  | C227R169 | 39s ribosomal protein mitochondrial precursor | 0.70 |
|  | C208R092 | sulfide:quinone mitochondrial | 0.70 |
|  | C215R143 | nesprin- partial | 0.70 |
|  | C165R135 | ntf2-related export protein 2 | 0.70 |
|  | C225R137 | UNKNOWN | 0.70 |
|  | C046R076 | novel protein | 0.70 |
|  | C204R131 | xin actin-binding repeat-containing protein 2 isoform 3 | 0.70 |
|  | C140R092 | vesicle-fusing atpase | 0.70 |
|  | C027R071 | 14 kda phosphohistidine phosphatase | 0.70 |
|  | C098R134 | bis(5 -nucleosyl)-tetraphosphatase | 0.70 |
|  | C013R126 | UNKNOWN | 0.70 |
|  | C066R052 | g-protein coupled receptor 182 | 0.70 |
|  | C021R165 | kinesin-like protein kif11-like | 0.70 |
|  | C129R017 | phosphoglucomutase 1 | 0.70 |
|  | C153R053 | protein fam184a | 0.70 |
|  | C060R085 | rab3 gtpase-activating protein non-catalytic subunit | 0.70 |
|  | C225R072 | transposase | 0.70 |
|  | C116R062 | UNKNOWN | 0.70 |
|  | C095R169 | UNKNOWN | 0.70 |
|  | C131R094 | fructose-bisphosphate aldolase a | 0.70 |
|  | C242R073 | acetyl-coenzyme a synthetase 2- mitochondrial-like | 0.70 |
|  | C065R085 | tcb1 transposase | 0.70 |
|  | C123R081 | gamma-crystallin m2-like | 0.70 |
|  | C174R161 | UNKNOWN | 0.70 |
|  | C128R054 | nucleolar protein 14 | 0.70 |
|  | C160R096 | tyrosine-protein kinase jak1 | 0.70 |
|  | C208R109 | UNKNOWN | 0.70 |
|  | C060R051 | UNKNOWN | 0.70 |
|  | C093R033 | UNKNOWN | 0.70 |
|  | C103R128 | UNKNOWN | 0.70 |
|  | C076R167 | choline transporter-like protein 2 | 0.70 |
|  | C052R056 | leupaxin | 0.70 |
|  | C204R087 | sra stem-loop-interacting rna-binding mitochondrial precursor | 0.70 |
|  | C234R027 | gelsolin isoform 1 | 0.70 |
|  | C255R076 | UNKNOWN | 0.70 |
|  | C074R128 | novel protein | 0.70 |
|  | C061R078 | UNKNOWN | 0.70 |
|  | C259R049 | leucine-rich repeat and wd repeat-containing protein 1 | 0.70 |
|  | C146R120 | calumenin isoform 6 | 0.70 |
|  | C125R091 | platelet-activating factor acetylhydrolase | 0.70 |
|  | C191R144 | growth hormone receptor 2 | 0.70 |
|  | C241R119 | UNKNOWN | 0.70 |
|  | C018R152 | enolase 2 | 0.70 |
|  | C068R049 | calmodulin-like protein 4 | 0.70 |
|  | C080R021 | dymeclin-like isoform x1 | 0.70 |
|  | C070R033 | nipped-b-like protein | 0.70 |
|  | C155R053 | pleckstrin homology domain-containing family a member 3 | 0.70 |
|  | C201R076 | UNKNOWN | 0.70 |
|  | C144R110 | UNKNOWN | 0.70 |
|  | C098R149 | sterol regulatory element-binding protein 1 | 0.70 |
|  | C090R106 | UNKNOWN | 0.70 |
|  | C118R098 | proteasomal ubiquitin receptor adrm1-like | 0.70 |
|  | C243R094 | UNKNOWN | 0.70 |
|  | C049R010 | grb2-associated-binding protein 1 isoform 2 | 0.70 |
|  | C080R163 | importin subunit alpha-2 | 0.70 |
|  | C063R052 | major egg antigen | 0.70 |
|  | C134R147 | metallothionein | 0.70 |
|  | C182R064 | transposable element tcb1 transposase | 0.70 |
|  | C010R015^§^ | cd276 antigen-like | 0.70 |
|  | C239R124 | UNKNOWN | 0.70 |
|  | C194R167 | UNKNOWN | 0.70 |
|  | C094R101 | UNKNOWN | 0.70 |
|  | C120R115 | UNKNOWN | 0.70 |
|  | C172R088 | eukaryotic translation initiation factor 4e | 0.70 |
|  | C129R170 | tcb1 transposase | 0.70 |
|  | C104R018 | u5 small nuclear ribonucleoprotein 40 kda protein | 0.70 |
|  | C222R144 | UNKNOWN | 0.70 |
|  | C116R135 | UNKNOWN | 0.70 |
|  | C111R064 | UNKNOWN | 0.70 |
|  | C129R061 | UNKNOWN | 0.70 |
|  | C249R144 | UNKNOWN | 0.70 |
|  | C147R067 | PREDICTED: ankyrin-1-like | 0.70 |
|  | C056R116 | UNKNOWN | 0.70 |
|  | C105R066 | ras-related protein rab-11b | 0.70 |
|  | C248R111 | dual specificity protein kinase ttk | 0.70 |
|  | C112R115 | UNKNOWN | 0.70 |
|  | C231R119 | gigaxonin | 0.69 |
|  | C085R139 | oocyte zinc finger protein 20 | 0.69 |
|  | C165R078 | UNKNOWN | 0.69 |
|  | C141R137 | tubulin epsilon chain | 0.69 |
|  | C044R119 | atp-dependent rna helicase ddx18 | 0.69 |
|  | C158R050 | UNKNOWN | 0.69 |
|  | C195R132 | poly -binding protein 4-like | 0.69 |
|  | C092R140 | transmembrane protein 237a-like | 0.69 |
|  | C155R062 | thioredoxin domain-containing protein 15 | 0.69 |
|  | C043R061 | adp-ribosylation factor-binding protein gga1-like | 0.69 |
|  | C231R139 | transposable element tcb1 transposase | 0.69 |
|  | C049R077 | UNKNOWN | 0.69 |
|  | C199R081 | UNKNOWN | 0.69 |
|  | C030R115 | UNKNOWN | 0.69 |
|  | C122R077 | tetraspanin-5 | 0.69 |
|  | C219R095 | aspartyl aminopeptidase | 0.69 |
|  | C175R071 | protein transport protein sec61 subunit gamma | 0.69 |
|  | C130R125 | canalicular multispecific organic anion transporter 2-like | 0.69 |
|  | C032R137 | e3 ubiquitin-protein ligase arih2 | 0.69 |
|  | C248R039 | UNKNOWN | 0.69 |
|  | C082R087 | nadh dehydrogenase | 0.69 |
|  | C062R092 | UNKNOWN | 0.69 |
|  | C187R163 | cell division control protein 2 homolog | 0.69 |
|  | C232R141 | cytoglobin | 0.69 |
|  | C126R076 | thrombospondin 4 | 0.69 |
|  | C003R088 | protein fam212a-like | 0.69 |
|  | C208R104 | zinc finger protein 521 | 0.69 |
|  | C104R028 | frizzled homolog 7a | 0.69 |
|  | C234R112 | dihydrolipoyllysine-residue acetyltransferase component of pyruvate dehydrogenase mitochondrial | 0.69 |
|  | C139R058 | krueppel-like factor 11-like | 0.69 |
|  | C012R051 | steroid receptor rna activator 1 | 0.69 |
|  | C110R041 | glycine dehydrogenase | 0.69 |
|  | C150R154 | fast kinase domain-containing protein 1 | 0.69 |
|  | C254R121 | UNKNOWN | 0.69 |
|  | C220R165 | UNKNOWN | 0.69 |
|  | C143R043 | retinoic acid receptor rxr-alpha-like | 0.69 |
|  | C154R109 | dual adapter for phosphotyrosine and 3-phosphotyrosine and 3-phosphoinositide | 0.69 |
|  | C153R081 | mitochondrial ornithine transporter 1 | 0.69 |
|  | C051R026 | UNKNOWN | 0.69 |
|  | C150R088 | UNKNOWN | 0.69 |
|  | C067R008 | mitochondrial 28s ribosomal protein s28 | 0.69 |
|  | C118R021 | UNKNOWN | 0.69 |
|  | C068R036 | target of rapamycin complex 2 subunit mapkap1-like | 0.69 |
|  | C063R048 | UNKNOWN | 0.69 |
|  | C213R134 | ras-related protein rab-32-like | 0.69 |
|  | C121R075 | sun domain-containing protein 1 | 0.69 |
|  | C021R057 | UNKNOWN | 0.69 |
|  | C242R010 | UNKNOWN | 0.69 |
|  | C135R066 | teashirt homolog 1-like | 0.69 |
|  | C125R039 | laminin subunit beta-2-like | 0.69 |
|  | C055R024 | mitochondrial nucleoid factor 1 | 0.69 |
|  | C243R144 | haus augmin-like complex subunit 6-like | 0.69 |
|  | C265R143 | spry domain-containing protein 4 | 0.69 |
|  | C198R093 | pleckstrin homology domain-containing family a member 7 | 0.69 |
|  | C210R034 | splicing factor 45 | 0.69 |
|  | C102R138 | t-cell surface glycoprotein cd4-2 | 0.69 |
|  | C123R128 | atp-dependent zinc metalloprotease yme1l1-like | 0.69 |
|  | C166R114 | dna damage-regulated autophagy modulator protein 2 | 0.69 |
|  | C059R026 | syndecan-3 | 0.69 |
|  | C198R132 | exocyst complex component 3 | 0.69 |
|  | C142R132 | UNKNOWN | 0.69 |
|  | C057R104 | UNKNOWN | 0.69 |
|  | C122R107 | UNKNOWN | 0.69 |
|  | C014R141 | dna-directed rna polymerase i subunit rpa43 | 0.69 |
|  | C059R123^‡^ | sperm-associated antigen 5 | 0.69 |
|  | C186R134 | UNKNOWN | 0.69 |
|  | C125R071 | set and mynd domain-containing protein 1 isoform x2 | 0.69 |
|  | C167R099 | UNKNOWN | 0.69 |
|  | C130R155 | UNKNOWN | 0.69 |
|  | C090R060 | secretagogin | 0.69 |
|  | C026R007 | protein disulfide-isomerase a5-like | 0.69 |
|  | C242R080 | UNKNOWN | 0.69 |
|  | C258R130 | protein smg8-like | 0.69 |
|  | C052R015 | mitochondrial nucleoid factor 1 | 0.69 |
|  | C203R070 | serine threonine-protein kinase ulk1 | 0.69 |
|  | C019R142 | UNKNOWN | 0.69 |
|  | C168R119 | transmembrane protein 100 | 0.69 |
|  | C070R024 | ran gtpase-activating protein 1-like | 0.69 |
|  | C241R074 | UNKNOWN | 0.69 |
|  | C149R028 | proline-rich protein bca3 | 0.68 |
|  | C220R073 | ubiquitin carboxyl-terminal hydrolase 28 | 0.68 |
|  | C073R044 | UNKNOWN | 0.68 |
|  | C144R069 | UNKNOWN | 0.68 |
|  | C218R134 | UNKNOWN | 0.68 |
|  | C131R130 | adenylosuccinate lyase | 0.68 |
|  | C195R106 | UNKNOWN | 0.68 |
|  | C153R135 | UNKNOWN | 0.68 |
|  | C193R154 | UNKNOWN | 0.68 |
|  | C181R170 | UNKNOWN | 0.68 |
|  | C132R049 | UNKNOWN | 0.68 |
|  | C001R044 | UNKNOWN | 0.68 |
|  | C242R086 | dual specificity protein kinase clk2 | 0.68 |
|  | C086R036 | UNKNOWN | 0.68 |
|  | C199R092 | UNKNOWN | 0.68 |
|  | C078R131 | proteasome subunit beta type-7 precursor | 0.68 |
|  | C180R063 | pyruvate kinase | 0.68 |
|  | C160R134 | UNKNOWN | 0.68 |
|  | C082R020 | histone-lysine n-methyltransferase ehmt1 | 0.68 |
|  | C172R141 | UNKNOWN | 0.68 |
|  | C089R086 | nuclear pore complex protein nup98-nup96-like | 0.68 |
|  | C125R025 | cholecystokinin | 0.68 |
|  | C131R042 | monocarboxylate transporter 2-like | 0.68 |
|  | C131R168 | UNKNOWN | 0.68 |
|  | C062R142 | UNKNOWN | 0.68 |
|  | C256R106 | transcriptional regulator kaiso-like | 0.68 |
|  | C005R139 | mature t-cell proliferation 1 neighbor | 0.68 |
|  | C028R083 | exosome complex component mtr3-like | 0.68 |
|  | C152R075 | transmembrane protein 184b | 0.68 |
|  | C115R125 | ino80 complex subunit c | 0.68 |
|  | C127R129 | UNKNOWN | 0.68 |
|  | C199R053 | disabled homolog 2-interacting | 0.68 |
|  | C109R127 | placenta-specific protein 9-like | 0.68 |
|  | C075R144 | homeobox protein six3 | 0.68 |
|  | C060R140 | UNKNOWN | 0.68 |
|  | C124R010 | mitochondrial inner membrane protease subunit 1 | 0.68 |
|  | C153R122 | ras-related protein rab-1a | 0.68 |
|  | C007R090 | mediator of rna polymerase ii transcription subunit 13-like | 0.68 |
|  | C120R059 | zinc finger protein 180 | 0.68 |
|  | C202R159 | 14-3-3 protein beta alpha-2 | 0.68 |
|  | C111R137 | gmp reductase 1 | 0.68 |
|  | C001R055 | UNKNOWN | 0.68 |
|  | C045R129 | stress-associated endoplasmic reticulum protein 1 | 0.68 |
|  | C140R018 | whey acidic protein precursor | 0.68 |
|  | C248R102 | 26s proteasome non-atpase regulatory subunit 2 | 0.68 |
|  | C210R050 | probable xaa-pro aminopeptidase 3 | 0.68 |
|  | C127R007 | lysosomal protective | 0.68 |
|  | C020R091 | pyrroline-5-carboxylate reductase 3 | 0.68 |
|  | C103R105 | UNKNOWN | 0.68 |
|  | C253R006 | tripartite motif-containing protein 16-like | 0.68 |
|  | C137R128 | probable udp-sugar transporter protein slc35a4 | 0.68 |
|  | C174R113 | UNKNOWN | 0.68 |
|  | C101R023 | annexin max4-like | 0.68 |
|  | C150R163 | methylmalonate-semialdehyde dehydrogenase | 0.68 |
|  | C096R079 | UNKNOWN | 0.68 |
|  | C033R119 | UNKNOWN | 0.68 |
|  | C034R048 | kelch domain-containing protein 4 | 0.68 |
|  | C200R164 | ictacalcin | 0.68 |
|  | C261R044 | alpha-actinin-3-like isoform 2 | 0.68 |
|  | C079R150 | stress-70 mitochondrial-like | 0.68 |
|  | C088R124 | tight junction-associated protein 1 | 0.68 |
|  | C232R081 | bladder cancer-associated protein | 0.68 |
|  | C242R064 | cullin 4a isoform cra_a | 0.68 |
|  | C107R060 | u6 snrna-associated sm-like protein lsm5 | 0.68 |
|  | C202R057 | nuclear autoantigenic sperm protein (histone-binding) | 0.68 |
|  | C017R054 | UNKNOWN | 0.68 |
|  | C163R052 | UNKNOWN | 0.68 |
|  | C216R047 | iron-sulfur protein nubpl-like | 0.68 |
|  | C205R125 | cytochrome c oxidase subunit mitochondrial-like | 0.68 |
|  | C128R164 | UNKNOWN | 0.68 |
|  | C135R033 | kelch domain-containing protein 3-like isoform x1 | 0.68 |
|  | C028R116 | UNKNOWN | 0.68 |
|  | C239R126 | transposable element tcb1 transposase | 0.68 |
|  | C183R038 | trio and f-actin-binding | 0.68 |
|  | C194R142 | solute carrier family 25 (mitochondrial carrier dicarboxylate transporter) member 10 | 0.68 |
|  | C141R113 | geranylgeranyl transferase type-2 subunit alpha | 0.68 |
|  | C178R144 | adenosine 3 -phospho 5 -phosphosulfate transporter 2 | 0.68 |
|  | C039R091 | cell differentiation protein rcd1 homolog | 0.68 |
|  | C114R013 | beta-taxilin | 0.67 |
|  | C169R060 | UNKNOWN | 0.67 |
|  | C204R086 | cytochrome c oxidase subunit mitochondrial-like | 0.67 |
|  | C162R130 | protein regulator of cytokinesis 1 | 0.67 |
|  | C087R053 | xylose isomerase | 0.67 |
|  | C233R139 | UNKNOWN | 0.67 |
|  | C033R012 | ankyrin repeat domain-containing protein 13c-a-like | 0.67 |
|  | C174R165 | UNKNOWN | 0.67 |
|  | C085R088 | UNKNOWN | 0.67 |
|  | C040R048 | UNKNOWN | 0.67 |
|  | C142R018 | UNKNOWN | 0.67 |
|  | C150R084 | UNKNOWN | 0.67 |
|  | C242R103 | tax1-binding protein 3 | 0.67 |
|  | C166R011 | ran gtpase-activating protein 1-like | 0.67 |
|  | C200R073 | drebrin-like protein | 0.67 |
|  | C204R026 | UNKNOWN | 0.67 |
|  | C097R086 | zinc finger protein 408 | 0.67 |
|  | C242R028 | UNKNOWN | 0.67 |
|  | C088R033 | macrophage receptor marco | 0.67 |
|  | C060R086 | ribosomal protein s18 | 0.67 |
|  | C016R109 | 3 -5 exoribonuclease 1-like | 0.67 |
|  | C066R130 | UNKNOWN | 0.67 |
|  | C056R047 | protein phosphatase 1 regulatory subunit 12c | 0.67 |
|  | C073R054 | myozenin-2 | 0.67 |
|  | C037R057 | UNKNOWN | 0.67 |
|  | C111R078 | UNKNOWN | 0.67 |
|  | C090R044 | dipeptidyl peptidase 9 | 0.67 |
|  | C063R121 | 40s ribosomal protein s5 | 0.67 |
|  | C078R130 | endonuclease domain-containing 1 | 0.67 |
|  | C224R128 | UNKNOWN | 0.67 |
|  | C094R091 | n-alpha-acetyltransferase auxiliary subunit | 0.67 |
|  | C166R051 | UNKNOWN | 0.67 |
|  | C064R074 | carbonyl reductase family member 4 | 0.67 |
|  | C164R084 | UNKNOWN | 0.67 |
|  | C263R129 | guanine nucleotide-binding protein g subunit gamma-t1 | 0.67 |
|  | C119R016 | methylmalonic aciduria and homocystinuria type c protein homolog | 0.67 |
|  | C246R089 | UNKNOWN | 0.67 |
|  | C190R168 | UNKNOWN | 0.67 |
|  | C010R019 | protein | 0.67 |
|  | C173R090 | dehydrogenase reductase sdr family member 4 | 0.67 |
|  | C093R156 | UNKNOWN | 0.67 |
|  | C042R058 | cytochrome c oxidase subunit vib isoform 1 | 0.67 |
|  | C103R111 | 14 kda phosphohistidine phosphatase | 0.67 |
|  | C010R045 | prefoldin subunit 2 | 0.67 |
|  | C161R085 | ras-related protein rab-5c | 0.67 |
|  | C171R166 | UNKNOWN | 0.67 |
|  | C252R104 | hemoglobin subunit beta-1 | 0.67 |
|  | C067R163 | UNKNOWN | 0.67 |
|  | C071R069 | gtpase imap family member 7-like | 0.67 |
|  | C243R131 | UNKNOWN | 0.67 |
|  | C242R044 | atp synthase subunit mitochondrial-like | 0.67 |
|  | C194R011 | nuclear distribution protein nude homolog 1-like | 0.67 |
|  | C224R160 | UNKNOWN | 0.67 |
|  | C253R046 | UNKNOWN | 0.67 |
|  | C189R096 | protein fam184a-like | 0.67 |
|  | C149R046 | UNKNOWN | 0.67 |
|  | C159R146 | UNKNOWN | 0.67 |
|  | C004R153 | tropomodulin-4 | 0.67 |
|  | C102R043 | myozenin 1-like | 0.67 |
|  | C185R155 | suppression of tumorigenicity 5 | 0.67 |
|  | C133R153 | e3 ubiquitin-protein ligase rnf25 | 0.67 |
|  | C227R151 | UNKNOWN | 0.67 |
|  | C019R079 | phospholipid scramblase 1 | 0.67 |
|  | C191R166 | ribosomal large subunit pseudouridine synthase b | 0.67 |
|  | C122R089 | endothelial cell-specific molecule 1-like | 0.67 |
|  | C215R122 | UNKNOWN | 0.67 |
|  | C140R045 | UNKNOWN | 0.67 |
|  | C165R125 | ataxin-10 | 0.67 |
|  | C001R105 | stress-70 mitochondrial | 0.67 |
|  | C242R082 | membrane protein mlc1-like | 0.67 |
|  | C254R117 | UNKNOWN | 0.67 |
|  | C123R106 | UNKNOWN | 0.67 |
|  | C217R036 | sperm-associated antigen 7 | 0.67 |
|  | C261R133 | tudor domain-containing protein 3-like | 0.67 |
|  | C189R164 | acidic leucine-rich nuclear phosphoprotein 32 family member b | 0.67 |
|  | C162R135 | UNKNOWN | 0.67 |
|  | C228R131 | dna polymerase delta catalytic subunit | 0.67 |
|  | C191R116 | trna selenocysteine 1-associated protein 1 | 0.67 |
|  | C266R147 | importin 5 | 0.67 |
|  | C164R056 | mitochondrial ribosomal protein s16 | 0.67 |
|  | C204R139 | UNKNOWN | 0.67 |
|  | C242R087 | oocyte zinc finger protein 20 | 0.67 |
|  | C092R111 | UNKNOWN | 0.67 |
|  | C254R073 | allograft inflammatory factor 1 | 0.67 |
|  | C078R046 | hypoxia up-regulated isoform cra_a | 0.67 |
|  | C224R086 | rnf213 protein | 0.67 |
|  | C097R013 | UNKNOWN | 0.67 |
|  | C166R137 | cyclic amp-dependent transcription factor atf-1 | 0.67 |
|  | C120R023 | protein transport protein sec61 subunit gamma | 0.67 |
|  | C127R016 | UNKNOWN | 0.66 |
|  | C082R116 | sec23-interacting protein | 0.66 |
|  | C077R080 | UNKNOWN | 0.66 |
|  | C017R119 | UNKNOWN | 0.66 |
|  | C164R128 | alkylated dna repair protein alkb homolog 1 | 0.66 |
|  | C102R135 | 39s ribosomal protein mitochondrial | 0.66 |
|  | C065R083 | UNKNOWN | 0.66 |
|  | C178R160 | UNKNOWN | 0.66 |
|  | C097R043 | zinc finger protein 292 | 0.66 |
|  | C104R030 | ran protein | 0.66 |
|  | C021R009 | lim-domain binding factor 3 | 0.66 |
|  | C162R071 | UNKNOWN | 0.66 |
|  | C022R034 | mhc class i | 0.66 |
|  | C030R066 | transposase | 0.66 |
|  | C138R113 | UNKNOWN | 0.66 |
|  | C165R024 | protein farnesyltransferase geranylgeranyltransferase type-1 subunit alpha | 0.66 |
|  | C221R109 | protein dpy-30 homolog | 0.66 |
|  | C201R112 | zinc finger bed domain-containing protein 1-like | 0.66 |
|  | C156R101 | mediator of rna polymerase ii transcription subunit partial | 0.66 |
|  | C051R107 | serologically defined colon cancer antigen 3 homolog | 0.66 |
|  | C013R145 | ras-related protein rab-10 | 0.66 |
|  | C059R106 | alpha- -mannosyl-glycoprotein 2-beta-n-acetylglucosaminyltransferase | 0.66 |
|  | C054R036 | tsc22 domain family protein 3 isoform 1 | 0.66 |
|  | C214R057 | UNKNOWN | 0.66 |
|  | C112R053 | UNKNOWN | 0.66 |
|  | C039R041 | UNKNOWN | 0.66 |
|  | C209R084 | protein pat1 homolog 1 | 0.66 |
|  | C087R035 | oligosaccharyltransferase complex subunit ostc | 0.66 |
|  | C011R073 | UNKNOWN | 0.66 |
|  | C145R053 | unnamed protein product | 0.66 |
|  | C043R083 | gastrula zinc finger | 0.66 |
|  | C077R088 | UNKNOWN | 0.66 |
|  | C252R073 | tumor suppressor candidate 4 | 0.66 |
|  | C208R159 | cytosolic non-specific dipeptidase | 0.66 |
|  | C021R017 | cytoskeleton-associated protein 2 | 0.66 |
|  | C107R035 | yth domain family protein 3 isoform x4 | 0.66 |
|  | C182R044 | UNKNOWN | 0.66 |
|  | C124R145 | myelin basic | 0.66 |
|  | C092R110 | 80 kda mcm3-associated protein | 0.66 |
|  | C230R085 | serrate rna effector molecule homolog | 0.66 |
|  | C137R142 | polyadenylate-binding protein 4 | 0.66 |
|  | C009R108 | wd repeat-containing protein c2orf44 homolog | 0.66 |
|  | C244R089 | UNKNOWN | 0.66 |
|  | C246R046 | fibroblast growth factor 3 | 0.66 |
|  | C050R024 | transmembrane and coiled-coil domain-containing protein 7 | 0.66 |
|  | C019R094 | UNKNOWN | 0.66 |
|  | C085R117 | nucleosome-remodeling factor subunit bptf | 0.66 |
|  | C227R116 | structural maintenance of chromosomes protein 5 | 0.66 |
|  | C196R110 | c19orf29 partial | 0.66 |
|  | C016R116 | gamma b (breast cancer-specific protein 1) | 0.66 |
|  | C013R012 | poly -specific endoribonuclease-c-like | 0.66 |
|  | C229R057 | UNKNOWN | 0.66 |
|  | C124R008 | protein canopy homolog 4 | 0.66 |
|  | C110R033 | ccr4-not transcription complex subunit 10-like | 0.66 |
|  | C147R080 | dna repair protein complementing xp-a cells | 0.66 |
|  | C197R093 | dolichyl-diphosphooligosaccharide--protein glycosyltransferase subunit stt3a | 0.66 |
|  | C016R167 | UNKNOWN | 0.66 |
|  | C173R075 | UNKNOWN | 0.66 |
|  | C146R126 | extracellular matrix protein 1 precursor | 0.66 |
|  | C041R034 | histidine--trna cytoplasmic isoform 1 | 0.66 |
|  | C061R060 | UNKNOWN | 0.66 |
|  | C109R061 | cul2 protein | 0.66 |
|  | C156R156 | UNKNOWN | 0.66 |
|  | C234R101 | UNKNOWN | 0.66 |
|  | C064R098 | UNKNOWN | 0.66 |
|  | C104R157 | transcription factor | 0.66 |
|  | C162R138 | cytochrome b-c1 complex subunit mitochondrial precursor | 0.66 |
|  | C074R036 | UNKNOWN | 0.66 |
|  | C213R045 | guanosine-3 -bis 3 -pyrophosphohydrolase mesh1 | 0.66 |
|  | C205R097 | eomesodermin-like protein a-1 | 0.66 |
|  | C052R006 | epoxide hydrolase 1-like | 0.66 |
|  | C144R158 | trans- -enoyl- reductase-like | 0.66 |
|  | C235R017 | amyloid beta a4 precursor protein-binding family a member 1 | 0.65 |
|  | C165R089 | UNKNOWN | 0.65 |
|  | C187R110 | zinc finger protein 782- partial | 0.65 |
|  | C149R157 | phosphatidylinositol-binding clathrin assembly | 0.65 |
|  | C141R134 | low quality protein: obscurin-like | 0.65 |
|  | C220R046 | UNKNOWN | 0.65 |
|  | C171R124 | UNKNOWN | 0.65 |
|  | C263R075 | zinc finger protein 592 | 0.65 |
|  | C008R125 | lysine-specific histone demethylase 1a-like | 0.65 |
|  | C057R087 | transposable element tcb1 transposase | 0.65 |
|  | C116R127 | adenylosuccinate lyase | 0.65 |
|  | C135R128 | solute carrier organic anion transporter family member 1c1-like | 0.65 |
|  | C158R102 | UNKNOWN | 0.65 |
|  | C120R052 | proline-rich protein 5 | 0.65 |
|  | C259R056 | UNKNOWN | 0.65 |
|  | C140R003 | lysozyme g | 0.65 |
|  | C047R145 | UNKNOWN | 0.65 |
|  | C163R063 | fermitin family homolog 3 | 0.65 |
|  | C085R147 | cleavage and polyadenylation specificity factor subunit 5 | 0.65 |
|  | C096R105 | nadh dehydrogenase iron-sulfur protein 5 | 0.65 |
|  | C223R153 | UNKNOWN | 0.65 |
|  | C236R072 | g patch domain-containing protein 8-like isoform x1 | 0.65 |
|  | C252R089 | oocyte zinc finger protein 20 | 0.65 |
|  | C177R102 | UNKNOWN | 0.65 |
|  | C065R101 | nesprin-1-like | 0.65 |
|  | C173R077 | protein kinase c eta type | 0.65 |
|  | C231R060 | phosphatidylinositol-4-phosphate 5-kinase type-1 beta | 0.65 |
|  | C056R119 | UNKNOWN | 0.65 |
|  | C133R077 | fas-associated factor 1 | 0.65 |
|  | C250R019 | small subunit processome component 20 homolog | 0.65 |
|  | C169R150 | glycogen debranching enzyme | 0.65 |
|  | C191R130 | probable g-protein coupled receptor 148-like | 0.65 |
|  | C088R055 | UNKNOWN | 0.65 |
|  | C042R115 | UNKNOWN | 0.65 |
|  | C154R064 | macrophage receptor marco | 0.65 |
|  | C081R081 | complement c4-1 | 0.65 |
|  | C147R125 | calpain-1 catalytic subunit | 0.65 |
|  | C132R072 | f-box only protein 46 | 0.65 |
|  | C029R089 | bifunctional purine biosynthesis protein purh | 0.65 |
|  | C121R162 | nicotinamide riboside kinase 2 | 0.65 |
|  | C164R146 | tetratricopeptide repeat protein 5 | 0.65 |
|  | C036R042 | actin-related protein 2 3 complex subunit 2 | 0.65 |
|  | C261R017 | collagen alpha-3 chain-like | 0.65 |
|  | C121R090 | eukaryotic translation initiation factor 4 gamma 2-like | 0.65 |
|  | C227R073 | lipoprotein lipase | 0.65 |
|  | C198R126 | UNKNOWN | 0.65 |
|  | C157R041 | c-jun-amino-terminal kinase-interacting protein 1-like | 0.65 |
|  | C009R039 | UNKNOWN | 0.65 |
|  | C205R058 | periostin-like | 0.65 |
|  | C231R059 | tubulin polyglutamylase complex subunit 2 | 0.65 |
|  | C065R122 | rho-related gtp-binding protein | 0.65 |
|  | C109R134 | UNKNOWN | 0.65 |
|  | C076R016 | probable phosphatase phospho1-like | 0.65 |
|  | C155R077 | angiotensin-converting enzyme-like | 0.65 |
|  | C063R153 | UNKNOWN | 0.65 |
|  | C257R103 | UNKNOWN | 0.65 |
|  | C002R131 | UNKNOWN | 0.65 |
|  | C006R113 | activator of 90 kda heat shock protein atpase homolog 1 | 0.65 |
|  | C202R116 | UNKNOWN | 0.65 |
|  | C208R057 | collagen alpha-3 chain-like | 0.65 |
|  | C204R081 | mitochondrial import inner membrane translocase subunit tim8 a | 0.65 |
|  | C218R035 | ice nucleation protein | 0.65 |
|  | C013R130 | zinc finger mym-type protein 1-like | 0.65 |
|  | C114R169 | nucleolar protein 8 | 0.65 |
|  | C100R119 | caspase-1 precursor | 0.65 |
|  | C072R096 | transmembrane protein 8c-like | 0.65 |
|  | C098R099 | ribosomal protein s6 kinase beta-1-like | 0.65 |
|  | C018R137^‡^ | heterogeneous nuclear ribonucleoprotein l | 0.65 |
|  | C011R107 | UNKNOWN | 0.65 |
|  | C156R070 | UNKNOWN | 0.65 |
|  | C110R070 | e3 ubiquitin-protein ligase ubr3 | 0.65 |
|  | C170R088 | pogo transposable element with znf domain a | 0.65 |
|  | C060R105 | s-antigen partial | 0.65 |
|  | C119R065 | collagen alpha-1 chain | 0.65 |
|  | C169R099 | pleckstrin homology domain-containing family h member 2 | 0.65 |
|  | C250R053 | sorbin and sh3 domain containing 3 | 0.65 |
|  | C034R106 | insulin-like growth factor 1 receptor | 0.65 |
|  | C157R147 | UNKNOWN | 0.65 |
|  | C211R092 | transcriptional regulator erg isoform 1 | 0.64 |
|  | C017R040 | thymosin beta-12 | 0.64 |
|  | C188R066 | UNKNOWN | 0.64 |
|  | C152R085 | UNKNOWN | 0.64 |
|  | C034R116 | sickle tail protein homolog | 0.64 |
|  | C035R064 | novel protein vertebrate nebulin | 0.64 |
|  | C001R106 | cd81 antigen | 0.64 |
|  | C144R056 | UNKNOWN | 0.64 |
|  | C079R009 | nad mitochondrial-like isoform x1 | 0.64 |
|  | C207R028 | zinc finger ccch domain-containing protein 18-like | 0.64 |
|  | C214R075 | g patch domain-containing protein 8-like isoform x1 | 0.64 |
|  | C059R032 | fish virus induced trim protein | 0.64 |
|  | C248R116 | phenylalanine--trna ligase alpha subunit | 0.64 |
|  | C108R169 | solute carrier family 30 (zinc transporter) member 1 | 0.64 |
|  | C066R103 | UNKNOWN | 0.64 |
|  | C020R076 | protein rft1 homolog | 0.64 |
|  | C035R077 | UNKNOWN | 0.64 |
|  | C132R033 | UNKNOWN | 0.64 |
|  | C095R143 | UNKNOWN | 0.64 |
|  | C038R090 | rap1 gtpase-gdp dissociation stimulator 1-like | 0.64 |
|  | C207R155 | atp-dependent rna helicase dhx8 | 0.64 |
|  | C155R037 | basal cell adhesion molecule precursor | 0.64 |
|  | C191R120 | sodium potassium calcium exchanger 3-like | 0.64 |
|  | C118R112 | UNKNOWN | 0.64 |
|  | C013R085 | protein arginine n-methyltransferase 1 isoform 2 | 0.64 |
|  | C130R128 | atpase family aaa domain-containing protein 3 | 0.64 |
|  | C156R084 | protein virilizer homolog | 0.64 |
|  | C247R084 | polymorphic mucin truncated splice variant ic6 2 | 0.64 |
|  | C258R070 | sjoegren syndrome scleroderma autoantigen 1 | 0.64 |
|  | C156R098 | beta-taxilin | 0.64 |
|  | C111R139 | multidrug resistance protein 1-like | 0.64 |
|  | C179R122 | zinc finger protein 214 | 0.64 |
|  | C239R132 | digestive organ expansion factor homolog | 0.64 |
|  | C114R170 | tropomodulin-4 | 0.64 |
|  | C183R145 | UNKNOWN | 0.64 |
|  | C201R021 | UNKNOWN | 0.64 |
|  | C066R096 | protein mon2 homolog | 0.64 |
|  | C087R151 | UNKNOWN | 0.64 |
|  | C127R070 | pericentriolar material 1 protein | 0.64 |
|  | C191R015 | UNKNOWN | 0.64 |
|  | C014R091 | jmjc domain-containing protein 4-like | 0.64 |
|  | C042R083 | protein tob1-like | 0.64 |
|  | C171R064 | tbc domain-containing protein kinase-like partial | 0.64 |
|  | C220R077 | atr-interacting protein | 0.64 |
|  | C099R103 | UNKNOWN | 0.64 |
|  | C064R084 | ubiquitin carboxyl-terminal hydrolase 4 isoform 1 | 0.64 |
|  | C074R021 | UNKNOWN | 0.64 |
|  | C206R027 | UNKNOWN | 0.64 |
|  | C169R064 | host cell factor 1 | 0.64 |
|  | C160R139 | zinc finger protein 180 | 0.64 |
|  | C035R140 | integrase core domain protein | 0.64 |
|  | C098R135 | reticulon 2 | 0.64 |
|  | C212R112 | myocyte-specific enhancer factor 2a | 0.64 |
|  | C228R127 | UNKNOWN | 0.64 |
|  | C120R122 | peroxisomal leader peptide-processing protease | 0.64 |
|  | C022R038 | macrophage migration inhibitory factor | 0.64 |
|  | C234R139 | zinc finger and btb domain-containing protein 17 | 0.64 |
|  | C176R096 | dna-directed rna polymerases and iii subunit rpabc5 | 0.64 |
|  | C077R145 | gtpase imap family member 7 | 0.64 |
|  | C141R020 | UNKNOWN | 0.64 |
|  | C094R120 | atp-dependent rna helicase dhx30-like | 0.64 |
|  | C230R079 | structural maintenance of chromosomes protein 1a | 0.64 |
|  | C153R084 | atp-dependent rna helicase dhx8 | 0.64 |
|  | C082R105 | lymphocyte cytosolic protein 2 | 0.64 |
|  | C132R028 | UNKNOWN | 0.64 |
|  | C144R042 | UNKNOWN | 0.64 |
|  | C164R147 | p53 and dna damage-regulated protein 1 | 0.64 |
|  | C147R007 | UNKNOWN | 0.64 |
|  | C110R085 | patched domain-containing protein 3-like | 0.64 |
|  | C176R122 | UNKNOWN | 0.64 |
|  | C253R077 | protein yippee-like 2 | 0.64 |
|  | C075R155 | arf-gap with sh3 ank repeat and ph domain-containing protein 1 | 0.64 |
|  | C163R020 | gastrula zinc finger protein | 0.64 |
|  | C182R122 | oxysterol-binding protein 6-like | 0.64 |
|  | C153R063 | 60s ribosomal protein l8 | 0.64 |
|  | C106R123 | eomesodermin homolog | 0.64 |
|  | C165R107 | bone morphogenetic protein 3b-like | 0.64 |
|  | C063R090 | centromere protein u-like | 0.64 |
|  | C203R137 | probable e3 ubiquitin-protein ligase trim8 | 0.63 |
|  | C110R165 | UNKNOWN | 0.63 |
|  | C065R069 | cugbp elav-like family member 4-like | 0.63 |
|  | C068R056 | metabotropic glutamate receptor 2 | 0.63 |
|  | C144R016 | rna binding motif protein 14 | 0.63 |
|  | C025R075 | guanine nucleotide exchange factor mss4 | 0.63 |
|  | C218R024 | UNKNOWN | 0.63 |
|  | C040R163 | thioredoxin 2 | 0.63 |
|  | C093R081 | UNKNOWN | 0.63 |
|  | C131R073 | protein fam84a | 0.63 |
|  | C034R082 | centrosomal protein of 70 kda-like | 0.63 |
|  | C078R033 | UNKNOWN | 0.63 |
|  | C161R036 | leucine-rich repeat and immunoglobulin-like domain-containing nogo receptor-interacting protein 3 | 0.63 |
|  | C132R029 | complement component c1q receptor | 0.63 |
|  | C001R029 | atp-dependent rna helicase | 0.63 |
|  | C041R146 | dna topoisomerase 2-binding protein 1 | 0.63 |
|  | C168R098 | f-box-like wd repeat-containing protein tbl1xr1-like | 0.63 |
|  | C164R113 | zinc finger protein 423 | 0.63 |
|  | C145R024 | UNKNOWN | 0.63 |
|  | C255R052 | high mobility group-t protein | 0.63 |
|  | C238R116 | ankyrin repeat domain-containing protein 1-like | 0.63 |
|  | C061R128 | presenilin-2 | 0.63 |
|  | C257R084 | UNKNOWN | 0.63 |
|  | C030R055 | atpase family aaa domain-containing protein 2 | 0.63 |
|  | C261R058 | zinc finger mym-type protein 1 | 0.63 |
|  | C051R162 | UNKNOWN | 0.63 |
|  | C172R143 | 26s proteasome complex subunit dss1 | 0.63 |
|  | C217R065 | probable glutamyl-trna amidotransferase subunit mitochondrial-like | 0.63 |
|  | C162R074 | s100-a1 | 0.63 |
|  | C054R121 | disabled homolog 2 isoform 1 | 0.63 |
|  | C174R078 | coiled-coil alpha-helical rod protein 1 | 0.63 |
|  | C090R075 | transposase | 0.63 |
|  | C193R017 | general transcription factor iie subunit 1 | 0.63 |
|  | C187R156 | nadh dehydrogenase subunit 1 | 0.63 |
|  | C057R089 | protein | 0.63 |
|  | C092R066 | interferon-related developmental regulator 2 | 0.63 |
|  | C223R091 | lamin-b receptor | 0.63 |
|  | C070R091 | map kinase-activated protein kinase 3 | 0.63 |
|  | C219R145 | UNKNOWN | 0.63 |
|  | C242R141 | cytochrome b-c1 complex subunit 8 | 0.63 |
|  | C036R023 | UNKNOWN | 0.63 |
|  | C017R086 | ankyrin repeat and mynd domain-containing protein 2 | 0.63 |
|  | C016R132 | serine threonine-protein kinase tousled-like 1-b-like isoform 3 | 0.63 |
|  | C215R110 | nucleobindin 2a | 0.63 |
|  | C042R030 | tyrosine-protein kinase 223-like | 0.63 |
|  | C146R147 | UNKNOWN | 0.63 |
|  | C044R129 | mitochondrial intermembrane space import and assembly protein 40 | 0.63 |
|  | C099R121 | nuclear migration protein nudc | 0.63 |
|  | C222R142 | eukaryotic peptide chain release factor subunit 1 | 0.63 |
|  | C184R120 | zinc finger protein 451 isoform x1 | 0.63 |
|  | C118R067 | interferon-stimulated 20 kda exonuclease-like 2 | 0.63 |
|  | C086R088 | acidic leucine-rich nuclear phosphoprotein 32 family member a | 0.63 |
|  | C186R049 | UNKNOWN | 0.63 |
|  | C033R013 | UNKNOWN | 0.63 |
|  | C230R150 | akirin 2 | 0.63 |
|  | C166R022 | gon-4-like protein | 0.63 |
|  | C233R131 | transmembrane protein mitochondrial-like | 0.63 |
|  | C046R087 | cytochrome c oxidase subunit mitochondrial-like | 0.63 |
|  | C132R124 | transforming protein | 0.63 |
|  | C050R055 | tubulin-specific chaperone d | 0.63 |
|  | C122R035 | mitochondrial intermediate peptidase | 0.63 |
|  | C146R140 | transposable element tcb1 transposase | 0.63 |
|  | C018R079 | UNKNOWN | 0.63 |
|  | C172R103 | lissencephaly-1 homolog b | 0.63 |
|  | C246R053 | UNKNOWN | 0.63 |
|  | C012R045 | enolase 3-2 | 0.63 |
|  | C188R100 | transmembrane protein 25-like | 0.63 |
|  | C105R126 | UNKNOWN | 0.63 |
|  | C068R077 | u2 small nuclear ribonucleoprotein b | 0.63 |
|  | C202R083 | fatty acid-binding intestinal | 0.63 |
|  | C028R034 | nuclear transport factor 2 | 0.63 |
|  | C159R170 | UNKNOWN | 0.63 |
|  | C178R120 | lymphocyte cytosolic protein 2 | 0.63 |
|  | C229R038 | heat shock protein hsp 90-alpha 1-like | 0.63 |
|  | C128R066 | UNKNOWN | 0.63 |
|  | C025R167 | perforin-1-like | 0.63 |
|  | C108R062 | wd repeat-containing protein mio-like | 0.62 |
|  | C039R055 | sarcoplasmic reticulum histidine-rich calcium-binding protein | 0.62 |
|  | C130R039 | glutathione s-transferase c-terminal domain-containing protein | 0.62 |
|  | C105R033 | UNKNOWN | 0.62 |
|  | C122R111 | UNKNOWN | 0.62 |
|  | C018R051 | endoplasmin- partial | 0.62 |
|  | C154R042 | UNKNOWN | 0.62 |
|  | C028R103 | UNKNOWN | 0.62 |
|  | C229R095 | zinc finger ccch domain-containing protein 7b-like | 0.62 |
|  | C172R133 | epidermis-type lipoxygenase 3-like | 0.62 |
|  | C206R148 | 14-3-3 protein beta alpha | 0.62 |
|  | C142R098 | lim-domain binding factor 3 | 0.62 |
|  | C025R170 | cellular tumor antigen p53 | 0.62 |
|  | C155R090 | proprotein convertase subtilisin kexin type 6-like | 0.62 |
|  | C130R143 | pyrroline-5-carboxylate reductase 2 | 0.62 |
|  | C078R054 | exocyst complex component 6b | 0.62 |
|  | C237R079 | pap-associated domain-containing protein partial | 0.62 |
|  | C076R159 | ras and ef-hand domain-containing | 0.62 |
|  | C035R072 | dapper homolog 1 | 0.62 |
|  | C197R079 | nadh dehydrogenase subunit 4 | 0.62 |
|  | C202R103 | huwe1 partial | 0.62 |
|  | C196R093 | unconventional myosin-ic-like | 0.62 |
|  | C245R019 | pleckstrin homology domain-containing family m member 1 | 0.62 |
|  | C194R089 | UNKNOWN | 0.62 |
|  | C172R016 | UNKNOWN | 0.62 |
|  | C158R046 | myomesin-3 | 0.62 |
|  | C080R046 | adipocyte enhancer-binding protein 1-like | 0.62 |
|  | C176R053 | eosinophil peroxidase-like | 0.62 |
|  | C131R077 | myosin light chain kinase 3 | 0.62 |
|  | C247R137 | UNKNOWN | 0.62 |
|  | C012R007 | peptide mitochondrial | 0.62 |
|  | C248R063 | UNKNOWN | 0.62 |
|  | C163R045 | phosphatidylinositol 3-kinase catalytic subunit type 3-like | 0.62 |
|  | C010R047 | UNKNOWN | 0.62 |
|  | C093R148 | dna polymerase delta catalytic subunit-like | 0.62 |
|  | C136R050 | UNKNOWN | 0.62 |
|  | C190R075 | type alpha partial | 0.62 |
|  | C165R091 | protein emsy | 0.62 |
|  | C183R031 | UNKNOWN | 0.62 |
|  | C233R147 | myb-binding protein 1a-like | 0.62 |
|  | C168R102 | tyrosine-protein kinase jak1-like | 0.62 |
|  | C078R024 | mitochondrial sodium hydrogen exchanger 9b2 | 0.62 |
|  | C129R107 | glutaminyl-peptide cyclotransferase-like | 0.62 |
|  | C116R148 | protein | 0.62 |
|  | C163R082 | cyclin-d-binding myb-like transcription factor 1 | 0.62 |
|  | C159R030 | sulfotransferase 6b1 | 0.62 |
|  | C119R035 | transcription factor 12-like isoform 1 | 0.62 |
|  | C142R031 | transforming growth factor-beta-induced protein ig-h3 | 0.62 |
|  | C023R036 | UNKNOWN | 0.62 |
|  | C107R105 | cellular retinoic acid-binding protein 2-like | 0.62 |
|  | C009R095 | phosphatidylinositol 5-phosphate 4-kinase type-2 beta | 0.62 |
|  | C064R076 | UNKNOWN | 0.62 |
|  | C227R061 | abl interactor 1 | 0.62 |
|  | C172R168 | peroxisome proliferator-activated receptor beta | 0.62 |
|  | C019R107 | UNKNOWN | 0.62 |
|  | C049R045 | estrogen-related receptor gamma | 0.62 |
|  | C166R010 | plac8-like protein 1 | 0.62 |
|  | C153R029 | UNKNOWN | 0.62 |
|  | C028R026 | kelch-like protein 31 | 0.62 |
|  | C141R061 | protein tyrosine phosphatase type iva 3-like isoform 2 | 0.62 |
|  | C141R021 | ring-box protein 1 | 0.62 |
|  | C183R057 | fish virus induced trim protein | 0.62 |
|  | C178R167 | transducin -like 2 | 0.62 |
|  | C003R029 | multidrug resistance-associated protein 1-like | 0.62 |
|  | C028R170 | transposase | 0.62 |
|  | C035R018 | acidic leucine-rich nuclear phosphoprotein 32 family member b | 0.62 |
|  | C214R029 | serine threonine-protein phosphatase pp1-alpha catalytic subunit isoform 1 | 0.62 |
|  | C146R110 | myosin binding protein cardiac | 0.62 |
|  | C216R094 | UNKNOWN | 0.62 |
|  | C109R148 | atp-dependent rna helicase dhx29 | 0.62 |
|  | C140R037 | transposable element tc1 transposase | 0.61 |
|  | C039R082 | perforin-1-like | 0.61 |
|  | C070R090 | gamma-crystallin m2-like | 0.61 |
|  | C128R158 | n-alpha-acetyltransferase auxiliary subunit | 0.61 |
|  | C084R107 | unhealthy ribosome biogenesis protein 2 homolog | 0.61 |
|  | C034R073 | UNKNOWN | 0.61 |
|  | C099R129 | UNKNOWN | 0.61 |
|  | C013R148 | UNKNOWN | 0.61 |
|  | C172R150 | UNKNOWN | 0.61 |
|  | C074R053 | troponin slow skeletal and cardiac muscles | 0.61 |
|  | C074R089 | rac-alpha serine threonine-protein kinase | 0.61 |
|  | C080R134 | UNKNOWN | 0.61 |
|  | C221R127 | tripartite motif-containing protein 16-like | 0.61 |
|  | C217R034 | transmembrane protein 60 | 0.61 |
|  | C076R033 | UNKNOWN | 0.61 |
|  | C207R139 | glycogen debranching enzyme | 0.61 |
|  | C172R121 | 60s ribosomal protein l7 | 0.61 |
|  | C096R085 | ras-interacting protein 1 | 0.61 |
|  | C236R133 | UNKNOWN | 0.61 |
|  | C161R086 | small subunit processome component 20 homolog | 0.61 |
|  | C157R071 | dna-directed rna polymerase i subunit rpa1 | 0.61 |
|  | C030R134 | traf4-associated factor 1-like | 0.61 |
|  | C062R060 | breast carcinoma-amplified sequence 3 isoform 1 | 0.61 |
|  | C014R145 | UNKNOWN | 0.61 |
|  | C186R157 | 60s ribosomal protein l27 | 0.61 |
|  | C130R051 | prostaglandin e synthase 3 | 0.61 |
|  | C047R144 | e3 ubiquitin-protein ligase trim39-like | 0.61 |
|  | C253R060 | voltage-dependent l-type calcium channel subunit beta-2 | 0.61 |
|  | C162R164 | UNKNOWN | 0.61 |
|  | C126R155 | eukaryotic translation initiation factor 3 subunit a-like | 0.61 |
|  | C216R131 | triadin | 0.61 |
|  | C127R061 | dapper homolog 1 | 0.61 |
|  | C094R138 | b-cell cll lymphoma 7 protein family member a | 0.61 |
|  | C191R074 | neuronal nitric oxide synthase | 0.61 |
|  | C244R062 | UNKNOWN | 0.61 |
|  | C182R034 | si:dkeyp- protein | 0.61 |
|  | C146R145 | UNKNOWN | 0.61 |
|  | C103R160 | polyamine-modulated factor 1 | 0.61 |
|  | C114R103 | UNKNOWN | 0.61 |
|  | C231R152 | butyrate response factor 2 | 0.61 |
|  | C187R119 | tetratricopeptide repeat protein 4-like | 0.61 |
|  | C178R115 | u6 snrna-associated sm-like protein lsm1 | 0.61 |
|  | C063R087 | arachidonate 5-lipoxygenase | 0.61 |
|  | C228R056 | diencephalon mesencephalon homeobox protein 1-b-like isoform 2 | 0.61 |
|  | C253R037 | formin-binding protein 4 | 0.61 |
|  | C182R095 | amp deaminase 1 | 0.61 |
|  | C041R165 | glyceraldehyde-3-phosphate dehydrogenase | 0.61 |
|  | C099R097 | sid1 transmembrane family member 2 | 0.61 |
|  | C076R087 | UNKNOWN | 0.61 |
|  | C007R011 | UNKNOWN | 0.61 |
|  | C015R039 | peptidase inhibitor 16 | 0.61 |
|  | C019R136 | splicing factor 3b subunit 1 | 0.61 |
|  | C002R093 | cyclin-dependent kinase 4 inhibitor b | 0.61 |
|  | C087R093 | smoothelin-like protein 2 | 0.61 |
|  | C056R017 | zinc finger ccch domain-containing protein 18 | 0.61 |
|  | C057R074 | 60s ribosomal protein l36a | 0.61 |
|  | C149R170 | cytochrome c oxidase subunit vib isoform 1 | 0.61 |
|  | C236R112 | UNKNOWN | 0.61 |
|  | C056R114 | heat repeat-containing protein 3 | 0.61 |
|  | C009R107 | rna-directed dna polymerase from mobile element jockey-like | 0.61 |
|  | C150R076 | UNKNOWN | 0.61 |
|  | C260R062 | mitotic checkpoint serine threonine-protein kinase bub1 | 0.61 |
|  | C163R095 | beta-taxilin | 0.61 |
|  | C028R168 | egf-like repeat and discoidin i-like domain-containing protein 3 | 0.61 |
|  | C091R059 | UNKNOWN | 0.61 |
|  | C097R099 | chloride intracellular channel protein 4 | 0.61 |
|  | C204R085 | UNKNOWN | 0.61 |
|  | C207R027 | type iv secretion protein rhs | 0.61 |
|  | C030R125 | hepatocyte growth factor activator-like | 0.61 |
|  | C211R028 | spermatogenesis-associated protein 20 | 0.61 |
|  | C209R049 | UNKNOWN | 0.61 |
|  | C031R102 | ras association domain-containing protein 10-like | 0.61 |
|  | C131R028 | next to brca1 gene 1 protein | 0.61 |
|  | C100R066 | myomesin-1 isoform 1 | 0.61 |
|  | C167R146 | cd83 | 0.61 |
|  | C209R138 | enhancer of rudimentary homolog | 0.61 |
|  | C193R099 | UNKNOWN | 0.61 |
|  | C124R170 | e3 ubiquitin-protein ligase sh3rf2-like isoform x1 | 0.61 |
|  | C264R048 | hyaluronidase-2-like | 0.61 |
|  | C174R060 | sex comb on midleg-like protein 2-like | 0.61 |
|  | C190R009 | UNKNOWN | 0.61 |
|  | C139R011 | cellular tumor antigen p53 | 0.61 |
|  | C194R064 | UNKNOWN | 0.61 |
|  | C144R135 | dna-binding protein rfx2-like | 0.61 |
|  | C077R151 | histone-lysine n-methyltransferase mll3 | 0.60 |
|  | C071R089 | collagen alpha-1 chain-like | 0.60 |
|  | C180R131 | nadh dehydrogenase | 0.60 |
|  | C219R087 | reverse transcriptase-like protein | 0.60 |
|  | C178R116 | ww domain-binding protein 11 | 0.60 |
|  | C168R017 | fras1-related extracellular matrix protein 1 | 0.60 |
|  | C236R164 | UNKNOWN | 0.60 |
|  | C075R111 | acyl- synthetase family member mitochondrial | 0.60 |
|  | C113R162 | short coiled-coil protein | 0.60 |
|  | C156R132 | lish domain and heat repeat-containing protein kiaa1468 homolog | 0.60 |
|  | C091R163 | UNKNOWN | 0.60 |
|  | C130R131 | anaphase-promoting complex subunit 2 | 0.60 |
|  | C234R113 | chromosome-associated kinesin kif4a | 0.60 |
|  | C074R029 | UNKNOWN | 0.60 |
|  | C016R118 | a-kinase anchor protein 9-like isoform x3 | 0.60 |
|  | C110R080 | vcl protein | 0.60 |
|  | C214R103 | dna-directed rna polymerases and iii subunit rpabc4 | 0.60 |
|  | C185R072 | adenylosuccinate lyase | 0.60 |
|  | C208R145 | glutamate-rich wd repeat containing 1 | 0.60 |
|  | C071R044 | UNKNOWN | 0.60 |
|  | C080R090 | m-phase phosphoprotein 9 | 0.60 |
|  | C103R047 | t-cell activation rho gtpase-activating protein | 0.60 |
|  | C179R074 | gmp reductase 1 | 0.60 |
|  | C201R166 | solute carrier family facilitated glucose transporter member 3-like | 0.60 |
|  | C139R124 | UNKNOWN | 0.60 |
|  | C266R039 | UNKNOWN | 0.60 |
|  | C121R053 | kelch repeat and btb domain-containing protein 12 | 0.60 |
|  | C190R100 | UNKNOWN | 0.60 |
|  | C164R145 | enhancer of zeste homolog 2 (enx-1) isoform cra_a | 0.60 |
|  | C228R110 | calcium-transporting atpase sarcoplasmic endoplasmic reticulum type (calcium pump) | 0.60 |
|  | C061R021 | protein | 0.60 |
|  | C177R095 | novel protein human titin | 0.60 |
|  | C204R062 | n-sulphoglucosamine sulphohydrolase precursor | 0.60 |
|  | C020R081 | troponin slow skeletal muscle-like | 0.60 |
|  | C030R158 | gamma-interferon-inducible lysosomal thiol reductase precursor | 0.60 |
|  | C188R158 | transposable element tcb1 transposase | 0.60 |
|  | C171R097 | lws opsin | 0.60 |
|  | C232R031 | UNKNOWN | 0.60 |
|  | C131R120 | calumenin isoform 1 | 0.60 |
|  | C185R066 | zgc:158564 protein | 0.60 |
|  | C189R138 | nicotinamide n-methyltransferase | 0.60 |
|  | C012R154 | inactive serine protease 35 | 0.60 |
|  | C032R015 | calmodulin-like protein 4 | 0.60 |
|  | C089R082 | UNKNOWN | 0.60 |
|  | C243R102 | g-protein coupled receptor 182 | 0.60 |
|  | C084R035 | UNKNOWN | 0.60 |
|  | C150R052 | 40s ribosomal protein s10 | 0.60 |
|  | C071R037 | nadh dehydrogenase | 0.60 |
|  | C225R028 | transcription factor dp-1 | 0.60 |
|  | C144R046 | 60s ribosomal protein l12 | 0.60 |
|  | C265R102 | UNKNOWN | 0.60 |
|  | C004R091 | histone-lysine n-methyltransferase prdm9-like | 0.60 |
|  | C011R035 | apolipoprotein b-100-like | 0.60 |
|  | C023R035 | protein | 0.60 |
|  | C094R108 | adenomatous polyposis coli protein | 0.60 |
|  | C249R008 | voltage-dependent n-type calcium channel subunit alpha-1b-like | 0.60 |
|  | C055R090 | rna-binding protein 38-like | 0.60 |
|  | C057R167 | golgi ph regulator-like | 0.60 |
|  | C094R011 | rna-binding protein 45 | 0.60 |
|  | C005R117 | atp-dependent clp protease proteolytic mitochondrial | 0.60 |
|  | C168R135 | nadh dehydrogenase | 0.60 |
|  | C114R045 | pancreatic secretory granule membrane major glycoprotein gp2 precursor | 0.60 |
|  | C154R022 | non-syndromic hearing impairment protein 5 | 0.60 |
|  | C217R029 | UNKNOWN | 0.60 |
|  | C210R102 | sorbin and sh3 domain-containing protein 2 isoform 5 | 0.60 |
|  | C238R022 | histone-arginine methyltransferase carm1-like | 0.60 |
|  | C156R013 | nuclear transport factor 2 | 0.60 |
|  | C110R162 | acidic leucine-rich nuclear phosphoprotein 32 family member b | 0.59 |
|  | C264R121 | chymotrypsin-like elastase family member 2a-like | 0.59 |
|  | C051R158 | myoferlin-like isoform x2 | 0.59 |
|  | C060R030 | glia maturation factor beta | 0.59 |
|  | C066R060 | schwannomin-interacting protein 1 | 0.59 |
|  | C076R148 | alpha-internexin-like | 0.59 |
|  | C177R131 | ddb1- and cul4-associated factor 10 | 0.59 |
|  | C265R142 | sjchgc04011 protein | 0.59 |
|  | C119R129 | UNKNOWN | 0.59 |
|  | C250R108 | minor histocompatibility protein ha-1-like | 0.59 |
|  | C142R042 | adp-ribosylation factor-related protein 1 | 0.59 |
|  | C248R107 | UNKNOWN | 0.59 |
|  | C070R111 | h(+) cl(-) exchange transporter 5 | 0.59 |
|  | C085R023 | camp-regulated phosphoprotein 19 | 0.59 |
|  | C104R068 | myozenin 1 | 0.59 |
|  | C185R035 | hydroxyacid-oxoacid mitochondrial | 0.59 |
|  | C217R122 | ras gtpase-activating-like protein iqgap1-like | 0.59 |
|  | C016R010 | serine threonine-protein kinase sgk1 | 0.59 |
|  | C238R091 | acyl- dehydrogenase -like | 0.59 |
|  | C187R111 | gtp-binding protein sar1b | 0.59 |
|  | C200R114 | transitional endoplasmic reticulum atpase-like | 0.59 |
|  | C170R069 | 4-aminobutyrate mitochondrial | 0.59 |
|  | C038R141 | nadh dehydrogenase | 0.59 |
|  | C129R069 | transposase | 0.59 |
|  | C189R165 | UNKNOWN | 0.59 |
|  | C042R065 | UNKNOWN | 0.59 |
|  | C012R050 | small glutamine-rich tetratricopeptide repeat-containing protein alpha | 0.59 |
|  | C168R019 | krueppel-like factor 11-like | 0.59 |
|  | C173R096 | pdlim7 protein | 0.59 |
|  | C076R147 | e3 ubiquitin-protein ligase rnf31-like | 0.59 |
|  | C022R010 | zinc finger protein 319-like | 0.59 |
|  | C174R020 | UNKNOWN | 0.59 |
|  | C199R082 | hypothetical protein M91_01401, partial | 0.59 |
|  | C133R159 | tgf-beta-activated kinase 1 and map3k7-binding protein 2 | 0.59 |
|  | C005R123 | sjchgc09650 protein | 0.59 |
|  | C182R120 | loc734178 protein | 0.59 |
|  | C129R030 | UNKNOWN | 0.59 |
|  | C040R052 | amp deaminase 1 | 0.59 |
|  | C115R099 | sorting and assembly machinery component 50 homolog | 0.59 |
|  | C194R123 | neutral cholesterol ester hydrolase 1-like | 0.59 |
|  | C051R079 | late secretory pathway protein avl9 homolog | 0.59 |
|  | C149R068 | UNKNOWN | 0.59 |
|  | C151R105 | UNKNOWN | 0.59 |
|  | C131R145 | ets domain-containing protein elk-3 | 0.59 |
|  | C044R033 | UNKNOWN | 0.59 |
|  | C083R154 | transforming growth factor beta regulator 1 | 0.59 |
|  | C104R134 | kruppel-like factor 4 | 0.59 |
|  | C132R054 | uroporphyrinogen decarboxylase | 0.59 |
|  | C073R167 | UNKNOWN | 0.59 |
|  | C059R085 | neutrophil cytosolic factor 4 | 0.59 |
|  | C169R059 | dnaj homolog subfamily c member 25-like | 0.59 |
|  | C144R152 | UNKNOWN | 0.59 |
|  | C121R059 | krueppel-like factor 12-like | 0.59 |
|  | C055R083 | PREDICTED: hypothetical protein LOC557772 | 0.59 |
|  | C108R021 | UNKNOWN | 0.59 |
|  | C086R151 | sulfotransferase 6b1 | 0.59 |
|  | C200R037 | creatine mitochondrial 1b | 0.59 |
|  | C155R081 | tripartite motif-containing protein 45-like | 0.59 |
|  | C148R126 | synapsin 2a | 0.59 |
|  | C225R096 | UNKNOWN | 0.59 |
|  | C079R025 | p2x purinoceptor 3 | 0.59 |
|  | C247R129 | nucleoprotein tpr | 0.59 |
|  | C177R125 | protocadherin-18-like isoform x1 | 0.59 |
|  | C136R016 | oligosaccharyltransferase complex subunit ostc-like | 0.59 |
|  | C066R037 | thrombospondin-3 isoform 3 | 0.59 |
|  | C055R170 | ephrin type-b receptor partial | 0.59 |
|  | C126R062 | cytochrome c oxidase subunit vib isoform 1 | 0.59 |
|  | C093R051 | PREDICTED: uncharacterized protein LOC101168094 | 0.59 |
|  | C138R066 | UNKNOWN | 0.59 |
|  | C076R028 | UNKNOWN | 0.59 |
|  | C201R035 | pre-mrna branch site protein p14-like | 0.59 |
|  | C058R057 | retinoid x beta | 0.59 |
|  | C006R120 | protein-tyrosine kinase 2-beta isoform 2 | 0.59 |
|  | C200R167 | alstrom syndrome protein 1 | 0.59 |
|  | C238R078 | proteoglycan 4 | 0.59 |
|  | C120R164 | UNKNOWN | 0.58 |
|  | C058R127 | desmin | 0.58 |
|  | C205R127 | nadh dehydrogenase subunit 2 | 0.58 |
|  | C104R023 | protein rrnad1-like | 0.58 |
|  | C041R087 | calcium-binding protein | 0.58 |
|  | C148R098 | conserved oligomeric golgi complex subunit partial | 0.58 |
|  | C161R083 | UNKNOWN | 0.58 |
|  | C159R149 | tnf receptor-associated factor 4 | 0.58 |
|  | C139R168 | UNKNOWN | 0.58 |
|  | C108R052 | microtubule-associated proteins 1a 1b light chain 3a | 0.58 |
|  | C165R019 | sodium channel subunit beta-1-like | 0.58 |
|  | C065R041 | oocyte zinc finger protein 6-like | 0.58 |
|  | C158R129 | PREDICTED: uncharacterized protein LOC101472525 | 0.58 |
|  | C042R086 | cytochrome c oxidase subunit vib isoform 1 | 0.58 |
|  | C085R022 | cwf19-like protein 2 | 0.58 |
|  | C033R136 | set and mynd domain-containing protein 5 | 0.58 |
|  | C125R021 | PREDICTED: uncharacterized protein LOC101485590 | 0.58 |
|  | C172R148 | tripartite motif-containing protein 16-like | 0.58 |
|  | C229R059 | probable g-protein coupled receptor 125 | 0.58 |
|  | C192R149 | UNKNOWN | 0.58 |
|  | C095R079 | UNKNOWN | 0.58 |
|  | C198R157 | UNKNOWN | 0.58 |
|  | C232R021 | zinc finger protein 207 | 0.58 |
|  | C148R028 | fatty-acid amide hydrolase 2 | 0.58 |
|  | C187R071 | transferrin receptor protein 1-like | 0.58 |
|  | C235R036 | ribosomal l1 domain-containing protein 1-like | 0.58 |
|  | C258R062 | transposase | 0.58 |
|  | C085R049 | UNKNOWN | 0.58 |
|  | C099R162 | gastrula zinc finger protein | 0.58 |
|  | C133R122 | UNKNOWN | 0.58 |
|  | C135R074 | kelch repeat and btb domain-containing protein 10-like | 0.58 |
|  | C138R118 | nadph--cytochrome p450 reductase | 0.58 |
|  | C254R100 | UNKNOWN | 0.58 |
|  | C178R002 | protein fam53b-like | 0.58 |
|  | C214R141 | UNKNOWN | 0.58 |
|  | C017R077 | UNKNOWN | 0.58 |
|  | C021R116 | forkhead box protein p4 | 0.58 |
|  | C031R065 | riken cdna 2300009a05 gene | 0.58 |
|  | C114R054 | ubiquitin-conjugating enzyme e2 g1 | 0.58 |
|  | C227R140 | dna polymerase subunit gamma- mitochondrial | 0.58 |
|  | C066R027 | PREDICTED: hypothetical protein LOC560439 | 0.58 |
|  | C086R039 | loc799552 protein | 0.58 |
|  | C078R089 | UNKNOWN | 0.58 |
|  | C207R167 | protein vac14 homolog | 0.58 |
|  | C061R057 | transmembrane gamma-carboxyglutamic acid protein 4-like | 0.58 |
|  | C110R010 | actin-related protein 2 3 complex subunit 2 | 0.58 |
|  | C208R049 | predicted protein | 0.58 |
|  | C159R010 | transketolase-like protein 2 | 0.58 |
|  | C129R129 | tartrate-resistant acid phosphatase type 5 | 0.58 |
|  | C112R060 | cdk8 protein | 0.58 |
|  | C129R126 | transposase | 0.58 |
|  | C085R159 | UNKNOWN | 0.58 |
|  | C085R103 | UNKNOWN | 0.58 |
|  | C155R170 | homeobox protein hox-b2a-like | 0.58 |
|  | C129R052 | ras-related protein rab-11b | 0.58 |
|  | C109R088 | adenylate kinase | 0.58 |
|  | C157R028 | plexin-d1 | 0.58 |
|  | C125R108 | protein | 0.58 |
|  | C040R169 | sec1 family domain-containing protein 2 | 0.58 |
|  | C077R044 | UNKNOWN | 0.58 |
|  | C102R072 | selectin e | 0.58 |
|  | C252R113 | atp-binding cassette sub-family d member 3 isoform 2 | 0.58 |
|  | C261R094 | platelet-derived growth factor receptor beta | 0.58 |
|  | C138R160 | lim-domain binding factor 3 | 0.58 |
|  | C079R070 | dna topoisomerase 2-binding protein 1 | 0.58 |
|  | C129R093 | uncharacterized protein LOC100191015 | 0.57 |
|  | C063R083 | dna polymerase subunit gamma-1 | 0.57 |
|  | C014R074 | UNKNOWN | 0.57 |
|  | C250R124 | u2 snrnp-associated surp motif-containing protein | 0.57 |
|  | C144R120 | lymphatic vessel endothelial hyaluronic acid receptor 1 | 0.57 |
|  | C130R148 | monoacylglycerol lipase abhd6-like | 0.57 |
|  | C044R116 | cytochrome c oxidase subunit vib isoform 1 | 0.57 |
|  | C145R069 | mitogen-activated protein kinase 7-like | 0.57 |
|  | C121R129 | 60s ribosome subunit biogenesis protein nip7 homolog | 0.57 |
|  | C113R106 | UNKNOWN | 0.57 |
|  | C099R128 | mitochondrial import receptor subunit tom6 homolog | 0.57 |
|  | C163R036 | UNKNOWN | 0.57 |
|  | C115R025 | probable e3 ubiquitin-protein ligase partial | 0.57 |
|  | C046R150 | heat shock protein beta-1 | 0.57 |
|  | C001R046 | UNKNOWN | 0.57 |
|  | C164R107 | cop9 signalosome complex subunit 5 | 0.57 |
|  | C257R041 | UNKNOWN | 0.57 |
|  | C214R111 | UNKNOWN | 0.57 |
|  | C258R042 | UNKNOWN | 0.57 |
|  | C262R032 | vesicle transport protein sft2a | 0.57 |
|  | C048R072 | wd repeat-containing protein 44 | 0.57 |
|  | C118R080 | protein polybromo-1 | 0.57 |
|  | C220R133 | short transient receptor potential channel 1 | 0.57 |
|  | C052R132 | transposable element tcb1 transposase | 0.57 |
|  | C209R093 | tyrosine-protein kinase transmembrane receptor ror2 | 0.57 |
|  | C043R098 | proline-rich protein 5 | 0.57 |
|  | C120R016 | liprin-beta-2- partial | 0.57 |
|  | C187R009 | clathrin coat assembly protein ap180 | 0.57 |
|  | C171R079 | UNKNOWN | 0.57 |
|  | C106R090 | high choriolytic enzyme 1 precursor | 0.57 |
|  | C017R024 | p2y purinoceptor 8 | 0.57 |
|  | C239R104 | kinesin-like protein kif20b-like | 0.57 |
|  | C128R064 | 39s ribosomal protein mitochondrial-like | 0.57 |
|  | C178R075 | myosin light chain 4-like | 0.57 |
|  | C031R137 | inhibitor of growth protein 4 | 0.57 |
|  | C077R149 | lysine-specific demethylase 4b | 0.57 |
|  | C212R099 | e3 ubiquitin-protein ligase trim39-like | 0.57 |
|  | C094R122 | beta-2-glycoprotein 1-like | 0.57 |
|  | C104R137 | transmembrane protein 120a | 0.57 |
|  | C029R101 | a chain molecular basis of histone h3k36me3 recognition by the pwwp domain of | 0.57 |
|  | C028R067 | cell surface protein | 0.57 |
|  | C040R084 | UNKNOWN | 0.57 |
|  | C133R031 | heme oxygenase | 0.57 |
|  | C217R059 | UNKNOWN | 0.57 |
|  | C160R024 | UNKNOWN | 0.57 |
|  | C214R048 | pdz domain containing 2-like | 0.57 |
|  | C163R062 | cytochrome p450 2u1-like | 0.57 |
|  | C209R137 | UNKNOWN | 0.57 |
|  | C084R013 | cleavage and polyadenylation specificity factor subunit 7 | 0.57 |
|  | C026R111 | UNKNOWN | 0.57 |
|  | C062R112 | phosphatidylinositol- -bisphosphate 3-kinase catalytic subunit gamma isoform-like | 0.57 |
|  | C017R148 | vesicle-fusing atpase | 0.57 |
|  | C020R126 | dual specificity protein kinase ttk | 0.57 |
|  | C099R114 | beta-soluble nsf attachment protein | 0.57 |
|  | C229R050 | drebrin 1 | 0.57 |
|  | C264R084 | protein fam117a-like | 0.56 |
|  | C013R153 | gdp-man:man c -pp-dol alpha- -mannosyltransferase-like | 0.56 |
|  | C078R124 | zinc finger and btb domain-containing protein 10 | 0.56 |
|  | C014R156 | methyltransferase-like protein 16 | 0.56 |
|  | C185R140 | nitrogen permease regulator 3-like | 0.56 |
|  | C135R063 | zinc finger protein 135-like | 0.56 |
|  | C230R162 | cofilin-2 | 0.56 |
|  | C162R016 | bcl2 adenovirus e1b 19 kda protein-interacting protein 3-like | 0.56 |
|  | C158R076 | 40s ribosomal protein s27 | 0.56 |
|  | C168R081 | high mobility group protein b1 | 0.56 |
|  | C012R120 | UNKNOWN | 0.56 |
|  | C157R010 | protein | 0.56 |
|  | C149R069 | probable helicase with zinc finger domain | 0.56 |
|  | C063R169 | dna mismatch repair protein msh2-like | 0.56 |
|  | C198R165 | UNKNOWN | 0.56 |
|  | C067R080 | thrombomodulin | 0.56 |
|  | C072R011 | UNKNOWN | 0.56 |
|  | C146R093 | UNKNOWN | 0.56 |
|  | C258R049 | heterogeneous nuclear ribonucleoprotein k | 0.56 |
|  | C157R130 | UNKNOWN | 0.56 |
|  | C052R059 | UNKNOWN | 0.56 |
|  | C187R061 | nadh dehydrogenase | 0.56 |
|  | C129R050 | cell division cycle protein 27 homolog isoform 1 | 0.56 |
|  | C076R035 | tho complex subunit 6 homolog | 0.56 |
|  | C174R114 | UNKNOWN | 0.56 |
|  | C165R015 | methionine aminopeptidase 1 | 0.56 |
|  | C002R053 | interferon-induced protein 44-like | 0.56 |
|  | C073R095 | dnaj homolog subfamily c member 7 isoform 2 | 0.56 |
|  | C102R085 | UNKNOWN | 0.56 |
|  | C112R158 | protein | 0.56 |
|  | C070R080 | serine threonine-protein kinase 38 | 0.56 |
|  | C112R149 | n-acetylated-alpha-linked acidic dipeptidase 2 | 0.56 |
|  | C094R024 | tyrosine-protein kinase csk | 0.56 |
|  | C156R042 | dna-directed rna polymerases and iii subunit rpabc4 | 0.56 |
|  | C036R151 | UNKNOWN | 0.56 |
|  | C074R025 | gonadotropin-releasing hormone | 0.56 |
|  | C055R091 | UNKNOWN | 0.56 |
|  | C189R154 | triadin | 0.56 |
|  | C246R100 | microcephalin | 0.56 |
|  | C007R093 | UNKNOWN | 0.56 |
|  | C057R027 | UNKNOWN | 0.56 |
|  | C156R153 | eukaryotic translation initiation factor 4b | 0.56 |
|  | C075R043 | UNKNOWN | 0.56 |
|  | C234R061 | 60s acidic ribosomal protein p2 | 0.56 |
|  | C016R094 | UNKNOWN | 0.56 |
|  | C112R141 | f-box wd repeat-containing protein 11 isoform 2 | 0.56 |
|  | C166R128 | methionine aminopeptidase 1 | 0.56 |
|  | C094R124 | UNKNOWN | 0.56 |
|  | C209R151 | sarcoplasmic endoplasmic reticulum calcium atpase 1-like | 0.56 |
|  | C091R122 | hypothetical protein GLRG_11022 | 0.56 |
|  | C157R045 | wiskott-aldrich syndrome protein family member 3 | 0.56 |
|  | C215R147 | cytochrome c oxidase subunit 3 | 0.56 |
|  | C106R023 | inositol -triphosphate receptor-interacting | 0.56 |
|  | C142R116 | hepatocyte cell adhesion molecule | 0.56 |
|  | C123R169 | tripartite motif-containing protein 16-like | 0.56 |
|  | C116R036 | UNKNOWN | 0.56 |
|  | C164R017 | UNKNOWN | 0.56 |
|  | C002R098 | UNKNOWN | 0.56 |
|  | C098R125 | acylphosphatase-1 isoform x2 | 0.56 |
|  | C173R018 | phosphoribosyl pyrophosphate synthetase-associated protein 1 | 0.56 |
|  | C233R074 | UNKNOWN | 0.56 |
|  | C232R067 | protein ssuh2 homolog | 0.56 |
|  | C254R151 | isotocin precursor | 0.56 |
|  | C124R018 | wd repeat-containing protein 67 | 0.56 |
|  | C160R064 | PREDICTED: hypothetical protein LOC100697841 | 0.56 |
|  | C187R133 | methionine synthase reductase | 0.55 |
|  | C026R046 | UNKNOWN | 0.55 |
|  | C010R120 | cytochrome b-c1 complex subunit mitochondrial precursor | 0.55 |
|  | C045R036 | 5-phosphohydroxy-l-lysine phospho-lyase-like | 0.55 |
|  | C130R012 | cathepsin s precursor | 0.55 |
|  | C123R011 | p2y purinoceptor 8 | 0.55 |
|  | C078R080 | dual specificity testis-specific protein kinase 1-like | 0.55 |
|  | C156R106 | UNKNOWN | 0.55 |
|  | C175R023 | rna binding protein fox-1 homolog 1-like | 0.55 |
|  | C123R129 | fish virus induced trim protein | 0.55 |
|  | C033R068 | integrin-linked kinase-associated serine threonine phosphatase 2c | 0.55 |
|  | C128R021 | PREDICTED: uncharacterized protein LOC101467272 isoform X1 | 0.55 |
|  | C058R016 | stromal cell-derived factor 1 precursor | 0.55 |
|  | C161R120 | protein fam69b-like | 0.55 |
|  | C176R014 | obscurin | 0.55 |
|  | C139R076 | UNKNOWN | 0.55 |
|  | C009R071 | UNKNOWN | 0.55 |
|  | C128R056 | UNKNOWN | 0.55 |
|  | C030R147 | UNKNOWN | 0.55 |
|  | C241R010 | t-cell surface glycoprotein cd3 zeta chain precursor | 0.55 |
|  | C228R010 | integrator complex subunit 4 | 0.55 |
|  | C130R145 | 3-oxo-5-alpha-steroid 4-dehydrogenase 2 | 0.55 |
|  | C260R058 | transmembrane protein 88-like | 0.55 |
|  | C169R127 | UNKNOWN | 0.55 |
|  | C251R131 | serine threonine-protein kinase greatwall-like | 0.55 |
|  | C034R076 | transgelin | 0.55 |
|  | C073R004 | UNKNOWN | 0.55 |
|  | C103R102 | epoxide hydrolase 1-like | 0.55 |
|  | C254R095 | matrix metalloproteinase-23-like | 0.55 |
|  | C070R039 | probable udp-sugar transporter protein slc35a5 | 0.55 |
|  | C236R107 | traf-type zinc finger domain-containing protein 1 | 0.55 |
|  | C205R170 | nebulin-related-anchoring protein | 0.55 |
|  | C217R119 | telomeric repeat-binding factor 2 | 0.55 |
|  | C002R056 | UNKNOWN | 0.55 |
|  | C013R158 | set and mynd domain-containing protein 1-like isoform 2 | 0.55 |
|  | C007R067 | zinc finger protein 678 | 0.55 |
|  | C180R168 | transposable element tcb2 transposase | 0.55 |
|  | C121R146 | cell division cycle 7-related protein kinase | 0.55 |
|  | C163R131 | myogenin | 0.55 |
|  | C219R080 | neurofilament light polypeptide | 0.55 |
|  | C129R096 | poly -specific endoribonuclease-c-like | 0.55 |
|  | C034R031 | mucosa-associated lymphoid tissue lymphoma translocation protein 1 isoform 2 | 0.55 |
|  | C024R022 | endonuclease domain-containing 1 | 0.55 |
|  | C169R116 | slain motif-containing | 0.55 |
|  | C245R046 | mucosa-associated lymphoid tissue lymphoma translocation protein 1 isoform 2 | 0.55 |
|  | C246R074 | zinc finger protein 51 | 0.55 |
|  | C079R043 | slam family member 8 | 0.55 |
|  | C266R145 | UNKNOWN | 0.55 |
|  | C199R090 | UNKNOWN | 0.55 |
|  | C211R088 | UNKNOWN | 0.55 |
|  | C226R167 | zinc finger protein 271-like | 0.55 |
|  | C116R051 | wd repeat-containing protein 82-like | 0.55 |
|  | C183R097 | UNKNOWN | 0.55 |
|  | C029R088 | cd9 antigen | 0.55 |
|  | C152R018 | zinc finger and scan domain-containing protein 29-like | 0.55 |
|  | C020R065 | ras-related c3 botulinum toxin substrate 2 | 0.55 |
|  | C181R072 | filamin a-interacting protein 1-like | 0.55 |
|  | C197R120 | cg050 protein | 0.55 |
|  | C128R072 | protein hexim-like | 0.55 |
|  | C056R009 | protein qn1 homolog | 0.55 |
|  | C181R169 | protein fam65c | 0.55 |
|  | C112R081 | e3 ubiquitin-protein ligase rnf130 | 0.55 |
|  | C207R092 | nuclear transport factor 2 | 0.55 |
|  | C131R020 | 60s ribosomal protein l32 | 0.54 |
|  | C099R099 | fibronectin-like isoform 2 | 0.54 |
|  | C143R125 | serine threonine-protein kinase 17a-like | 0.54 |
|  | C061R160 | UNKNOWN | 0.54 |
|  | C131R106 | UNKNOWN | 0.54 |
|  | C111R089 | basement membrane-specific heparan sulfate proteoglycan core | 0.54 |
|  | C122R121 | tyrosyl-dna phosphodiesterase 2-like | 0.54 |
|  | C261R136 | heat shock factor-binding protein 1 | 0.54 |
|  | C178R102 | UNKNOWN | 0.54 |
|  | C019R045 | UNKNOWN | 0.54 |
|  | C065R034 | UNKNOWN | 0.54 |
|  | C211R135 | UNKNOWN | 0.54 |
|  | C071R154 | dna-directed rna polymerases and iii subunit rpabc4 | 0.54 |
|  | C122R101 | UNKNOWN | 0.54 |
|  | C240R120 | UNKNOWN | 0.54 |
|  | C059R035 | UNKNOWN | 0.54 |
|  | C091R133 | nicotinamide mononucleotide adenylyltransferase 1 | 0.54 |
|  | C131R016 | lissencephaly-1 homolog b | 0.54 |
|  | C249R105 | protein | 0.54 |
|  | C180R119 | ubiquitin-conjugating enzyme e2 k | 0.54 |
|  | C019R098 | fam32a-like | 0.54 |
|  | C223R044 | non-ltr retrotransposable element | 0.54 |
|  | C169R012 | UNKNOWN | 0.54 |
|  | C188R083 | UNKNOWN | 0.54 |
|  | C100R021 | protein | 0.54 |
|  | C204R114 | polypeptide n-acetylgalactosaminyltransferase 6 | 0.54 |
|  | C192R008 | UNKNOWN | 0.54 |
|  | C047R156 | 28s ribosomal protein mitochondrial-like | 0.54 |
|  | C131R110 | cold shock domain-containing protein e1 | 0.54 |
|  | C164R038 | y-box-binding protein 2-a | 0.54 |
|  | C257R110 | snf-related serine threonine-protein kinase | 0.54 |
|  | C037R146 | rab gdp dissociation inhibitor beta | 0.54 |
|  | C053R045 | keratin-associated protein 10-4-like | 0.54 |
|  | C185R056 | UNKNOWN | 0.54 |
|  | C263R100 | xin actin-binding repeat-containing protein 1 | 0.54 |
|  | C103R075 | zinc finger protein 709-like | 0.54 |
|  | C151R043 | 60s ribosomal protein l6 | 0.54 |
|  | C096R164 | rna binding motif protein | 0.54 |
|  | C123R155 | hhip-like protein 1 | 0.54 |
|  | C218R051 | neutrophil cytosolic factor 1 | 0.54 |
|  | C015R089 | UNKNOWN | 0.54 |
|  | C007R139 | chemokine ck-1 | 0.54 |
|  | C019R035 | f-box wd repeat-containing protein 5 | 0.54 |
|  | C012R009 | lysm and peptidoglycan-binding domain-containing protein 3-like | 0.54 |
|  | C165R114 | UNKNOWN | 0.54 |
|  | C249R168 | ogdh protein | 0.54 |
|  | C157R006 | solute carrier family 43 member 3-like | 0.54 |
|  | C175R101 | UNKNOWN | 0.54 |
|  | C153R037 | alpha cardiac muscle 1 | 0.54 |
|  | C224R013 | forkhead box protein n3-like | 0.54 |
|  | C031R135 | bcl-2-like protein 12 | 0.54 |
|  | C087R037 | cnot1 protein | 0.54 |
|  | C128R018 | UNKNOWN | 0.54 |
|  | C168R151 | zinc finger protein 318 | 0.54 |
|  | C051R028 | mkl myocardin-like protein 1 | 0.54 |
|  | C170R071 | hyccin | 0.54 |
|  | C244R099 | UNKNOWN | 0.54 |
|  | C103R046 | sjchgc03012 protein | 0.54 |
|  | C073R120 | solute carrier family 12 member 9-like | 0.54 |
|  | C062R061 | UNKNOWN | 0.54 |
|  | C077R043 | tgf-beta-activated kinase 1 and map3k7-binding protein 2 | 0.54 |
|  | C167R014 | PREDICTED: uncharacterized protein LOC101486179 | 0.54 |
|  | C183R052 | spectrin beta non-erythrocytic 1-like | 0.54 |
|  | C053R106 | guanine nucleotide exchange factor for rab-3a-like | 0.53 |
|  | C038R147 | UNKNOWN | 0.53 |
|  | C266R068 | malcavernin-like protein | 0.53 |
|  | C123R085 | UNKNOWN | 0.53 |
|  | C063R072 | gdp-man:man c -pp-dol alpha- -mannosyltransferase-like | 0.53 |
|  | C080R026 | beta- -galactosyltransferase 2 | 0.53 |
|  | C112R143 | decorin precursor | 0.53 |
|  | C219R038 | single-stranded dna-binding protein 2 isoform 1 | 0.53 |
|  | C236R043 | interferon inducible mx protein | 0.53 |
|  | C198R124 | leucine-rich repeat-containing protein 16c-like | 0.53 |
|  | C008R086 | zinc finger protein 135-like | 0.53 |
|  | C142R020 | UNKNOWN | 0.53 |
|  | C067R137 | UNKNOWN | 0.53 |
|  | C256R075 | sarcosine mitochondrial | 0.53 |
|  | C161R076 | UNKNOWN | 0.53 |
|  | C237R170 | msx2-interacting protein | 0.53 |
|  | C089R008 | myosin-binding protein fast-type-like | 0.53 |
|  | C130R018 | zinc transporter zip9 | 0.53 |
|  | C100R058 | deoxyribonuclease gamma precursor | 0.53 |
|  | C163R133 | UNKNOWN | 0.53 |
|  | C111R080 | tumor necrosis factor ligand superfamily member 13b | 0.53 |
|  | C137R035 | very long-chain specific acyl- mitochondrial isoform 1 | 0.53 |
|  | C030R117 | UNKNOWN | 0.53 |
|  | C066R097 | nicotinamide riboside kinase 2 | 0.53 |
|  | C149R067 | selenoprotein p | 0.53 |
|  | C173R066 | muscle-related coiled-coil | 0.53 |
|  | C254R080 | glucocorticoid-induced transcript 1 protein | 0.53 |
|  | C084R051 | myomesin 2 | 0.53 |
|  | C023R130 | collagen-like protein 2 | 0.53 |
|  | C249R065 | ankyrin repeat and socs box protein 14-like | 0.53 |
|  | C061R022 | plexin-d1 | 0.53 |
|  | C063R064 | transposable element tcb1 transposase | 0.53 |
|  | C246R044 | chromosome transmission fidelity protein 8 homolog | 0.53 |
|  | C076R098 | UNKNOWN | 0.53 |
|  | C129R074 | 60s ribosomal protein l22 | 0.53 |
|  | C229R105 | UNKNOWN | 0.53 |
|  | C055R093 | semaphorin-7a-like isoform x2 | 0.53 |
|  | C120R022 | nadh dehydrogenase subunit 1 | 0.53 |
|  | C095R019 | 60s ribosomal protein l7 | 0.53 |
|  | C183R073 | h-2 class ii histocompatibility antigen gamma chain | 0.53 |
|  | C048R157 | lymphatic vessel endothelial hyaluronic acid receptor 1 | 0.53 |
|  | C003R102 | zinc finger fyve domain-containing protein 16 | 0.53 |
|  | C161R016 | nadh-cytochrome b5 reductase 2-like | 0.53 |
|  | C121R158 | protein lsm12 homolog | 0.53 |
|  | C104R116 | nascent polypeptide-associated complex subunit alpha | 0.53 |
|  | C048R140 | cadherin-18-like | 0.53 |
|  | C240R164 | UNKNOWN | 0.53 |
|  | C251R154 | protein prune homolog 2-like | 0.53 |
|  | C047R128 | protein apcdd1-like | 0.53 |
|  | C191R115 | proteasome activator complex subunit 4 | 0.53 |
|  | C234R021 | lymphocyte cytosolic protein 2 | 0.53 |
|  | C007R142 | bone morphogenetic protein 2 | 0.53 |
|  | C076R015 | UNKNOWN | 0.53 |
|  | C059R056 | e3 ubiquitin-protein ligase hectd1 | 0.53 |
|  | C232R104 | UNKNOWN | 0.53 |
|  | C257R068 | cytochrome c oxidase assembly protein 1 homolog | 0.52 |
|  | C146R035 | condensin-2 complex subunit g2 | 0.52 |
|  | C141R102 | UNKNOWN | 0.52 |
|  | C104R117 | serine arginine-rich splicing factor 4 | 0.52 |
|  | C002R119 | UNKNOWN | 0.52 |
|  | C003R091 | UNKNOWN | 0.52 |
|  | C061R157 | cyclin-dependent kinase 13-like | 0.52 |
|  | C153R133 | UNKNOWN | 0.52 |
|  | C222R023 | UNKNOWN | 0.52 |
|  | C057R088 | deoxyribonuclease gamma | 0.52 |
|  | C119R109 | serine-protein kinase atm | 0.52 |
|  | C094R045 | neuroendocrine convertase 2 precursor | 0.52 |
|  | C121R038 | UNKNOWN | 0.52 |
|  | C063R091 | homologous-pairing protein 2 homolog | 0.52 |
|  | C234R074 | UNKNOWN | 0.52 |
|  | C233R153 | glycylpeptide n-tetradecanoyltransferase 2-like | 0.52 |
|  | C018R059 | potassium voltage-gated channel subfamily c member 3-like isoform x2 | 0.52 |
|  | C084R029 | cadherin-1 isoform 1 | 0.52 |
|  | C162R073 | UNKNOWN | 0.52 |
|  | C163R081 | UNKNOWN | 0.52 |
|  | C152R011 | collagen alpha-1 chain | 0.52 |
|  | C191R109 | hemoglobin subunit beta-1 | 0.52 |
|  | C101R065 | mycbp-associated protein | 0.52 |
|  | C147R057 | alpha cardiac muscle 1 | 0.52 |
|  | C064R102 | protein memo1 | 0.52 |
|  | C188R033 | zinc finger and scan domain-containing protein 25-like isoform x1 | 0.52 |
|  | C131R062 | vitamin k epoxide reductase subunit 1-like 1 | 0.52 |
|  | C151R082 | peroxisomal bifunctional enzyme | 0.52 |
|  | C029R028 | adenylosuccinate lyase | 0.52 |
|  | C073R023 | cysteine and glycine-rich protein 3 | 0.52 |
|  | C129R079 | UNKNOWN | 0.52 |
|  | C234R078 | transcription factor hes-1 | 0.52 |
|  | C260R112 | novel protein | 0.52 |
|  | C122R064 | homeobox protein mox-2 | 0.52 |
|  | C014R031 | sorting nexin-18 | 0.52 |
|  | C131R031 | UNKNOWN | 0.51 |
|  | C144R039 | apoptosis-related protein 3-like | 0.51 |
|  | C101R113 | UNKNOWN | 0.51 |
|  | C255R109 | heme-binding protein 2 | 0.51 |
|  | C030R008 | phosphotriesterase-related protein | 0.51 |
|  | C238R023 | UNKNOWN | 0.51 |
|  | C118R014 | t-complex protein 1 subunit theta | 0.51 |
|  | C070R143 | creb atf bzip transcription factor | 0.51 |
|  | C047R124 | mesencephalic astrocyte-derived neurotrophic factor-like | 0.51 |
|  | C126R030 | serine threonine-protein phosphatase 2a regulatory subunit b subunit gamma | 0.51 |
|  | C137R109 | protein fam193b-like | 0.51 |
|  | C211R073 | dna-binding partial | 0.51 |
|  | C213R078 | UNKNOWN | 0.51 |
|  | C256R061 | UNKNOWN | 0.51 |
|  | C133R021 | PREDICTED: hypothetical protein LOC565988 | 0.51 |
|  | C189R086 | zinc finger mym-type protein 1 | 0.51 |
|  | C112R052 | UNKNOWN | 0.51 |
|  | C150R029 | UNKNOWN | 0.51 |
|  | C006R060 | f-box only protein 42 | 0.51 |
|  | C176R043 | pwwp domain-containing protein mum1-like | 0.51 |
|  | C170R126 | UNKNOWN | 0.51 |
|  | C098R097 | wd and tetratricopeptide repeats protein 1 | 0.51 |
|  | C072R114 | spondin-2-like isoform x1 | 0.51 |
|  | C124R054 | c-c motif chemokine 25 precursor | 0.51 |
|  | C074R020 | cub and zona pellucida-like domain-containing protein 1-like | 0.51 |
|  | C173R056 | e3 ubiquitin-protein ligase itchy homolog | 0.51 |
|  | C157R055 | UNKNOWN | 0.51 |
|  | C021R155 | smoothelin-like protein 2-like | 0.51 |
|  | C090R149 | UNKNOWN | 0.51 |
|  | C240R162 | alpha cardiac muscle 1 | 0.51 |
|  | C039R086 | alpha-actinin-3-like isoform 1 | 0.51 |
|  | C118R101 | protein-tyrosine kinase 2-beta-like | 0.51 |
|  | C102R009 | UNKNOWN | 0.51 |
|  | C186R010 | eukaryotic peptide chain release factor subunit 1 | 0.51 |
|  | C060R027 | xeroderma complementation group a | 0.51 |
|  | C036R088 | zinc finger protein 654 | 0.51 |
|  | C043R036 | splicing factor 3b subunit 1 | 0.51 |
|  | C185R063 | UNKNOWN | 0.51 |
|  | C228R080 | neuroligin 3b | 0.51 |
|  | C081R054 | UNKNOWN | 0.51 |
|  | C092R028 | transmembrane protease serine 9-like | 0.51 |
|  | C010R067 | UNKNOWN | 0.51 |
|  | C103R092 | rotatin | 0.51 |
|  | C160R103 | beta-2-glycoprotein 1-like | 0.51 |
|  | C170R129 | atp-dependent dna helicase q4 | 0.51 |
|  | C065R036 | acyl- synthetase family member mitochondrial-like | 0.50 |
|  | C164R024 | mpv17-like | 0.50 |
|  | C153R058^‡^ | dep domain-containing protein 1a isoform 2 | 0.50 |
|  | C013R120 | zinc finger protein 703-like | 0.50 |
|  | C001R131 | UNKNOWN | 0.50 |
|  | C004R077 | UNKNOWN | 0.50 |
|  | C116R071 | calcium calmodulin-dependent protein kinase type ii subunit gamma | 0.50 |
|  | C103R057 | cytochrome c oxidase subunit 4 isoform mitochondrial precursor | 0.50 |
|  | C116R024 | gonadotropin-releasing hormone | 0.50 |
|  | C128R074 | perilipin-2 isoform 1 | 0.50 |
|  | C260R051 | gig2-like protein | 0.50 |
|  | C021R029 | UNKNOWN | 0.50 |
|  | C079R134 | UNKNOWN | 0.50 |
|  | C052R013 | titin isoform n2-a | 0.50 |
|  | C184R133 | inhibitor of growth protein 4 | 0.50 |
|  | C069R058 | UNKNOWN | 0.50 |
|  | C068R137 | UNKNOWN | 0.50 |
|  | C124R049 | UNKNOWN | 0.50 |
|  | C164R085 | prolyl endopeptidase-like | 0.50 |
|  | C095R138 | UNKNOWN | 0.50 |
|  | C125R152 | UNKNOWN | 0.50 |
|  | C208R018 | transmembrane protein 111-like | 0.50 |
|  | C159R163 | chromodomain y-like protein | 0.50 |
|  | C161R062 | retinoic acid receptor rxr-gamma-b | 0.50 |
|  | C132R109 | serine threonine-protein kinase plk4-like | 0.50 |
|  | C121R134 | rab5 gdp gtp exchange factor | 0.50 |
|  | C044R160 | UNKNOWN | 0.50 |
|  | C129R110 | dual specificity protein phosphatase 26-like | 0.50 |
|  | C010R119 | hyaluronidase-2 isoform x1 | 0.50 |
|  | C127R105 | actin-related protein 2 3 complex subunit 2 | 0.50 |
|  | C075R161 | carbohydrate sulfotransferase 14 | 0.50 |
|  | C155R129 | protein asteroid homolog 1 | 0.50 |
|  | C108R133 | alpha-ketoglutarate-dependent dioxygenase alkb homolog 2 | 0.50 |
|  | C132R062 | protein-tyrosine kinase 2-beta isoform 2 | 0.50 |
|  | C119R170 | deoxyribonuclease lysosomal | 0.50 |
|  | C174R024 | UNKNOWN | 0.50 |
|  | C075R013 | rad52 motif-containing protein 1 | 0.49 |
|  | C201R124 | sumo-activating enzyme subunit 2 | 0.49 |
|  | C183R130 | UNKNOWN | 0.49 |
|  | C106R087 | myocilin-like | 0.49 |
|  | C210R025 | UNKNOWN | 0.49 |
|  | C264R138 | nucleosome-remodeling factor subunit bptf | 0.49 |
|  | C016R136 | ctp synthase 1-like | 0.49 |
|  | C133R064 | spindle assembly abnormal protein 6 homolog | 0.49 |
|  | C161R081 | atp-binding cassette sub-family b member 9-like | 0.49 |
|  | C115R132 | nadh dehydrogenase | 0.49 |
|  | C175R061 | cytochrome c oxidase polypeptide vic-2 | 0.49 |
|  | C092R082 | 40s ribosomal protein s25 | 0.49 |
|  | C196R149 | UNKNOWN | 0.49 |
|  | C118R159 | UNKNOWN | 0.49 |
|  | C128R130 | dystrobrevin beta-like | 0.49 |
|  | C237R008 | protein casc5 | 0.49 |
|  | C208R108 | UNKNOWN | 0.49 |
|  | C003R068 | UNKNOWN | 0.49 |
|  | C169R125 | nucleoprotein tpr | 0.49 |
|  | C134R047 | cd8 beta | 0.49 |
|  | C260R032^§^ | t-cell surface glycoprotein cd5-like | 0.49 |
|  | C206R039 | UNKNOWN | 0.49 |
|  | C128R152 | protein-tyrosine sulfotransferase 1-like | 0.48 |
|  | C002R051 | sestrin-3 isoform 2 | 0.48 |
|  | C089R049 | gamma-glutamyl hydrolase-like | 0.48 |
|  | C099R074 | activating transcription factor 7-interacting protein 1 | 0.48 |
|  | C032R088 | pyruvate kinase | 0.48 |
|  | C113R033 | protein sgt1 isoform 1 | 0.48 |
|  | C138R018 | serine threonine-protein phosphatase 2a 55 kda regulatory subunit b delta isoform-like | 0.48 |
|  | C110R134 | UNKNOWN | 0.48 |
|  | C128R017 | caspase recruitment domain-containing protein 11-like | 0.48 |
|  | C004R110 | deleted in lung and esophageal cancer protein 1 | 0.48 |
|  | C049R164 | UNKNOWN | 0.48 |
|  | C133R087 | caveolin-3 | 0.48 |
|  | C075R034 | UNKNOWN | 0.48 |
|  | C014R093 | UNKNOWN | 0.48 |
|  | C025R008 | tropomyosin 4 | 0.48 |
|  | C123R009 | immunoglobulin light chain | 0.48 |
|  | C183R063 | UNKNOWN | 0.48 |
|  | C155R046 | arf-gap with sh3 ank repeat and ph domain-containing protein 3 | 0.48 |
|  | C132R066 | coiled-coil domain-containing protein 97 | 0.48 |
|  | C250R023 | probable phospholipid-transporting atpase vd-like | 0.48 |
|  | C066R041 | 5-aminolevulinate mitochondrial | 0.48 |
|  | C143R143 | 40s ribosomal protein s2-like | 0.48 |
|  | C094R169 | myomesin-1 isoform 1 | 0.48 |
|  | C207R077 | UNKNOWN | 0.48 |
|  | C260R027 | tetratricopeptide repeat protein 39a-like | 0.48 |
|  | C226R069 | myosin light chain 2 | 0.48 |
|  | C143R060 | UNKNOWN | 0.48 |
|  | C173R073 | gamma-glutamyltransferase 5-like | 0.48 |
|  | C041R043 | transcription elongation factor spt6 | 0.48 |
|  | C093R078 | UNKNOWN | 0.47 |
|  | C064R027 | myogenic factor 5 | 0.47 |
|  | C149R077 | upf0583 protein c15orf59 homolog | 0.47 |
|  | C029R109 | myosin heavy chain | 0.47 |
|  | C023R138 | 60s ribosomal protein l29 | 0.47 |
|  | C184R049 | translation initiation factor eif-2b subunit delta-like | 0.47 |
|  | C083R061 | unnamed protein product | 0.47 |
|  | C100R025 | telomere-associated protein rif1 isoform x2 | 0.47 |
|  | C071R116 | UNKNOWN | 0.47 |
|  | C224R061 | clathrin light chain a | 0.47 |
|  | C128R037 | tyrosine--trna mitochondrial | 0.47 |
|  | C002R129 | myh9 protein | 0.47 |
|  | C141R157 | maltase- intestinal | 0.47 |
|  | C173R138 | cytochrome c oxidase polypeptide mitochondrial precursor | 0.47 |
|  | C006R012 | UNKNOWN | 0.47 |
|  | C030R054 | zinc finger protein | 0.47 |
|  | C075R143 | btb poz domain-containing adapter for cul3-mediated degradation protein 3 | 0.47 |
|  | C088R062 | zinc transporter zip11 isoform 1 | 0.47 |
|  | C138R161 | nadh dehydrogenase 1 beta subcomplex subunit mitochondrial precursor | 0.47 |
|  | C060R120 | centrosomal protein of 97 kda | 0.47 |
|  | C066R111 | interferon-induced very large gtpase 1-like | 0.47 |
|  | C098R038 | chicken-ii-type gonadotropin-releasing hormone precursor | 0.47 |
|  | C108R153 | complement factor d | 0.47 |
|  | C056R162 | UNKNOWN | 0.46 |
|  | C060R108 | cytochrome c oxidase subunit 2 | 0.46 |
|  | C223R122 | heat shock protein beta-7 | 0.46 |
|  | C051R058 | cornichon homolog 4 | 0.46 |
|  | C171R162 | ubiquitin-like modifier-activating enzyme 1-like | 0.46 |
|  | C031R021 | lumican | 0.46 |
|  | C145R100 | myosin heavy chain | 0.46 |
|  | C152R161 | UNKNOWN | 0.46 |
|  | C177R053 | suprabasin isoform 1 precursor | 0.46 |
|  | C247R088 | interferon-induced guanylate-binding protein 1 | 0.46 |
|  | C244R012 | t-box 1 | 0.46 |
|  | C110R061 | UNKNOWN | 0.46 |
|  | C125R129 | plastin-1 | 0.46 |
|  | C089R113 | UNKNOWN | 0.46 |
|  | C099R123 | phosducin-like protein | 0.46 |
|  | C135R026 | lysosome-associated membrane glycoprotein 2 | 0.46 |
|  | C106R081 | UNKNOWN | 0.46 |
|  | C227R065 | nadh dehydrogenase | 0.46 |
|  | C018R118 | fermitin family homolog 1 | 0.46 |
|  | C228R112 | upf0708 protein c6orf162 homolog | 0.46 |
|  | C131R148 | dpy-30 homolog | 0.46 |
|  | C169R138 | troponin skeletal muscle | 0.46 |
|  | C127R120 | retrograde golgi transport protein rgp1 homolog | 0.46 |
|  | C077R015 | UNKNOWN | 0.46 |
|  | C178R159 | alpha-aminoadipic semialdehyde dehydrogenase | 0.45 |
|  | C237R111 | camp-responsive element-binding 2 | 0.45 |
|  | C217R115 | tyrosinase | 0.45 |
|  | C118R138 | UNKNOWN | 0.45 |
|  | C012R013 | cardiac muscle alpha actin 1 | 0.45 |
|  | C085R042 | nance-horan syndrome | 0.45 |
|  | C079R135 | histone-lysine n-methyltransferase mll3 | 0.45 |
|  | C153R144 | twist-related protein 2-like | 0.45 |
|  | C230R143 | platelet endothelial cell adhesion molecule-like | 0.45 |
|  | C017R035 | polycomb group protein asxl1 | 0.45 |
|  | C088R069 | UNKNOWN | 0.45 |
|  | C247R090 | zinc finger swim domain-containing protein 5 | 0.45 |
|  | C241R022 | phosphatidylinositol-binding clathrin assembly protein | 0.45 |
|  | C226R122 | UNKNOWN | 0.45 |
|  | C264R063 | myosin va | 0.45 |
|  | C100R002 | matrix-remodeling-associated protein 8-like | 0.45 |
|  | C221R097 | far upstream element-binding protein 2-like | 0.45 |
|  | C164R069 | ubiquitin-conjugating enzyme e2 l3 | 0.45 |
|  | C213R107 | cell division control protein 6 homolog | 0.45 |
|  | C093R060 | tsc22 domain family protein 3 isoform 1 | 0.45 |
|  | C256R034 | lim-domain binding factor 3 | 0.45 |
|  | C123R152 | UNKNOWN | 0.44 |
|  | C218R084 | UNKNOWN | 0.44 |
|  | C030R120 | adhesive plaque matrix protein | 0.44 |
|  | C237R046^‡^ | prostaglandin e synthase 3 | 0.44 |
|  | C094R043 | diol dehydrogenase-like | 0.44 |
|  | C242R052 | trimethylguanosine synthase-like | 0.44 |
|  | C077R054 | transmembrane protein 131-like | 0.44 |
|  | C135R024 | atp synthase subunit mitochondrial | 0.44 |
|  | C093R055 | geranylgeranyl transferase type-2 subunit beta | 0.44 |
|  | C102R168 | vasorin-like | 0.44 |
|  | C008R059 | spore coat assembly protein | 0.44 |
|  | C214R061 | adhesive plaque matrix protein | 0.44 |
|  | C124R109 | UNKNOWN | 0.44 |
|  | C250R056 | transcriptional regulator atrx | 0.44 |
|  | C057R111 | cysteine and glycine-rich protein 3 | 0.44 |
|  | C100R097 | PREDICTED: hypothetical protein LOC557772 | 0.44 |
|  | C169R018 | UNKNOWN | 0.44 |
|  | C112R126 | gamma-glutamyltransferase 7 | 0.44 |
|  | C086R148 | mitogen-activated protein kinase kinase kinase kinase 5 | 0.44 |
|  | C007R148 | solute carrier family 15 member 2 | 0.44 |
|  | C039R146 | protein | 0.44 |
|  | C053R041 | protein vac14 homolog | 0.44 |
|  | C148R069 | autophagy-related protein 13 isoform 2 | 0.44 |
|  | C039R096 | myogenin | 0.44 |
|  | C232R083 | complement c4-1 | 0.44 |
|  | C038R111 | UNKNOWN | 0.44 |
|  | C007R073 | UNKNOWN | 0.44 |
|  | C208R163 | UNKNOWN | 0.44 |
|  | C135R016 | casp8-associated protein 2 | 0.44 |
|  | C073R003 | zinc finger protein 52 | 0.44 |
|  | C075R016 | neurofilament heavy polypeptide-like isoform x2 | 0.44 |
|  | C062R056 | rna binding protein fox-1 homolog 1-like | 0.44 |
|  | C099R024 | ap-3 complex subunit delta-1 | 0.44 |
|  | C245R120 | clip-associating protein 2-like | 0.44 |
|  | C165R018 | ubiquitin carboxyl-terminal hydrolase 28-like | 0.44 |
|  | C118R074 | cytoskeleton-associated protein 5 | 0.43 |
|  | C133R095 | protein nlrc3-like | 0.43 |
|  | C009R091 | fibulin-1 precursor | 0.43 |
|  | C166R034 | UNKNOWN | 0.43 |
|  | C098R120 | acyl- synthetase family member mitochondrial-like | 0.43 |
|  | C142R152 | UNKNOWN | 0.43 |
|  | C179R010 | light chain 12 | 0.43 |
|  | C084R169 | a2 protein | 0.43 |
|  | C064R015 | UNKNOWN | 0.43 |
|  | C034R158 | UNKNOWN | 0.43 |
|  | C194R062 | mitochondrial nadh-ubiquinone oxidoreductase 75 kda subunit | 0.43 |
|  | C085R026 | atp-dependent rna helicase dhx8 | 0.43 |
|  | C071R076 | zz-type zinc finger-containing protein 3 | 0.43 |
|  | C213R128 | asc-type amino acid transporter 1-like | 0.43 |
|  | C037R103 | dysferlin-interacting protein 1-like | 0.43 |
|  | C013R125 | glutathione peroxidase 4a | 0.43 |
|  | C199R157 | UNKNOWN | 0.43 |
|  | C128R044 | PREDICTED: uncharacterized protein C1orf112 homolog isoform X1 | 0.43 |
|  | C232R118 | rna-binding protein fus | 0.43 |
|  | C082R108 | protein | 0.43 |
|  | C105R010 | endothelial lipase-like | 0.43 |
|  | C244R017 | cysteine and glycine-rich protein 3 | 0.43 |
|  | C203R027 | complement component c1q receptor | 0.43 |
|  | C167R106 | UNKNOWN | 0.43 |
|  | C172R117 | calcineurin b homologous protein 2 | 0.42 |
|  | C088R014 | UNKNOWN | 0.42 |
|  | C208R126 | UNKNOWN | 0.42 |
|  | C114R088 | alpha-actinin-2 isoform 2 | 0.42 |
|  | C107R024 | hypothetical protein BRAFLDRAFT_86387 | 0.42 |
|  | C098R103 | fibroblast growth factor 1 | 0.42 |
|  | C241R059 | matrilin-2 precursor | 0.42 |
|  | C121R028 | membrane spaning protein | 0.42 |
|  | C052R163 | shc sh2 domain-binding protein 1 | 0.42 |
|  | C122R054 | serine threonine-protein kinase d3 | 0.42 |
|  | C020R060 | nck-associated protein 1-like | 0.42 |
|  | C135R061 | immunoglobulin superfamily member 10 isoform 1 | 0.42 |
|  | C048R082 | leukotriene b4 receptor | 0.42 |
|  | C120R159 | dynein heavy chain | 0.42 |
|  | C007R144 | mhc class i | 0.42 |
|  | C252R059 | hermansky-pudlak syndrome 3 protein | 0.42 |
|  | C252R157 | troponin slow skeletal and cardiac muscles | 0.42 |
|  | C057R043 | disintegrin and metalloproteinase domain-containing protein 33 | 0.42 |
|  | C242R149 | axin-2-like | 0.42 |
|  | C179R108 | c-c motif chemokine 20 precursor | 0.42 |
|  | C200R008 | parvalbumin-7-like isoform x1 | 0.42 |
|  | C040R096 | UNKNOWN | 0.42 |
|  | C079R047 | smad nuclear-interacting protein 1 | 0.42 |
|  | C107R158 | fibronectin type iii and spry domain-containing protein 2 | 0.42 |
|  | C052R071 | wiskott-aldrich syndrome protein | 0.42 |
|  | C178R138 | UNKNOWN | 0.42 |
|  | C127R060 | UNKNOWN | 0.42 |
|  | C008R108 | cathepsin l | 0.41 |
|  | C023R031 | mhc class ii alpha partial | 0.41 |
|  | C097R152 | g protein pathway suppressor 2 | 0.41 |
|  | C004R141 | UNKNOWN | 0.41 |
|  | C050R077 | probable e3 ubiquitin-protein ligase herc6-like | 0.41 |
|  | C103R023 | arachidonate 5-lipoxygenase-activating protein | 0.41 |
|  | C139R165 | actinin alpha 2 | 0.41 |
|  | C009R143 | mucin 11a | 0.41 |
|  | C204R163 | 40s ribosomal protein s6 | 0.41 |
|  | C137R043 | UNKNOWN | 0.41 |
|  | C233R067 | protein dpy-30 homolog | 0.41 |
|  | C114R022 | novel protein vertebrate nebulin | 0.41 |
|  | C001R096 | tata box-binding protein-associated factor rna polymerase i subunit a | 0.41 |
|  | C226R125 | isoform cra_a | 0.41 |
|  | C227R164 | paired amphipathic helix protein sin3a-like | 0.41 |
|  | C071R019 | t-complex protein 1 subunit theta | 0.41 |
|  | C135R052 | l _3-like | 0.41 |
|  | C113R119 | UNKNOWN | 0.41 |
|  | C160R142 | caveolin-2-like | 0.40 |
|  | C049R146 | cyclic amp-dependent transcription factor atf-1 | 0.40 |
|  | C197R155 | UNKNOWN | 0.40 |
|  | C128R051 | transcription intermediary factor 1-alpha | 0.40 |
|  | C004R093 | arf-gap with coiled- ank repeat and ph domain-containing protein 3-like | 0.40 |
|  | C098R094 | UNKNOWN | 0.40 |
|  | C007R060 | oocyte zinc finger protein 20 | 0.40 |
|  | C177R167 | UNKNOWN | 0.40 |
|  | C041R018 | u2 snrnp-associated surp motif-containing protein | 0.40 |
|  | C017R036 | differentially expressed in fdcp 6 homolog | 0.40 |
|  | C116R033 | kelch repeat and btb domain-containing protein 12-like | 0.40 |
|  | C155R032 | suppression of tumorigenicity 5 | 0.40 |
|  | C046R055 | dapper homolog 1 isoform 2 | 0.40 |
|  | C007R146 | coatomer subunit alpha | 0.40 |
|  | C137R001 | UNKNOWN | 0.40 |
|  | C169R117 | elongator complex protein 4 | 0.40 |
|  | C211R035 | phosphatase and actin regulator 4 | 0.40 |
|  | C221R048 | upf0193 protein evg1 isoform 2 | 0.40 |
|  | C243R086 | UNKNOWN | 0.40 |
|  | C246R022 | inter-alpha-trypsin inhibitor heavy chain h3-like | 0.40 |
|  | C252R013 | c14orf159 protein | 0.39 |
|  | C122R078 | hypothetical protein | 0.39 |
|  | C015R062 | ependymin precursor | 0.39 |
|  | C065R038 | nadh dehydrogenase subunit 3 | 0.39 |
|  | C125R078 | svil protein | 0.39 |
|  | C107R140 | importin-13 | 0.39 |
|  | C090R130 | son of sevenless homolog 1 | 0.39 |
|  | C136R130 | telomeric repeat-binding factor 2 | 0.39 |
|  | C082R133 | UNKNOWN | 0.39 |
|  | C264R127 | UNKNOWN | 0.39 |
|  | C260R055 | myosin binding protein cardiac | 0.39 |
|  | C135R056 | actin-binding rho-activating | 0.39 |
|  | C223R078 | UNKNOWN | 0.39 |
|  | C217R085 | UNKNOWN | 0.39 |
|  | C096R165 | immunoglobulin mu heavy chain | 0.38 |
|  | C064R028 | dual specificity testis-specific protein kinase 2 | 0.38 |
|  | C126R058 | UNKNOWN | 0.38 |
|  | C061R059 | protein kinase c and casein kinase substrate in neurons protein 2-like | 0.38 |
|  | C191R135 | integrin alpha-iib | 0.38 |
|  | C003R145 | suppressor of cytokine signaling 5 | 0.38 |
|  | C157R060 | ataxin-2-like protein | 0.38 |
|  | C204R112 | UNKNOWN | 0.38 |
|  | C221R134 | inter-alpha-trypsin inhibitor heavy chain h3-like | 0.38 |
|  | C137R106 | UNKNOWN | 0.38 |
|  | C206R009 | UNKNOWN | 0.38 |
|  | C194R166 | protein nlrc3-like | 0.38 |
|  | C129R111 | ras association domain-containing protein 10-like | 0.38 |
|  | C067R162 | cyclic amp-dependent transcription factor atf-4 | 0.38 |
|  | C005R137 | UNKNOWN | 0.37 |
|  | C141R081 | protein nlrc3-like | 0.37 |
|  | C110R127 | transcription initiation factor tfiid subunit 1-like | 0.37 |
|  | C047R107 | lymphocyte-specific protein tyrosine kinase | 0.37 |
|  | C156R075 | nadh dehydrogenase subunit 4 | 0.37 |
|  | C230R159 | secreted phosphoprotein 24 | 0.37 |
|  | C229R120 | centrosomal protein of 135 kda | 0.37 |
|  | C040R069 | ubiquitin carboxyl-terminal hydrolase 4 isoform 1 | 0.37 |
|  | C215R093 | zinc finger mym-type protein 4-like | 0.37 |
|  | C260R107 | rho gtpase-activating protein 24 | 0.37 |
|  | C002R144 | myoblast determination protein 2 | 0.37 |
|  | C252R090 | myoblast determination protein 2 | 0.37 |
|  | C131R142 | mitochondrial import inner membrane translocase subunit tim16 | 0.37 |
|  | C118R069 | UNKNOWN | 0.37 |
|  | C198R110 | rrna methyltransferase mitochondrial | 0.36 |
|  | C128R160 | creatine kinase | 0.36 |
|  | C254R062 | inter-alpha-trypsin inhibitor heavy chain h3 | 0.36 |
|  | C003R151 | cysteine and glycine-rich protein 3 | 0.36 |
|  | C025R040 | homeobox protein pknox1 | 0.36 |
|  | C099R030 | pogo transposable element with krab domain-like | 0.36 |
|  | C102R169 | probable e3 ubiquitin-protein ligase rnf144a-a | 0.36 |
|  | C186R121 | complement c4-like | 0.35 |
|  | C212R169 | swi snf-related matrix-associated actin-dependent regulator of chromatin subfamily b member 1 isoform 1 | 0.35 |
|  | C118R056 | tyrosine-protein kinase fer- partial | 0.35 |
|  | C242R008 | UNKNOWN | 0.35 |
|  | C238R137 | bchain human vinculin head (1-258) in complex with human vinculin tail (879-1066) | 0.35 |
|  | C193R039 | syntaxin-12 | 0.35 |
|  | C178R019 | UNKNOWN | 0.35 |
|  | C263R059 | ubiquitin-associated protein 2 | 0.35 |
|  | C077R041 | yth domain family protein 2-like | 0.34 |
|  | C120R150 | sh2 domain-containing protein 1a | 0.34 |
|  | C063R031 | ras gtpase-activating-like protein iqgap3-like | 0.33 |
|  | C107R161 | cathepsin l | 0.33 |
|  | C160R062 | mothers against decapentaplegic homolog 1 | 0.33 |
|  | C233R135 | myosin heavy chain | 0.33 |
|  | C147R129 | UNKNOWN | 0.33 |
|  | C085R007 | PREDICTED: hypothetical protein LOC100690503 | 0.32 |
|  | C106R167 | chromosome 7 open reading frame isoform cra_b | 0.32 |
|  | C144R031 | 14-3-3 protein eta | 0.32 |
|  | C130R016 | UNKNOWN | 0.32 |
|  | C003R058 | msx2-interacting protein | 0.31 |
|  | C224R069 | tho complex subunit 5 homolog | 0.31 |
|  | C241R094 | inosine-5 -monophosphate dehydrogenase 2 | 0.31 |
|  | C206R142 | UNKNOWN | 0.31 |
|  | C052R097 | copine-8 | 0.31 |
|  | C074R049 | UNKNOWN | 0.30 |
|  | C101R089 | thimet oligopeptidase | 0.30 |
|  | C200R144 | UNKNOWN | 0.30 |
|  | C198R074 | protein | 0.30 |
|  | C169R047 | myocilin-like | 0.30 |
|  | C116R108 | mkl myocardin-like protein 1 | 0.30 |
|  | C148R140 | e3 ubiquitin-protein ligase synoviolin-like | 0.29 |
|  | C047R125 | UNKNOWN | 0.29 |
|  | C052R118 | UNKNOWN | 0.29 |
|  | C231R077 | structural maintenance of chromosomes protein 4 | 0.29 |
|  | C238R102 | beta-crystallin b3 | 0.29 |
|  | C016R060 | adp-ribosylation factor-binding protein gga1-like | 0.29 |
|  | C073R043 | tetraspanin-3-like isoform 1 | 0.28 |
|  | C050R043 | tetraspanin-18 | 0.28 |
|  | C136R001 | UNKNOWN | 0.28 |
|  | C132R074 | type-4 ice-structuring protein precursor | 0.28 |
|  | C010R106 | UNKNOWN | 0.27 |
|  | C050R154 | UNKNOWN | 0.27 |
|  | C193R122 | lymphocyte-specific protein tyrosine kinase | 0.26 |
|  | C044R040 | ubiquitin-associated protein 1-like | 0.26 |
|  | C140R061 | retinol-binding protein 2-like protein | 0.26 |
|  | C138R009 | UNKNOWN | 0.26 |
|  | C256R071 | leucine-rich repeat-containing protein 51 | 0.26 |
|  | C047R111 | myotilin isoform 1 | 0.25 |
|  | C147R072 | asph protein | 0.25 |
|  | C041R016 | acyl- -binding domain-containing protein 6 | 0.25 |
|  | C228R108 | ttd non-photosensitive 1 protein homolog | 0.25 |
|  | C094R067 | papain-like cysteine prorease | 0.25 |
|  | C011R090 | serine threonine-protein kinase pak 2-like | 0.24 |
|  | C116R075 | tho complex subunit 1 | 0.24 |
|  | C103R085 | e3 ubiquitin-protein ligase trim21-like | 0.24 |
|  | C211R037 | circumsporozoite protein | 0.23 |
|  | C019R022 | f-box only protein 48 | 0.23 |
|  | C113R135 | regulator of chromosome condensation | 0.23 |
|  | C020R131 | phosphatidylinositol transfer protein beta isoform isoform 2 | 0.22 |
|  | C152R152^a^ | ependymin-1 precursor | 0.20 |
|  | C115R074 | death-inducer obliterator 1 | 0.19 |
|  | C237R146 | catalase | 0.18 |
|  | C164R166 | UNKNOWN | 0.17 |
|  | C176R119 | apolipoprotein a-i precursor | 0.16 |
|  | C132R101 | replication factor c subunit 3 | 0.15 |
|  | C164R059 | protein | 0.11 |
|  | C133R140 | apolipoprotein a-i precursor | 0.09 |
|  | C251R080 | myosin regulatory light chain atrial isoform | 0.06 |
|  | C086R103 | myosin light polypeptide 4 | 0.04 |
| X35 | C139R113 | transposable element tcb1 transposase | 2.8 |
|  | C237R040^‡^ | PREDICTED: hypothetical protein LOC324610 | 2.58 |
|  | C211R110^‡^ | c4b-binding protein alpha chain precursor | 2.12 |
|  | C007R074 | zw10 interactor-like | 0.62 |
|  | C211R100 | tar dna-binding protein 43 | 0.54 |
|  | C059R123^‡^ | sperm-associated antigen 5 | 0.53 |
|  | C264R097 | magnesium-dependent phosphatase 1 | 0.49 |
|  | C153R058^‡^ | dep domain-containing protein 1a isoform 2 | 0.46 |
|  | C151R040 | UNKNOWN | 0.45 |
|  | C235R104 | nadh dehydrogenase subunit 5 | 0.44 |
|  | C018R137^‡^ | heterogeneous nuclear ribonucleoprotein l | 0.42 |
|  | C237R046^‡^ | prostaglandin e synthase 3 | 0.36 |
|  | C202R134 | protein regulator of cytokinesis 1 | 0.31 |

^*^Significantly differentially transcribed genes were annotated in Blast2GO using the BLASTx algorithm and the best BLASTx hit (e-value < 10^-6^) is presented.

^§^Genes differentially transcribed by juveniles from the enriched vs. unenriched environments in families X22 and X11.

^‡^Genes differentially transcribed by juveniles from the enriched vs. unenriched environments in families X35 and X11.

^a^While this gene was initially annotated as ependymin-1, further analyses suggest that contig C152R152 is chimeric
